# To Biotic or Abiotic: Biohybrid Systems for Artificial Photosynthesis

**DOI:** 10.1021/acs.chemrev.5c00963

**Published:** 2026-06-29

**Authors:** Yifat Cohen, Oren Bachar, Roy Cohen, Matan M. Meirovich, Omer Yehezkeli

**Affiliations:** † Faculty of Biotechnology and Food Engineering, 26747 Technion − Israel Institute of Technology, Haifa 3200003, Israel; ‡ The Resnick Sustainable Center for Catalysis, Technion − Israel Institute of Technology, Haifa 3200003, Israel; § The Nancy and Stephen Grand Technion Energy Program, Technion − Israel Institute of Technology, Haifa 3200003, Israel

## Abstract

Biotic-abiotic interfaced configurations hold great promise for application in renewable energy and artificial photosynthesis systems. Recent advances in synthetic biology, computational, and visualization techniques, along with enhanced high-resolution characterization, have enabled a deeper fundamental understanding of the interface, which, in turn, has improved electron transfer processes and the design architecture. These developed configurations open new routes to mimic the photosynthetic apparatus or add new applications based on biotic and abiotic catalytic reactions. Aiming to surpass natural systems, researchers have examined methods to reconfigure these block sets into new designs. This review focuses on the advances in artificial photosynthesis and coupled biotic-abiotic biohybrid systems. The work presents the development of artificial photosynthesis configurations aimed at generating light-induced energy or fuels. The use of natural photosynthetic proteins, inorganic photocatalysts, and advanced biohybrid materials is presented and discussed, aiming to enable future biotic-abiotic design and the ambitious goal of developing real-world applications.

## Graphical abstract

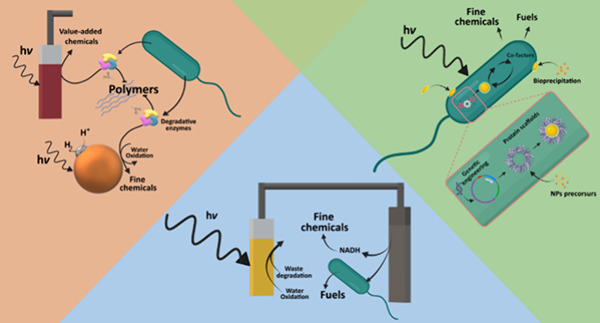

## 1 Introduction

The fundamental photosynthesis process that harnesses light energy evolved 3.5 billion years ago. The process altered Earth by enabling the conversion of light energy to chemical energy and the production of organic matter. The reaction also opened new routes for producing biological components that later emerged in living organisms.

Through evolution, the Z-scheme-based oxygenic process has been evolved. The improved efficiency of the Z-scheme apparatus has ushered in a new era on earth, in which oxygen serves as a key molecule, enabling the evolution of higher-level organisms. Oxygenic photosynthesis comprises the light-dependent and light-independent cycles. Those cycles harness visible light and facilitate the capture of CO_2_ for the production of carbohydrates. The central photosynthesis paradigm is based on two light-activated centers that enable an extremely high-efficiency light-induced charge separation. Both photosystems, Photosystem I (PSI) and Photosystem II (PSII), hold efficiencies reaching 100%.^1^ However, the overall efficiency of photosynthesis is much lower, estimated at 1–4%.^2^ During the last century, humankind has tried to harness the Sun’s irradiation as an ultimate and unlimited energy source. Since the discovery of semiconductors and, later, the development of photovoltaics, methodologies for directly converting light energy into electrical power have been developed and implemented. Those methodologies often require smart grid installation and control and must be coupled with energy storage devices or energy management apparatus. In parallel to photovoltaics, photoelectrochemical and dye-sensitized solar cell configurations were also developed.^3–5^ These cells generated electrical power or fuels, with light energy acting as the driving force.

Inspired by nature, Honda and co-workers developed the first light-induced photoelectrochemical cell, PEC, to enable full water splitting for O_2_ and H_2_. The cell was constructed with a TiO_2_-based photoanode, which has a wide bandgap and can be activated by UV light irradiation.This first demonstration sparked a critical scientific effort to develop improved configurations, aiming to convert light energy into electrical energy or produce fuels. In parallel, a crucial understanding of the photosynthesis apparatus was achieved by Feher and co-workers.^6^ These insights into the electron and proton transfer processes have led to the development of light-induced systems inspired by the natural Reaction Center. During the 50s to the 70s, a significant scientific effort led to the Z-scheme configurations comprising two reaction centers. These findings open the way for advanced devices with greater potential for various energy applications. Furthermore, Z-scheme configurations sparked scientific efforts to develop PECs using biomimetic approaches.

Over the last few decades, tremendous efforts have been made to develop PECs. These efforts have led to an enhanced stability, light absorption, power output, and catalytic processes. The latter were mainly focused on water oxidation and oxygen reduction; nevertheless, hydrogen evolution and CO_2_ and N_2_ reduction processes were also pursued. A key challenge in those processes is selectivity, and cocatalysts are frequently coupled with the photoanode or photocathode to prevent side reactions. The added cocatalyst promotes improved hole or electron transfer, which, in turn, enhances photooxidation or photoreduction, respectively. While the use of photoelectrodes has great promise, light-induced catalysis can be achieved using colloidal NPs. These nanoelements exhibit unique properties that are governed by their size and morphology. The quantum size effect dictates the properties of semiconductor-based nanomaterials, which can therefore be tuned by controlling their size. By tailoring the energy band positions at the nanoelements, one can control the absorption spectrum and the catalytic properties of the generated holes and electrons at the valence and conduction bands, respectively.

Furthermore, the NPs’ surface-to-volume ratio and their outstanding solubility in various solvents enable excellent mass transfer of the NPs or substrates, promoting reactions that could not be achieved using photoelectrochemistry. Alternatively, metallic nanoelements can also be used as catalysts. However, here, the metals’ plasmonic properties dictate the energy levels and the nanomaterial absorption properties. In many cases, these nanoelements have dimensions and photophysical properties similar to those of the photosynthetic proteins; therefore, they may be used to enhance photosynthetic apparatus activity or to replace its components.

The Z-scheme light-driven configuration regenerates two critical energy carriers: ATP and NADPH. These bioenergy molecules provide cells with the energy required for CO_2_ assimilation and other essential processes in organisms. In recent years, artificial photosynthesis configurations that can regenerate NADPH/NADH or ATP have been developed. These systems use chemical, electrochemical, photochemical, and photoelectrochemical apparatuses to produce energy-currency molecules. The photosynthesis process enables the critical transformation of inorganic CO_2_ into hydrocarbons and energy; however, it can also be used to generate other forms of energy such as electricity and hydrogen fuels.

Furthermore, one can harness the high reduction potential of the process or its energy currency molecules to generate fuels or to develop an enzymatic cascade for fine chemical production. Such cascades open a path to the production of enantiomerically pure compounds, which are highly desired in many processes, particularly in the pharmaceutical industry. Moreover, as enzymatic cascades can be utilized for waste degradation, these processes can lead to carbon-neutral fuel generation and are, therefore, routinely pursued.

Designing biotic-abiotic configurations toward artificial photosynthesis aims to utilize the advances of each discipline. However, those are often not easy to designate and are highly dependent on the configuration.^7^ For example, using natural photosynthetic proteins as light absorbers may lead to high quantum efficiencies. However, those photosensitizers are limited to specific wavelengths and degrade because of back reactions under high light intensities. In contrast, QDs can be tuned to absorb the entire visible spectrum; however, the light-induced electron-transfer process is poorly directional and lacks specificity, thereby frequently hindering the desired reaction. While some approaches put the natural elements in the foreground (photosynthetic proteins, photosynthetic organisms, and redox enzymes), others use them as external moieties to complement robust abiotic design.

Recent efforts have aimed to couple abiotic processes with biological processes to achieve higher efficiency, robustness, and versatility. This review will focus on these directions, the different approaches, opportunities, and current limitations in applications, and on improving understanding of the biotic and abiotic interfaces. Figure 1 exhibits the key scientific efforts toward biotic-abiotic and artificial photosynthesis systems. The blue panel exhibits photo­(bio)­electrochemical cells that convert light energy to chemical fuels. Light-driven photooxidation processes can facilitate water oxidation, mimicking the function of the photosystem II. Alternatively, the oxidation potential can be used for waste degradation. The coupled cathode enables the production of fuels or fine chemicals, facilitated by biocatalysts, such as enzymes or whole-cell bacteria. The orange panel illustrates the use of a biocatalyst to degrade polymeric waste into small molecules that, in turn, serve as sacrificial electron donors and can be further converted to value-added chemicals. Similarly, QD catalysts can be coupled with the photoinduced reactions for the production of fine chemicals while generating H_2_ fuel. The green panel presents approaches for constructing biotic-abiotic biohybrids for whole-cell artificial photosynthesis configurations, where added QDs or internally grown QDs are used to enhance biochemical processes.

**Figure 1:**
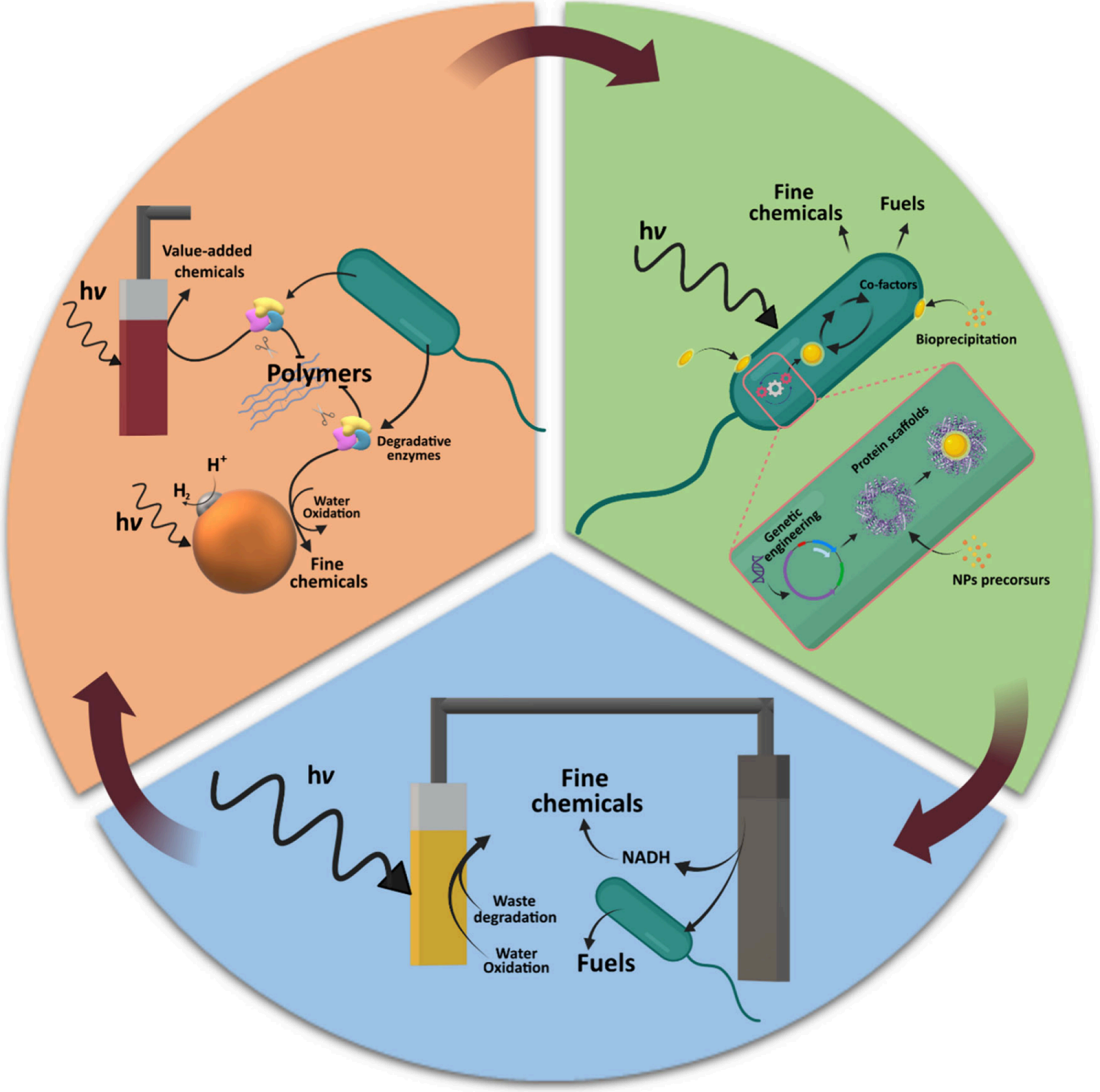
Schematic representation of the standard biotic-abiotic and artificial photosynthesis configurations.

Herein, we wish to present the latest research in artificial photosynthesis, aiming especially at biotic-abiotic configurations. These systems aim to address obstacles to efficient and sustainable artificial photosynthesis, focusing on photochemistry, bioengineering, and photoelectrochemistry. Each of these disciplines has advantages and disadvantages; therefore, a holistic approach enables enhanced activity that can even surpass the natural one. To that end, we first present and discuss artificial photosynthesis configurations that use natural photosynthetic proteins. Methodologies for establishing electrical communication between those proteins and abiotic elements, such as quantum dots, conductive polymers, and electrodes, are presented and discussed. Architecture is a critical factor enabling remarkable efficiency in natural photosynthesis. Therefore, methodologies to design scaffolds that enable light-induced electron transfer and charge separation are presented and discussed. We then dive deeper into the construction of biotic-abiotic configurations by presenting critical published research with a focus on methodologies for constructing photosynthesis-mimicry biohybrids *in vitro* and *in vivo*. Here, the ability to couple bricks from different toolboxes provides great flexibility for designing new reactions that utilize artificial photosensitizers in conjunction with enzyme cascades or whole-cell bacteria. The next section centers on (photo)­bioelectrochemical cells, their design, critical parameters, obstacles, and suitability for real-world artificial photosynthesis applications.

Furthermore, critical parameters, such as photoinduced water oxidation and charge separation, and the challenges that arise when they are coupled to oxygen-sensitive enzymes or bacteria are discussed. The scope was then extended to present different approaches for exploiting natural or synthetic polymers to produce electrical energy carriers and value-added chemicals. In a way, we present a reversal of the natural photosynthesis process that degrades polymers into energy and critical compounds. State-of-the-art configurations comprising biohybrid PECs or photochemical ones are presented and discussed. The last part of the review presents the essential parameters for quantitative evaluation of biotic-abiotic artificial photosynthesis systems. Section 11 finalized the review with the parameters needed to be addressed toward the real-world application based on biotic-abiotic and artificial photosynthesis configurations.

## 2 Photosynthesis Mimicry: Do and Undo

The engine of the photosynthetic paradigm is without doubt the photosystem II complex. The PSII protein complex comprises the Mn_4_CaO_5_ cluster, an inorganic complex that enables the water-oxidation reaction via five consecutive intermediate states, S0 through S4. The process facilitated by the oxygen-evolving complex, OEC, enables the oxidation of H_2_O, generating an electron transfer to the Q_b_ site and, in parallel, releasing O_2_. To achieve the strong oxidation conditions required for the process at the Mn_4_CaO_5_ cluster, light-absorption steps occur. By sequential excitations at the P680 reaction center, the essential energy is provided to shift the manganese ions into a higher oxidation state, enabling H_2_O oxidation. Since the OEC determination,^8^ efforts to construct stable and effective OEC complex analogs have been pursued.^9^ These efforts included a similar structure or function to the natural complexes,^10–15^ as well as the use of precious and novel metals or metal oxide layers that enable high turnover rates.^16–19^ While the developed complexes developed reached turnover frequencies that are similar to the natural process at the range of 100–400 s^–1^, the stability of those complexes at homogeneous and heterogeneous catalysis falls short. The designed complexes inspired by nature contributed tremendously to basic science understanding. Furthermore, biophysical techniques have been developed, making tremendous contributions to scientific progress. However, currently, thin-layer oxides or similar thin layers based on abundant metals such as cobalt, manganese, nickel, and iron seem more suitable for real-world applications with stable, continuous activation. Frequently, developed catalysts that present high turnover rates fall short in terms of robustness and long-term stability. In many reports, the determined parameter accounts for short measurements of seconds or minutes. Therefore, the catalyst’s aptness for applications cannot be concluded. While the catalyst’s structural and mechanistic understanding is of great importance for developing new complexes and configurations, an indication of the systems’ stability for at least 30 min should become standard practice in such scientific reports. Post analysis of the catalyst should also be included.

Developed catalysts can be activated chemically, photochemically, or photoelectrochemically. In the case of the last two mentioned, the developed water oxidation catalysts should be coupled with light-induced photosensitizers, electronic bridges, or redox mediators to enable the activation of the OEC. These systems should be designed to be bias-free and donor (or acceptor)-free, resembling the natural apparatus. In many cases, this is the hurdle that limits advancement into applications.

While some advances toward artificial photosynthesis have been made using different approaches, for example, an artificial leaf,^20^ decoupling oxygen and hydrogen production,^21^ and photochemical cells enabling full water splitting at a large scale,^22^ a commercialized system has not yet been fully introduced.

## 3 Photosynthetic Apparatus-Based Devices

### 3.1 Photosystem I-Based Configurations

#### 3.1.1 Conjugation of Proteins with Inorganic Catalysts

PSI comprises 12 or 13 subunits that form the active protein in cyanobacteria or plants, respectively.^23^ Naturally, the highly negative potential developed at the Fb iron–sulfur cluster, which can reach −0.75 V vs Ag/AgCl at pH 7, enables the reduction of ferredoxin or flavodoxin (−0.65 V). These diffusional proteins act as redox mediators to activate the ferredoxin NADP­(+) reductase (FNR) enzyme. The latter generates an NADPH flux, which triggers the Calvin cycle. The electron transfer chain has been optimized and evolved to enable an internally efficient electron transfer process at the protein core, starting at the excited P700 and ending at the Fb iron sulfur cluster.^24^ The extremely high reducing power developed by the light reaction can be exploited to enable a variety of alternative chemical reactions or electron-transfer processes that can benefit humankind. Thus, a significant research effort has been focused on redirecting the electron flux to generate electrical or fuel energy.

As presented, the Photosystem I light-harvesting complex has attracted significant effort toward its use as a photosensitizer in biohybrid systems. In 2001, photosystem I was first coupled with a Pt cluster/NP to enable direct hydrogen generation. Millsaps and co-workers have photoirradiated isolated Photosystem I proteins in an oxygen-free environment. Alternatively, platinum salts were added in the presence of ascorbate and plastocyanin, as an electron donor and a redox mediator, respectively, to oxidized P700. The excited PSI exhibits a high reducing potential and a unique electron-transfer chain that enables the site-specific reduction of platinum salts in proximity to the Fb site. The formed PSI-Pt NP biohybrid was then irradiated in the presence of a sacrificial electron donor, enabling a similar electron-transfer process; however, the Pt catalyst facilitated hydrogen generation. This pioneering work presented the great promise of PSI-NP hybrids as a H_2_ fuel generation apparatus.^25^ Since the first demonstration, different approaches have been used to enable the conjugation of Pt or gold NPs with photosystem I. Golbeck and co-workers presented a unique approach where a molecular wire interfaced gold or Pt NPs with the Fb Fe-P cluster.^26^ The interface configurations have led to hydrogen generation. The same group has reported different approaches to conjugate NPs in proximity to the Fb site, leading to hydrogen generation.^27^ Reducing a Pt cluster or interface Pt NPs at the Fb site was also demonstrated in follow-up research. Utschig and co-workers investigated the formation of PSI-Pt NP biohybrids and their photocatalytic hydrogen generation.^28,29^ Bruce and co-workers have shown the use of a PSI-Pt cluster for hydrogen generation.^30^ In their work, Cyt C6 was utilized as an electron redox mediator, which in turn enabled the Pt salt reduction and the hydrogen generation as schematically presented in Figure 2.

**Figure 2:**
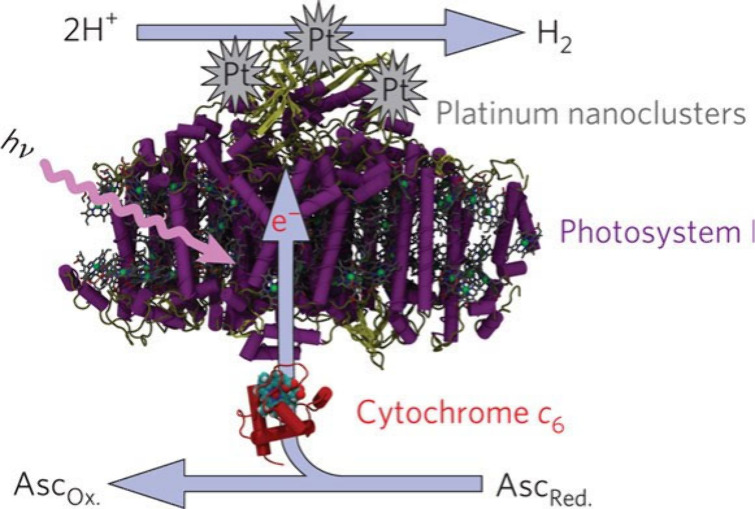
Schematic of the electron flow in the photosystem I catalytic nanoparticle. The monomeric form of the *T. elongatus* PSI is shown with chlorophyll cofactors (magnesium ligated in macrocycle shown in green van der Waals radius) and the protein colored by secondary structure (helical regions in purple, beta sheets in yellow, and unstructured regions in brown). A partially docked form of the cyt *c*_6_ is shown, providing the rereduction of P_700_, deriving electrons from the oxidation of ascorbate (Asc_Red._ → Asc_Ox._ + e^–^). The platinum clusters on the stromal surface of PSI are shown as gray stars catalyzing the reduction of protons to hydrogen with electrons of lower potential, using the energy of the absorbed photon. Reproduced with permission from ref 30. Copyright Springer 2010 Nature.

Although extensive research has been conducted, the exact positions of the Pt clusters or NPs at the PSI have not been fully determined. Recently, Gisriel and co-workers investigated a biohybrid structure to position Pt NPs at the PSI surface. The work provided significant evidence derived from cryo-EM imaging that two different sites can accommodate the formed NPs in a parallel manner. The research suggests that no perturbation to the PSI machinery occurs due to the biohybrid formation. Moreover, while two NP sites were found, one positioned in proximity to the Fd binding site, and another at an alternate position,^31^ only the larger NPs in proximity to the Fb Fe-S cluster act as catalysts for the H_2_ generation. While most of the formed PSI-NP biohybrids were designed for hydrogen generation, other interesting directions were also explored. For example, the use of Au NPs or photosensitizers^32^ as a light-absorbing antenna, where the NPs’ plasmonic properties or the dye enabled light-induced energy transfer.^33^

As mentioned, unlike PSII, PSI is highly stable and can maintain its activity for months if continually supplied with electron donors and acceptors.^34^ Therefore, a photodriven configuration for hydrogen generation using a biohybrid comprising the PSI light-harvesting protein and a metal cluster or an NP holds great promise. The use of renewable, environmentally friendly materials stands out as an alternative to chemically synthesized photosensitizers such as QDs. It should be mentioned that the high purification cost of the protein complex from plants or cyanobacteria may limit its use in real-world applications. Furthermore, in most of the research described, ascorbic acid was used as a sacrificial electron donor. While ascorbic acid is a well-known and efficient sacrificial electron donor, alternative chemicals should be explored, especially side stream chemicals that accumulate in industrial or household waste processes. Using such compounds may offer economic and environmental advantages, paving the way for real-world applications. Alternatively, Kim and colleagues have developed a biotic-abiotic configuration in which BiVO_4_ particles were modified with gold clusters, which served as linkers to couple the PSI-Pt electronically.^35^ The integrated system mimicked the natural Z-scheme configuration, where BiVO_4_ acts as an alternative to the natural PSII and facilitates light-induced water oxidation. At the same time, the PSI enables proton reduction to hydrogen fuel, as shown in Figure 3.

**Figure 3:**
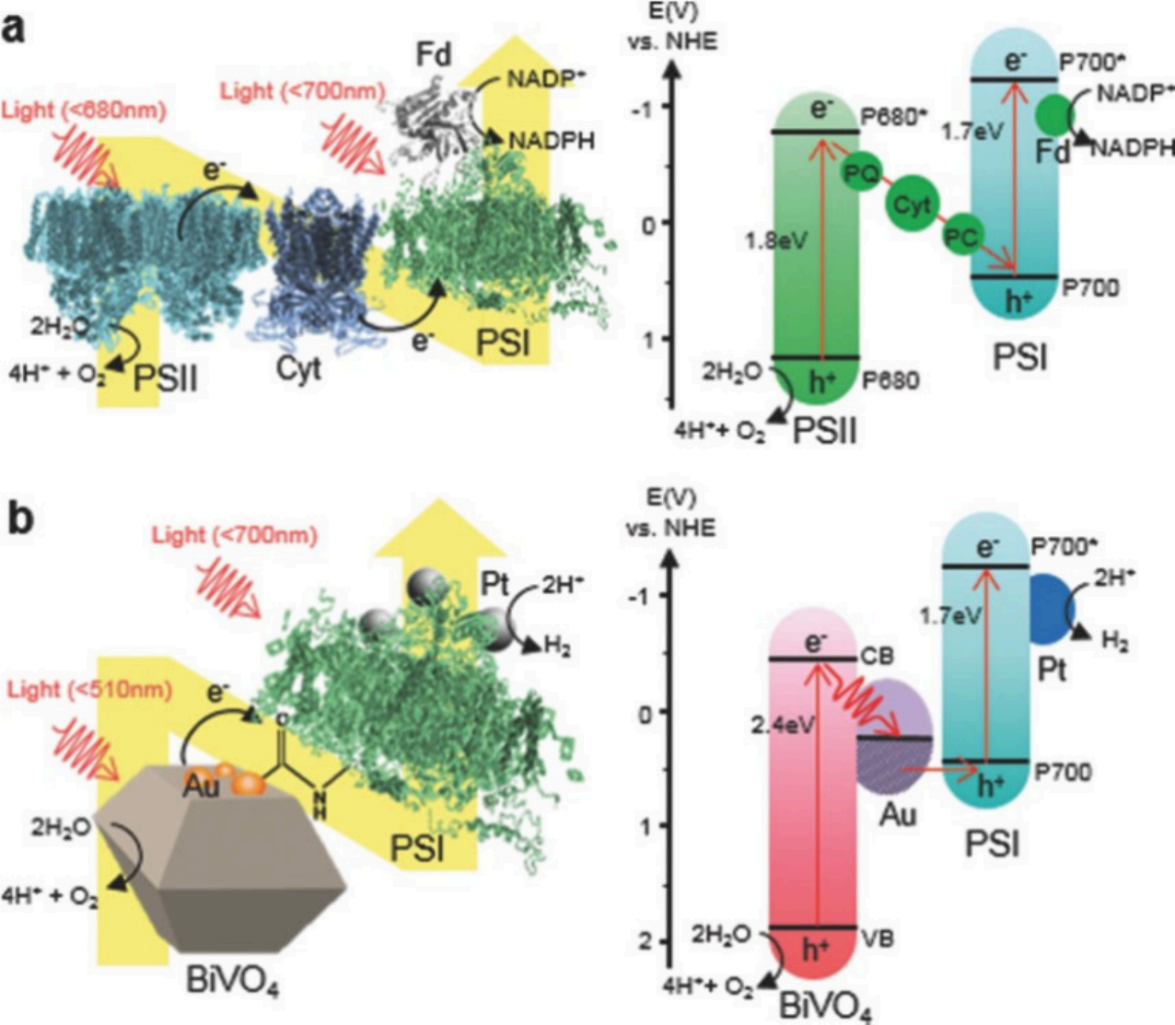
a) Natural Z-scheme and its energy diagram; photosynthesis follows serial reactions: oxidation of water and electron excitation in PSII, electron transfer through the electron transport chain (plastoquinone (PQ), cytochrome c6 (cyt), plastocyanin (PC)), electron excitation in PSI, and reduction of NADP in ferredoxin-NADP+ reductase (FNR). b) Hybrid Z-scheme and its energy diagram (mediator is Au). Photosynthetic water splitting follows serial reactions: oxidation of water and electron excitation in BiVO_4_, electron transfer through Au, electron excitation in PSI, and reduction of hydrogen catalyzed by Pt. Reproduced with permission from ref 35. Copyright 2015 Wiley-VCH GmbH.

#### 3.1.2 Electrochemical Activation of Photosystem I-Based Devices

The PSI P700 acts as a photosensitizer and upon light excitation facilitates the transfer of high-energy electrons. These short-lived excited states undergo charge separation through the PSI protein’s redox-active acceptor chain.^1^ The highly efficient charge separation minimizes back reactions and the recombination process.^36^ It also develops an extraordinary potential gap between oxidized P700 and the Fb acceptor side. The developed potential reaches 1 V in the small dimensions of a 7 nm protein.^23,24^ The reduced Fb site exhibits a strong reducing potential of −0.8 V vs Ag/AgCl, which, thermodynamically, can be used to reduce any natural biological process. Naturally, the electrons are transferred from the Fb Fe-S cluster to ferredoxins or flavodoxins. However, PSI can also be used for unorthodox reduction processes. Alternatively, to the natural electron-transfer process, under irradiation, the PSI can facilitate anodic or cathodic photocurrents. Generating power by activating an environmentally friendly photosynthesizer has great promise for sustainable energy-producing devices and should therefore be pursued.^37–39^

Toward this end, several prerequisites should be considered:

(i) The PSI protein shell is an insulator. The PSI contains redox centers that enable efficient internal electron transfer. However, it allows specific routes to be externally accessed through the P700 or the F_ab_ sites. Therefore, an electronic bridge with a short electron transfer pathway from the protein to the electrode (or vice versa) should be developed. Such bridges can facilitate short electron-transfer processes to electron sinks or donors, respectively.
(ii) To prevent short circuits, the protein redox centers are embedded within the protein structure with only the Fab sites and the P700 prone to electrical communication with their surroundings.
(iii) For an efficient electron-transfer process, methods to order and orient the PSI proteins’ P700 or Fab sites relative to the electrodes or acceptors are required.
(iv) The developed devices should provide clear paths for absorption of visible light irradiation.

Indeed, over the last two decades, extensive research has aimed to utilize PSI for electrical power generation. While PSI offers significant advantages, this research direction was first demonstrated with reaction centers that absorb light in the near-IR range.^40–43^ Those demonstrations ignited extensive research to couple isolated PSI proteins with electrodes, both anodes and cathodes^44^ to unlock their potential for light-induced anodic or cathodic photocurrent generation, respectively. Carmeli and colleagues have self-assembled the isolated protein on metal electrode surfaces. The assembled device showed potential buildup due to light irradiation.^45^ Cliffel and Jennings introduced a vacuum technique to improve the PSI self-assembly on the electrode surface.^46^ By increasing the electrode surface area and improving immobilization using dialdehyde cross-linker, they managed to enhance the generated photocurrents by 8 times to reach 800 nA/cm^2^.^47^ By applying consecutive layers of the PSI on top of the electrode surface, an increased cathodic photocurrent was reached.^48^ As mentioned in the previous section, metal clusters/NPs can be formed at the Fb site. These metal clusters enable the integration and orientation of the PSI proteins toward the electrode.^34,49^ While the PSI can be used for anodic or cathodic photocurrents, the latter yield higher currents and present high quantum efficiency, QE.^50^ Badura and co-workers have shown that an Os complex-based polymer, with a potential of ca. 0.2 V vs Ag/AgCl, can act as an efficient redox mediator for the PS1 oxidized P700. In parallel with electron transfer from the electrode to P700^+^, the generated electrons can be transferred to methyl viologen, an efficient diffusional redox mediator. The viologen radical reacts efficiently with atmospheric oxygen to form high cathodic photobioelectrocatalytic currents in the range of 29 μA/cm^2^ and Incident-Photon-to-Current Efficiency (IPCE) of 3.1%. As mentioned, the PSI redox-active centers are isolated to prevent short-circuiting and enable efficient charge separation. While charge separation limits back reactions, each electron-transfer process lowers the redox potential gradient. An elegant approach used by Terasaki involved replacing the natural VK1 quinone with a VK1 quinone analog. The analog holds a viologen end, which acts as an electron acceptor and a positive binding site for interacting with the negatively charged electrode surface,^51^ Figure 4.

**Figure 4:**
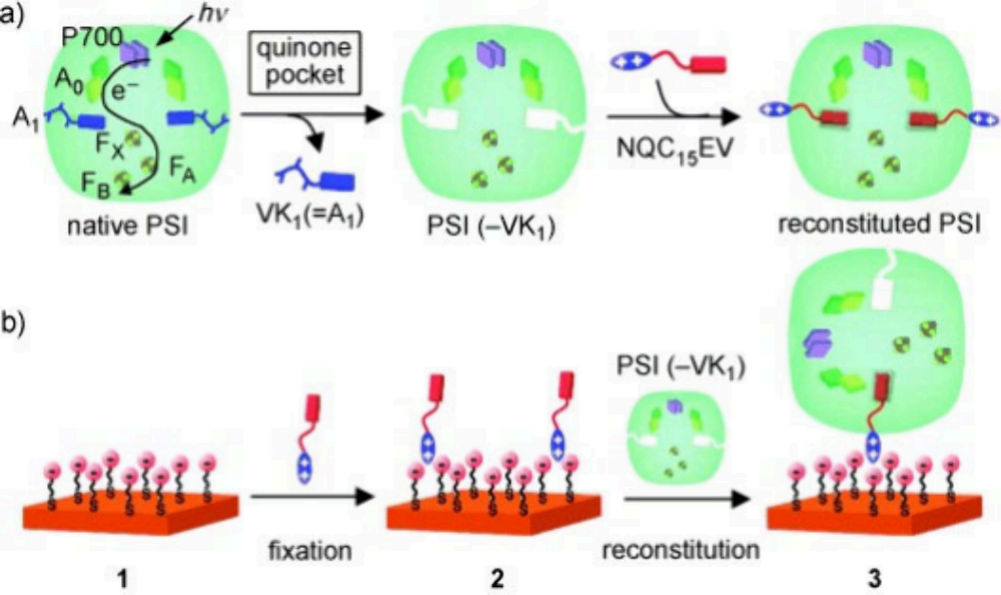
Schematic illustration of the procedures for the extraction of VK_1_ used to prepare a) a quinone pocket and b) reconstitution with a molecular wire adsorbed on a gold electrode. Reproduced with permission from ref 51. Copyright 2009 Wiley-VCH GmbH.

This approach enabled the extraction of electrons at a higher potential, about 300 mV, compared to the naturally extracted electrons’ potential from the Fab site. The use of site-specific redox mediators has a crucial effect on protein orientation relative to the electrodes. It also controls the distance between electron donors and acceptors, which dictates the rate of electron transfer. Biotechnological tools were also used to control PSI orientation with respect to the electrode. Mershin and co-workers overexpressed ZnO metal-binding peptide in proximity to the Fab redox centers. The ZnO photoanode was then modified with the bioengineered PSI to form a fully oriented PSI monolayer. This unique configuration enables the generation of a photocurrent of 362 μA/cm^2^ under Sun illumination and an applied bias of 0.5 V.^52^ The PSI-based photoanode was coupled with a cathode, which continuously reduced the cobalt phenanthroline complex, which acted as a redox mediator and enabled current flow initiated by the photoinduced reaction. Thus, the cell could operate continuously. The work nicely demonstrates the contribution of biotechnology, material science, and engineering toward enhanced performance.

Naturally, plastocyanin acts as an electron donor and Fd as an electron acceptor to enable the photoactivation of PSI and prevent a back-reaction. Therefore, artificial activation of the PSI should include these elements as well.

Manocchi and colleagues developed a PSI-based configuration in which the protein was self-assembled onto hydrocarbon monolayers (self-assembled monolayers, SAM) with different head groups, Figure 5a,b. They show that the negatively charged head enabled improved orientation on the electrode surface, thereby increasing the generated photocurrents. The work showed that both the electron donor (Os complex) and the electron acceptor (methyl viologen) are essential for the efficient electron transfer process, Figure 5c–f. As depicted in Figure 5f, it is crucial to adjust the redox mediator’s potential to the P700^+^ and the Fb Fe-S to enable correct electron flow.^53^ Furthermore, as will be discussed later, the thermodynamic parameters are essential; however, they are not consistently enough to facilitate such a reaction, and other parameters such as charge, size, affinity, and mobility should be considered.^54^

**Figure 5:**
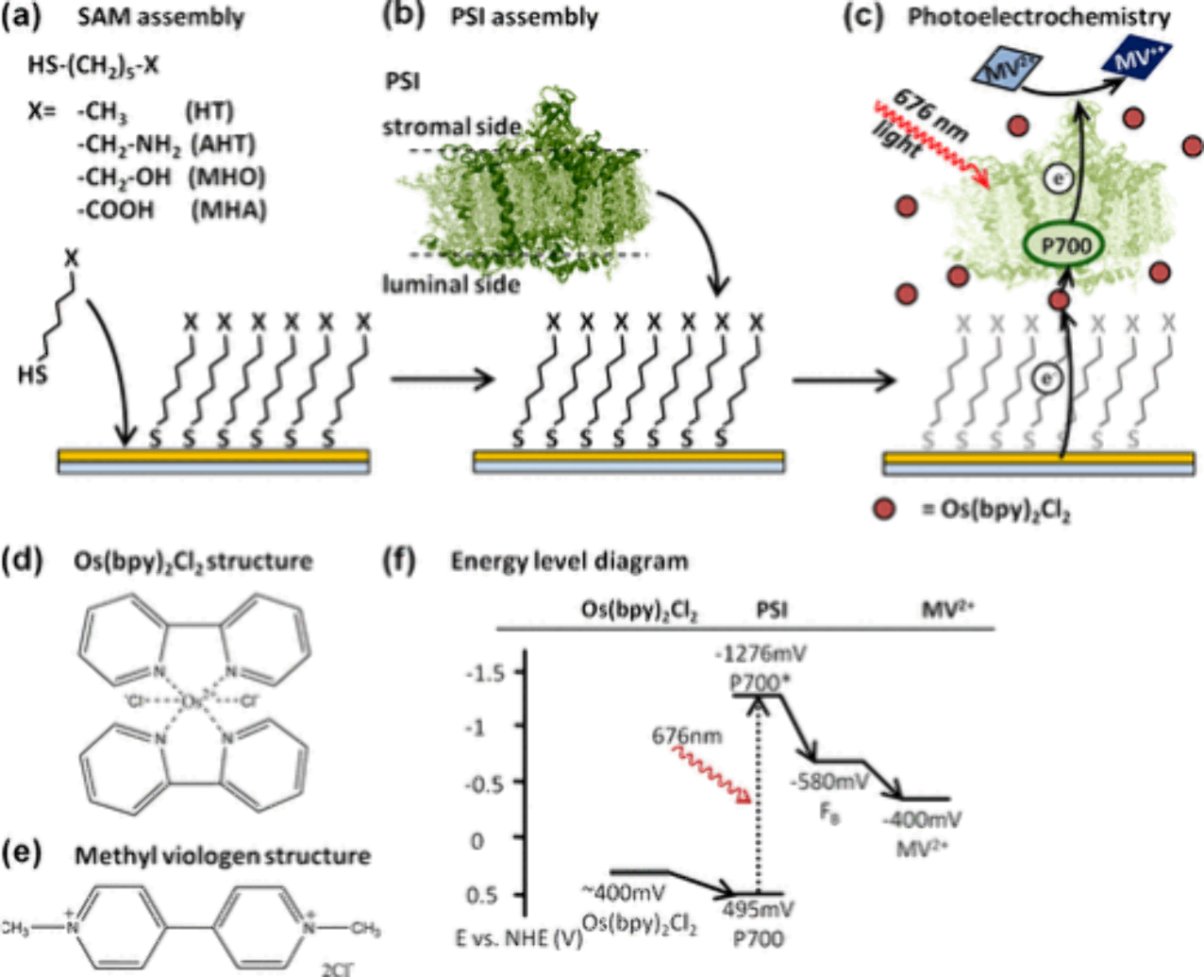
Schematic of PSI assembly on electrodes modified with alkanethiols (a) Gold electrodes are first modified with alkanethiols to form SAMs, followed by (b) PSI assembly. (c) Light-induced electron transfer associated with surface-assembled PSI on SAM-modified electrodes. Os­(bpy)_2_Cl_2_ and methyl viologen (MV^2+^) are used as redox mediators in solution. (d) Os­(bpy)_2_Cl_2_ and (e) MV^2+^ chemical structures. (f) Energy level diagram relating redox potentials of mediators and PSI. Adapted from ref 53. Copyright 2013 American Chemical Society.

The addition of redox-active mediators to shorten the electron-transfer distance between PSI redox centers and electrodes or oxygen indeed enhanced photocurrents and yields; however, fully integrated systems can minimize diffusion limitations, which is essential for improved bioelectrocatalytic processes. Therefore, redox-active polymers were examined, with a layer-by-layer assembly implemented. The PSI and polybenzylviologen (PBV) were deposited on an ITO electrode. The redox-active polymer enabled an improved electron transfer process from the photoexcited PSI photosynthetic proteins to the electrode while providing stabilizing structural support due to the strong electrostatic interaction between the charged electrode, the photosynthetic proteins, and the charge viologen polymer, Figure 6a.^44^ A layer-by-layer technique was used to integrate the PSI proteins on top of the negatively charged ITO electrode. Using a positively charged viologen-based polymer and the photosynthetic protein, a multilayer was formed. As depicted in Figure 6b, the ITO/PBV/PSI electrodes were further used for the photocurrent generation. Under oxygen-free conditions and with the addition of a sacrificial electron donor, anodic photocurrent correlated with PSI absorbance was measured as a function of the amount of layer deposited, as shown in Figure 6c. Alternatively, application of chopped light irradiation at 680 nm resulted in similar photocurrents, Figure 6d. Interestingly, omitting the electron donor-coupled system (DCPIP/ascorbic acid) led to the development of cathodic photobiocatalytic currents. In the presence of O_2_, a single layer or double layer produced cathodic photocurrents as presented in Figure 6d,e, respectively. The design configuration features a fully integrated biocathode that requires only an atmospheric oxygen for its activation. Using a similar approach, integrated PSI-based photocathodes were coupled to a photoanode to develop a Z-scheme-like configuration. A Photosystem II-based photoanode, as presented in Figure 7, or a BiVO_4_-based photoanode was coupled to the PSI-based photocathode for the construction of photobioelectrochemical cells.^55,56^

**Figure 6:**
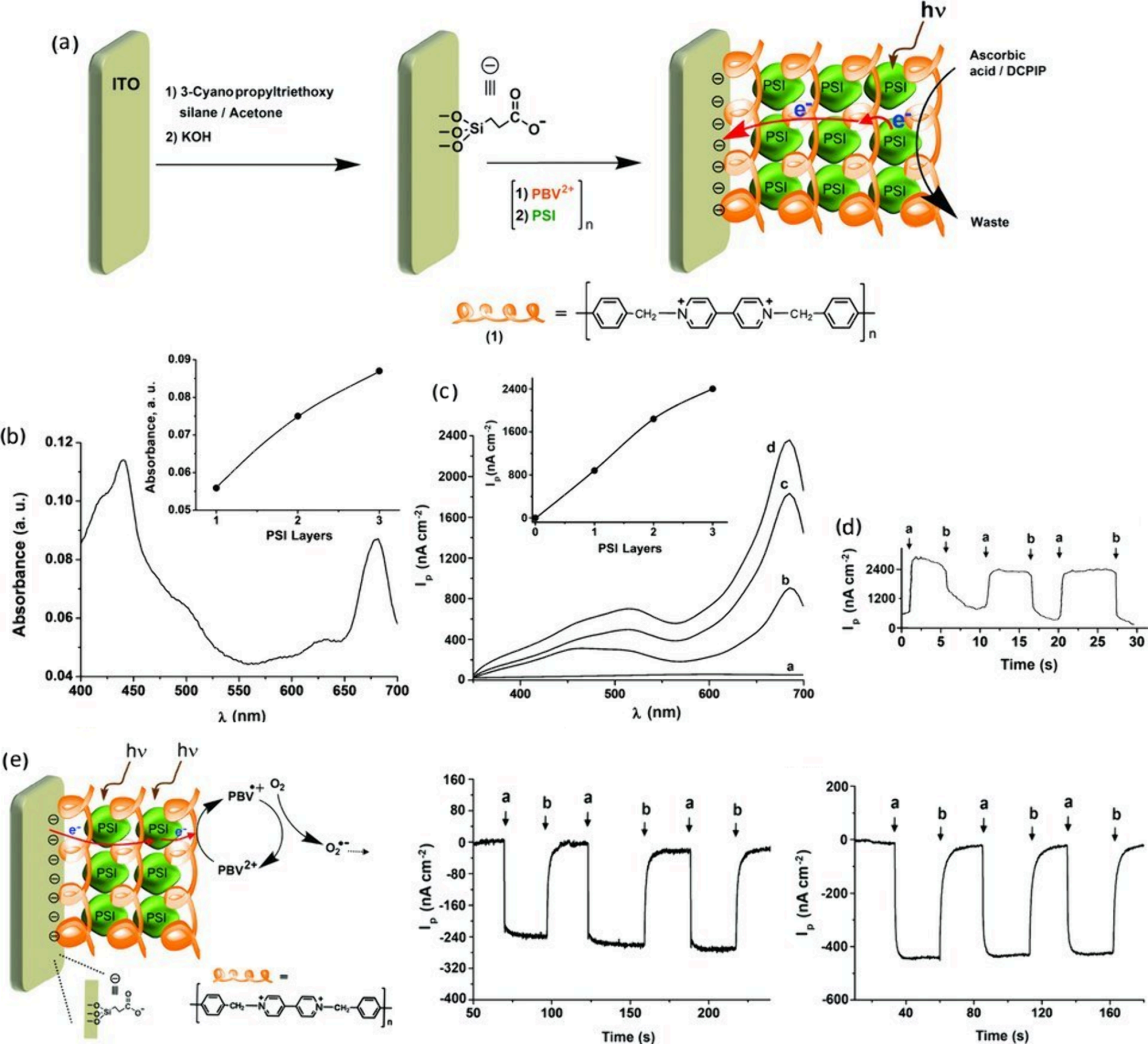
(a) Schematic assembly of the layered PBV^2+^/PSI photoactive composite on an ITO electrode. (b) Absorption spectrum corresponding to an ITO surface modified with 3 layers of the PBV^2+^/PSI composite. The inset shows the dependence of the absorption intensity, at λ = 680 nm, on the number of layers deposited. (c) Photocurrent action spectra corresponding to the layer-by-layer deposited assemblies of the PBV^2+^/PSI composites on the ITO electrode. The curves correspond to (a) Bare ITO; (b) 1; (c) 2, and (d) 3 PBV^2+^/PSI composite layers. The inset shows the dependence of the photocurrent, at λ = 680 nm, on the number of layers deposited on the electrode. (d) Photocurrent responses, at λ = 680 nm, upon the cyclic switchable illumination of the PBV^2+^/PSI-modified electrode, consisting of 3 composite layers. Switch “ON” (illumination on) - marked (a); Switch “OFF” - marked (b). Potential applied *E* = −0.05 V vs Ag/AgCl. The effective illumination area was 0.25 cm^2^. All measurements were performed in an Ar-dehydrated phosphate buffer (0.1 M, pH = 7.2) that contained ascorbic acid, 40 mM, and DCPIP, 50 μM. (e) Cathodic photocurrents generated by PSI/PBV^2+^ based photocathode in the presence of O_2_ and applied potential of 0 V vs Ag/AgCl. Adapted with permission from ref 44. Copyright 2013 Wiley-VCH GmbH.

**Figure 7:**
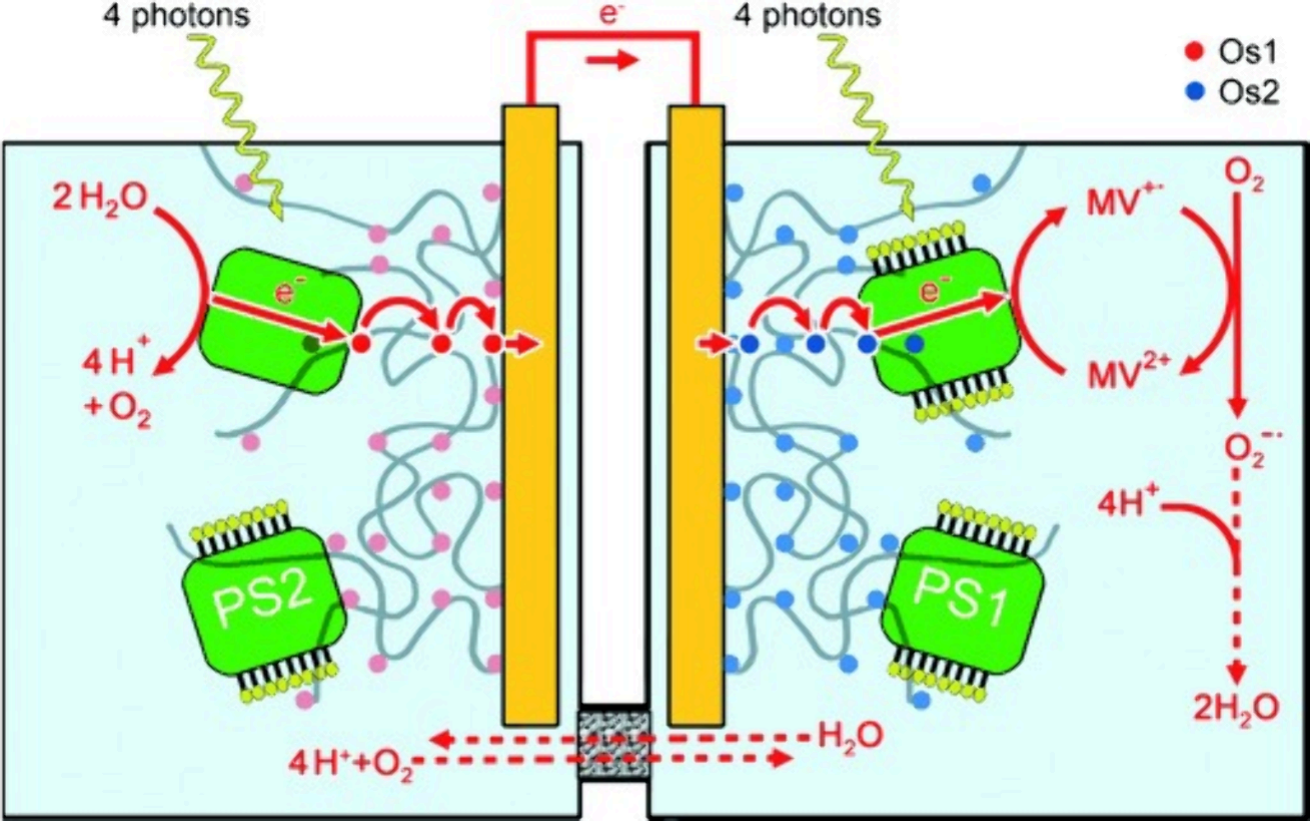
Representation of the proposed biophotovoltaic cell combining a PSII-based photoanode and a PSI-based photocathode. Upon photon absorption, water molecules split into electrons and protons. The electrons are transferred to the cathodic half-cell via the outer circuit, where PSI generates a reductive force of −580 mV vs SHE and reduces the electron acceptor (methyl viologen). The methyl viologen radical cation is reoxidized by molecular oxygen, resulting in water as the final product. Reproduced with permission from ref 56. Copyright 2013 Wiley-VCH GmbH.

Utilizing redox-active polymers to enable a short electron-transfer process is of great importance; however, most of these configurations can lead to nondirected electron flow, resulting in short circuits and a limited efficiency. To tackle these issues, the P700 or Fb site should be oriented toward or away from the electrode surface to generate cathodic or anodic photocurrent, respectively. A recent work by Buesen and co-workers modeled the parameters and electron transfer in biophotoelectrochemical processes. This work presents guidelines to minimize short circuits and back-reaction, which often hinder efficient photocurrent development.^57^ Schuhmann and co-workers addressed this issue by using the Langmuir–Blodgett technique to control the orientation of the PSI on the electrode surface.^58–60^ It is important to note that full coverage of the electrode surface is critical for controlling the direction of electron flow and preventing short circuits. Naturally, cyanobacterial PSI forms a trimer. Therefore, in terms of geometry, casting a densely packed monolayer on the electrode surface is challenging. To tackle this issue, the electrode surfaces were first modified with trimeric PSI, followed by a second deposition of the monomeric form. The resulting PSI-based photoelectrode showed improved photocurrent generation due to greater electrode coverage, which enhanced current and blocked the unmodified electrode surface, which can contribute to undesired, uncontrolled electron-transfer processes.^61^

The electrode and Photosystem I interfacing configurations opened the way toward energy device construction. As mentioned in the previous section, PSI can be used as a potent reducing agent and facilitate hydrogen generation when coupled with an appropriate catalyst. The main disadvantage of such configurations is the use of sacrificial electron donors. Hence, coupling such systems to the electrode surface could potentially solve these issues, as the electrode serves as the electron donor, and Pt cluster/NP^62^ or the hydrogenase^59,63^ enzyme can be used as the hydrogen generation catalysts. It has been found that PSI stability is altered by peroxide species being formed by the photocatalytic process.^64^ To tackle this issue, a quinone electron acceptor was added, presenting enhanced stability.^65^

### 3.2 PSII

#### 3.2.1 Conjugation of Proteins with Inorganic Catalysts and Electrodes

The Photosystem II protein facilitates the fundamental conversion of light energy into chemical energy. In terms of energy and mechanism, water oxidation is a challenging reaction that requires both light absorption and a multielectron-catalytic process.^66^ The protein complex facilitates the light-induced water oxidation reaction by overcoming thermodynamic barriers. The evolution of oxygenic photosynthesis has tremendously changed Earth, enabling the variety that exists today. The importance of oxygen is, of course, crucial to many developed species; however, the evolved oxygen is a byproduct of the generated electrons. Naturally, the internal electron transfer and charge separation end at the Q_b_ site, where the electrons are transferred to PQ, a quinone that serves as a diffusional electron-transfer mediator across the membrane to reduce B6f. The PSII stability under irradiation is very limited due to back-reactions that lead to deactivation.^67^ Unlike the PSI, PSII does not develop a strong reducing power that can be utilized for essential reactions such as hydrogen generation or CO2 fixation. However, like its natural function, it can be coupled with biotic (such as phycobilisomes),^68^ inorganic photosensitizers or nanoelements to achieve the essential higher reducing power, or alternatively, an electron sink, respectively.^69^ Feng and co-workers presented an interesting configuration to facilitate the production of ATP by an artificial system.^70^ In this work, PSII and ATPase were isolated and purified. Core–shell microspheres were designed to encapsulate the PSII complex, which includes the light-harvesting complex and the OEC, Figure 8. To achieve active and stable PSII complexes, it has been cross-linked using glutaraldehyde and BSA as a scaffold to form a PSII-BSA-CaCO_3_. The CaCO_3_ was then removed, leaving a porous structure. The PSII modified protoliposomes were then modified with the ATPase. The ATPase positioned at the membrane, which by light irradiation in the presence of DCPIP as electron scavenger enables the production of ATP. The work demonstrates an artificial biotic abiotic hybrid consisting of PSII as water oxidation catalyst, which in turn, like in the natural system, produces proton gradient which activates the ATPase. The artificial scaffold allows flexible design to reach direct route to biological energy currency, ATP.

**Figure 8:**
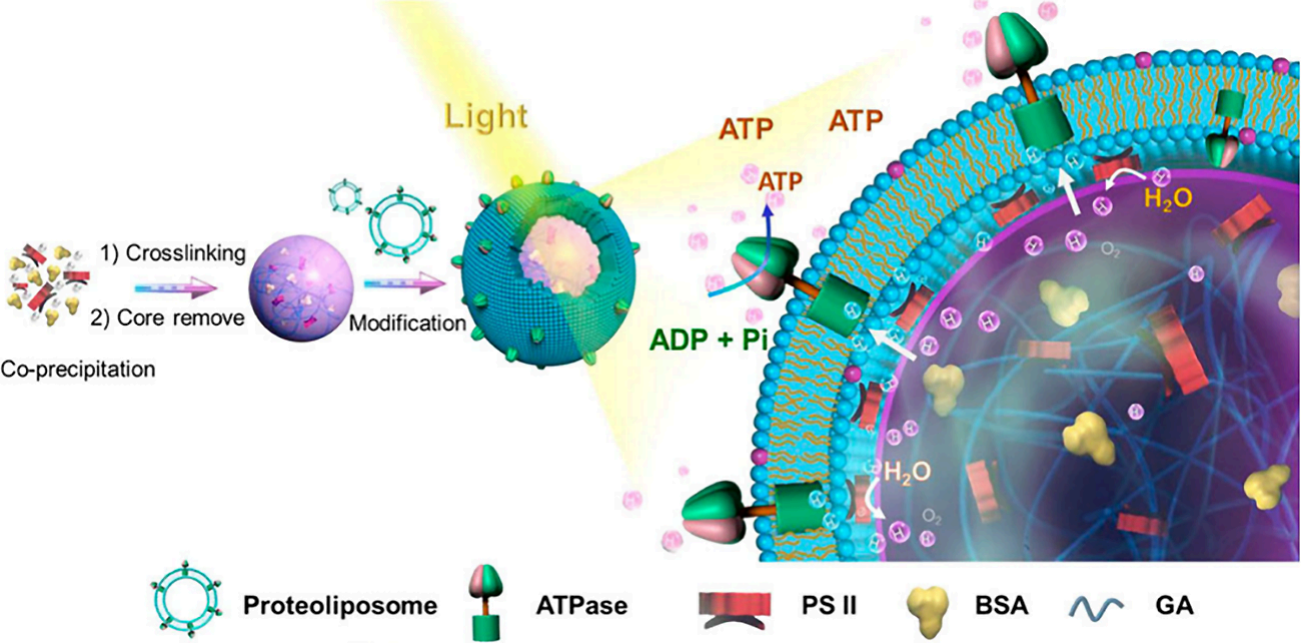
Schematic representation of core–shell microspheres designed for light-driven ATP Synthesis. Adapted from ref^70^. Copyright 2016 American Chemical Society.

This concept was further extended by expanding the light-harvesting range using both the reaction center (RC) and PSII to produce ATP independently and in parallel.^71^ The critical role of the photosynthetic apparatus is to utilize the generated energy currency (ATP and NADPH) and enable the CO_2_ assimilation process. Miller and co-workers presented an advanced approach in which the photoinduced electrons generated by the thylakoids were used to activate an enzymatic cascade that enabled CO_2_ capturing within water-in-oil droplets. The artificial enzymatic cascade was designed to replace the natural CO_2_ assimilation process.^72,73^ Toward this end, 16 enzymes of the crotonyl-CoA/ethylmalonyl-CoA/hydroxybutyryl-CoA (CETCH) cycle were incorporated in the droplets, mimicking both the light and dark cycles, aiming to artificially replicate the chloroplast’s role, as shown in Figure 9. As presented, NADPH could be photogenerated by the thylakoid membranes when a ferredoxin redox mediator was added, as shown in Figure 9a. Also, ATP was continuously produced, with light irradiation greatly enhancing its production, as shown in Figure 9b. As presented, the generated energy currencies, NADPH and ATP, were further coupled to enzymatic reactions for CO_2_ assimilation, Figures 9c and 9d. Stability issues still hinder the adoption of more-applicable approaches. However, a clear route toward artificial photosynthesis has been established. The utilization of isolated PSII in photobiochemical configurations has been quite limited. However, its conjugation with electrodes for water oxidation and electrical energy generation has great promise and has, therefore, been a focus of artificial photosynthesis research in recent decades.

**Figure 9:**
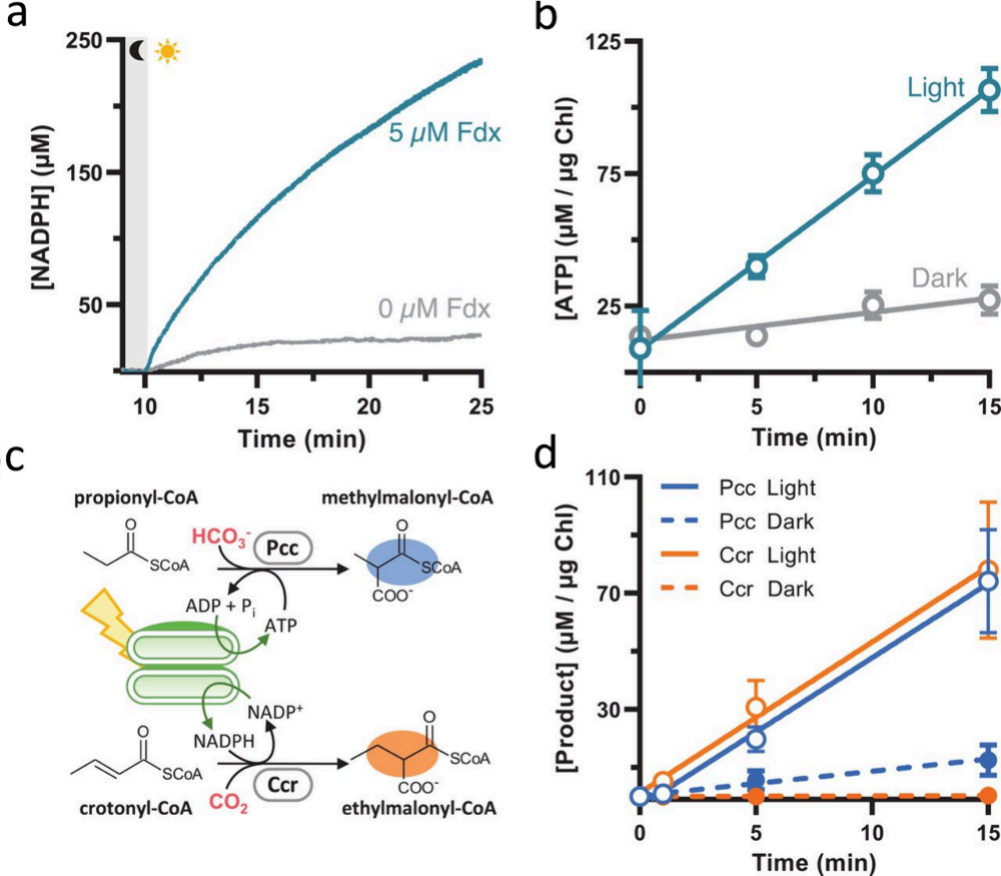
(a) NADPH production is dependent on light and externally added ferredoxin. (b) ATP production is dependent on light, with some background reaction in the dark caused by membrane-bound adenylate kinase. (c) Scheme of TEM-driven carboxylation reactions of Pcc and Ccr using light-produced ATP and NADPH, respectively. (d) Reactions coupled to TEM (2.5 or 5 μg Chl and 60 μmol photons m^–2^ s^–1^) (*N* = 6). Adapted with permission from ref 72. Copyright 2020 AAAS.

#### 3.2.2 PSII Electrochemical Activation

To construct PSII-based photobioelectrochemical devices, the proteins must be oriented to facilitate an efficient electron transfer to the electrode. Unlike the PSI, the protein can function only at the anode to generate photobioelectrocatalytic currents.^74^ By light irradiation, the OEC enables water oxidation, which generates electrons that can be transferred to electrodes. To enhance the generated photocurrents, the Q_b_ protein site should be oriented toward the electrode surface to facilitate efficient electron transfer. Alternatively, using redox mediators or redox-active polymers shortens electron-transfer pathways between the protein and the redox-active moieties, enabling an enhanced protein capacity on electrode surfaces with efficient electron transfer. PSII stability is compromised by D1 subunit degradation.^75^ In nature, D1 can be routinely replaced. However, this cannot be performed in an artificial photosynthetic configuration using an isolated PSII protein. To minimize protein degradation and, in turn, deactivation, back-reactions should be minimized. This could be achieved through tailored charge separation processes, which may be achieved through appropriate orientation and the use of redox-active charge mediators.

Miyachi and co-workers presented an interesting approach for the assembly of a PSII-nanoparticle biohybrid. The biohybrids were designed to enable improved orientation and short-range electron transfer between the PSII protein and the Pt nanoparticles, Figure 10a. As described, an analog to the natural PQ9 was designed. The analog consists of the PQ9 quinone head with a thiolate tail, replacing the natural hydrocarbon tail. The thiolate moiety enabled efficient integration with the novel metal nanoparticle, facilitating a short electron-transfer process. The hybrids were then deposited on electrode surfaces to enable photocurrent generation using a sacrificial electron donor.^76^ Alternatively, His-tag,^74,77^ and reduced graphene oxide^78^ were used to dictate a proper orientation of the PSII toward the electrode for efficient photocurrent generation. Self-assembly monolayers have been used to improve the orientation of PSII on the electrode surface and further stabilize it. Through electrostatic interactions, the PSII Q_b_ site was oriented toward the negatively charged ITO layer, enabling improved photocurrent generation.^79^ Alternatively, the use of a bonded redox-active mediator was examined. By integrating a quinone into the mesoporous ITO electrodes, an efficient electron transfer process was achieved.^80^ The integration of enzymes with osmium (Os) based complexes was realized three decades ago, initially utilized for the construction of biosensing devices. It has been shown that replacing the Os complex ligands can tune the complex redox potential to the system’s requirements. Due to its high stability, pH independence, and efficient electron transfer, it can be used for a wide range of bioelectrochemical applications. Sokol and co-workers integrated Os-complex modified polymer and a phenothiazine-modified polymer with PSII on hierarchically structured inverse opal indium thin oxide (IO-ITO) electrodes. As shown in Figure 10b, the redox-active polymer chains can act as an “electron sink,” enabling continuous photocurrent generation.^81^ As mentioned before, quinones have a similar structure and hydrophobicity to interact with the Q_b_ site pocket. Therefore, quinone, as a redox mediator, may dictate proximity, leading to high turnover rates. Indeed, quinone-based polymers were synthesized to enable high photocurrent generation^82^ or a full H_2_O/O_2_ cycle power-generating device.^83^

**Figure 10:**
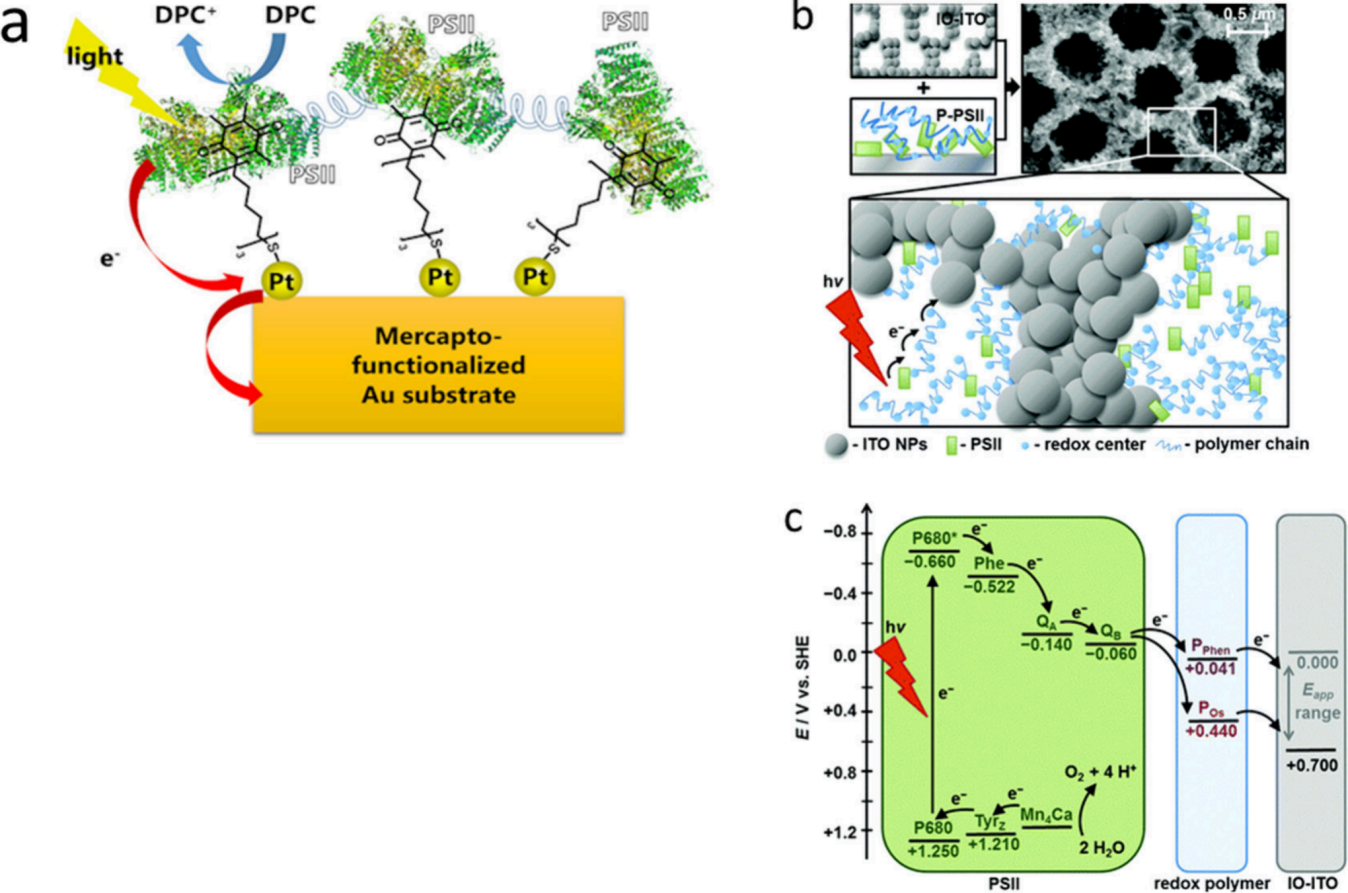
(a) Schematic representation of Photosystem II (PSII)-modified gold electrodes, prepared by the deposition of PSII reconstituted with platinum nanoparticles (PtNPs) on Au electrodes. Adapted from ref 76. Copyright 2017 American Chemical Society. (b) Schematic representation of PSII wired via a redox polymer network to the IO-ITO electrode. (c) Energy level diagram showing electron transfer pathways between PSII and the redox polymer. Adapted from ref 81. Available under a CC-BY 3.0 license. Copyright 2016 The Author(s).

As in the natural process, the electrons generated by the water-oxidation process can be utilized to generate chemical energy, such as hydrogen or formic acid, by reducing H^+^ ^84^ or CO_2_.^85^ As in the natural photosynthesis process, PSII cannot generate enough reducing power to enable the above reactions. Thus, by utilizing Z-scheme configurations, the potential energy required for water oxidation and proton reduction, or for CO_2_ reduction, can be achieved. Alternatively, the system can be biased to compensate for the missing potential.^84^ It should be noted that increased surface area or the use of redox active polymers allowing high electronically wired photosynthetic proteins are essential to reach real-world application photocurrents. Indeed, a significant effort is directed toward this end.^38,44,86–93^

Riedel and co-workers have introduced an interesting approach to developing a Z-scheme biotic-abiotic configuration.^94^ In the designed configuration, PSII was coupled with QDs. An Os-based redox-active polymer was synthesized and used as a buffering layer and redox mediator between the PSII proteins and PbS QDs. The PSII photoexcited electrons were shuttled to the osmium redox-active moieties, which, in turn, acted as electron donors to the photoexcited QDs, Figure 11a. The osmium layer plays two important roles: (i) acting as an electron donor to prevent recombination at the QDs and (ii) acting as an electron acceptor to facilitate the transfer of electrons from the QDs to the TiO_2_ to allow continuous electron flow from the PSII Q_b_ site to the oxidized Os polymer, which minimizes back-reactions and short circuits. The gained potential was further utilized for the activation of bilirubin oxidase, BOD, which enabled oxygen reduction in the cathode chamber, Figure 11b. As shown, the osmium-based redox-active polymer improves charge separation and enables an electron-accumulating layer that closes the photogenerated holes produced by excited QDs. Interestingly, this approach can be tuned to any desired absorption wavelength by controlling the NPs’ dimensions, which are governed by quantum size effects.

**Figure 11:**
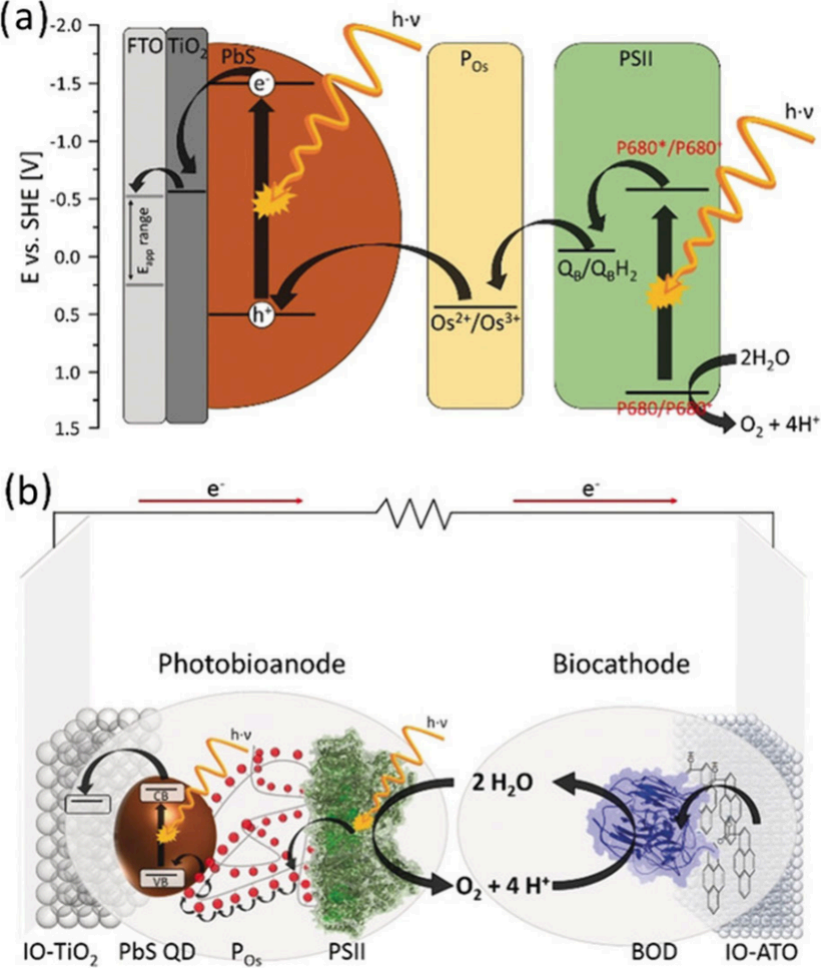
a) Schematic of the electron transfer steps and energetic level of the components of the light-driven signal chain composed of TiO_2_, PbS QDs, redox polymer (P_Os_), and PSII. b) Scheme of the photobioelectrochemical cell consisting of an IO-TiO_2_|PbS|P_Os_|PSII anode and an IO-ATO|PC|BOD cathode. Reproduced with permission from ref 94. Copyright 2018 Wiley-VCH GmbH.

As presented in this section, diverse photosynthetic protein-based devices have been developed. We summarize the main performance characteristics to enable easy comparison between the systems in Table 1.

**Table 1:** Photosynthetic Protein-Based Devices

Photosystem unit	Photoelectrochemical/Photochemical system	Diffusional electron donor/electron acceptor	The catalytic reaction	Maximal photocurrent/photocurrent density	Stability	The applied potential	Maximum activity (in terms of product generation)	Efficiency parameters (maximal value)	Ref.
PSI purified from spinach	Purified PSI reaction centers, platinized at the reducing end and plastocyanin.	Sodium ascorbate	HER	N.A.^a^	H_2_ evolution sustained during 2 h illumination cycles.	N.A.	3.93 μmol H_2_/h/mg Chl	N.A.	Millsaps et al. (ref 25)
PSI from Synechococcus sp. PCC 7002	PSI was covalently linked to Pt and Au NP surfaces by 1,6-hexanedithiol.	DCPIP, sodium ascorbate, Cyt C6	HER	N.A.	N.A.	N.A.	Hydrogen production rate of 49.3 μmol H_2_ mg Chl^–1^ h^–1^ was reported with Pt NPs, rebuilt PS I, 1.6-hexanedithiol, Cyt *c*_6_ system	N.A.	Grimme at al. (ref 26)
PSI from *Synechocystis* sp. PCC 6803	PSI with site-specific attachment of a naphthoquinone molecular-wire–([NQ[CH_2_)_15_S)_2_]) -Pt nanoparticle at A_1_A/A_1_B sites.	DPIP, sodium ascorbate, Cyt C6	HER	N.A.	The rate of hydrogen evolution declined with a half-time of 9 h.	N.A.	The rate of hydrogen generation reached a maximum of 67.6 ± 8.2 μmol of H_2_ (mg of Chl)^−1^ h^–1^ after 1 h.	N.A.	Gorka et al. (ref 27)
PSI from Synechococcus lividus, Synechococcus leopoliensis	PSI with electrostatically associated Pt nanoparticles (PSI/Pt NP).	Ascorbate, cyt c_6_	HER	N.A.	Hydrogen generation stopped after 4 h due to depletion of the sacrificial electron donor.	N.A.	244 μmol H_2_ (mg chlorophyll)^−1^ h^–1^ or 21 034 mol H_2_ (mole PSI)^−1^ h^–1^	N.A.	Utschig et al. (ref 28)
PSI isolated from the thermophilic cyanobacterium *T. Elongatus*	*PSI assembled into PSI-Pt ’photosynthetic NPs’*	Ascorbate, cyt c_6._	HER	N.A.	>85 days (hydrogen yield maintained, intermittent testing)	N.A.	5.5 μmol H_2_ h^–1^ mg^–1^ chlorophyll	N.A.	Iwuchukwu et al. (ref 30)
PSI isolated from *Synechococcus lividus* PCC 6717,	PSI bound to Pt NPs (PSI-Pt NPs biohybrids)	Ascorbate, cyt c_6_	HER	N.A.	The rate of hydrogen evolution was measured for 400 min.	N.A.	6070 mol H_2_ mol PSI^–1^ h^–1^ and a turnover number of 36,600 in 400 min	N.A.	Gisriel et al. (ref 31)
PSI purified from *Thermosynechococcus vulcanus*	PSI/PtNP + artificial light-harvesting dye (Lumogen Red, LR) composite. The combination of PSI with PtNP and LR enabled hydrogen production in the presence of visible light.	ascorbic acid	HER	N.A.	Hydrogen generation was measured for 12 h.	N.A.	26.3 mol H_2_ (mol PSI)^−1^ h^–1^	N.A.	Nagakawa et al. (ref 32)
PSI from Synechocystissp. PCC 6803	Cysteine mutated- PSI covalently bound to gold electrode with Pt deposited on top of each SPI	N.A.	Photovoltage generation	0.12 mA/cm^–2^	N.A.	N.A.	A photovoltage of 0.386 ± 1.4 was generated by the trilayer configuration	N.A.	Frolov et al. (ref 34)
PSI from spinach	BiVO_4_/Au//PSI/Pt or BiVO_4_/Ag//PSI/Pt. H_2_O was the electron donor. The Pt-PSI and the mt-BiVO_4_ was covalently bound.	N.A.	HER	N.A.	Hydrogen evolution was measured for 72 h.	N.A.	Hydrogen evolution of 34 and 15 nmol h^–1^ using Au and Ag as mediators, respectively. 0.42 ± 0.20 and 0.091 ± 0.044 μmol h^–1^ mg Chl^–1^ of H_2_ were produced in the Au- and Ag-mediated systems.	External quantum efficiency of 10^–5^	Kim et al. (ref 35)
RC isolated from *Rhodobacter sphaeroides*	Self-assembled photoelectrochemical complex, comprised of an assembly of reaction centers, membrane scaffold proteins, and phospholipids onto SWCNTs.	Ferrocyanide, ubiquinone	^C^	20 nA	Stability was measured for 168 h, every 32 h a regeneration cycle was operated for a duration of 8 h.	OC potential	N.A.	Photoconversion efficiency >300% over 168 h	Ham et al. (ref 40)
RC from *Rb. sphaeroides*	Monolayers of RC immobilized on gold electrodes.	2,3- dimethoxy-5-methyl-6-geranyl-1,4-benzoquinone, Cyt c	^C^	150 nA	Photocurrent was measured for 80 s	–0.1 V vs Ag/AgCl	Electron transfer rate of 1.16 × 10^2^ (tilting RC orientation, with cytochrome)	N.A.	Lebedev et al. (ref 41)
RCs from *Rhodobacter sphaeroides*	RCs bound on a Ni NTA terminated self-assembled monolayers on gold surface. The RC were bound with the acceptor side looking to the solution through a poly histidine tag (His_7_) constructed at the C-terminal end of the RC M-subunit.	ubiquinone-10, atrazine	^C^	30 nA	N.A.	OC potential	N.A.	N.A.	Trammell et al. (ref 42)
Light-harvesting antenna core (LH1–RC) complexes isolated from *Rhodoseudomonaspalustris*	The LH1–RC complexes self-assembled on an alkanethiol-modified gold electrode	Methyl viologen	^c^	Maximal photocurrent density of 5.1 ± 0.5 × 10^7^ (A cm-^2^ Abs^–1^)	N.A.	–0.2 V vs Ag/AgCl	N.A.	Maximal quantum yield of 8.7 ± 0.8 (×10^3^%)	Kondo et al. (ref 43)
PSI and PSII from thermophilic cyanabacterium *Mastigocladus Laminsus*	Layered assemblies of PSI, or PSII, on ITO electrodes with PBV^2+^ used as an interprotein “glue” and electron mediator. PBV^2+^/PSI/PBQ/PSII photoactive composite on an ITO electrode	Poly benzyl-viologen (PVB^2+^), poly lysine benzoquinone (PBQ), DCPIP, ascorbate	OER, ORR	2.2 μA cm^–2^ for the PBV^2+^/PSI/PBV^2+^/PSI layered assembly	N.A.	–0.05 V vs Ag/AgCl	N.A.	Quantum yield 4%	Yehezkeli et al. (ref 44)
PSI from Synechocystis sp. PCC 6803	Cysteine mutated- PSI covalently bound to gold electrode	N.A.	Light-induced charge separation	N.A.	N.A.	N.A.	A photovoltage of 0.45 V	N.A.	Carmeli et al. (ref 45)
PSI from spinach	PSI monolayers assemble on a gold electrode. Several covalent attachment strategies were examined.	2,6-dichloroindophenol (DCIP), sodium ascorbate	c	100 nA/cm^2^	Stable photocurrent over several hrs for dense monolayers from vacuum-assisted assembly.	–0.11 V vs Ag/AgCl	N.A.	N.A.	Faulkner et al. (ref 46)
PSI from spinach	PSI immobilized on the surface of nanoporous gold leaf (NPGL) electrodes	DCPIP, sodium ascorbate	^C^	∼800 nA/cm^2^	N.A.	–0.1 V vs Ag/AgCl	N.A.	N.A.	Ciesielski et al. (ref 47)
PSI from spinach	Multilayer assemblies od PSI on gold and glass substrates	Ferricyanide, ferrocyanide	^C^	12 μA/cm^2^ ^b^	N.A.	∼300 mV vs Ag/AgCl	N.A.	Internal quantum efficiency (films fabricated by three deposition steps) of 0.014% and 0.017% at of 437 and 678 nm, respectively	Ciesielski et al. (ref 48)
PSI from *Mastigocladus laminosus*	Bis-aniline-cross-linked Pt NPs/PSI composites on Au electrodes and Bis-aniline-Cross-Linked Ferredoxin/Pt NP–Pt nanocluster/PSI composite on a Au electrode.	Ascorbic acid, DCPIP	c	1000 nA	N.A.	0.3 V vs Ag/AgCl	N.A.	Quantum yields of ∼ 2.6% and ∼ 3.8% and IPCE values of ∼ 0.35% and ∼ 0.5% (λ = 420 nm) for the configuration without and with the Fd unit, respectively.	Yehezkeli et al. (ref 49)
PSI from *Thermosynechococcus elongatus*	PSI immobilized on a gold electrode surface via an Os complex containing redox polymer hydrogel	Methyl viologen	ORR	29 μA cm^–2^	N.A.	0 V vs Ag/AgCl	N.A.	IPCE of 3.1%	Badura et al. (ref 50)
PSI from *Thermosynechococcus elongatus*	PSI natural VK1 quinone replaced with a VK1 quinone analog, with viologen end, adsorbed on a gold electrode	Viologen, DCIP, ascorbate	c	40 nA cm^–2^	N.A.	0 V vs Ag/AgCl	N.A.	N.A.	Terasaki Et al. (ref 51)
PSI from *Thermosynechococcus elongatus*	PSI with fused ZnO-binding peptide tag bound to ZnO nanowire	Co(II)/Co(III) ion-containing electrolyte Z813, methyl viologen	ORR	362 μA/cm^2^	N.A.	0 V vs Ag/AgCl	N.A.	Incident-light to electrical external power conversion efficiency of 0.08% with UV, 0.07% without	Mershin et al. (ref 52)
	PSI with designer peptide A_6_K air-dried onto nanocrystalline TiO_2_ paste on FTO glass								
PSI from Thermosynechoccus elongatus	PSI self- assembled onto hydrocarbon monolayers with different head groups deposited on gold electrode	Os(bpy)_2_Cl_2_ and methyl viologen	C	75 nA	>3 h of intermittent illumination.	–0.1 V vs Ag/AgCl	N.A.	N.A.	Manocchi et al. (ref 53)
PSI from *Thermo synechococcus elongatus*	PSI containing redox-hydrogel film of a pH-dependent a poly(vinyl)imidazole Os(bispyridine)_2_Cl	Methyl viologen	ORR	322 ± 19 μA cm^–2^	Depends on the irradiation intensity, half-life ranging from <30 min for the strongest irradiation up to 15 h at 1 mW cm^–2^	0 mV vs Ag/AgCl	Electron transfer rate of 335 ± 14 e^–^ s^–1^ PS1^–1^	N.A.	Kothe et al. (ref 54)
PSI *from Mastigocladus laminosus*	Unbiased PEC of Polymethylene blue/PSI/polybutyl-viologen//BiVO_4_/CoP	Polymethylene blue, polybutyl-viologen	OER, ORR	∼200 μA/cm^2^	a photocurrent drop of less than 20% during 5 h of biocathode measurement.	–0.2 V vs Ag/AgCl	∼0.1 mM H_2_O_2_ generated during 2 h	IPCE of 0.12% for	Herzallh et al. (ref 55)
					Stable OCV for more than 15 h.				
PSI and PSII from *Thermosynechococcus elongatus*	Biophotovoltaic cell comprised of PSII immobilized on the electrode via an imidazole-coordinated bispyridyl osmium complex-based redox hydrogel and PSI immobilized on the electrode via a pyridine-coordinated bispyridyl osmium complex-based redox hydrogel.	Methyl viologen	OER, ORR	1.5 μA cm^–2^ (for the two-compartment cell)	N.A.	unbiased	N.A.	conversion efficiency of 3.6 × 10^–7^	Kothe et al. (ref 56)
PSI from *Thermosynechococcus elongatus*	Biomimetic folded PSI monolayers on an Au electrode modified with P–Os redox polymer (poly(1-vinylimidazole-*co*-allylamine)-[Os(bpy)_2_Cl]Cl). The Langmuir–Blodgett (LB) technique was used to control PSI orientation on the electrode. Several illumination configurations were examined.	Methyl viologen	ORR	∼3 μA/cm^2^	N.A.	0 mV vs Ag/AgCl	N.A.	External quantum efficiency of 0.012 ± 0.003)% for the tilted configuration	Wang et al. (ref 58)
PSI from *Thermosynechococcus elongatus* and PSII	Au/P-Os(I) redox polymer/PSI/viologen modified polymer/Hydrogenase biocathode combined with a P-Os (II) redox polymer/PSII-based bioanode demonstrating a fully light-driven Z-scheme mimic biophotovoltaic cell for bias-free water splitting.	Methyl viologen, viologen modified polymer (p-vio)	OER, HER	∼−17 μA cm^–2^ for Au/P-Os(I)/ PSI-LB with p-vio layer (while subjected to 210 mV vs SHE)	N.A.	The anode and the cathode- 0 mV vs Ag/AgCl	electron throughput of 183 ± 25 e^–^ PSI^–1^ s^–1^ for (Au/P-Os(I)/PSI-LB/P-vio assembly under ambient air)	N.A.	Zhao Et al. (ref 59)
						The full cell is unbiased			
PSI from *Thermosynechococcus elongatus*	Au/P-vio/PSI-LB/PEGDGE/P-Os/GOx biophootoanode coupled with carbon electrode modified with 1-pyrenebutyric acid *N*-hydroxysuccinimide ester (PBSE), GOx and HRP biocathode	Glucose	Glucose oxidation, H_2_O_2_ reduction	The biophotoanode generated 17.6 μA cm^–2^ at 200 mV vs SHE	The biophotoanode stability was tested for 90 min.	The full cell is unbiased	N.A.	N.A.	Wang et al. (ref 60)
PSI from *Thermosynechococcus elongatus*	Au/Os-complex-modified redox polymer deposited with mixed PSI monolayers of PSI trimers and monomers. A top of H_2_ase/BPEI-[CoCp_2_] or H_2_ase/viologen modified polymer (P-vio) were examined	Methyl viologen	HER	–(9 ± 1) μA cm^–2^ for the Au/P-Os/mixed PSI monolayers.	N.A.	0 mV vs Ag/AgCl	N.A.	N.A.	Wang et al. (ref 61)
PSI from *Thermosynechococcus elongatus*	Au electrodes modified with PSI-Pt NP complexes integrated in a poly(vinyl)imidazole Os(bispyridine)_2_Cl redox hydrogel.	Methyl viologen	HER	4.8 ± 0.4 μA cm^–2^, under anaerobic conditions	N.A.	0 mV vs Ag/AgCl	N.A.	N.A.	Zhao et al. (ref 62)
PSI from *Synechocystis sp. PCC 6803*	PSI-H_2_ase fusions embedded eithin Poly(1-vinylimidazole-coallylamine)-[Os(2,2’-bipyridine)2Cl]Cl redox polymer immobilized on Au electrode	Methyl viologen	HER	∼700 nA/cm^2^	Hydrogen production *in vivo* was tested for 25 min.	0 mV vs Ag/AgCl	0.01–0.02 μmol H_2_/min/mg Chl (*in vivo*)	N.A.	Wang et al. (ref 63)
					Photocurrent response was measured for a few min.				
PSI from *Thermosynechococcus elongatus*	PSI/Os-complex-modified redox polymer (Os-P) on Au electrode. Catalase and superoxide dismutase were included.	Cu-tris(2-pyridylmethyl)amine nitrate (CuTPA), methyl viologen	ORR	∼375 nA while in O_2_-saturated solution	87% of the initial photocurrent was maintained while in air equilibrated solution	0 mV vs Ag/AgCl/	N.A.	N.A.	Zhao et al. (ref 64)
PSI from *Thermosynechococcus elongatus*	PSI/Os-complex-modified redox polymer (Os-P) on Au electrode.	Methyl viologen, 2,3-dimethoxy-5-methyl-1,4-benzoquinone (Q_0_)	c	∼150 μA cm^–2^	Tested for 930 min, improved stability with Q_0_ as electron acceptor under anaerobic conditions.	0 mV or 100 mV vs Ag/AgCl for MV^2+^ or Q_0_, respectively	N.A.	N.A.	Zhao et al. (ref 65)
PSII from *Thermosynechococcus elongatus*, Phycobilisomes (PBSs) from *Acaryochloris marina*, *Mastigocladus laminosus*, and *Synechocystis* sp. PCC 6803	PBS–PSII supercomplexes integrated within an Os-complex-modified hydrogel (P-Os) on macro-porous indium tin oxide electrodes	N.A.	OER	>10 μA cm^–2^ for all PSII-containing modifications	wavelength-dependent photocurrent response was measured during 700 s	0.4 V vs Ag/AgCl	C	IPCE 10.9% at 670 nm	Hartmann et al. (ref 68)
PSII from *Thermosynechococcus elongatus*	PSII-gold NPs (GNP) conjugates	Phenyl-*p*-benzoquinone (P*p*BQ), ferricyanide	OER	N.A.	N.A.	N.A.	575 ± 80 μmol O_2_ (mg Chl)^−1^ h^–1^	N.A.	Noji et al. (ref 69)
PSII-enriched membrane particles isolated from spinach (*Spinacia oleracea* L.)	F_o_F_1_-ATPase proteoliposome-coated PSII-based microspheres with core@shell structures.	DCPIP, K_3_[Fe(CN)_6_]	OER, ATP synthesis	N.A.	The rate of ATP production becomes much smaller and it reaches a plateau after about 80 min.	N.A.	1100 nmol ATP (mg Chl)^−1^. The rate of ATP production becomes much lower, reaching a plateau after 80 min	N.A.	Feng et al. (ref 70)
					At least 50% functionality of photodriven ATP synthesis was retained after 3 weeks of storage at 4 °C in dark				
Proteorhodopsin from *Gamma proteobacterium* and PSII from *Spinacia oleracea*	An artificial organelle combining two photoconverters -PSII proteorhodopsin and ATP synthase. The organelle is encapsulated in a giant vesicle comprising pyruvate carboxylase, acetylcoenzyme A, and bicarbonate	phenyl-p-benzoquinone	ATP synthesis, carbon fixation (oxaloacetate, C4 formation from pyruvate, C3)	∼−3 fA for the artificial organelle (described as photocurrents/chromophores)	The artificial organelle sustained ATP conversion for 3 days (half-maximum efficiency) at room temperature and 1 month at 4 °C	N.A.	∼300 (OAA chromophores) and ∼ 300 (ATP/chromophores) during 20 min	N.A.	Lee et al. (ref 71)
Thylakoid Membranes from the chloroplasts of *Spinacia oleracea*	Thylakoid membrane-based energy module coupled with two different CO_2_-fixing enzymes, crotonyl-coenzyme A carboxylase/reductase and propionyl-CoA carboxylase. Sixteen enzymes of the crotonyl-CoA/ethylmalonyl-CoA/hydroxybutyryl-CoA (CETCH) cycle and a glyoxylate/hydroxypyruvate reductase from *E. coli* coupled with the thylakoid membrane-based energy module within water-in-oil droplets	Ferredoxin	NADPH and ATP production, CO_2_ fixation	N.A.	The CETCH version 7.0 coupled to TEM operating inside microdroplets demostrated glycolate production during 150 min.	N.A.	The integrated system produced 47 ± 5 mM glycolate from CO_2_ during 90 min	Carbon-conversion efficiency ∼ 3.5% (integrated system, full CETCH cycle)	Miller et al. (ref 72)
PSII from *Thermosynechococcus elongatus*	PSII reconstituted with platinum nanoparticles deposited on gold electrodes	1,5-diphenylcarbazide	c	15 nA cm^–2^	N.A.	0.10 V vs Ag/AgCl	TOF of 2 e^–^ (PSII)^−1^ s^–1^	external quantum yield of 9.2 × 10^–7^	Miyachi et al. (ref 76)
PSII from *Thermosynechococcus vulcanus*	PSII, tagged with six histidine residues, was immobilized on a gold surface modified with a self-assembled monolayer (SAM) of a nickel-nitrilotriacetic acid complex (Ni-NTA).	N.A.	OER	2440 nA cm^–2^	Anodic photocurrent was maintained for at least tens of minutes for PSII immobilized on a planar gold electrode configuration	0.2 V vs Ag/AgCl	N.A.	N.A.	Terasaki et al. (ref 77)
PSII from spinach	ITO functionalized with layer-by-layer assembly of PSII and polyethylenimine-reduced graphene oxide multilayered films	–	OER	37.2 nA cm^–2^	∼65% of the initial photocurrent remained after 20 min illumination of the PEI–rGO/PSII films	0.2 vs Ag/AgCl	N.A.	Quantum efficiency of ∼ 0.0026%	Cai et al. (ref 78)
PSII from *Thermosynechococcus elongatus*	PSII covalently bound to ITO electrode modified with a self-assembled monolayer (SAM) of phosphonic acid ITO linkers with a dangling carboxylate moiety.	2,6-dichloro-1,4-benzoquinone	OER	4.5 μA cm^–2^	half-life time ∼ 12 min for the *C*-*meso*ITO|SAM-C_2_CO_2_^–^|PSII, MET configuration	0.3 V vs Ag/AgCl	TOF of 4.6 ± 0.6 mol O_2_ (mol PSII)^−1^ s^–1^ for the *C*-*meso*ITO|SAM-C_2_CO_2_^–^|PSII, MET configuration	IPCE of 0.1% for the *C*-*meso*ITO|SAM-C_2_CO_2_^–^|PSII, MET configuration	Kato et al. (ref 79)
PSII from *Thermosynechococcus elongatus*	PSII on a mesoporous indium–tin oxide electrode.	Potassium 1,4-naphthoquinone-2-sulfonate, 2,6-dichloro-1,4-benzoquinone	OER	1.6 ± 0.3 μA cm^–2^ and 22 ± 2 μA cm^–2^, for DET configuration and with 2,6-dichloro-1,4-benzoquinone as electron mediator, respectively	A half-life time of 4 to 5 min under continuous red light illumination	0.3 V vs Ag/AgCl	TOF of 0.18 ± 0.04 (mol O_2_) (mol PSII)^−1^ s^–1^ and 3.2 ± 0.4 (mol O_2_) (mol PSII)^−1^ s^–1^, for DET configuration and with 2,6-dichloro-1,4-benzoquinone as electron mediator, respectively	IPCE 0.3% (635 nm, + 0.5 V vs NHE, in presence of 1 mM of NQS)	Kato et al. (ref 80)
PSII from *Thermosynechococcus elongatus*	PSII, poly(1-vinylimidazole)-Os(bipy)_2_Cl-polymer (P_Os_) and phenothiazine-modified polymer integrated on hierarchically structured inverse opal indium tin oxide electrodes	2,6-dichloro-1,4-benzoquinone (DCBQ)	OER	381 ± 31 μA cm^–2^ and 513 ± 29 μA cm^–2^ for IO-ITO|P_Os_–PSII in DET configuration and with DCBQ as electron mediator, respectively	∼7% and 11% of the initial photocurrent was detected after 60 min of irradiation, for the IO-ITO|P_Os_–PSII electrode, in DET configuration and with DCBQ as electron mediator, respectively	0.3 V vs. Ag/AgCl	Maximal TOF of 4.0 ± 0.4 s^–1^ and 6.7 ± 0.7 s^–1^ for IO-ITO|P_Os_–PSII in DET configuration and with DCBQ as electron mediator, respectively	An external quantum efficiency of 6.9 ± 0.9% and 9.3 ± 1.2% for IO-ITO|P_Os_–PSII in DET configuration and with DCBQ as electron mediator, respectively	Sokol et al. (ref 81)
PSII from *Synechococcus bigranulatus*	PSII deposited on poly mercapto-*p*-benzoquinone (polySBQ) covered gold electrodes as a sensor for herbicide detection	Duroquinone	OER	8.17 × 10^–7^	Tested during 10 s of illumination with a 180 s interval between each light phase. The observed signal decreased exponentially with *t* ^1/2^ within ∼ 3 h.	250 mV vs Ag/AgCl	N.A.	N.A.	Maly et al. (ref 82)
PSII from *Mastigocladus laminosus*	poly(mercapto-p-benzoquinone), pMBQ/PSII//BOD/CNTs and bis-aniline-cross-linked Au NPs/PSII//BOD/CNTs PBEC	N.A.	OER, ORR	∼400 nA for pMBQ/PSII while subjected to 0.15 V vs SCE	For pMBQ/PSII/BOD/CNTs cell, stable photocurrent during 3h and then degraded by 15% upon 10 h	0.1 V vs Ag/AgCl and >0.1 V vs Ag/AgCl for pMBQ/PSII and for bis-aniline-cross-linked Au NPs/PSII anodes, respectively.	Electron transfer turnover rate between PSII and the electrode of 518 e^– s–1^, for pMBQ/PSII electrode	QY of 1.0% and 0.7% for pMBQ/PSII/BOD/CNTs and for bis-aniline-cross-linked Au NPs/PSII//BOD/CNTs PBECs, respectively.	Yehezkeli et al. (ref 83)
PSII core particles isolated from a CP47 His-tagged mutant from T. elongatus BP-1	IO-mesoITO|PSII photoanode// IO-mesoITO|H2ase cathode	2,6- dichloro-1,4-benzoquinone	OER, HER	For IO-mesoITO|PSII photoanode: maximal 930 ± 30 μA cm^–2^, 40 μm film thickness at 0.5 V vs NHE.	IO-mesoITO|PSII photoanode decayed to a few percent of its initial value within 1 h while subjected to 0.5 V vs NHE.	For a full cell, potentials >0.6 V are required for photocurrent generation.	After 1 h of full cell illumination and 0.9 V applied potential, 0.52 ± 0.04 (μmol O_2_) cm^–2^, and 0.96 ± 0.08 (μmol H_2)_ cm^–2^ were generated.	Faradaic yield of (104 ± 5)% and (98 ± 2)% for OER and HER, respectively (full cell activation)	Mersch et al. (ref 84)
				For IO-mesoITO|H2ase cathode: ∼ −1.5 mA/cm^2^ at −0.6 V vs NHE	IO-mesoITO|H2ase cathode: After 5 h, >80% of the initial current remained, while subjected to −0.6 V vs NHE.				
				Full cell: 450 ± 10 μA cm^–2^, 0.9 V					
PSII from *Thermosynechococcus elongatus*	PSII-based photoanode combining dpp (a phosphonated diketopyrrolopyrrole dye) and P_Os_ [poly(1-vinylimidazole-*co*-allylamine)-[Os(bipy)_2_Cl]Cl redox polymer] coupled with FDH adsorbed on a hierarchically structured inverse opal titanium dioxide scaffold (IO-TiO_2_|FDH)	N.A.	OER, CO_2_ reduction to formate	For IO-TiO_2_|FDH −240 μA cm^–2^ at −0.6 V vs SHE	For IO-TiO_2_|FDH ∼ 83% of initial activity retained after 2 h at −0.6 vs SHE	The full cell was biased with 0.3 V	0.185 ± 0.017 μmol cm^–2^ formate (full cell)	Faradaic efficiency of (70 ± 6)% for formate generation	Sokol et al. (ref 85)
					For the full cell, the photocurrent decayed from 92 to 7 μA cm^–2^ after 1 h with a half-life time of ∼8 min				
PSII from *Thermosynechococcus elongatus*	PSII-loaded on three-dimensional ITO and graphene electrodes.	2,5-dichloro-1,4-benzoquinone	OER	165.17 ± 24.93 μA cm^–2^ for ITO-40 750 nm electrode, with electron mediator	half-life of protein films inside the 750 nm IO–ITO electrode of ∼ 4 min, while subjected to 0.5 V vs SHE, with electron mediator	0.3 V vs Ag/AgCl	TOF of 7.17 ± 0.30 s^–1^ for ITO-10 3 μm electrode with electron mediator	N.A.	Fang et al. (ref 87)
PSII from spinach and from *Thermosynechococcus elongatus*	PSII adsorbed on hierarchically structured ITO electrodes	2,6-dichlorobenzoquinone C60 (N,N-dimethyl pyrrolidinium) idodide	OER	∼18 μA cm^–2^ for PSII adsorbed on ITO in the presence of C_60_-DMePyl matrix while subjected to 0.7 V vs SCE	N.A.	Stepped chronoamperometry measurements in the potential range of 0.15–0.75 V vs Ag/AgCl	N.A.	N.A.	Zhang et al. (ref 89)
PSI from *Thermosynechococcus elongatus BP-1*	Biohybrid electrodes comprising electrospun (e-spun) 3D ITO, PSI and cytochrome *c*.	N.A.	ORR	–1.5E^–5^ A cm^–2^ for e-spun ITO/PSI/cyt c with 60 min e-spun ITO, while subjected to −0.1 V vs Ag/AgCl	Photocurrent was measured with different light intensities and bias potentials for 25 min.	–0.1/–0.2 V vs Ag/AgCl	Number of electrons processed by each protein complex per second of 6.2 ± 1.5, for electrodes with 10 min spinning time	EQE of 0.14% for electrodes with 60 min spinning time (20 mW/cm^2^)	Nioradze et al. (ref 90)
PSI from *Thermosynechococcus elongatus*	PSI immobilized on 3D ITO electrodes with cyt *c*	Cyt c, 2,3-dimetoxy-5-methyl-parabenzoquinone (Q_0_)	ORR	270 μA cm^–2^ (for 3D-ITO_15×_–PSI–cyt *c* electrode, while subjected to – 0.15 V vs. Ag/AgCl)	A photocurrent of ∼ 100 μA cm^–2^ is retained during 30 min with Q_0_ for 3D-ITO_15×_–PSI–cyt *c* electrode, while subjected to −0.15 V vs. Ag/AgCl	–0.15/–0.2 vs Ag/AgCl	turnover number of 30 e^–^ s^–1^ at PSI	N.A.	Ciornii et al. (ref 91)
PSI from *Thermosynechococcus elongatus*	PSI immobilized on IO-ITO	N.A.	ORR	10.1 μA cm^–2^	90% of the photocurrent output was retained after repeated illuminations for 90 min.	–0.1 V vs Ag/AgCl	TOF of 6.5 e^–^ PSI^–1^ s^–1^	IQE of 4% and 0.8% EQE	Morlock et al. (ref 92)
					After 3 days at room temperature and a total operation time of 3 h, 90% of the photocurrent is retained.				
PSII from T. elongatus	IO-TiO_2_/PbS QDs/P_Os_/PSII anode and an inverse-opal antimony tin oxide (IO-ATO)| pyrenecarboxylic acid (PC)|BOD cathode	N.A.	OER, ORR	160 ± 36 μA cm^–2^ (while subjected to 0 mV vs Ag/AgCl). (anode) −287 ± 18 μA cm^–2^, (cathode, 8 IO-ATO layers) 76 ± 7 μA cm^–2^ (full cell)	Full cell stability tested during 30 min with 0.4 V applied.	The anode tested while subjected to 0 V vs Ag/AgCl.	electron turnover frequency of *k*_et_ = 5.2 ± 1.1 e^–^ s^–1^ (anode)	EQE of 0.36 ± 0.08 for the anode	Riedel et al. (ref 94)
						Maximal cathodic current at 0.3 vs Ag/AgCl			

a N.A. - Data were not listed or are irrelevant for this configuration.

b Estimated from data within the article.

c Photocurrent generation was used as a measure of the photoelectrochemical electron transfer, rather than the catalytic product formation.

## 4 Architectures for Efficient Charge Transfer Processes

The photosynthesis process comprises highly efficient light-absorbing photosensitizers with near-unity efficiencies. However, the excited electrons will undergo back reactions and recombination unless the charges are spatially separated following the initial excitation.^95^ Sophisticated biotic architecture has developed, enabling efficient intra- and interelectron transfer processes. Therefore, developing successful artificial photosynthesis configurations requires the construction of scaffolds or layers that are tuned for an optimal electron-transfer process and charge separation.^96^ Over the last several decades, the fundamental structural and functional understanding of each photosynthetic component has been investigated, shedding light on its importance. The research stemming from the basic research has led to two distinct subdirections. The first is improving the natural process, mainly by rational design and bioengineering methodologies.^97–99^ The second focused on replacing natural components with external unnatural elements that can perform similarly or surpass them.^2,100^ This direction can be further divided into fully abiotic components^101–104^ or biotic-abiotic interfaces.^105–112^ In all of the above, one should consider how to pin each element into its specific spatial position to maximize performance and prevent back reactions, inactivation processes, short circuits, and more.^113^

Previous and current research aimed to mimic the water-oxidation machinery by constructing new inorganic complexes^11,114–119^ and further conjugating them with photosensitizers. Alternatively, semiconductor-based photocatalysts were used to enable efficient, long-term performance in water oxidation reactions.^101,103,120–123^

The natural model dictates precise control over the photosensitizers and the redox-active position and orientation.^124^ Hence, toward photosynthesis biomimicry, different architectures have been developed to enable controllable charge separation and electron donor distances.^125–127^ This section provides a variety of scaffold materials for the design of artificial photosynthesis.

### 4.1 Peptides or DNA Scaffolds for Artificial Photosynthesis

The utilization of DNA for unique architecture and scaffold construction has significantly evolved during the last few decades.^128–130^ The developed platform enables site-specific positioning by using sticky ends. Thus, the DNA scaffold could be used to assemble site-specific photosensitizers and charge-separation acceptors,^131^ nanomaterials,^132,133^ dyes,^134^ or enzymes^135–137^ with controllable distance and position. An interesting approach involves using biological components, such as peptides^138–140^ or DNA strands, as building blocks for self-assembled 2D and 3D structures.^134,141–146^

Amino acids are building blocks for biological machinery. Over billions of years of evolution, nature has evolved proteins and enzymes that enable a magnificent variety of catalytic reactions to exist on our planet. Can we use these simple blocks for the design of minimal photosynthesis processes?

The use of two- or three-amino-acid peptides for self-assembled 2D and 3D structures has been demonstrated for a variety of purposes. Kim and co-workers developed a photosystem-like configuration in which the photosensitizer, tetra­(p-hydroxyphenyl) porphyrin (THPP), was coupled to diphenylalanine to form a wire-like structure, as shown in Figure 12.^147^ The designed system enabled light-induced generation of NADPH by activating [Cp*Rh­(bpy)­H_2_O]^2+^, M, which acted as a redox catalyst. NADPH produced, in turn, was used as a cofactor to facilitate the enzymatic reaction. It should be noted that the system requires the addition of TEOA, which is a sacrificial electron donor for continuous NADPH production.

**Figure 12:**
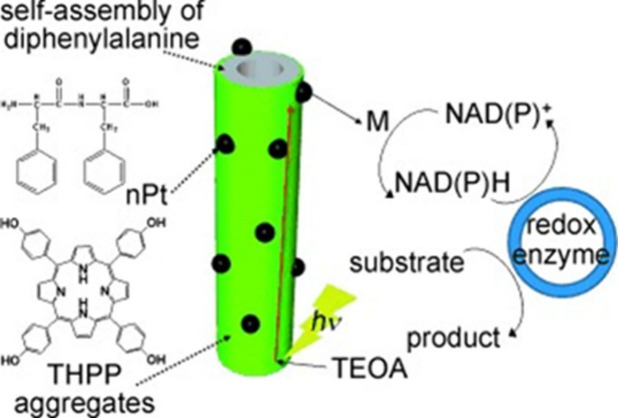
Light-harvesting peptide nanotubes, synthesized by the self-assembly of diphenylalanine with THPP and platinum nanoparticles, for light-induced NADPH generation. [Cp*Rh­(bpy)­H_2_O]^2+^ and triethanolamine, TEOA, were used as electron mediator (M) and electron donor, respectively. Adapted with permission from ref 147. Copyright 2011 Wiley-VCH GmbH.

Ma and co-workers utilized a DNA scaffold to construct a Z-scheme configuration as illustrated in Figure 13.^148^ As presented, the DNA scaffold provided a controlled distance between the two photocenters, the TiO_2_ and the CdS nanorods, enabling water oxidation and H_2_ generation, respectively. The TiO_2_ acts as a “PSII-like” photocatalyst while the CdS, which holds higher reducing energy due to its conductive band edges positions, acts as a “PSI-like”. Pt clusters were grown on the CdS nanorods to improve the proton reduction process. To enable an efficient electron transfer process and improved charge separation, a redox center that can shuttle electrons and protons is required. Therefore, benzoquinone was tailored on the DNA scaffold using a single-stranded DNA tail. The quinone facilitated the electron transfer process between the two photoactive centers and enhanced H_2_ generation.

**Figure 13:**
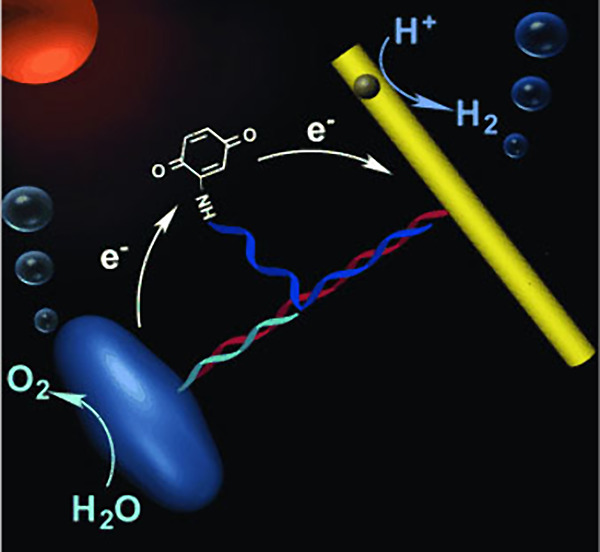
TiO_2_ and CdS nanocrystals were organized into a Z-scheme photosynthesis system by using DNA as a structure-directing agent. Increased H_2_ production from water splitting was observed compared to either the photocatalyst alone or dispersed mixtures of the two. The inclusion of the electron mediator benzoquinone equidistant between the TiO_2_ and CdS through DNA assembly further increased H_2_ production. Reproduced with permission from ref 148. Copyright 2015 Wiley-VCH GmbH.

It has been shown that the optimal configuration enables a complete water-splitting reaction, producing both hydrogen and oxygen. The designed system has several key features that are critical for future artificial apparatuses:

a. The DNA scaffold introduced a controllable spatial separation between the photocatalysts and the redox mediator.
b. The system absorbs a broad wavelength range, with TiO_2_ absorbing at shorter wavelengths and CdS absorbing at longer ones.
c. Most semiconductor-based hydrogen generation systems require sacrificial electron donors to operate continuously. Here, TIO_2_/CdS coupling enables its operation without sacrificial electron donors.

While this model presents an interesting approach, its absorbance is still limited, and shorter bandgap semiconductors, such as CdTe, may be coupled to extend the visible-wavelength absorbance.

### 4.2 Molecular Bridges for Artificial Photosynthesis

The development of new chemistries and methodologies for integrating chemical moieties or molecules in a controlled manner has enabled the construction of new electronic devices. Aiming for artificial photosynthesis processes, several components should be incorporated:

(i) Light-harvesting antenna.
(ii) A photosensitizer.
(iii) Charge separation processes.
(iv) Electron acceptors/catalysts.

Gust and Moore presented the fundamental principles for developing such configurations. These include chemical bond bridges that enable precise control of the distance and orientation of each component.^126^ Indeed, over the last few decades, significant efforts have been made to develop architectures that enable artificial photosynthesis configurations.^149,150^

Li and co-workers presented a host–guest configuration that enables the self-assembly of a photosensitizer with a water-oxidation catalyst, Figure 14. The Ru-based photocatalyst was conjugated to a cyclodextrin, enabling host–guest interactions with the Ru-based water oxidation catalyst. The elegantly designed system reached a quantum efficiency of 84% under photoirradiation at 450 nm with a sacrificial electron acceptor.^151^

**Figure 14:**
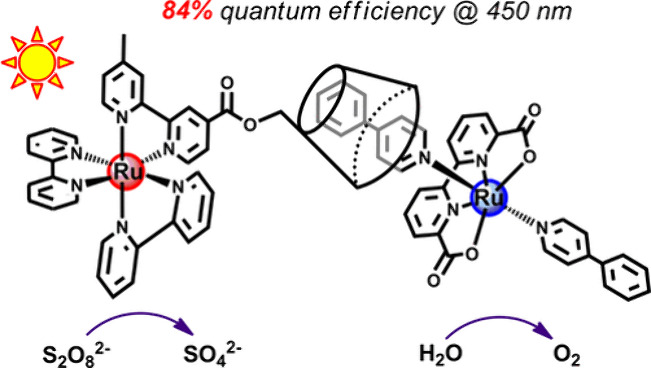
A host–guest configuration for light-driven water oxidation, comprising a cyclodextrin-modified ruthenium as a photosensitizer and phenyl-modified ruthenium complexes as the catalysts. Adapted from ref 151. Copyright 2015 American Chemical Society.

As mentioned, charge separation processes are critical for improving the performance of artificial photosynthesis systems. To that end, molecular wires have been developed to enable charge transport through insulating layers, thereby enhancing activity.^152^ The conjugated molecular wire can be tuned by adding with- or donating-groups to the designed wire. Efficient hole transport through a SiO_2_ membrane was achieved, enabling the activation of inorganic catalysts.^152,153^ The developed methodology opens new possibilities to conjugate catalysts toward artificial photosynthesis, or to enable coupling abiotic elements with biotic ones,^154^ as will be discussed later. The use of metal–organic frameworks for artificial photosynthesis was also examined.^155^ By conjugation of water oxidation catalysts with CO_2_ ^156^ to formate or CO^157^ reduction processes, a standalone artificial photosynthesis configuration can be developed.^155^

Hong and co-workers recently developed an artificial photosynthesis configuration that fully mimics the photosynthetic light cycle.^158^ The designed system comprises a water oxidation catalyst, a charge transfer unit, and an NADP reduction catalyst to NADPH. The two phasic solution configurations utilized light irradiation as the sole energy source for facilitating water oxidation to O_2_ ^159^ and the production of NADPH. While the system presented is not fully bridged, it clearly demonstrates that an inorganic catalyst can provide a stable water-oxidation and NADP-reduction catalyst that can be activated by light irradiation in conjugated processes.

### 4.3 Photosynthesis Mimicry by Catalysts or Electrode Surface Design

Utilizing electrode surfaces for photodriven electrocatalysis, and specifically for photosynthesis mimicry, is of great importance for future energy applications. Unlike homogeneous catalysis, the heterogeneous catalysis process occurs at the electrode surface, allowing operation without an installed membrane or separation barriers between the anodic and the cathodic sides. As mentioned at the beginning of this article, the first demonstration of full water splitting sparked tremendous research into artificial photosynthesis. While photodriven water splitting generates hydrogen fuel, other critical chemical fuels derived from N_2_ or CO_2_ can be very beneficial.^160^ The first demonstration has used a wide-band gap TiO_2_ photoanode, which developed the essential potential to enable both water oxidation and reduction.

Nevertheless, the developed system was limited to UV wavelengths for activation. With advances in material fabrication and design, a photoelectrode could be fabricated with the required properties to mimic the photosynthetic apparatus. Over the last few decades, dye-sensitized solar cells have been developed, enabling electrical power generation while visible light is being used for activation.^161,162^ In the developed cells, redox mediators facilitated the charge transfer between the photoanode and the cathode, enabling electrical power or H_2_ fuel generation.^163^

While wide-bandgap semiconductors can potentially meet the energy requirements to facilitate CO_2_ or proton reduction without biasing or the addition of sacrificial electron donors, they have limited visible-light absorbance. Therefore, designing a Z-scheme configuration with adjustable absorbance wavelength and energy levels surpasses the capabilities of a single photosensitizer.^164^ Toward that, several key features should be considered:

A. The semiconductor layers may be fabricated at one^165^ or two electrodes.
B. Cocatalysts should be considered to lower the activation energy and improve the catalytic processes.^166^
C. The hole- and electron-transfer processes should be optimized at the cathode and anode, respectively.
D. Stability of the electrodes through the catalytic process in aqueous conditions and under continuous light irradiation.
E. Complementary wavelength absorbance should be used by two (or more) semiconductors^167^ to fully exploit the visible light wavelength range fully.

Over the last few decades, electrodeposition and ALD techniques have been developed and refined to enable precise control of the layer composition and thickness. These layers enabled enhanced stability, improved hole and electron transport, and cocatalyst integration for an efficient catalysis.

An interesting approach demonstrated the development of bias-free devices enabling CO_2_ or proton reduction from water.^168^ Figure 15 schematically presents the designed configuration. TiCo cocatalyst was deposited on the BiVO_4_-based photoanode to facilitate an efficient and stable water-oxidation reaction. The photocathode was constructed from an organic semiconductor blend, enabling efficient light absorption and adequate energy levels to support CO_2_ or proton reduction. The designed cell achieved a photovoltaic cell efficiency (PCE) of 10.7 ± 0.9%.

**Figure 15:**
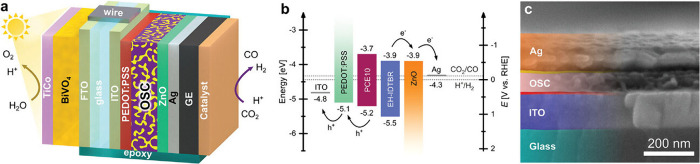
Design of OPV-BiVO_4_ artificial leaves. a) Schematic diagram of an unassisted OPV-BiVO_4_ artificial leaf. The PCE10:EH-IDTBR layer is illustrated as a violet-yellow blend. b) Energy level diagram of the OPV. Vacuum energy levels and redox potentials were obtained from the reported literature. c) Cross-section SEM image of an OPV cell. Adapted from ref 168. Available under a CC-BY 4.0 license. Copyright 2024 The Author(s).

## 5 Semiartificial Cells: From Biohybrid to Functional Cells

### 5.1 Photosensitizers Enzyme Hybrids for Fine Chemicals or Fuel Generation

#### 5.1.1 Developing Photocatalytic Biohybrids Performing Semiartificial Photosynthesis

Artificial photosynthesis seeks to capture sunlight and convert available feedstocks (e.g., H^+^, CO_2_, N_2_) into fuels and value-added chemicals.^169^ Abiotic photoelectrochemical devices typically achieve high solar-to-chemical conversion but often lack the remarkable selectivity of enzymes, which operate under mild conditions.^111^ Semiartificial photosynthetic biohybrid strategies bridge these strengths by interfacing inorganic light absorbers (e.g., semiconductors, molecular dyes) with biocatalysts (purified enzymes or whole cells).^170^

Upon illumination, the inorganic photoabsorber generates charge-separated states (e.g., promotion of electrons to the conduction band of a semiconductor or to an excited state of a molecular dye), which are then relayed to a biological acceptor to drive reductive biocatalysis (Figure 16).^171^ Electron transfer process can proceed either by direct electron transfer (DET) across a wired interface or by mediated electron transfer (MET) via a diffusible redox mediator. For MET, the mediator’s potential must lie between that of the excited photosensitizer and the acceptor to ensure thermodynamically favored electron transfer. Hole quenching is provided by a sacrificial electron donor (SED) that fills photogenerated holes (h^+^).^172^ Although water serves this role in oxygenic photosynthesis, efficient water oxidation remains challenging for most artificial absorbers. Consequently, practical systems commonly employ chemical SEDs such as triethanolamine (TEOA) or ascorbate.^173^

**Figure 16:**
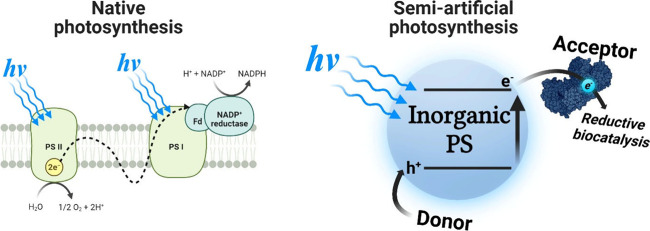
A comparison between native and semiartificial photosynthesis. Left – a schematic illustration of the light-harvesting process performed by photosystem I (PSI) and photosystem II (PSII) in the thylakoid membrane in photosynthetic organisms. Water is used as the sacrificial electron donor, while ferredoxin (Fd) and NADP+ reductase accept the excited electrons to facilitate the reduction of NADP+ to NADPH. Right – a schematic illustration of a typical semiartificial photosynthetic process. An inorganic photosensitizer (PS) is irradiated, resulting in the generation of excited electrons (e^–^) together with a positively charged hole (h+). An external sacrificial electron donor is required to fill the generated holes while a biological acceptor (typically a redox enzyme) consumes the electrons to drive reductive biocatalysis. Figure generated with Biorender.com.

Many semiartificial photosynthetic configurations focus on wiring hydrogenases, the main catalyst for biohydrogen production across diverse biological systems, to light harvesters catalyzing proton reduction to H_2_.^174–178^ Owing to its high energy density and the high efficiencies achievable in conversion devices (such as fuel cells), hydrogen is viewed as one of the most promising alternative carbon-free energy sources.^179^ While encouraging advances have been achieved, biological hydrogen generation often faces challenges for practical application due to parallel photochemical reactions that are typically more robust and efficient.^180^ Alternatively, biohybrid systems have been used to drive more complex reactions for the production of fine chemicals and value-added products while using the remarkable stereo- and enantioselectivity of enzymes.^170^ When the target biocatalytic product is chiral, enzymatic reduction offers a significant advantage over traditional chemical methods, which often yield a mixture of stereoisomers.^181^ In contrast, many oxidoreductase enzymes have evolved to produce a single stereoisomer with a near-complete enantioselectivity. This property is especially critical in the pharmaceutical industry, where insufficient enantioselectivity can lead to adverse effects. Therefore, we propose that the practical implementation of semiartificial photosynthetic systems should be directed toward highly selective commodities rather than the production of relatively simple end compounds. Additionally, numerous semiartificial photosynthetic systems aim to be incorporated to address global challenges, such as CO_2_ and N_2_ fixation.^182^ Work in semiartificial photosynthesis is commonly grouped into two classes: nanoparticle-enzyme assemblies (also coined *in vitro* biohybrids) and whole cells (*in vivo* biohybrids).

Enzymes have evolved to enable the variety of chemical reactions facilitated on Earth. The enzymes lower the activation barriers, which are translated into the chemical reaction toolbox used in nature. Biocatalyst structures often regulate selectivity and chirality, which are crucial for many essential processes. Natural machinery, however, comprises regulations that prevent the accumulation of a single product and the accompanying high energy costs in terms of ATP or NAD­(P)­H currency. Those can limit the production of specific compounds in excess, for example, in industrial applications. Coupling photodriven reactions with an enzymatic cascade opens a route to lower energy requirements toward continuous production of chemicals or fuels. Several challenges should be addressed in order to advance this direction into applications:

A. The photosensitizer should be stable, enabling continuous operation.
B. The photoactivated material should be nontoxic or easily separable from the product.
C. The electron transfer (or, in some cases, hole transfer) should be efficient, with minimal back or side reactions.
D. Photodriven reactions can generate reactive species that may damage the biocatalyst. Preventing or hindering such reactions is critical.
E. If a chiral product is required, the reaction should be carried out exclusively by the biocatalyst to limit interference from the photocatalyst surface or ligands, which can be nonselective.

#### 5.1.2 NP-Enzyme Biohybrids

As mentioned, Honda and co-workers showed that illuminated aqueous suspensions of photosensitive semiconductor powders can reduce CO_2_ to formic acid, formaldehyde, methanol, and methane.^183^ Bridging the gap between light-harvesting inorganic materials, often synthesized and handled in organic solvents, and aqueous biological systems, this work has laid the groundwork for research on the biotic-abiotic interface. The study of artificially activating purified redox enzymes using inorganic light-harvesting semiconductor quantum dots (QDs) was further explored in the late 2000s, with most research efforts focused on hydrogenase enzymes. A landmark study coimmobilized a [NiFeSe] hydrogenase and a ruthenium dye on TiO_2_ nanoparticles, establishing visible-light H_2_ evolution at room temperature and clarifying the principles of scaffold–enzyme coupling, Figure 17a.^177^ This system demonstrated efficient solar-driven hydrogen production, characterized by a high turnover frequency (TOF) and long-term stability. Related studies further explored coupling hydrogenase with various nanomaterials, such as CdS nanorods (NRs), CdTe quantum dots (QDs), and carbon dots to enhance hydrogen generation.^184,185^

**Figure 17:**
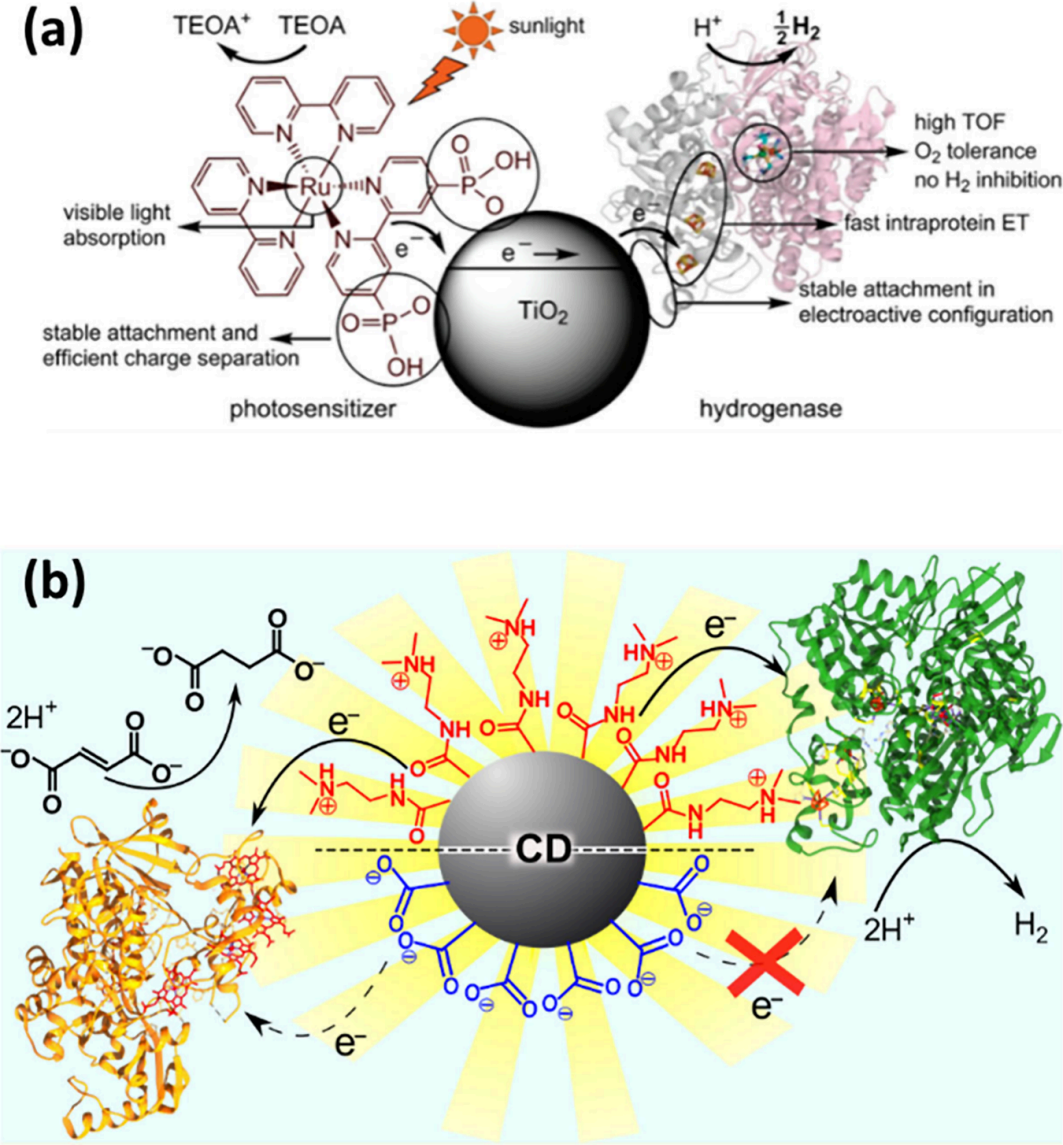
NP-hydrogenase hybrids for solar-driven hydrogen generation. (a) The activation of [NiFeSe]-hydrogenase by a synthetic ruthenium photosensitizer coattached to colloidal TiO_2_ NPs. Adapted from ref 177. Copyright 2009 American Chemical Society. (b) The effect of capping ligands on the electron transfer from light-irradiated carbon dots to hydrogenase and fumarate reductase. Adapted from ref.^184^ Available under a CC BY 4.0 license. Copyright 2016 The Author(s).

To improve quantum yield (i.e., the number of molecules converted relative to the number of absorbed photons), it is essential to favor electron transfer of photoexcited high-energy electrons to the enzyme over exciton recombination.^169^ To achieve this, many studies have focused on engineering the interfacial interactions between the biological and inorganic components, which critically influence electron transfer from the photoabsorber to the enzyme. These noncovalent interactions are primarily governed by the surface ligand design of the nanomaterials. A notable example was demonstrated by Reisner and colleagues, who showed that carbon dots capped with positively charged ammonium-terminated ligands could efficiently transfer photoexcited electrons to negatively charged enzymes (e.g., fumarate reductase and hydrogenase) with high efficiency and stability (Figure 17b).^184^

Alternatively, the inorganic photosensitizer can be covalently linked to the biological acceptor via bioconjugation.^186,187^ This covalent attachment minimizes the distance between the light-harvesting material and the enzyme, which is a key factor for enhancing electron-transfer rates. Such covalent anchoring is more common with molecular dye photosensitizers, due to their smaller size compared to nanoparticles.^174^

Extensive characterization of nanoparticle-hydrogenase biohybrids has paved the way for investigating more complex reductive transformations, such as CO_2_ reduction and nitrogen (N_2_) fixation, using various redox enzymes. As previously mentioned, there has been growing interest in reducing atmospheric CO_2_ due to its significant contribution to global warming. In 2019, Reisner and co-workers reported a system in which formate dehydrogenase (FDH) was coupled to TiO_2_ QDs functionalized with a ruthenium dye for photocatalytic reduction of CO_2_ to formate. Formate, a liquid and transportable product, serves as a valuable feedstock for fine chemical synthesis (see Figure 18a).^188^ In a recent study, photosensitizer-surfactant micelles that coassemble with redox enzymes (hydrogenases, FDH), formed enzyme-micelle hybrids that enable semiartificial photosynthesis in water with improved direct electron transfer operation for H_2_ evolution and CO_2_-to-formate conversion.^189^ Recently, MtrCAB, a multiheme transmembrane protein originating from *Shewanella oneidensis* MR-1, was incorporated into a transmembrane nanoreactor constructed by polar lipid extract and octyl glucoside. The micelle-based bioreactor encapsulated a hydrogenase enzyme that acted as a catalyst for H_2_ production. By coupling the hydrogenase-containing bioreactor with a carbon nitride photocatalyst, light-induced hydrogen generation could be observed incorporating MV to the inner compartment improved the catalytic performance to TOF 880 ± 154 h^–1^, which is doubled compared to the rates in its absence. This work introduces a new platform for semiartificial photosynthetic nanoreactors.^190^

**Figure 18:**
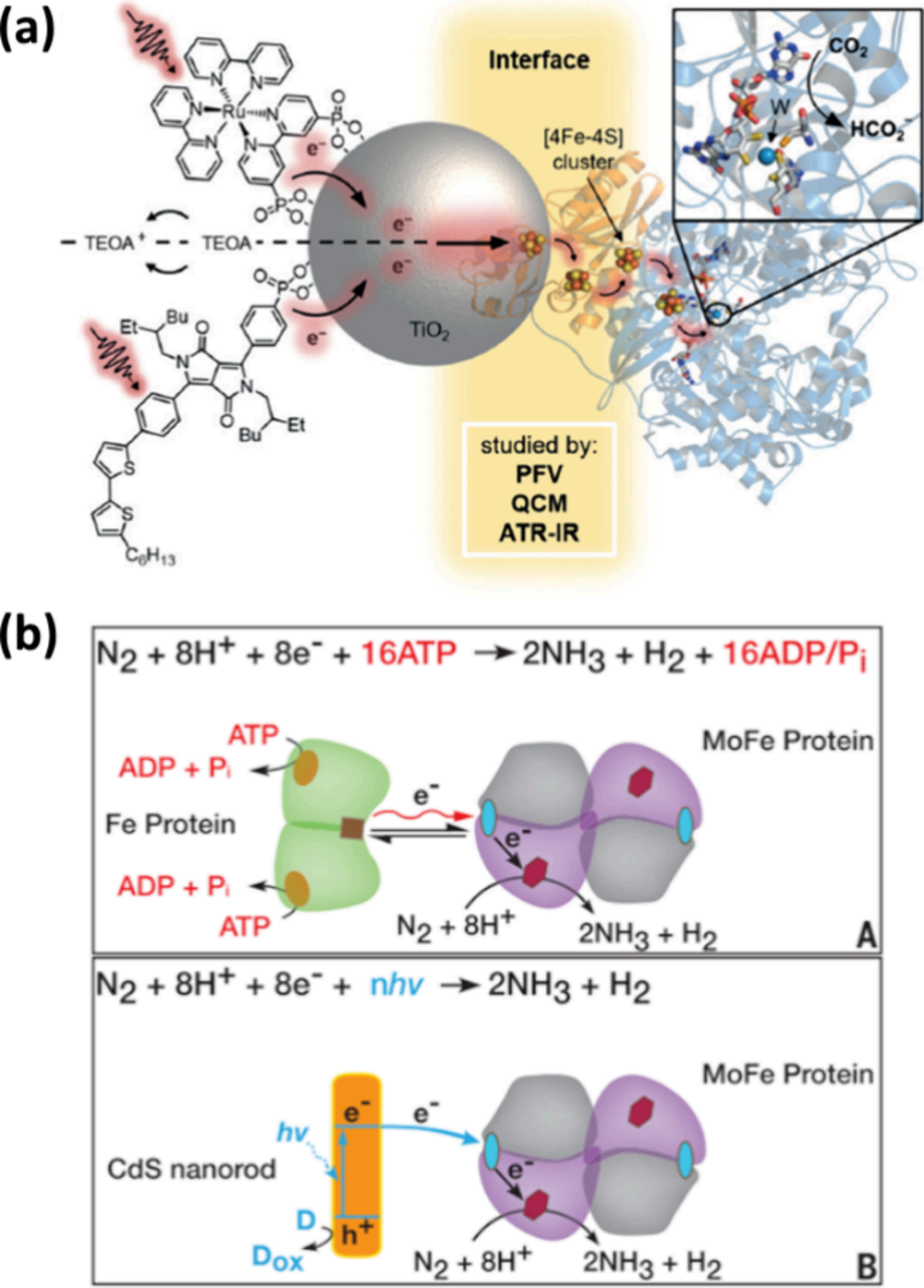
NP-enzymes biohybrids for CO_2_ and N_2_ fixation using sunlight as an energy source. (a) Demonstrating CO_2_ conversion with a dye–semiconductor–FDH photocatalytic system. Adapted from ref 188. Available under a CC BY 4.0 license. Copyright 2019 The Author(s). Licensed under (b). The artificial activation of Mo–Fe nitrogenase produces ammonia from nitrogen gas. (A) The native activity of Mo–Fe nitrogenase is equipped with an iron–sulfur cluster, a process that consumes 16 mol of ATP molecules per 1 mol of fixed nitrogen. (B) The artificial activation of the enzyme using CdS NRs as a light-harvesting unit, which transfers electrons directly into the enzyme’s active site. Adapted with permission from ref 191. Copyright 2016 AAAS.

One of the most energy-intensive industrial processes is the conversion of atmospheric nitrogen (N_2_) into ammonia (NH_3_) via the Haber–Bosch process, which accounts for over 1% of global energy consumption.^192^ A biohybrid photocatalytic approach successfully replicated this transformation using MoFe nitrogenase activated by CdS nanorods (Figure 18b).^191^ Naturally, the enzyme requires coupling with an iron–sulfur cluster, a process that consumes large amounts of adenosine triphosphate (ATP). Therefore, using light to power this enzyme offers a significant energy-saving alternative. This pioneering study has inspired many research groups worldwide to activate the nitrogenase enzyme using a nanobio interface artificially. Ding *et al.* report a CdS@ZnS-nitrogenase biohybrid that uses light to drive C–C coupling and hydrogenation of CO in water, producing hydrocarbons.^193^ It has been shown that product selectivity was tunable by illumination conditions, with higher photon flux favoring longer-chain products. Our lab has also investigated the incorporation of semiconductor QDs for the activation of MoFe nitrogenase. CdS QDs were synthesized with different capping ligands for either direct or mediated solar-driven ammonia production.^194^ In a follow-up work, the effect of the nanoparticle size on the NP-nitrogenase interface was demonstrated using CdSe QDs as photoabsorbers.^195^ It has been shown that biohybrids with small NPs resulted in higher H_2_ yields, possibly due to increased proximity of the small particles to the enzyme’s active site. Together, these examples highlight the vast potential of nanoparticle-enzyme biohybrid systems for the solar-powered production of fuels and essential chemicals.

While the construction of these biohybrids enabled photodriven enzyme activation, it required sacrificial electron donors, such as TEOA, for continuous activation, which limits their real-world applications. To tackle this issue, a Z-scheme configuration has been developed, Figure 19.^196^ The activation of the enzymatic reaction was achieved using two different semiconductor catalysts,^197^ BiVO_4_ and SrTiO_3_:La,Rh, to drive water oxidation and reduction processes, respectively. To enable efficient water oxidation and minimize back-reactions, a RuO_2_ cocatalyst was deposited on the BiVO_4_ particles. As described above, charge separation is crucial to efficient Z-scheme configurations. Therefore, Co­(bpy)_3_ was added to the reaction mixture and acted as a redox mediator between the oxidation and the reduction apparatus. While BiVO_4_ can oxidize water, its conductive band falls short of reducing directly protons or CO_2_. Hence, by coupling two semiconductors, we can achieve the potential required for the activation of the hydrogenase or formate dehydrogenase. This concept was further developed into biocatalytic CO_2_ reduction processes in the bacterial cells.

**Figure 19:**
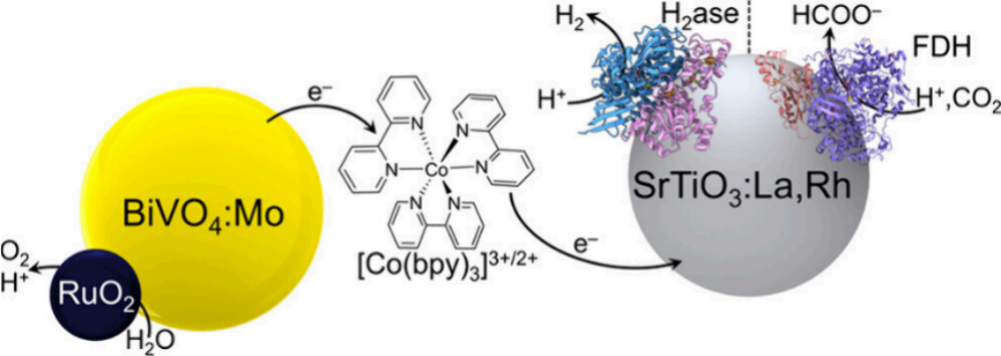
A Z-scheme configuration, which utilizes particulate SrTiO_3_:La, Rh, and BiVO_4_:Mo (light absorbers), hydrogenase or formate dehydrogenase (cocatalyst), and a molecular cobalt complex (a redox mediator). Adapted from ref 196. Available under a CC BY 4.0 license. Copyright 2024 The Author(s).

It should be noted that the semiconductors used have limited absorption in the visible range, and an improved energy-level design should extend their capacity to utilize a broader range of the visible spectrum.

As presented in the section, biotic-abiotic configurations for artificial photosynthesis can be versatile, comprising different enzymes and photosensitizers. We summarize the main findings of this part in Table 2. As presented, efficiency and stability parameters enable an easy comparison between the different systems. It should be mentioned that the dominant enzyme for biohybrid assemblies is hydrogenase. That is expected as green H_2_ was marked by many countries and official agencies as a critical green fuel for the different industries due to its high energy capacity (>120 MJ/kg), which is approximately 3 times that of gasoline. Also, bioengineering the proteins for extended performance and discovering new hydrogenases pushes further to advanced biotic-abiotic systems.

**Table 2:**
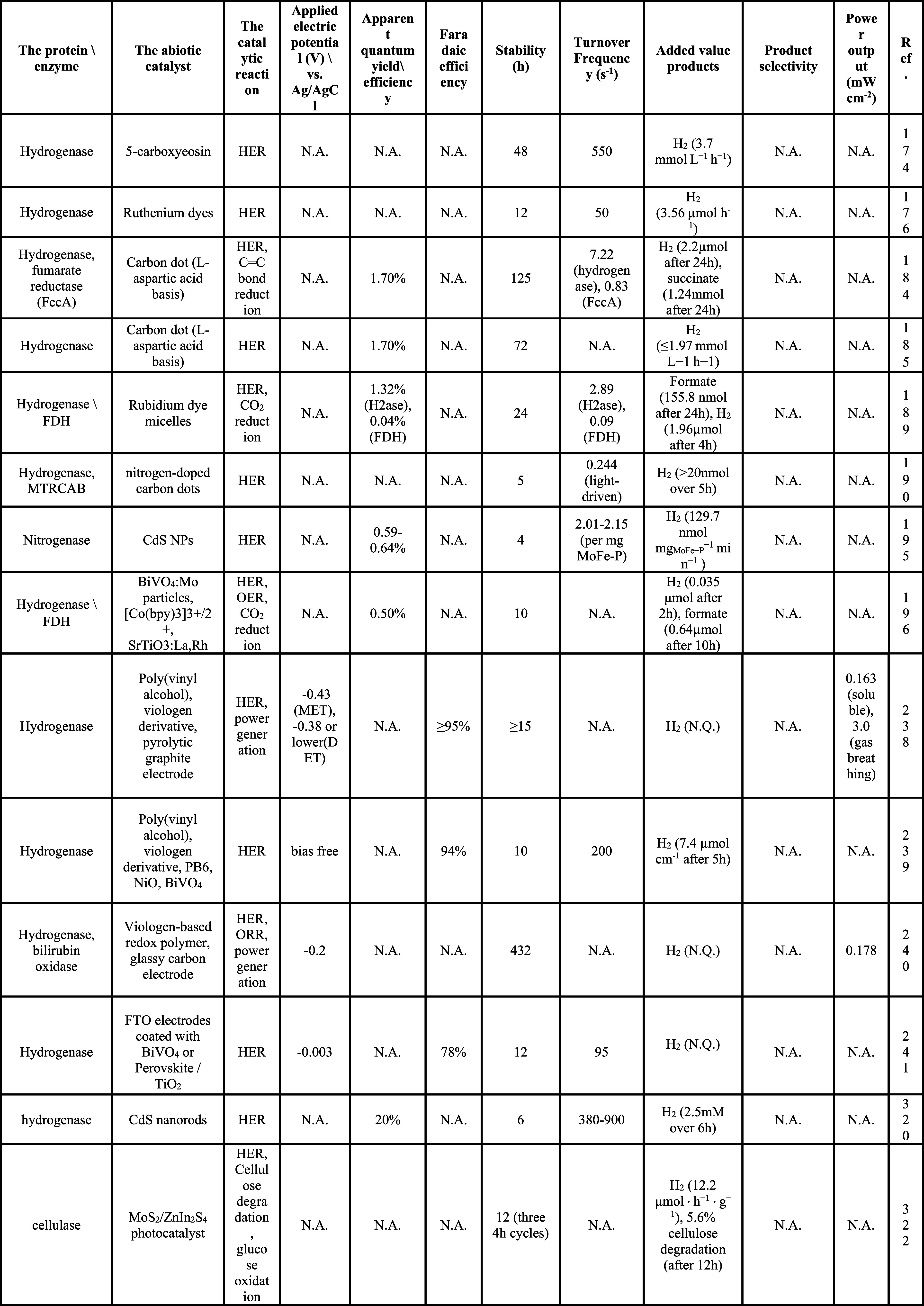

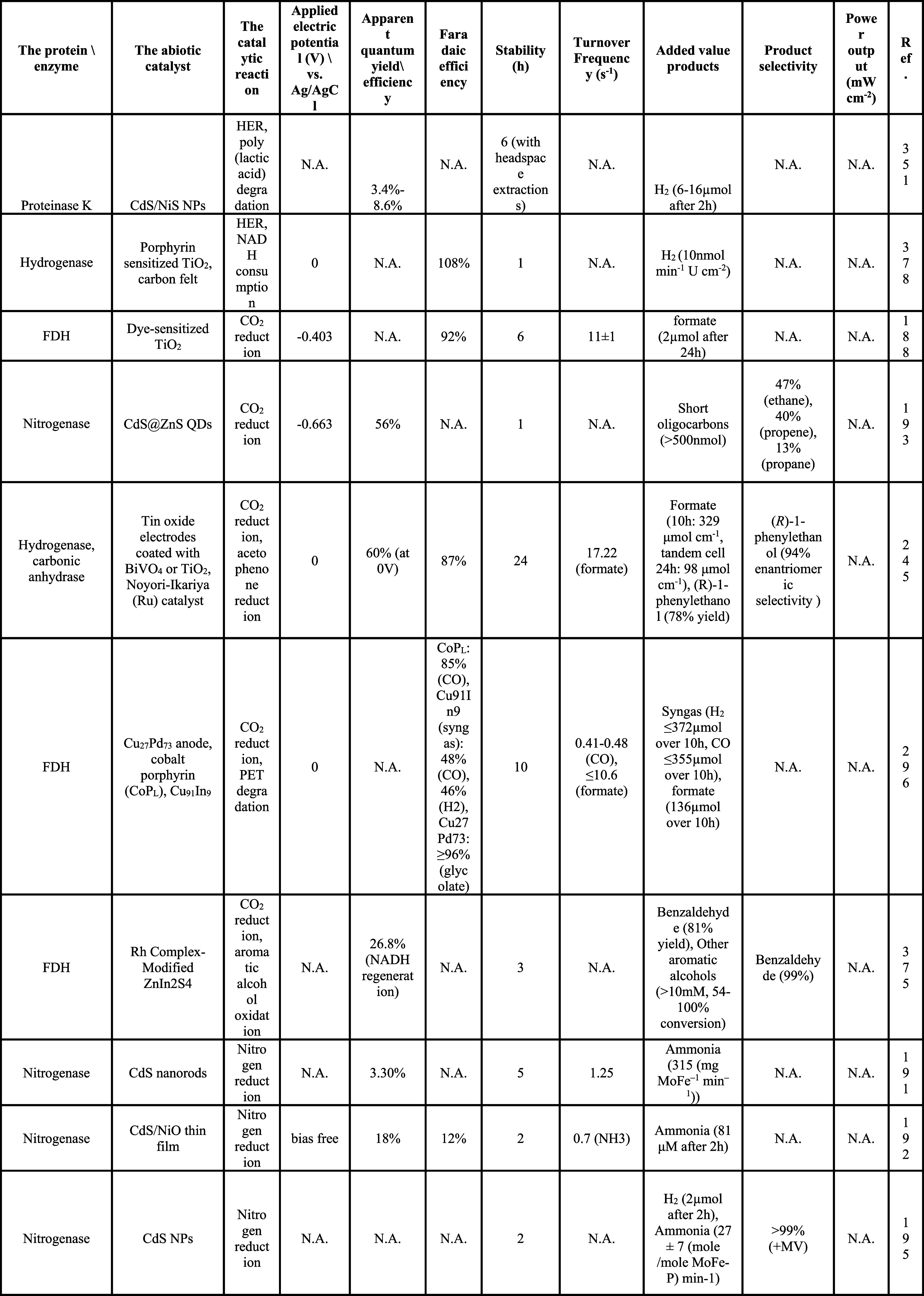

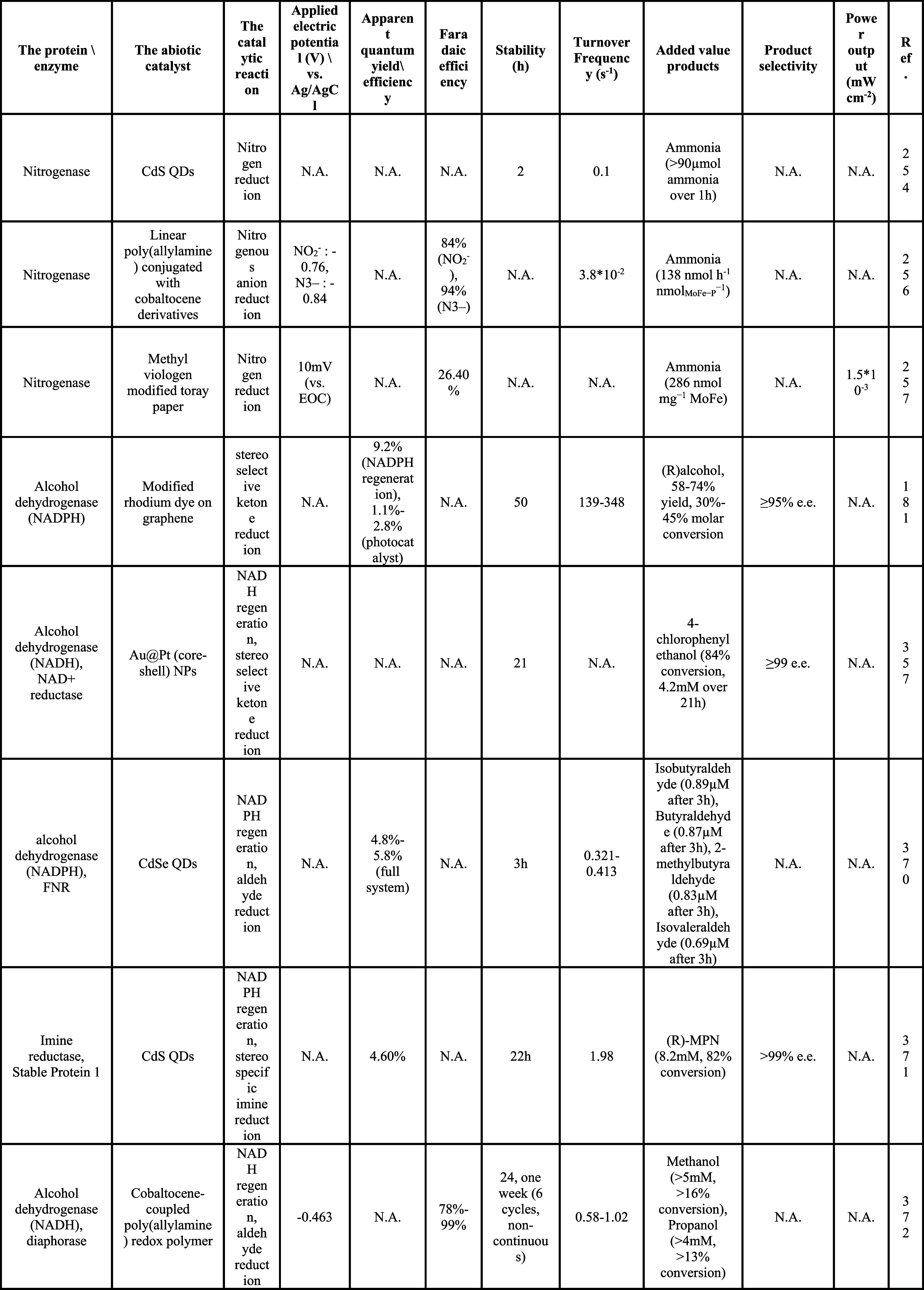

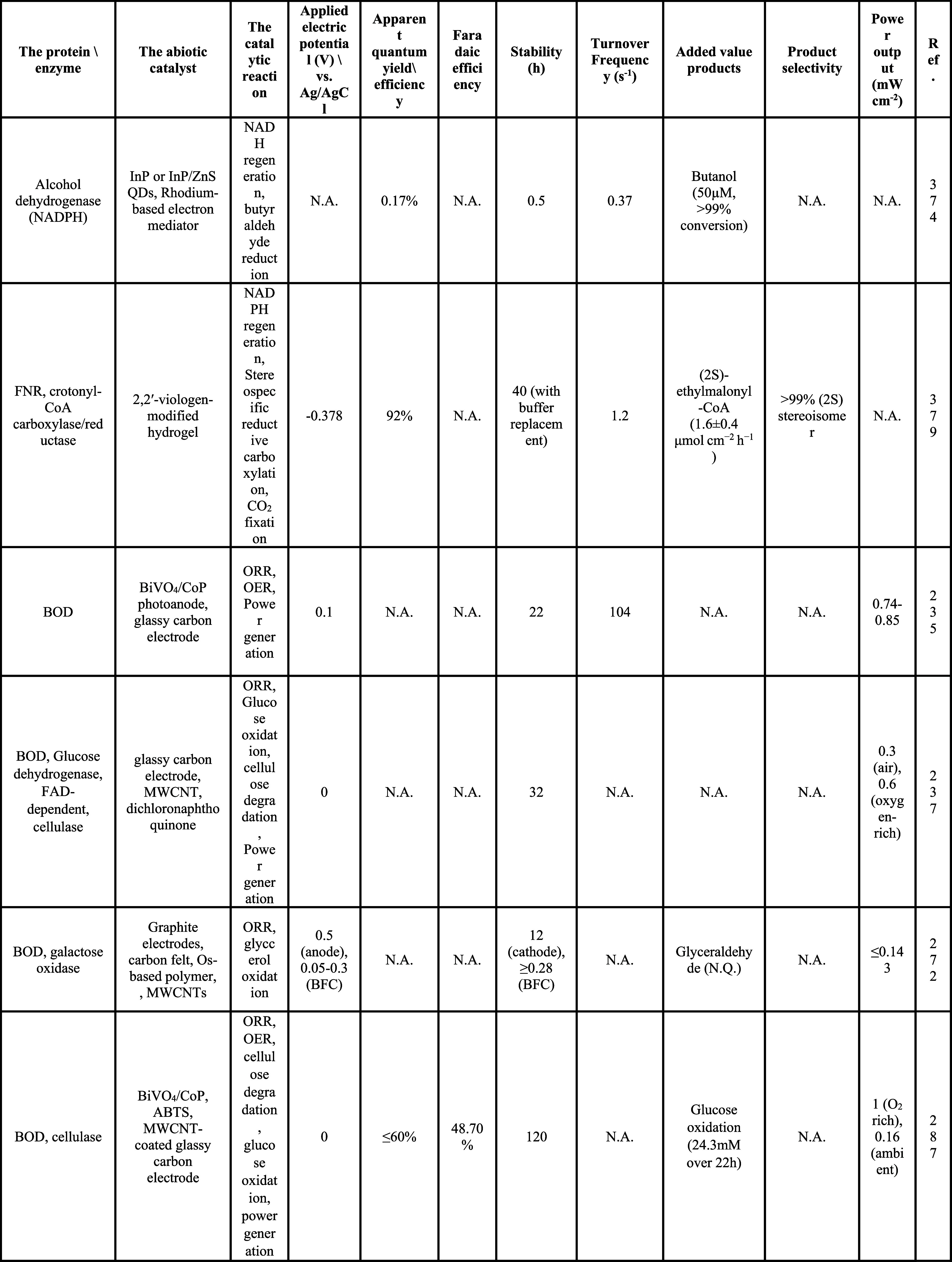

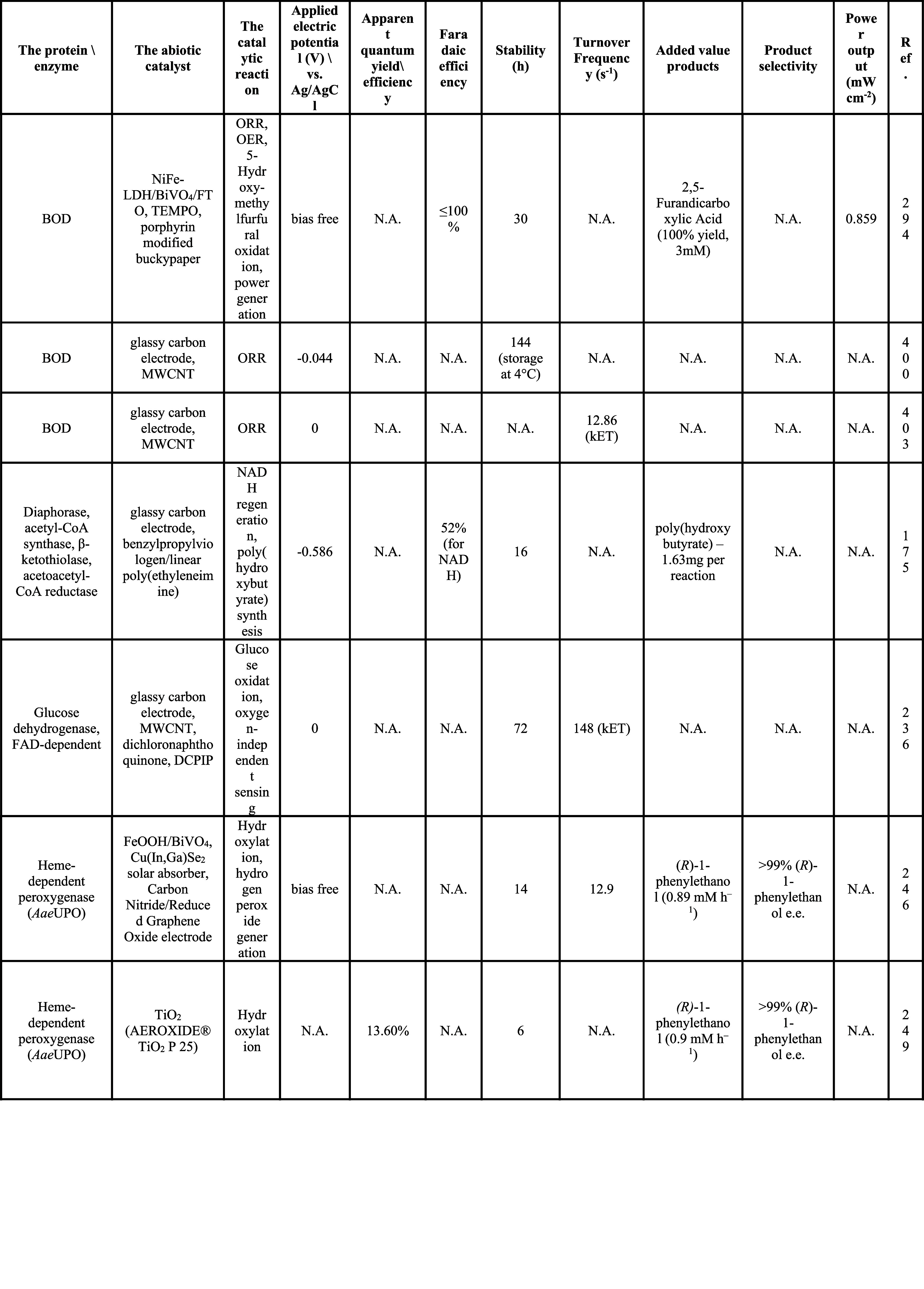

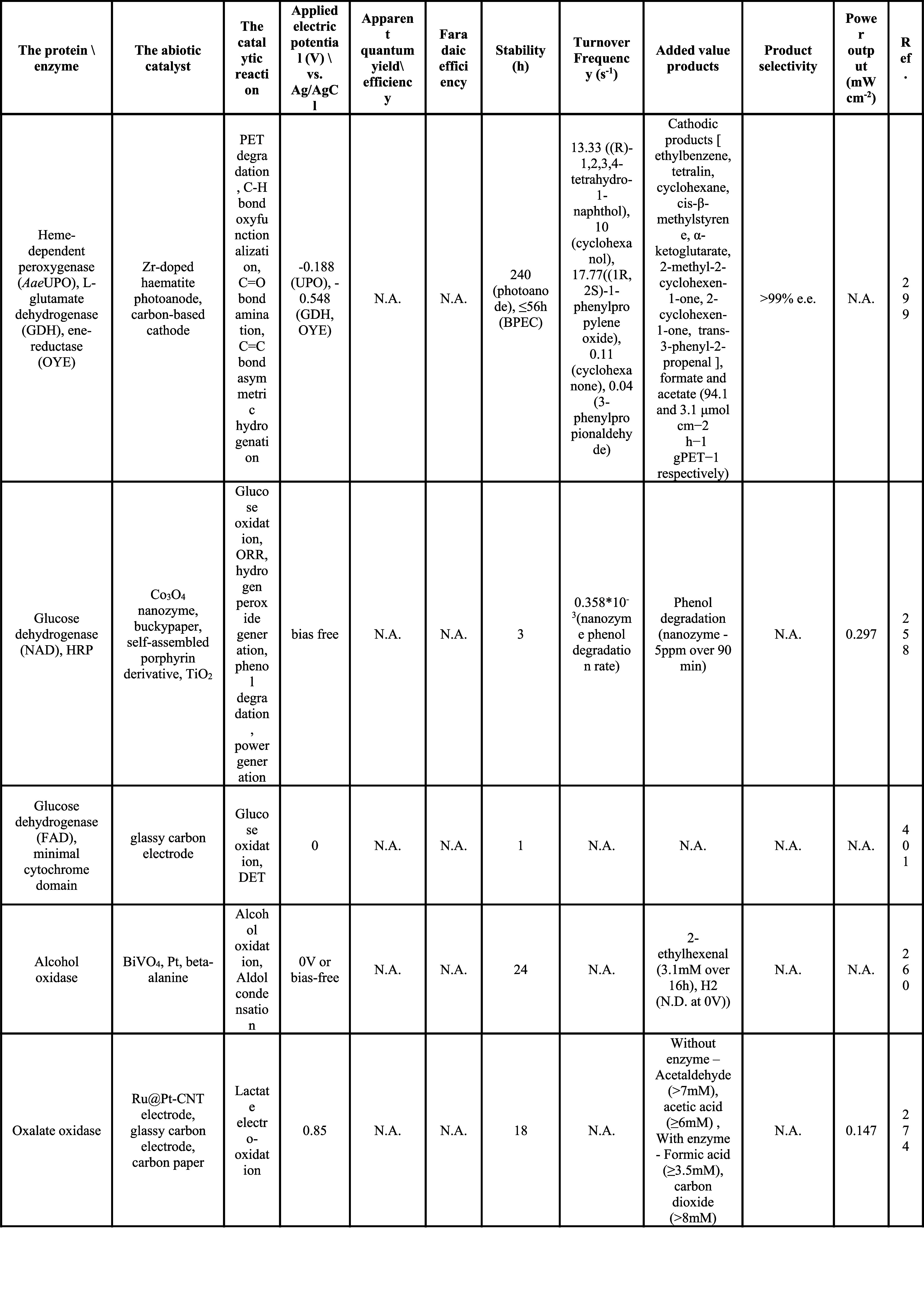

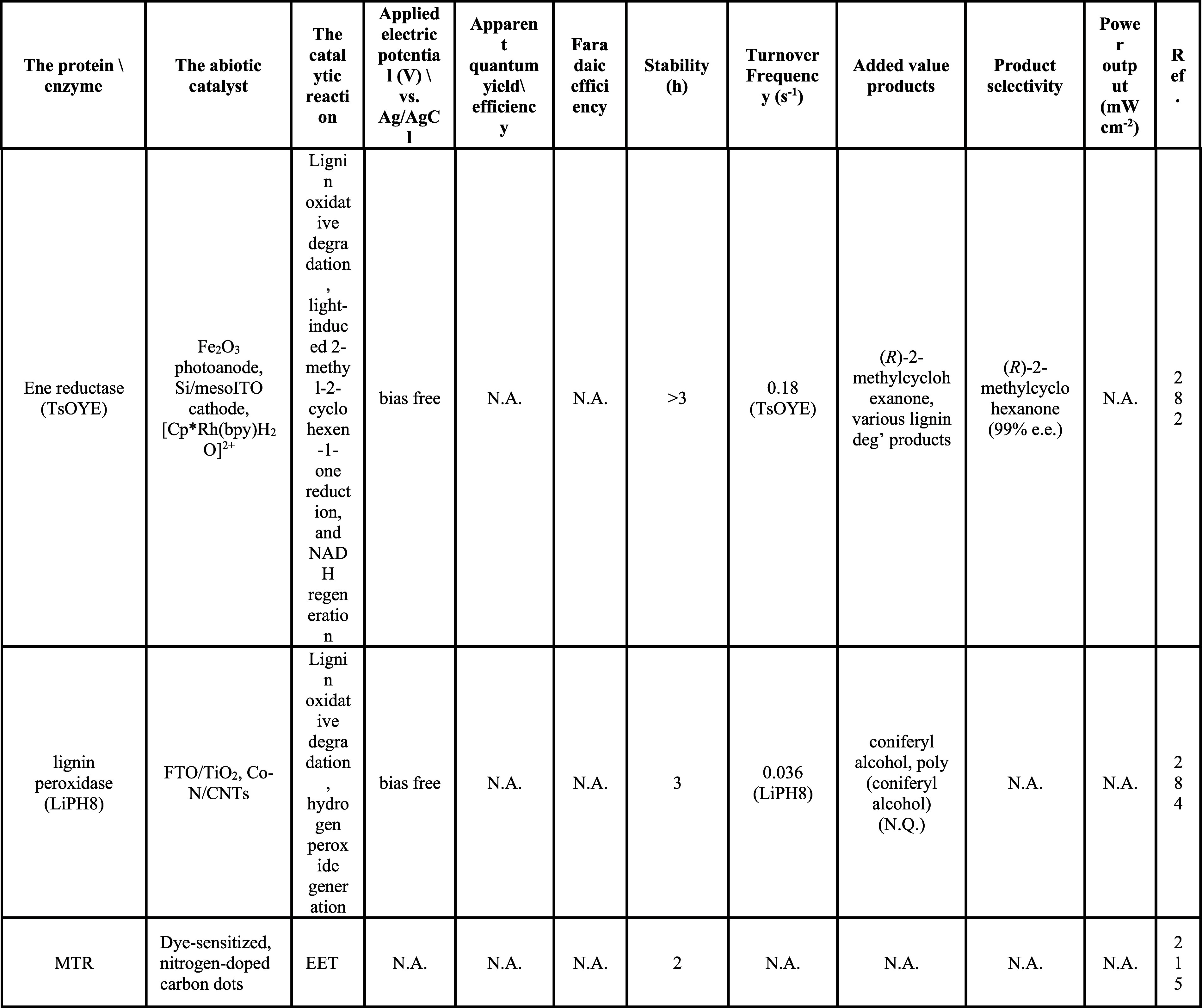
Protein-Based Biohybrids

a N.A. - not mentioned in the text.

b N.Q. - not quantified.

### 5.2 Whole Cell Biohybrids: Rewiring Nature

Compared to single-enzyme systems, whole-cell systems offer enhanced ability to catalyze complex, multistep reactions through integrated metabolic pathways and coordinated enzyme networks.^106,112,198^ Moreover, whole cells exhibit intrinsic regeneration capabilities, which contribute to greater operational longevity and efficiency while eliminating the need for protein purification and extensive maintenance.^199^ Nature itself offers an example in the form of photosynthetic microorganisms such as cyanobacteria, which naturally convert solar energy into chemical compounds. Significant efforts have been made to harness synthetic biology tools to redirect these native photosynthetic systems toward the production of value-added chemicals, rather than biomass or survival.^200^ Liang et al. demonstrate a biohybrid platform in which the cyanobacterium *Nostoc sphaeroides* self-assembles with indium-phosphide (InP) semiconductor nanoparticles to form a semiartificial photosynthetic system that drives enhanced production of the commodity chemical ethylene.^201^ The native ethylene-forming enzyme (Efe) in *N. sphaeroides* was verified, and the self-assembled InP-cyanobacteria hybrid showed an elevated photosystem I quantum yield and metabolic flux through ethylene production compared to unmodified cells. While promising, such efforts face substantial challenges due to the highly regulated nature of the photosynthetic apparatus, which limits its programmability. Consequently, recent research has increasingly focused on equipping nonphotosynthetic microorganisms with light-harvesting capabilities by integrating them with inorganic nanomaterials, which are not subjected to the same level of complex regulatory constraints.

When abiotic materials are integrated into a living system, toxicity against the host organism remains a major concern. Indeed, a large fraction of photosensitizer materials is composed of toxic elements, particularly heavy metals, that might interfere with essential biological functions.^202^ Figure 20 presents the major challenges that need to be addressed in order to design whole-cell biohybrids using internal or external stimuli. As presented, the cell membrane hinders a direct electron-transfer process and limits the activation of enzymatic cascades. In order to overcome the electrical insulation barrier, chemical or redox mediators can be used. Those could be based on natural redox-active proteins such as MtrCAB from *Shewanella* with OmcS from *Geobacter* that can be bioengineered to the desired host and enable cross-membrane electron transfer using heme moieties whose distances and orientation dictate the electron transfer rate. It should be mentioned that these natural electron transfer processes, which hold low reducing power thermodynamically, are limited to being used directly for cofactor regeneration.^203,204^ At pH 7, at least −0.62 V vs Ag/AgCl is required for H_2_ generation. Electrochemical measurements have shown that the CymA, the internal redox protein, is at least 200 mV more positive than the required potential. Even the more negative potential of OmcA or MtrC falls short.^204^ On the other hand, several reports presented electron transfer through those proteins to activate the hydrogenase enzyme for hydrogen generation.^190,205^ These differences could be explained by a small alteration in the protein structure while bonded to the membrane, or other structural changes that affect the redox potential.

**Figure 20:**
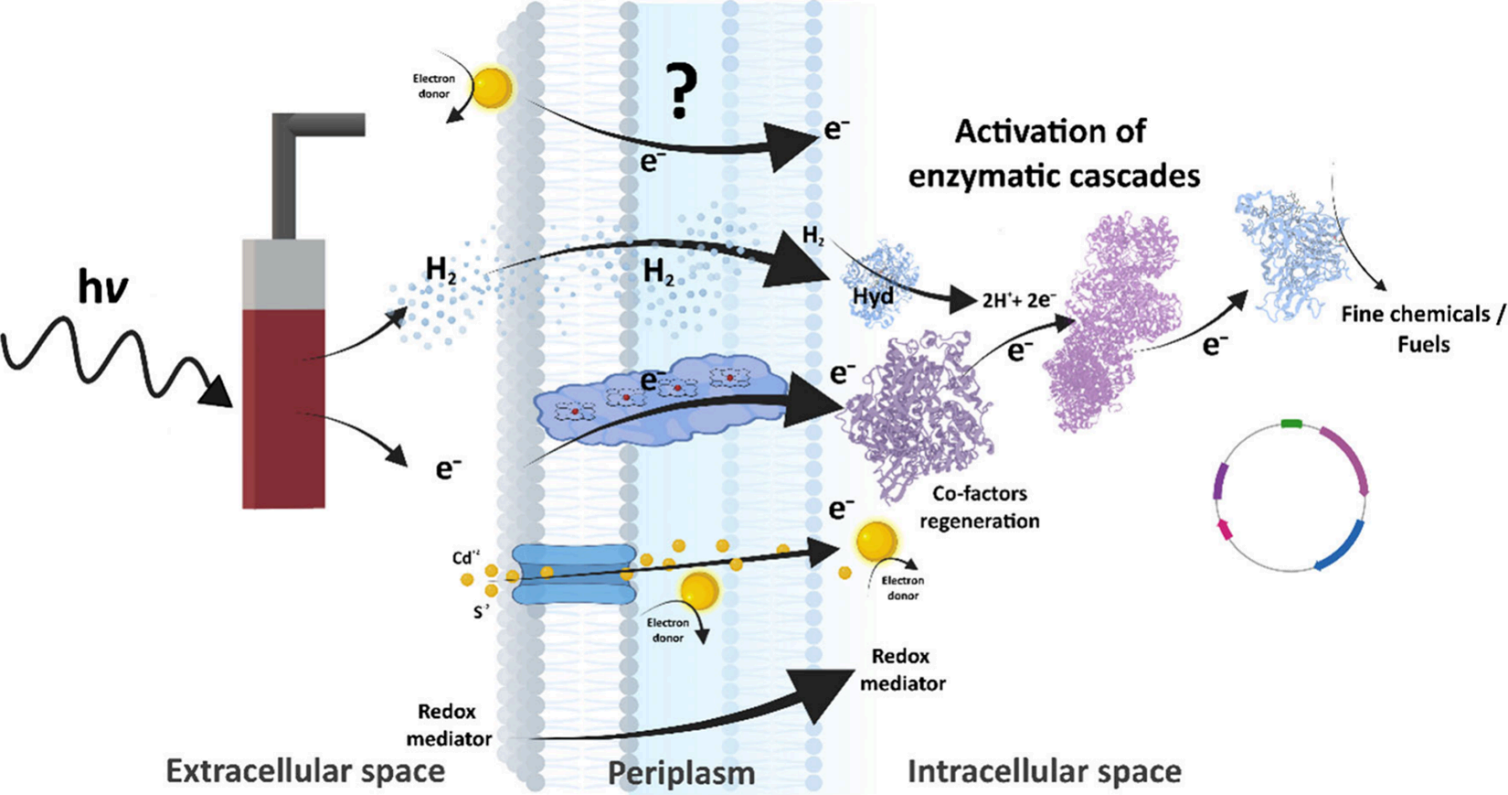
Schematic illustration of the electron transfer process between the abiotic electrode or NPs and a whole cell enzymatic biocatalysis. The activation could occur by gas diffusion into the cell through the membrane, by membrane-bound redox active proteins, or by diffusional redox mediators, all of which can be activated by electrodes or extrinsic photosensitizers. Alternatively, internally grown NPs can be used for photoactivation of overexpressed enzymes. By bioengineering, plasmids can be designed for different protein expression levels. The total scheme presents the leading routes toward interfacing abiotic with biotic and artificial photosynthesis configurations.

The phenomena can be found in other redox enzymes, for example, the activation by the nitrogenase enzyme by its electron donor, the iron protein.^202^ The interesting mechanism of the Omc and Mtr redox proteins should be fully elucidated, as it has great implications for whole-cell biocatalysis and future applications. Alternatively, photochemically or (photo)­electrochemically generated hydrogen can act as a chemical to electron carrier, enabling diffusion through the membrane. The hydrogenases can convert the generated H_2_ in the cell to energy currencies such as NADH. Alternatively, non-natural redox-active species, such as cobaltocene or viologens, can be used to enable transmembrane electron transfer. It should be noted that the periplasmic space can introduce changes in terms of trapped reducing equivalents or biocatalysts, which hinder desired processes. To establish efficient photoinduced electron transfer processes, the photocatalyst can be internally grown. This can be achieved by introducing salts into the host growth medium and allowing their absorption. Those methodologies can introduce toxicity, which can lead to a destructive outcome (see separate section on stability), or can lead to nonhomogeneous NP growth that limits their performance as photocatalysts. For further reading on this critical subject, a comprehensive study focusing on integrating an electrochemical biotic-abiotic biohybrid system was recently introduced.^203,204^

For example, chalcogenide-based semiconductors (e.g., CdS, CdSe, CdTe) are widely used in semiartificial photosynthetic systems, yet the presence of cadmium poses a significant biological risk.^106^ It should be noted that heavy metal toxicity is largely attributed to their ionic form (e.g., Cd^2+^, In^3+^) rather than their neutral form as nanomaterials.^206^ Once released, these cations interact with various cellular components, including enzymes and nucleic acids, causing an oxidative stress. Subsequently, many essential cellular processes could be damaged, including DNA replication, ATP synthesis, and membrane integrity.^207^ As mentioned, many organisms use biomineralization as a detoxification strategy to avoid the presence of such heavy metal ions.^206^ Hence, using directed and controlled biosynthesis of heavy-metal-containing nanomaterials can significantly reduce their toxicity.^208,209^

Photosensitizer cytotoxicity is not limited to heavy metals, as other organic light-harvesting compounds can inhibit cell viability. For instance, the widely used organic dye proflavine, an acridine derivative, exhibits strong antimicrobial activity through its reactivity with DNA and various essential cellular proteins.^210^ Therefore, employing heavy-metal-free and nontoxic photosensitizers (e.g., carbon dots) in semiartificial photosynthetic configurations holds great promise.^184^ By combining efficient light-harvesting capabilities with low cytotoxicity, such photosensitizers allow for improved biocompatibility, enhanced scalability, and low environmental impact.

A notable example of whole-cell semiartificial photosynthesis was reported in 2016 by Sakimoto and co-workers, who utilized the acetogenic bacterium *Moorella thermoacetica*, known for its ability to precipitate CdS particles on its surface.^211^ Remarkably, this process enables the bacterium to acquire photocatalytic activity. Upon light irradiation, these hybrid cells significantly enhanced the conversion of CO_2_ into acetic acid without the need for external electron mediators. This biomineralization occurs naturally as a detoxification response, wherein *M. thermoacetica* forms ∼ 10 nm CdS QDs on its surface when exposed to elevated Cd^2+^ concentrations. In their study, cysteine was introduced as both the sulfur source for QD formation and a sacrificial electron donor. When irradiated, the photoexcited QDs donated electrons, either directly or indirectly, to the Wood–Ljungdahl pathway, a native metabolic route responsible for CO_2_ fixation into acetic acid. Impressively, approximately 90% of the light energy harvested was directed toward acetic acid production, representing one of the first demonstrations of *in vivo* semiartificial photosynthesis.

Expanding on this concept, a similar system was later developed using externally synthesized gold nanoclusters (AuNCs) as the light-harvesting component.^212^ Owing to their semiconductor-like properties, small size (1–3 nm), and lower toxicity compared to cadmium-based materials, AuNCs have gained attention as a more biocompatible alternative. These nanoclusters exhibited improved cytoplasmic penetration, leading to enhanced acetic acid production within host cells.

The integration of genetic engineering with whole-cell biohybrid systems holds great potential to diversify the metabolites that can be produced *in vivo*. Genetically tunable organisms such as *E. coli* and S. cerevisiae offer ideal platforms for implementing electron-driven biocatalytic processes. For instance, Ishihara et al. (2016) reported the coupling of commercially available anatase-phase TiO_2_ nanoparticles with recombinant *E. coli* expressing [Fe–Fe]-hydrogenase genes for light-driven hydrogen production (Figure 21a).^213^ Methyl viologen (MV) served as an electron mediator, exploiting its membrane-permeable nature and redox activity. This study marked the first successful example of using a recombinant *E. coli* and inorganic NP hybrid for photocatalytic hydrogen generation.

**Figure 21:**
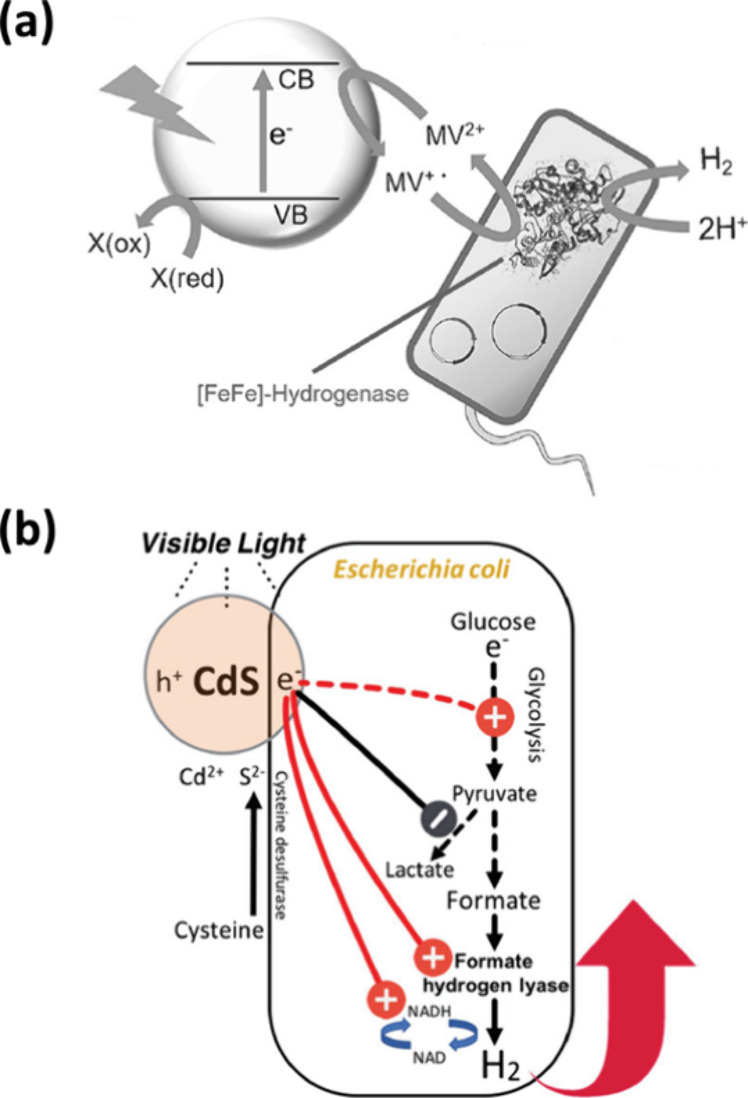
Enhanced hydrogen evolution in recombinant *E. coli* coupled with inorganic semiconductors. (a) TiO_2_ anatase semiconductor enabled photocatalytic production of hydrogen, using [Fe–Fe]-hydrogenase and methylviologen (MV) as an electron transfer mediator. Adapted with permission from ref 213. Copyright 2016 Wiley-VCH GmbH (b) Precipitation of CdS QDs on the cell due to the expression of a sulfur-reducing enzyme, followed by enhanced hydrogen production by endogenous [Ni–Fe]-hydrogenase. Adapted with permission from ref 214. Copyright 2017 Wiley-VCH GmbH.

In a complementary study published in 2017, Wang and co-workers engineered *E. coli* to biosynthesize CdS QDs on their surface for hydrogen production (Figure 21b).^214^ This was achieved by expressing *cysteine desulfhydrase*, an enzyme that converts cysteine into hydrogen sulfide (H_2_S), a necessary precursor for CdS precipitation. Under anaerobic and illuminated conditions, the activity of native [Ni–Fe]-hydrogenase in *E. coli* was significantly enhanced, resulting in an additional 400 μmol of hydrogen over 3 h compared to unmodified controls.

Metabolomic profiling further revealed substantial shifts in key intracellular metabolites (e.g., NADH, pyruvate, lactate, and formate), indicating the significant metabolic impact of the light-driven biohybrid activity.

As the field of light-powered whole-cell biohybrids continues to expand, most studies have focused on hydrogen production, either via native or recombinant hydrogenases. However, a significant challenge remains in distinguishing hydrogen generated directly by the semiconductor from that produced via biohybrid electron transfer pathways.^215^ Electron transfer (ET) from inorganic photosensitizers to target redox enzymes within cells is often hampered by two primary limitations: (i) the complex intracellular environment, which can lead to off-target electron transfer, and (ii) spatial separation between key functional modules, which reduces ET efficiency and overall product yields.^216^

To overcome these challenges, various strategies have been developed to improve the extracellular electron transfer (EET) in whole-cell biohybrids. One prominent approach leverages the *Mtr* complex, a native multiheme electron conduit in the electroactive bacterium *Shewanella oneidensis* MR-1.^217,218^ This system enables the exchange of electrons between the cell and its external environment. The *Mtr* pathway has also been heterologously expressed in *E. coli*, significantly enhancing EET capabilities.^217^ Inspired by this mechanism, Li and colleagues engineered a modular system wherein the outer membrane protein OmpA was fused with a TiO_2_-binding peptide to facilitate close coupling between TiO_2_ nanoparticles and intracellular redox machinery (Figure 22a).^219^ This design led to an 81-fold increase in hydrogen production mediated by the hydrogenase enzymes. Kalathil and colleagues have offered an elegant strategy to overcome EET limitations. In their study, a synthetic light harvester (carbon nitride) is interfaced with a two-microbe coculture.^220^ First, the inorganic light-harvesting module is interfaced with the electrically conductive bacterium *Geobacter sulfurreducens* (rich in multiheme c-type cytochromes), thereby enabling efficient EET. Then, the excited electrons are transferred to the methanogenic archaeon *Methanosarcina barkeri* via a conductive protein filament, allowing for solar-driven CH_4_ formation.

**Figure 22:**
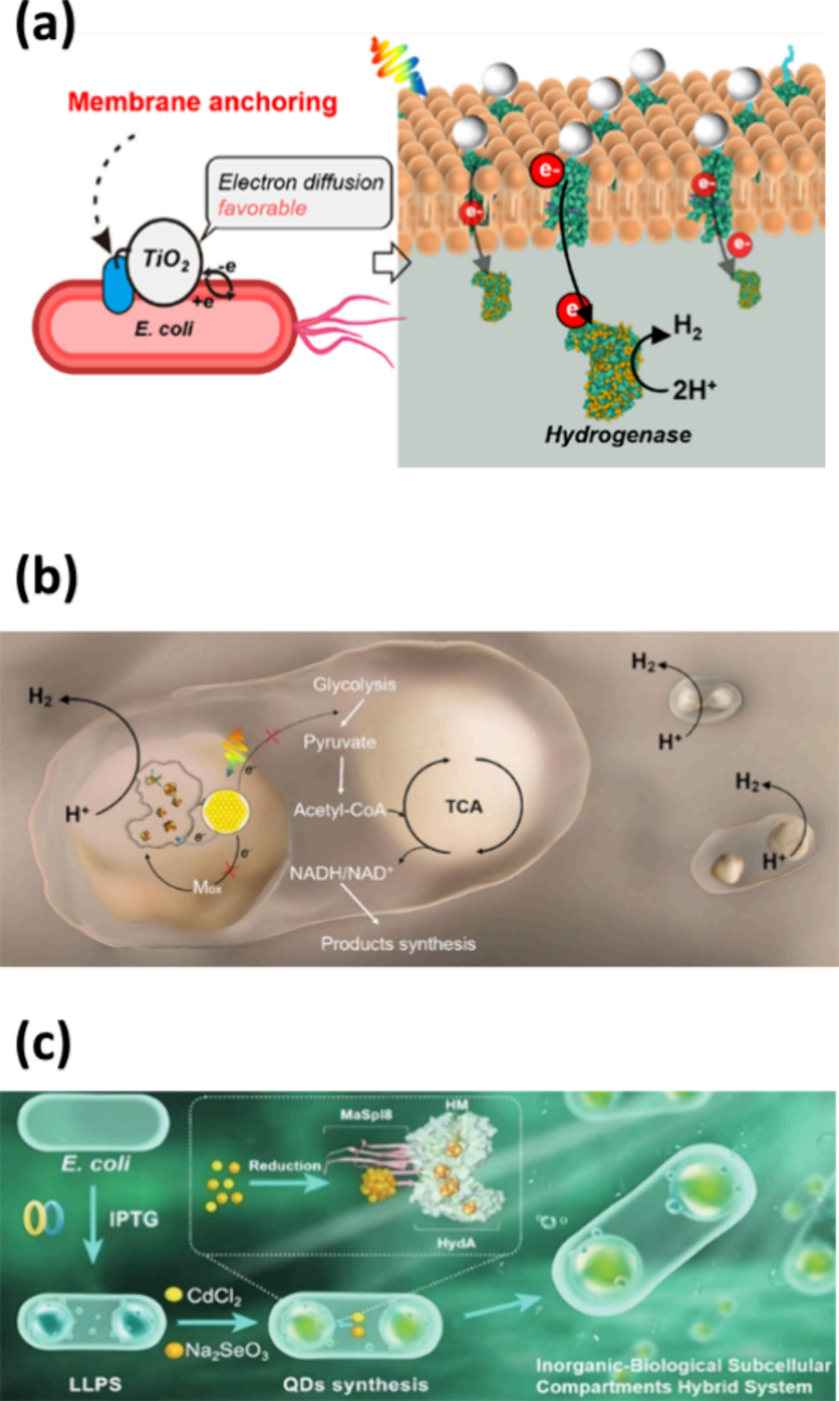
Establishing extracellular electron transfer (EET) in photosynthetic whole-cell biohybrids. (a) Using a membrane anchoring domain to establish a new NP-acceptor interface for enhanced EET. The anchoring protein OmpA binds both light-harvesting TiO_2_ NPs and the hydrogenase enzyme, generating a directed electron transfer pathway for improved light-driven hydrogen production. Adapted from ref 219. Copyright 2024 American Chemical Society. (b) and (c) Employing a liquid–liquid phase separation (LLPS) strategy to overcome limited EET in whole-cell biohybrid systems using gold nanoclusters and CdS QDs as light-harvesters, respectively. Adapted with permission from ref 224. Copyright 2024 Elsevier, Adapted from ref 225. Available under a CC BY 4.0 license, Copyright 2024 The Author(s), respectively.

A more straightforward and widely used approach involves the application of membrane-permeable redox mediators to shuttle electrons from external light-activated materials to intracellular targets.^172^ While effective, this strategy has notable drawbacks. Many redox mediators (such as MV) have limited biocompatibility^221^ and are known toxins (some were initially developed as herbicides).^222^ Their membrane permeability and intracellular distribution are also often unclear, and the reliance on exogenous chemicals limits system sustainability and scalability.^200^ Alternatively, by bioengineering, one can overexpress and tune natural redox mediators such as phenazines or derivatives to enable MET without toxicity.^223^

Another innovative strategy to overcome spatial compartmentalization involves the engineering of membraneless organelles within cells. In one such study, Li et al. colocalized AuNCs and hydrogenase enzymes inside *E. coli* using liquid–liquid phase separation (LLPS).^224^ A recombinant fusion protein containing hydrogenase and an intrinsically disordered region was used to facilitate phase-separated compartment formation (Figure 22b). Additionally, histidine-rich tags were introduced into hydrogenase to enhance its interaction with the AuNCs, resulting in a 40-fold enhancement in hydrogen production compared to that of control systems. In follow-up work, the same team demonstrated *in situ* synthesis of CdS QDs using hydrogenase as a capping agent (Figure 22c), creating defined intracellular nanoenvironments with improved photostability and catalytic performance.^225^ Luo et al. constructed a whole-cell biohybrid system by incorporating CuInS_2_/ZnS quantum dots into the periplasm of the electroactive bacterium *Shewanella oneidensis*, thereby enabling light-driven hydrogen production.^226^ Importantly, by colocalizing the nanomaterials and the acceptor hydrogenase enzyme within the periplasmic space, the authors overcome electron transfer limitations. Thus, the periplasmic strategy offers a promising route to improve robustness and modularity of semiartificial photosynthetic systems.

An increasingly attractive alternative is to enable whole cells to biosynthesize inorganic nanomaterials from metal salt precursors.^209^ This bottom-up approach eliminates the need for presynthesized nanomaterials, supports self-sustaining systems, and allows for continuous regeneration and turnover of active components.^170^ Furthermore, such biosynthesis can occur intracellularly, potentially bypassing EET limitations caused by compartmentalization. Kim and co-workers report an elegant whole-cell biohybrid approach in which the diazotroph *Azotobacter vinelandii* biosynthesizes cadmium-sulfide (CdS) semiconductor nanoparticles intracellularly by supplying CdCl_2_ and leveraging endogenous cysteine desulfurase activity to generate the sulfide precursor.^182^ Under illumination, the excited electrons in the *in vivo*-grown CdS QDs are transferred to the native nitrogenase enzyme, boosting light-driven ammonia production.

Bachar and co-workers presented a novel strategy for constructing whole-cell biohybrids based on the SP1 self-assembled protein scaffold to guide the biosynthesis of size-constrained inorganic nanomaterials within living cells (please see section 9.1).^209^ Stable protein 1 (SP1) is a homooligomeric protein comprised of 12 identical subunits that assemble into a ring-shaped structure with an inner diameter of ∼ 3 nm.^227^ This engineered nanocage functions as a template for nanomaterial production in the cell, endowing the host organism with entirely new capabilities. We showed that integrating semiconductor QDs biosynthesis could facilitate intracellular electron transfer to drive desirable redox transformations.

While most self-synthesized nanoparticle systems lack precise control over particle characteristics, protein-mediated biosynthesis offers a more tailored approach. For example, Wei and colleagues used a surface-display strategy to express the metal-binding protein PbrR on *E. coli*, facilitating the controlled formation of CdS QDs directly on the cell surface.^228^ This resulted in smaller, more uniform QDs with improved photocatalytic performance and reproducibility. Wang and co-workers developed a biotic-abiotic configuration that fully converts CO_2_ and H_2_O into chemical fuels and oxygen using solely light irradiation as an energy source.^229^ Here, the Z-scheme photocatalysts Cr_2_O_3_/Ru-SrTiO_3_:La,Rh|ITO|RuO_2_-BiVO_4_:Mo facilitated water splitting to oxygen and H_2_. The produced hydrogen was continuously uptaken into *S. ovata* bacteria, enhancing the production of acetic acid, Figure 23. The system presented a decline in activity after 15 h of operation. The authors attribute the activity loss to the Cr_2_O_3_ cocatalyst degradation. Indeed, the activity was restored by redepositing the Cr_2_O_3_ for at least 45 h with 82% activity as compared to the initial run. The generated acetic acid was further utilized for a “dark cycle” in which *G. sulfurreducens*, a commonly used bacterium in microbial fuel cells, consumed the acetic acid while generating electrical current. The work demonstrates the advantages of using biotic-abiotic interfaced systems, for example reloading cocatalyst similarly to the D1 replacement that occurred in PSII. The work presents a Z-scheme light cycle coupled with a dark cycle toward chemical production or electrical energy generation.

**Figure 23:**
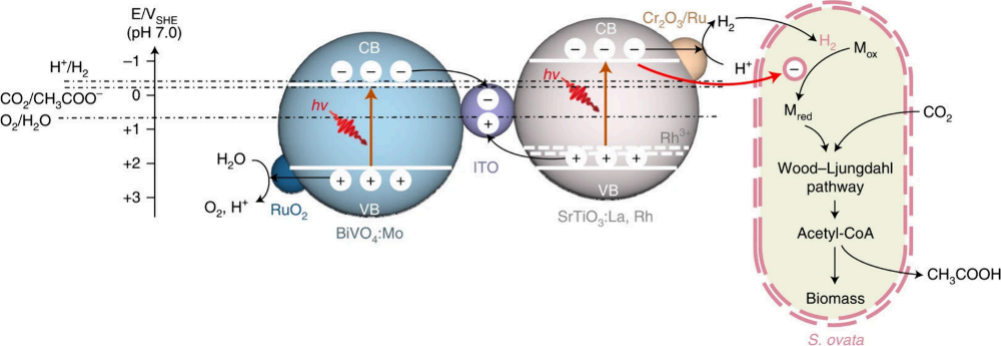
Schematic representation of a biotic-abiotic interface configuration that converts CO_2_ and H_2_O into chemical fuels and oxygen using solely light irradiation as an energy source. The system comprises Cr_2_O_3_/Ru-SrTiO_3_:La, Rh|ITO|RuO_2_-BiVO_4_:Mo that facilitates the water oxidation reaction to yield oxygen and hydrogen. The latter was uptaken into *S. ovata* bacteria, enhancing the production of acetic acid. Adapted with permission from ref 229. Copyright 2022 SNCSC.

As shown, whole-cell biohybrid configurations hold great potential for artificial photosynthetic systems. Transforming nonphotosynthetic bacterial cells into light-induced machineries by introducing abiotic photosensitizers may lead to real-world applications. Using whole-cell bacteria should allow a straightforward transformation into large-scale production. Table 3 summarizes the leading biotic-abiotic whole-cell configurations presented in this section.

**Table 3:** Whole-Cell Biohybrid

The microorganism	The abiotic catalyst	The catalytic reaction	Applied electric potential (V) \ vs Ag/AgCl	Apparent quantum yield/efficiency	Faradaic efficiency	Stability (h)	Added value products	Power output	Product selectivity	Ref.
*E. coli*, recombinant	[Ru(bpy)3]2+, Eosin Y, methyl viologen	HER	N.A.	1.50%	N.A.	120	H_2_ (138 nmol mL^–1^ h^–1^)	N.A.	N.A.	^172^
*E. coli*, recombinant	Eosin Y photosensitizer	HER	N.A.	1.1% (over 5 h)	N.A.	24	H_2_ (1.6 μmol mL^–1^ OD600^–1^)	N.A.	N.A.	^178^
*E. coli*, recombinant	CdS NPs	HER	N.A.	≤5.94%	N.A.	12	H_2_ (13.4 mmol over 12 h)	N.A.	N.A.	^208^
*E. coli*, recombinant	TiO_2_, methyl viologen	HER	N.A.	1.57%	N.A.	15	H_2_ (216 μmol over 15 h)	N.A.	N.A.	^213^
*E. coli*	CdS NPs	HER	N.A.	7.93%-9.59%	N.A.	3	H_2_ (>1.8 mmol μmol over 3 h)	N.A.	N.A.	^214^
*E. coli*, recombinant	Graphite felt electrode	EET	0.2	N.A.	N.A.	96	N.A.	N.A.	N.A.	^217^
*E. coli*, recombinant	TiO_2_ photosensitizer, methyl viologen	HER	N.A.	5.68%, 4.06% at *t* = 12 h	N.A.	12	H_2_ (3.6 μmol over 12 h)	N.A.	N.A.	^219^
*E. coli* strain 1/HM, recombinant	AuNPs, methyl viologen	HER	N.A.	20.30%	N.A.	168	H_2_ (>80 μmol over 24 h)	N.A.	N.A.	^224^
*E. coli*, recombinant	CdSeS NPs	HER	N.A.	11.63%	N.A.	27 (using 3h “on” and “off” cycles)	H_2_ (650.5 μmol over 15 h)	N.A.	N.A.	^225^
*Shewanella oneidensis* MR-1	CuInS_2_/ZnS	HER	N.A.	15.02%	N.A.	48	H_2_ (≤1.6 mmol over 48 h)	N.A.	N.A.	^226^
*E. coli*, recombinant	CdS NPs	HER	N.A.	N.A.	N.A.	96	H_2_ (81.8 μmol over 24 h)	N.A.	N.A.	^228^
*X. autotrophicus* 7C	CoPi, Co–P alloy cathode	HER, OOR, CO_2_ fixation, acetylene reduction	3	N.A.	4.50%	120	Ammonia (rate of 1.9 × 10^4^ molecules cell^–1^), ethylene (127 μM h^–1^ OD600^–1^)	N.A.	N.A.	^251^
*Shewanella oneidensis* MR-1	Eosin Y, proflavine, Ru(bpy)_3_^2+^, fluorescein, methyl viologen	HER, CO_2_ reduction, pyruvate reduction, fumarate reduction	N.A.	0.5%-2.1%	N.A.	96	H_2_ (1 μmol over 24 h)	N.A.	93% lactate (from pyruvate), 70% succinate and 30% malate (from fumarate)	^218^
*Shewanella oneidensis* MR-1	CuO/ZnO/CuO photoanode	HER	–0.8 ∼ −0.85	N.A.	20%-90%	48	H_2_ (13.6 mmol m^–2^ day^–1^)	N.A.	N.A.	^307^
*Azotobacter vinelandii*	CdS QDs	nitrogen reduction	N.A.	N.A.	N.A.	8	ammonia (3.56 mg g^–1^ cells over 8 h)	N.A.	N.A.	^182^
*Geobacter sulfurreducens KN400, Methanosarcina barkeri*	Carbon nitride photosensitizer	CO_2_ reduction, Interspecies electron transfer	N.A.	N.A.	N.A.	120 per cycle (three cycles recorded)	CH_4_ (110 μmol over 120 h)	N.A.	Up to 100% methane	^220^
*Chlorella minutissima*	ITO-WO_3_–PDA electrode	CO_2_ reduction	0.5	4.20%	86%	1	formate (4.2 μM after 1 h), 24 μA cm^–2^	N.A.	N.A.	^273^
*Methanobacterium* sp., *Hydrogenophilaceae*	TiO_2_/CdS, carbon paper electrode	CO_2_ reduction	bias free	N.A.	94.40%	144	CH_4_ (15L m^–2^ day^–1^)	N.A.	N.A.	^305^
Microbe-containing wastewater	Ni single-atom catalysts, Si wires	CO_2_ reduction, hydrogen production	0.294	N.A.	>96.6% (CO H_2_ mixture)	45	Gas mixture (45 μmol H_2_ and 30 μmol CO after 45 h), 1.0–1.1 mA cm^–2^	N.A.	60% H_2_,40% CO (after 45h)	^306^
*Synechocystis sp.* PCC 6803	Dichloro-benzoquinone, ITO, Pt	CO_2_ fixation, EET	–0.497 to −0.697	29%	N.A.	N.A.	236 μA cm^–2^	N.A.	N.A.	^93^
*Moorella thermoacetica*	CdS NPs	CO_2_ fixation	N.A.	2.50%	N.A.	120	acetic acid (1.25 mM over 60 h)	N.A.	N.A.	^211^
*M. thermoacetica*	Au nanoclusters	CO_2_ fixation	N.A.	Up to 3%	N.A.	96	acetic acid (4.5 mM over 4 days, per 1 g cells)	N.A.	N.A.	^212^
*Sporomusa ovata*	SrTiO_3_:La,Rh, BiVO_4_:Mo, ITO	CO_2_ fixation, OER	N.A.	21.30%	88.20%	15 per cycle (three cycles recorded)	acetate (9 mM over 15 h)	N.A.	N.A.	^229^
*R. palustris*	Zinc–nickel based catalyst (ZGGO:Ni)	CO_2_ fixation, lycopene biosynthesis	N.A.	N.A.	N.A.	480 (CO_2_ fixation)	Lycopene (8.80 mg L^–1^)	N.A.	N.A.	^381^
*R. palustris, Synechocystis PCC 6803*	Zinc based catalyst (ZGGO), Zinc–nickel based catalyst (ZGGO:Ni)	CO_2_ fixation, HER, NADPH regeneration, lycopene biosynthesis	N.A.	N.A.	N.A.	96	H_2_ (1.38 mmol h–1 g–1), lycopene (8.80 mg L^–1^)	N.A.	N.A.	^384^
*Clostridium thermocellum*	BiVO_4_/CoP photoanode	Cellulose degradation, glucose\cellobiose oxidation	0	60% (before cocatalyst deposition)	40.60%	76	H_2_ (33 μmol over 2h), >400 μA cm^–2^ (over 72 h)	N.A.	N.A.	^308^
*E. coli*, recombinant (SP-1 cadmium binding peptide)	CdS QDs, methyl viologen	NADPH regeneration, Stereospecific imine reduction	N.A.	N.A.	N.A.	9	(R)-MPN (∼8 mM over 9 h)	N.A.	99.2% (R)-MPN	^209^
*E. coli*, recombinant	CdS/NiO	NADPH regeneration, Stereospecific imine reduction	bias free	N.A.	85%, 51% (bias-free setup)	14	(R)-MPN (≤10 mM after 14 h)	N.A.	99.5% (R)-MPN	^312^
*E. coli*, recombinant	Cd QDs, Rhodium dye	NADH regeneration, CO_2_ fixation	N.A.	N.A.	N.A.	36	formate (up to 14.2 mM)	N.A.	N.A.	^377^
*Saccharomyces cerevisiae*	InP (polyphenol functionalized)	NADH regeneration, shikimic acid production	N.A.	1.58%	N.A.	72	shikimic acid (∼45 mg L^–1^)	N.A.	N.A.	^380^
*Ralstonia eutropha*	Eosin Y	NADH regeneration, CO_2_ fixation, acetoin production	N.A.	2.60%	N.A.	50	acetoin (1.41 mM over 25 h)	N.A.	N.A.	^382^
*Chlorella vulgaris*	Osmium redox polymer	Power generation, ORR	0.35	7.4 × 10^–4^	N.A.	N.A.	N.A.	1.5 μW cm^–2^	N.A.	^271^
*E. coli*, recombinant	Bioproduced phenazine, Carbon paper electrode	power generation, EET	–0.1 to 0.7 (SWV)	N.A.	N.A.	300	phenazines (>200 μM after 30 h), 200 μA cm^–2^	80.6 μW cm^–2^	N.A.	^275^
*Nostoc sphaeroides*	Indium phosphide NPs	Ethylene production	N.A.	40%	N.A.	120	ethylene (4.4 μmol per gr cells per day)	N.A.	N.A.	^201^

## 6 (Photo)bioelectrochemical Cells for Fine Chemical or Fuel Generation

### 6.1 Photodriven Water Oxidation Configurations

As discussed, PSII has a critical role as the engine of life. The light-induced water oxidation reaction provides the essential step to convert inorganic materials into chemical energy and biomass. Emulating this process using photobioelectrochemistry can be achieved by advanced material design and a well-interfaced biotic-abiotic configuration. During the past decades, two different approaches were mainly developed to allow water oxidation and fuel generation (H_2_ generation, CO_2_ reduction to fuels):

A. Using bioanodes, mainly PSII isolated proteins, thylakoid membranes, leaves, or cyanobacteria cells as photocatalysts on an electrode support.
B. Inorganic catalysts (semiconductors, homogeneous catalysts) were used to drive the water oxidation reaction.

The utilization of isolated PSII proteins for water oxidation has been discussed in Section 3.2.2.

As shown, the photocurrents and stability of such devices are limited and lag far behind those of alternatives. Recently, it has been demonstrated that the cyanobacterium *Synechocystis sp. PCC 6803* can be coupled to ITO electrodes to enable photocurrents of 245 μA/cm^2^ with 29% efficiency.^93^ These outstanding high photocurrents were achieved through state-of-the-art electrode surface design, in which micropillar ITO structures were printed onto an FTO-coated glass. While this is a significant advance and presents top performance, the designed configuration requires diffusional redox mediators and an applied bias of 0.5 V for its activation. By excluding the redox mediators, the system can still operate, however, with significantly lower photocurrents. Alternatively, semiconductor-based photoanodes, e.g., BiVO_4_/MO_*x*_, can achieve such performance using a 1 V lower applied potential!

Therefore, with the current advances, utilization of the biotic process at the anode is less favorable. On the other hand, CO_2_, N_2_, H^+^, and O_2_ reduction processes may be facilitated at the cathode, activated by the photoanode’s abiotic photoinduced reaction. In the next section, which focuses on waste degradation for energy generation, a holistic approach that uses both biotic and abiotic catalysts at the anode is presented and discussed.

A decade ago, Choi and co-workers presented an elegant, straightforward methodology for electrodepositing porous BiVO_4_ layers on conductive surfaces.^230,231^ The outstanding stability and efficiency of these fabricated photoanodes paved the way for their use in water oxidation devices, mainly PECs. As part of these scientific efforts, it was shown that these photoanodes can be coupled with biological processes. BiVO_4_ photoanodes can reach up to 90% incident photon-to-current efficiency,^232^ and the developed electrodeposition protocol can be easily scaled up.^233,234^

Toward realizing a biotic-abiotic photobioelectrochemical cell, Mukha and co-workers presented a design comprising a BiVO_4_ photoanode coupled with a biocathode.^235^ The BiVO_4_ photoanode was coupled with a biocathode based on bilirubin oxidase (BOD) as an oxygen reduction biocatalyst, Figure 24. The BiVO_4_ photoanode was used for water oxidation to generate the oxygen. To improve photoanode performance, water oxidation was further enhanced by electrodeposition of a nickel–iron (FeOOH/NiOOH) or cobalt phosphate (CoP) layer as a cocatalyst. Although the nickel–iron layer was more robust at high potentials, the CoP layer produced higher photocurrents at lower voltages, down to 0 V (vs Ag/AgCl). The mesoporous structure of the BiVO_4_-CoP results in a high surface area, leading to high photocurrent generation.^230^

**Figure 24:**
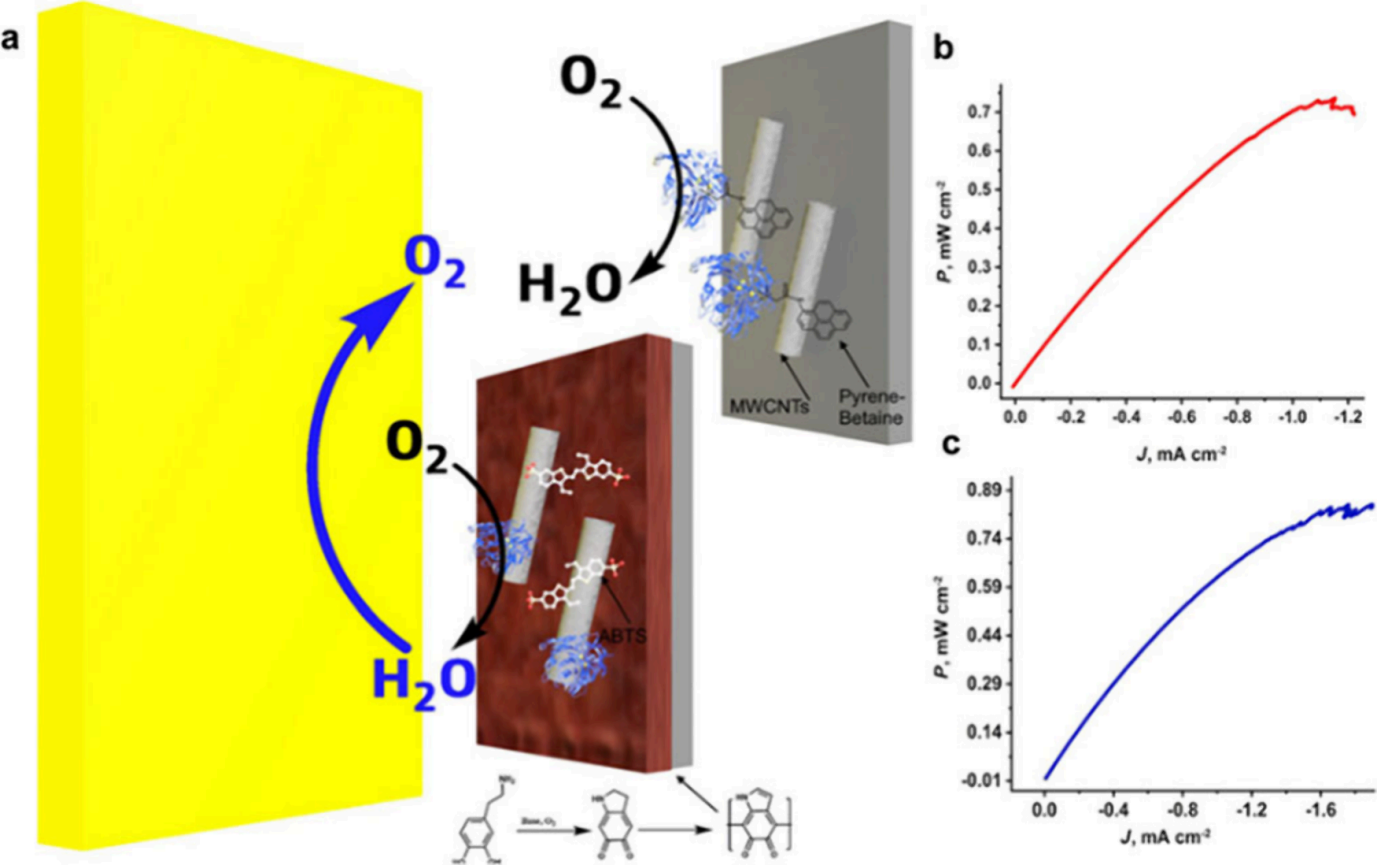
Highlights of a BOD-based PBEC. (a) Water oxidation by the BiVO_4_–CoP photoanode under irradiation (in yellow). In parallel, the BOD-based biocathode reduces molecular oxygen into water. The BOD biocathode utilized either direct or mediated ET (gray and brown-gray cathode, respectively). The power curves for direct and mediated ET PBEC are shown (b+c, respectively). Adapted with permission from ref 235. Copyright 2020 Wiley-VCH GmbH.

Under light irradiation and applied potential above −0.5 V vs Ag/AgCl, anodic photocurrent could be obtained. Therefore, to construct a bias-free photobioelectrochemical cell, a cathodic photocurrent should be reached while an applied potential is positive than −0.5 V. In the presented work, two different biocathodes were developed, based on either mediated or direct electron transfer process (MET and DET, respectively). Although both configurations generated bioelectrocatalytic currents of up to 3 mA/cm^2^ under oxygen-saturated conditions, the mediated approach using MvBOD and the ABTS redox mediator entrapped in a polydopamine layer displayed superior stability and lower onset potentials than DET configurations. Entrapping redox-active molecules or binding them to polymeric chains via enzymes has been proven successful in many bioelectrochemical systems, e.g., for sensing^236,237^ or hydrogen generation.^238–240^ By coupling the DET and MET biocathodes with the BiVO_4_ photoanode, power outputs of ∼ 0.8 and 0.7 mW/cm^2^ were observed under oxygen saturation, Figure 24b, 24c, respectively. These high-power outputs were dictated by the high photocurrents, bioelectrocatalytic currents, and open-circuit voltages (OCVs) of 1 V. Overall, the developed biotic-abiotic configuration demonstrates a bias-free O_2_/H_2_O recycling system that produces high-power outputs at low fabrication and materials cost.

Converting light energy into electrical power in a bias-free configuration is of great importance. Nevertheless, it requires an electrical grid or energy-storing devices for practical use in the future. Alternatively, water-oxidation reactions can be coupled to biocathodes to generate fuels. BiVO_4_-based photoanodes fall short in terms of developed potential to generate H_2_ without biasing the electrodes. Therefore, an additional photocatalyst is required for such a task. As learned from nature, the energy levels of the photosensitizers should be aligned to enable at least −0.41 and 0.82 V vs NHE at pH 7 to catalyze H_2_ generation or water oxidation, respectively. The above potential usually requires overpotentials for an effective catalytic process. Therefore, a few hundred mV should be added to the thermodynamic potential, depending on the catalysts and the system configuration. During the last decades, highly efficient photocathodes have been developed. However, often, their stability in aqueous solutions was limited. Moore and co-workers have developed a tandem system that couples the perovskite’s photo absorber with the BiVO_4_-based photooxidation catalyst.^241^ The biotic-abiotic configuration comprises a charge-separation layer that limits back-reactions and recombination processes, Figure 25. While perovskites have outstanding light-absorption efficiencies, they degrade rapidly at high-humidity conditions or in aqueous solutions. Therefore, protective layers were added to limit any contact between the perovskite layer and the solution. To enable efficient hydrogen generation, a hydrogenase originating from *Desulfovibrio vulgaris* Hildenborough was deposited as the biocatalyst. The *Desulfovibrio vulgaris* Hildenborough was selected due to its improved stability and activity in the presence of oxygen. The Z-scheme was designed to generate the required potential to enable bias-free activation of the biotic-abiotic photobioelectrochemical cell, with solar-to-hydrogen efficiency (STH) of 1.1%. Due to hydrogenase’s stability under oxygen, the system did not require a separation membrane for operation.

**Figure 25:**
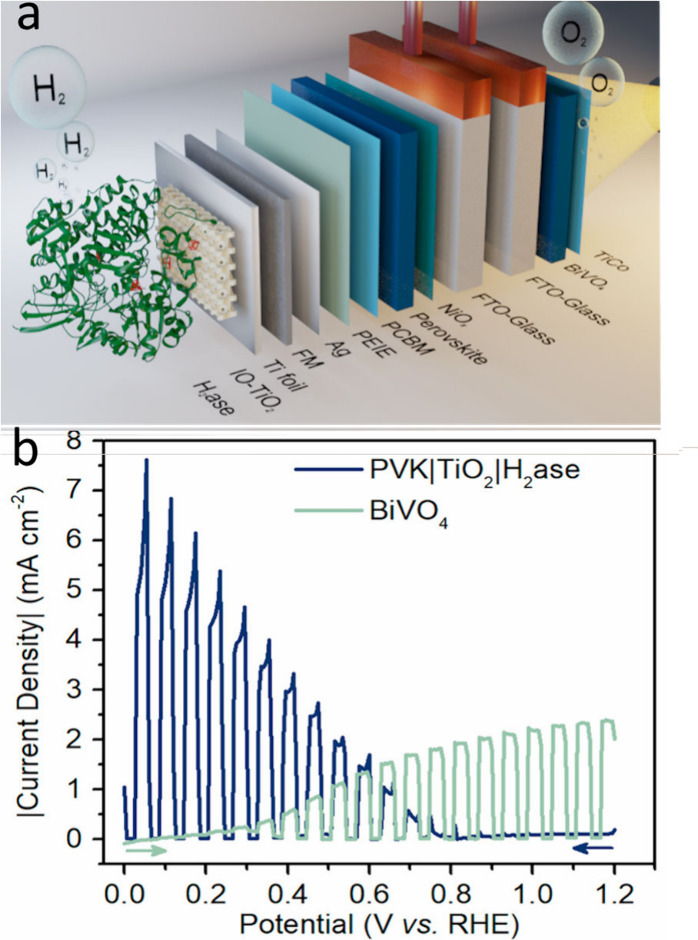
(a) Schematic representation of the tandem PEC cell consisting of a FM-encapsulated perovskite photocathode with H_2_ase integrated into an IO-TiO_2_ layer and a BiVO_4_ photoanode. TiCo refers to the water oxidation layer precursor: [Ti_4_O­(OEt)_15_(CoCl)]. PCBM: [6,6]-phenyl-C_61_-butyric acid methyl ester. PEIE: polyethylenimine. (b) Representative LSV of PVK|TiO_2_|H_2_ase (blue) and BiVO_4_ (green) electrodes with chopped illumination, forward scan, 10 mV s^–1^ scan rate, showing the absolute current densities. Adapted from ref 241. Copyright 2019 American Chemical Society.

Furthermore, the system enables activation via single-directional light irradiation, in which BiVO_4_ absorbs at shorter wavelengths (below 500 nm) and the cathode is harnessed above 500 nm. It should be noted that covering the entire visible light spectrum surpasses the natural photosynthetic systems. While the designed system demonstrated significant advances toward an abiotic water-splitting device, its stability was limited. Furthermore, to scale up the system, a separation between oxygen and hydrogen should be implemented. Cheng and co-workers developed a similar approach.^239^ BiVO_4_-based photoanode was utilized to facilitate photodriven water oxidation, while a PB6 dye was used at the cathode to supply the required potential for bias-free water splitting. As presented in Figure 26, to enable efficient activation and stabilization of the [FeFe]-H2ase from *Chlamydomonas reinhardtii*, a 2,2′-viologen-based redox polymer was mixed with the enzymes. Under light irradiation, the developed biotic-abiotic configuration reached a faradaic efficiency of 81% and STH of 0.124%. These results gained by biotic-abiotic water splitting devices are aligned with or even surpass similar abiotic configurations. The water-splitting process indeed follows the principles of natural photosynthesis, converting water and sunlight into fuels (H_2_).

**Figure 26:**
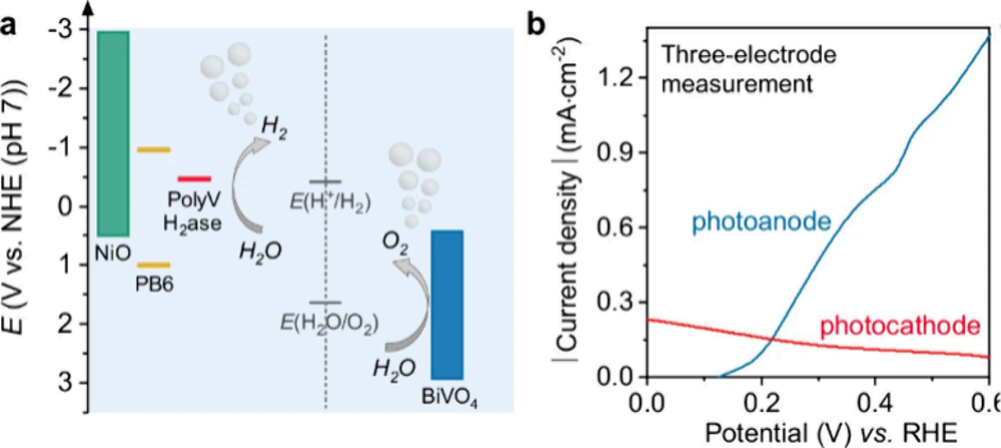
Performance of the overall water splitting system. A scheme of the device for overall water spitting. b LSV scans of NiO|PB6|PolyV|H_2_ase (photocathode) and modified BiVO_4_ (photoanode) under illumination, obtained from three-electrode measurements. The photocurrent density of the photocathode was inverted for comparison. Adapted from ref 239. Available under a CC BY 4.0 license. Copyright 2024 The Author(s).

Nevertheless, coupling the CO_2_ reduction process with water oxidation has several advantages:

a. It captures CO_2_ and helps to limit elevated atmospheric concentrations.
b. Liquid fuels, e.g., formic acid and methanol, produced from the CO_2_ reduction processes, can be stored and transported directly. Liquid fuels can also be used in industrial processes and in standard fuel engines.
c. The resulting CO_2_ reduction products can undergo further reduction steps as part of an enzymatic cascade.

Photoelectrochemical, electrochemical, and bioelectrochemical configurations for CO_2_ reduction have been developed over the last few decades. Holistic approaches were also developed. A flow electrolysis and bioreactor for microbial fermentation were coupled to enable CO_2_ conversion into long-chain fatty acids production.^242^ The developed system provides high currents and high conversion efficiency of CO_2_ into CO (100%), which is further converted into acetic acid that could be used for fermentation. However, those systems were commonly dependent on sacrificial electron donors, high temperatures, or applied bias for activation. Keun Kuk *et al.* presented a photobioelectrochemical cell that enables the conversion of CO_2_ and water into O_2_ and methanol fuel. The developed system coupled Co-Pi/α-Fe_2_O_3_|BiFeO_3_ with CpRh­(bpy)­(H_2_O)]^2+^ to enable the regeneration of NAD into NADH. The cofactor regeneration was, in turn, utilized for the activation of a sequential biocatalytic reduction process, enabling the conversion of CO_2_ into formic acid, formaldehyde, and methanol, a 6-electron reduction process.^243^ The configuration could operate without external bias, reaching 3% spontaneous regeneration of NADH. The NADH regeneration was further enhanced using a photobioelectrochemical cell constructed with BiVO_4_/NiOOH photoanode and perovskite cathode, enabling a dual absorbance tandem cell. The cell was conjugated to CpRh­(bpy)­(H_2_O)]^2+^ NADH regeneration system, which facilitated the bioelectrocatalytic conversion of ketoglutamate to l-glutamate.^244^

Just recently, See Yeung and co-workers presented a holistic configuration for the photoelectrochemical conversion of H_2_O + CO_2_ into formic acid fuel, Figure 27.^245^ Toward that, the BiVO_4_ photoanode was coupled with a PEDOT/PSS organic photosensitizer-based photocathode. The PEDOT/PSS photocathode was constructed with added charge-separation layers and a TiO_2_ high-surface-area mesoporous layer. The developed layered configuration was integrated with the formate dehydrogenase enzyme, which served as the biocatalyst for converting CO_2_ to formate. Naturally, the cyanobacterial photosynthetic process utilizes carboxysomes, microcompartments, to concentrate CO_2_ levels and limit the adverse effects of oxygen on CO_2_ assimilation. To mimic this process and increase the availability of CO_2_/bicarbonate in proximity to the FDH enzyme, carbonic anhydrase was codeposited on the cathode, next to the FDH. The developed cell demonstrates an architecture that enables water oxidation, a charge separation system, and CO_2_ reduction into liquid fuel in a bias-free biotic-abiotic configuration. Formic acid could be used as a H_2_ carrier or directly as a fuel.

**Figure 27:**
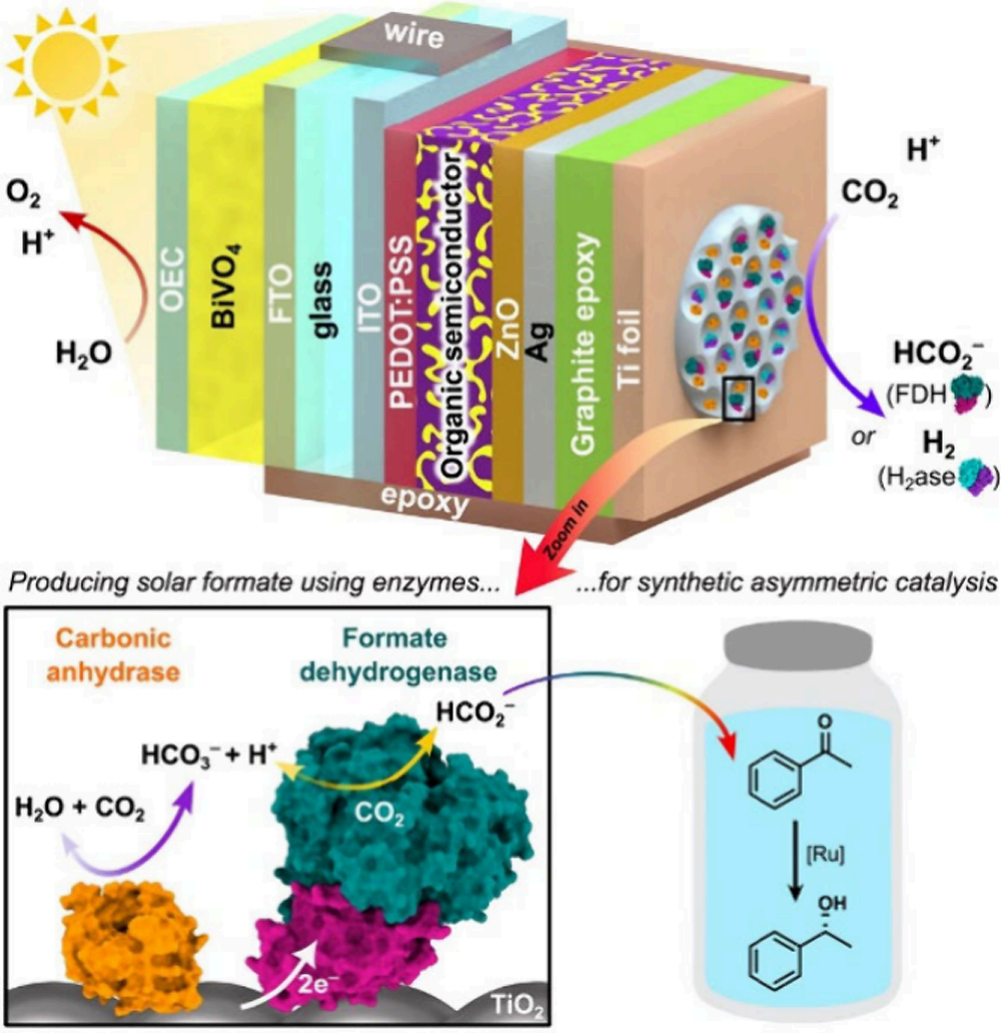
Biohybrid device incorporating organic semiconductors and enzymes for the photoelectrochemical conversion of H_2_O + CO_2_ into formic acid fuel. Reproduced from ref 245. Available under a CC BY 4.0 license. Copyright 2025 The Author(s).

Nevertheless, using it to produce pure chiral precursors for pharmaceuticals and other essential industries has greater economic and sustainability value. Therefore, the work demonstrated a proof-of-concept integration of the photobioelectrochemical cell with an asymmetric catalyst. As shown in Figure 28, the produced formate was used as a reductant to convert acetophenone into the chiral aromatic alcohol (R)-1-phenylethanol, with 78% yield and 94% enantiomeric selectivity.

**Figure 28:**
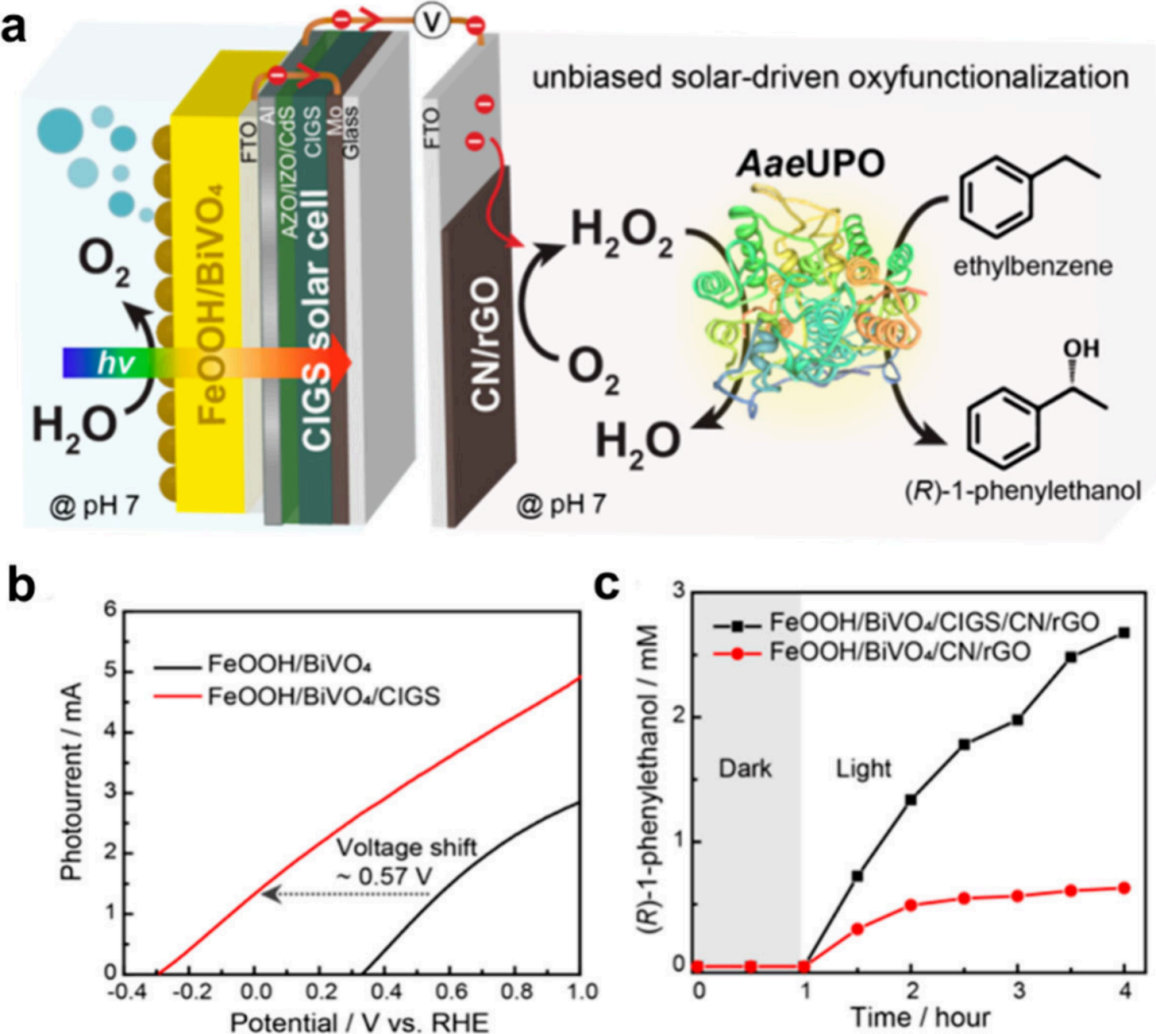
A light focusing PBEC for CO_2_ reduction. (a) Schematic representation of the system. The FeOOH/BiVO_4_ photoanode (yellow) and the CIGS solar cell (green) both absorb solar energy to generate an electric current from water oxidation. Then, the current is utilized by the CN/rGO cathode to reduce oxygen to hydrogen peroxide. The AaeUPO enzyme further reduces the peroxide while oxidizing ethylbenzene in a chiral manner. (b) Overpotential reduction by the CIGS solar absorber. Photocurrent is shown before (black) and after (red) addition of the CIGS solar cell. (c) Ethylbenzene conversion over time with (black) and without (red) the CIGS solar cell. Adapted from ref 246. Copyright 2019 American Chemical Society.

Harnessing solar radiation to drive chemical reactions is advantageous from an economic and environmental standpoint. In a paper by Choi *et al.*, light was utilized to synthesize (R)-1-phenylethanol from ethylbenzene using a PBEC at ambient conditions and without applied bias.^246^ The PBEC’s anodic component was assembled from a FeOOH/BiVO_4_ photoanode and a copper solar absorber (CIGS). While the FeOOH/BiVO_4_ photoanode absorbed light for water oxidation, the CIGS absorbed the remained irradiation. Absorbing the unabsorbed irradiation increased the thermodynamic driving force and reduced overpotential by 0.57 V. However, CIGS contains expensive elements such as indium and gallium, which limit its commercial potential. Still, alternative solar absorbers could offer greater economic feasibility for commercialization.^247,248^ To complement the BiVO–CIGS photoanode, the abiotic cathode contained a layer of carbon nitride and reduced graphene oxide (CN/rGO) on FTO for oxygen reduction. Additionally, the CN/rGO ratio was optimized to allow maximal faradaic efficiency of 92%. On the cathode’s surface, CN/rGO reduced O_2_ to H_2_O_2_, which in turn activates the AaeUPO peroxygenase enzyme. AaeUPO catalyzes stereospecific hydroxylation to produce ethylbenzene to (R)-1-phenylethanol. Furthermore, several structural analogs of ethylbenzene were also shown to undergo hydroxylation with either lower turnover or lower stereospecificity. (R)-1-phenylethanol is a key component for polishes, washing agents, and air fresheners, as well as a building block for pharmaceutical drugs. Using the full system, ethylbenzene was converted to (R)-1-phenylethanol with a total conversion rate of ∼ 2.5% over 3 h and a turnover rate of 12.9 s^–1^. The system operated at neutral pH, ambient temperature, and atmospheric conditions. The ethylbenzene conversion rate of ∼ 0.9 mM h^–1^ correlated with a previously reported system.^249^ Taken together, this work demonstrates the potential of combining specific enzymatic reactions with well-known electrochemical processes.

### 6.2 Enzymes or Bacterial Fuel Cells toward Electrical Energy, Fuels, or Ammonia Generation

One of the most critical chemical processes today is the Haber-Bosch reaction for ammonia generation. This abiotic reaction requires extreme conditions and an extensive energy input. Therefore, alternative methods to enable ammonia generation under more ambient conditions should be pursued. Coupling the natural nitrogen fixation process with electrodes or semiconductors has been at the center of extensive research over the past decade.^191,192,194,195,250–256^ Milton and co-workers have presented an elegant approach to construct a Haber–Bosch-like system.^257^ The work-coupled H_2_ oxidation anode was facilitated by a hydrogenase enzyme and a nitrogenase cascade-based cathode for the generation of ammonia. In both the anode and cathode, methyl viologen was used as a redox mediator to enable electron transfer. The system provides a route for utilizing enzymatic reactions to convert H_2_ and N_2_ into ammonia under ambient conditions, similar to the Haber-Bosch process. However, this process depends on H_2_ for operation; hence, it requires an additional activation step.

A recent work by Meirovich and co-workers has suggested a different path for activation. Nitrogen was chemically reduced into ammonia using a biotic-abiotic PEC-based configuration, Figure 29a.^192^ To mitigate the energy consumption used in the industrial nitrogen reduction process, the nitrogenase (MoFe-P) enzyme was used. MoFe-P, the Fe-P auxiliary protein, the (SPr)_2_V redox mediator, and carbon electrodes were all coupled to form a biocathode. To artificially activate MoFe-P and enable nitrogen reduction, semiconductor photoanodes of two types were examined: (i) BiVO_4_/CoP and (ii) CdS/NiO. Both photoanodes absorb in the visible range and can suppress the water oxidation reaction. The suppression effect negated side reactions and enabled the specific oxidation of glucose and ascorbic acid, respectively. BiVO_4_ can oxidize water into oxygen, which can inactivate the nitrogenase enzyme. Thus, suppressing the water oxidation is required. However, alternatively, oxidizing glucose is critical for a membrane-less configuration. Both photoanode and biocathode were then integrated to form a PBEC for ammonia production under minimal to no bias at ambient aqueous conditions. Although the BiVO_4_ photoanode generated a higher anodic current, ammonia production was higher using the CdS/NiO photoanode: ca. 55 μmol compared to 100 μmol, respectively, Figure 29b and Figure 29c. As CdS/NiO’s conductive band is higher than that of BiVO_4_, it enabled higher photocurrent at lower potentials, leading to higher ammonia generation. Although the system’s stability is limited by several components (ATP and enzymes), it succeeded in maintaining ambient conditions to produce ammonia without the need for expensive catalysts. The amounts produced are, of course, not yet practical for real-world applications. However, further development may lead to better yields and improved design.

**Figure 29:**
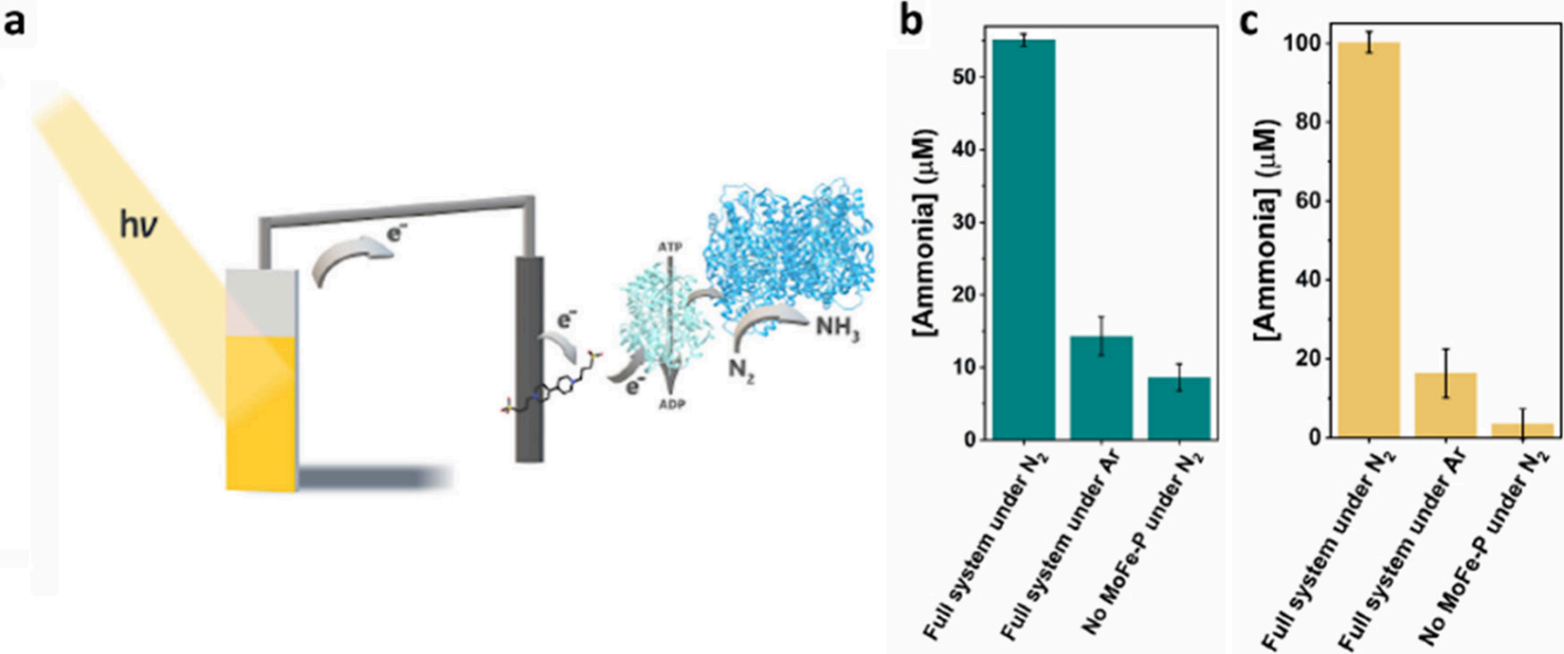
A nitrogen-reducing, light-assisted PBEC. (a) Schematic representation of the system, where the semiconductor photoanode is coupled with a graphite rod. The rod reduces the soluble (SPr)_2_V redox mediator to enable electron transfer. The electrons are first transferred to the auxiliary protein Fe-P, then to MoFe-P for nitrogen reduction. Using ATP, Fe-P both changes the conformation of MoFe-P and supplies electrons for nitrogen reduction. Ammonia generation without applied bias can be seen for two types of photoanodes – BiVO_4_-CoP (b) and CdS-NiOx (c). Adapted with permission from ref 192. Copyright 2024 Elsevier.

While photoelectrochemistry can facilitate the synthesis of valuable chemicals, it can also break down materials. This property is advantageous for the treatment of the chemical pollutants. These remains a consistent issue to this day, with materials such as pesticides, oil, organic solvents, cyanide, strong acids and bases, various sludges, and so on. A recent paper by Ding and co-workers described the breakdown of phenols in wastewater using a PBEC, Figure 30.^258^ The system comprised a biotic-abiotic configuration consisting of a biophotoanode and a cobalt catalyst buckypaper or an HRP cathode. The biophotoanode was fabricated using TiO_2_ NPs, and a dye photosensitizer, SA-TCP.P A NAD-dependent glucose dehydrogenase (NAD-GDH), was used to regenerate NADH. In the presence of NADH and visible light, a photocurrent was observed where NADH acted as the electron donor. To ensure continuous regeneration of the NADH cofactor, the NAD-GDH enzyme oxidizes soluble glucose and reduces NAD^+^ to NADH. The induced photocurrent was utilized by the cobalt catalyst (CoNOC) to drive the two-electron oxygen reduction reaction (ORR) and generate hydrogen peroxide. The observed OOR remained stable for 5h while generating up to 1 mA/cm^2^ under oxygen-saturated conditions. The generated H_2_O_2_ was used to activate the HRP enzyme, which, in turn, facilitated the degradation of phenols. The presented systems show a holistic approach for wastewater treatment and electrical energy generation. Coupling energy generation to an adjunct beneficial process is a key direction for developing new energy processes with economic viability.

**Figure 30:**
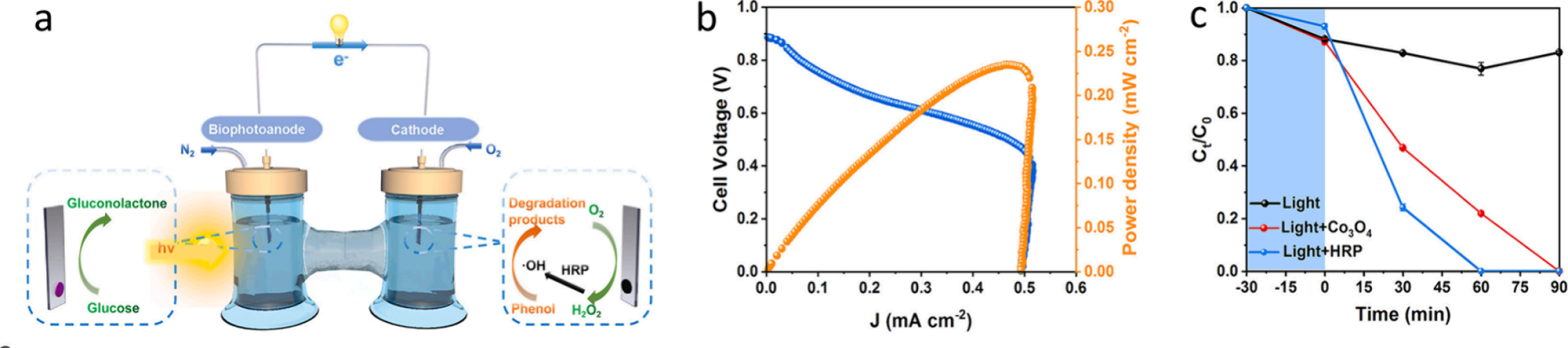
PBEC for wastewater treatment employing either HRP or a cobalt oxide nanozyme. (a) Schematic representation of the system. (b) Power curve of the PBEC in real wastewater. The voltage to current and power to current curves are presented (blue and orange, respectively). (c) Cathodic phenol degradation without a catalyst, with HRP, and with the cobalt oxide nanozyme (black, blue, and red curves, respectively). The blue area represents a time phase without illumination. Adapted with permission from ref 258. Copyright 2024 Elsevier.

One of the growing fields in energy storage is renewable fuel. As fossil fuels are finite and polluting, research has focused on developing alternatives, such as renewable fuels. For example, dozens of billions of liters of bioethanol are produced each year.^259^ However, ethanol’s energy density is relatively low, making it insufficient for some applications. Therefore, energy-rich renewable fuels are required to supplement or even replace fossil fuels. In an article by Harris *et al*., an abiotic PEC was integrated with enzymes to generate renewable fuel.^260^ The PEC was composed of abiotic electrodes and a solution containing two biocatalysts - alcohol oxidase (AOx) and beta-alanine. The BiVO_4_ photoanode oxidizes water, generating electrons. The PEC generated power by coupling a BiVO_4_ photoanode to a platinum cathode, Figure 31a. The generated oxygen served as an electron acceptor for AOx, which oxidized butanol to butanal. Then, pairs of butanal molecules underwent aldol condensation to form 2-ethylhexanal (2-EH). The organocatalyst beta-alanine catalyzed the condensation to form the nonvolatile ethylhexanal. While the observed butanol to butanal total conversion rate was 2.6%, the butanal to 2-EH conversion rate was significantly higher. Furthermore, removing the light from the system significantly reduced both butanal and 2-EH amounts. The authors suggested that AOx may have an unknown inhibition mechanism, even when beta-alanine pushes the reaction forward.

**Figure 31:**
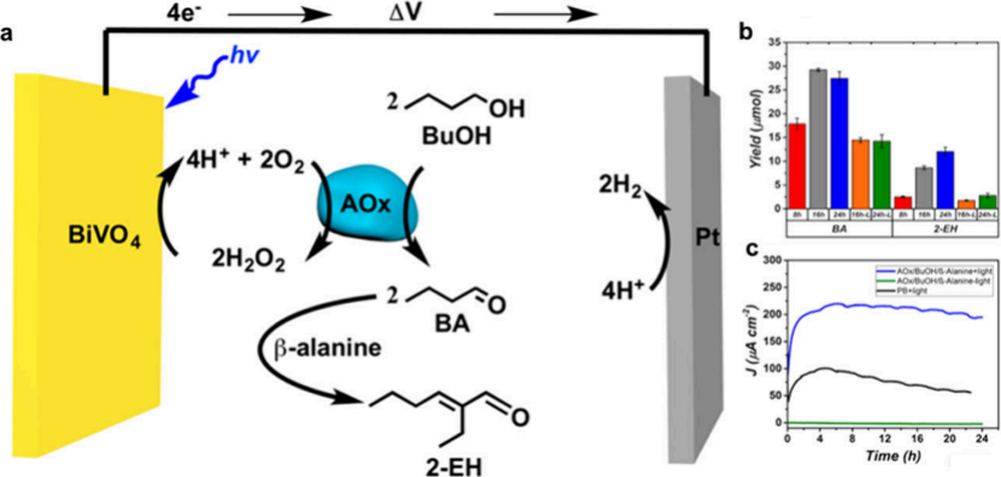
A PBEC that generates jet fuel from simple alcohols. (a) Schematic representation of the system. The BiVO_4_ photoanode oxidizes water, enriching the solution with oxygen and protons. Alcohol oxidase (AOx) reduces oxygen to hydrogen peroxide while oxidizing butanol to butanal (BA). Then, BA undergoes aldol condensation to form 2-EH. Simultaneously, the Pt cathode reduces the excess protons to hydrogen gas. (b) Yields of butanal and 2-EH with or without light at specific time points. (c) The effect of AOx presence on water oxidation at 0 V vs Ag/AgCl. According to the Le Chatelier principle, AOx reduces oxygen and pushes the water oxidation reaction forward. The improved water oxidation rate translates to increased current in AOx-containing cells (blue) vs the control cells (black) upon irradiation. Adapted from ref 260. Copyright 2017 American Chemical Society.

## 7 Electrochemical Photoreforming of Waste into Electrical Energy, Fuels, or Value-Added Chemicals

### 7.1 (Photo)­bioelectrochemical Cells for the Conversion of Plastic or Cellulose into Electrical Energy, Fuels, or Fine Chemicals

As agriculture expands and develops, various types of carbon-rich waste tend to accumulate (e.g., weeds, cutoff branches, peels, etc.). Biomass waste contains energy-rich polysaccharides, such as cellulose, the most abundant polymer on earth. Valorization of cellulose or lignin, a highly abundant material, could prove to be beneficial from environmental and economic perspectives.

Naturally, biomass undergoes enzymatic degradation of its biopolymers. Cellulose-degrading bacteria secrete an enzymatic complex, the cellulosome, which degrades the β-d-glucose polymer into cellobiose, a soluble sugar molecule that most organisms cannot use as a carbon or energy source. The bacterium breaks the cellobiose into glucose monomers using an internally expressed β-deglycosylase after its intake. This strategy limits the bacteria’s competition for critical resources. Developing methodologies to utilize cellulose waste directly could be highly beneficial and should therefore be pursued.

Fossil fuels are a great energy source that is easy to use and transport. Nevertheless, millions of years are required for the conversion of biomass into fossil fuels through natural processes. Over the last few centuries, humankind has burned biomass to generate heat, chemical energy, and electricity. However, these methodologies are harmful to the environment and have a limited efficiency.

Utilizing photochemical,^261,262^ (photo)­electrochemical,^263,264^ biochemical,^265–267^ and (photo)­bioelectrochemical configurations,^268–277^ for direct conversion of biomass into electricity or chemical fuel could be beneficial.^278–281^ However, several obstacles need to be addressed toward realization:

a. Polysaccharide polymers have poor solubility in aqueous solutions.
b. Cellulose is highly crystalline and densely packed, hindering its degradation by using (bio)­catalysts.
c. Harsh conditions are required for its chemical degradation.

Therefore, to exploit polysaccharides for the generation of fuels or electrical energy using (photo)­electrochemical tools, one should design a multidisciplinary approach that enables degradation and efficient oxidation.^282–284^ Such processes, in a way, will rewind the classical photosynthetic process and use biomass in reverse to generate energy. Ideally, to overcome activation barriers, these processes should occur spontaneously or be derived from a sustainable source, such as sunlight. A TiO_2_-based PEC was introduced for the degradation of cellulose. The cellulose degradation is facilitated by OH radicals formed by the photooxidation of hydroxy ions.^285^ While the system indeed facilitated the photooxidation of the deposited polymer, developed 1.1 V of photovoltage and a quantum efficiency up to 52%, strong basic pH and limited visible light absorbance hindered the presented configuration.

Furthermore, the system photo-oxidized only the deposited cellulose matrix and could not reach the nonsoluble material in the electrochemical cell. An improved PEC configuration was developed,^286^ in which the TiO_2_ layer was modified with CuBi_2_O_4_. The latter significantly suppressed the water oxidation reaction and improved the Faradaic efficiency under basic conditions. Suppressing water oxidation during organic matter oxidation is a key feature that enables an improved efficiency and reduced side reactions.

Alternatively, Shemesh and co-workers presented a biotic-abiotic PEC that utilizes cellulose waste to generate electrical power.^287^ Here, the cellulose waste was enzymatically digested by a cellulase complex added to the cell. The complex consists of 3 hydrolytic enzymes that enable the degradation of the β-d-glycosidic bonds into small glucose repeats at neutral pH. These were subsequently photooxidized by the N-type BiVO_4_/CoP photoanode, Figure 32a. The addition of a thick cobalt phosphate layer dictated a valence band position shift, leading to a full suppression of the water oxidation reaction, Figure 32b. Interestingly, a thin CoP layer commonly dictates an enhanced water oxidation reaction; however, tuning the CoP layer’s thickness and morphology enables suppression. Hence, the BiVO_4_ photoanode favors oxidation of both glucose and cellobiose and fully suppresses the water oxidation. This phenomenon was attributed to band bending, which led to a higher valence-band position at the CoP interface. The shift dictates a new energy level above the one that thermodynamically enables water oxidation. The oxidation products were detected by NMR and identified as small organic molecules useful for other processes, e.g., acetic acid and formic acid.

**Figure 32:**
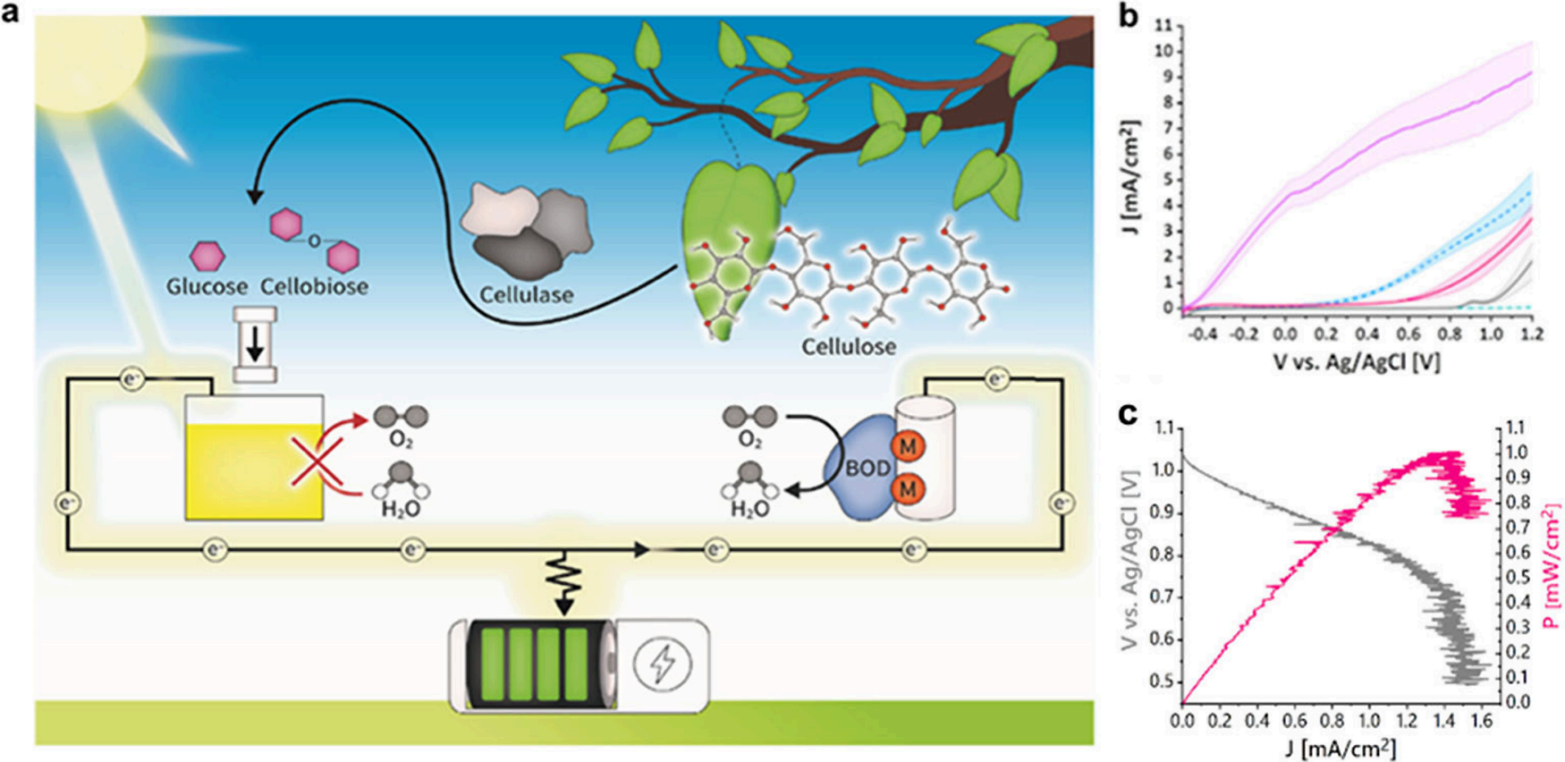
PBEC for the valorization of cellulose-rich waste. (a) a schematic of the cellulose-degrading PBEC. Cellulose waste is placed in the cell, where a cellulase enzyme complex degrades it into glucose and cellobiose. The BiVO_4_-CoP photoanode further oxidizes the degradation products while suppressing water oxidation. Then, the BOD-based biocathode utilizes the current to reduce oxygen, thus generating power. (b) Effect of the CoP water oxidation suppressing layer. The photocurrent of pristine BiVO_4_ before and after illumination is shown (gray and light blue curves, respectively). Additionally, the photocurrents of BiVO_4_ with thin and thick CoP layers are shown (Purple and pink curves, respectively). The colored areas around the curve denote the error for each measurement type. (c) Power curve of the full PBEC with the addition of cellulose. The gray curve describes potential to current, while the pink curve describes power to current. Adapted with permission from ref 287. Copyright 2023 Elsevier.

Furthermore, the remarkable stability of BiVO_4_/CoP enabled cellulose-derived photocurrents of ca. 0.6 mA/cm^2^ for at least 5 days. The BiVO_4_/CoP photoanode was then coupled with a bilirubin oxidase (BOD) biocathode to form a bias-free two-electrode photobioelectrochemical cell. The BOD biocathode was coupled with the BiVO_4_ photoanode to produce electrical power by facilitating the bioelectrocatalytic oxygen reduction, Figure 32c. By coupling the photoanode with the BOD-based cathode, an OCV of 1 V was reached, which in turn yielded up to ∼ 1 mW/cm^2^ power output (at O_2_ saturation). The wide OCV stems in part from the redox potential of BOD (0.6 V vs Ag/AgCl), which is 200 mV higher than that of the expensive Pt cathodes at pH 7.^288^ The PBEC displayed a faradaic efficiency of 48.7%, which is lower than the BiVO_4_/CoP photoanodes designed for water splitting.^289–291^

Poly terephthalate (PET) is a widely used plastic, commonly used in packaging, textiles, and films. However, spontaneous PET degradation occurs very slowly, which is insufficient, as 350 tons of waste are generated each year. Moreover, active abiotic PET degradation generally requires harsh conditions, which are therefore less attractive.^292,293^ Taking the environmental issue into account, biodegradable polymers such as polyethylene furan dicarboxylate (PEF) have been garnering increasing interest. To synthesize mass quantities of PEF, large amounts of the monomer 2,5-furandicarboxylic acid (FDCA) must be obtained. Therefore, a robust and sustainable method for FDCA synthesis is crucial for PEF production. In a recent work by Zhang and co-workers, FDCA was synthesized from 5-hydroxymethylfurfural using a NiFe/BiVO_4_ photoanode and a BOD-based biocathode, Figure 33.^294^ To improve both photocurrents and onset potential, pristine BiVO_4_ was modified with NiFe using a hydrothermal methodology. The NiFe modification lowered the onset potential and increased photocurrent output by improving the photoanode’s charge separation. On the photoanode’s surface, the NiFe cocatalyst oxidizes TEMPO, which oxidizes HMF in turn. TEMPO is regenerated by the constant photocurrent, enabling high conversion rates. The biocathode was formed on an ITO substrate coated with porphyrin-modified (PTCC) buckypaper. The ITO/BP/PTCC cathode was further modified with BOD and coated with a Nafion layer to enhance stability. The buckypaper enabled increased conductive surface area, while PTCC mediated facile electron transfer. The ITO/BP/PTCC/BOD biocathode achieved currents of over 2 mA/cm^2^ under oxygen saturation in phosphate buffer, pH 7. Since a nafion membrane separated the anodic and cathodic compartments, optimizing the pH in the cathodic compartment could have improved current density even further. According to HPLC measurements, HMF underwent oxidation to 2,5-diformyl furan or 5-formyl-2-furan carboxylic acid. Both these intermediate compounds could undergo further oxidation to FDCA, the PEF precursor. In a typical experiment, 3 mM were partially converted using pristine BiVO_4_ and TEMPO, while NiFe/BiVO_4_ fully converted HMF to FDCA over 9h of continuous activity, Figure 33c. As a result, the FE for pristine BiVO_4_ reached 79.4% while NiFe/BiVO_4_ reached 100% FE. In addition, the system demonstrated impressive stability, with up to 30h of continuous activity. While BiVO_4_ is known for its stability,^231,295^ enzyme-based electrodes may suffer from stability issues. In summary, this work demonstrates the combination of photoelectrochemistry with catalysis to synthesize valuable chemicals.

**Figure 33:**
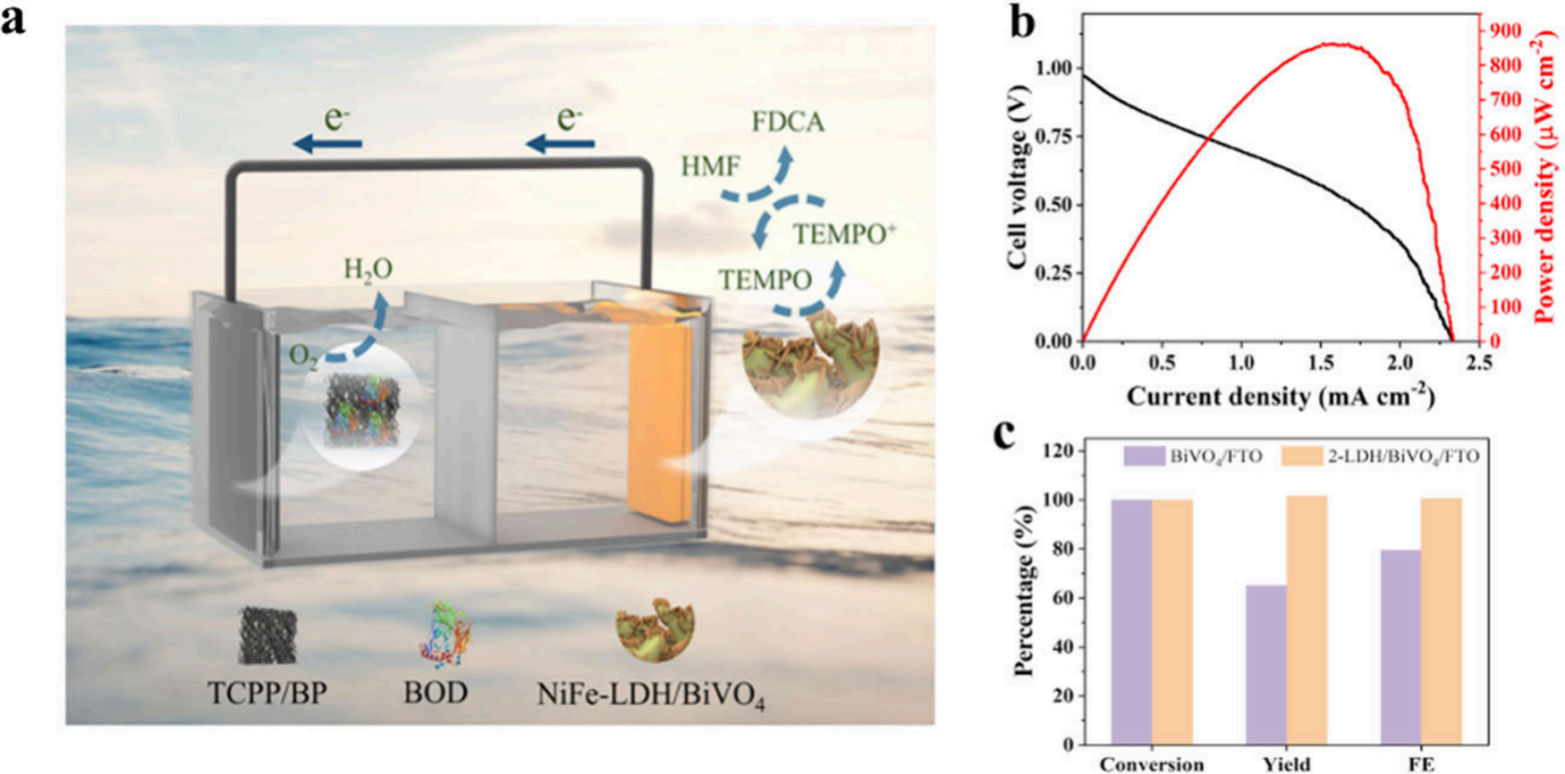
PBEC for the synthesis of FDCA, a precursor for biodegradable plastics. (a) Schematic representation of the system. While illuminated, the NiFe/BiVO_4_ photoanode oxidizes TEMPO, which in turn oxidizes HMF to FDCA. Simultaneously, the ITO/BP/PTCC/BOD biocathode uses the photocurrent to reduce oxygen to water and generate power. (b) Power curve (red) and current–voltage curve (black) of the illuminated PBEC under oxygen saturation. (c) Conversion rates of HMF after 9 h of continuous activity. While pristine and modified BiVO_4_ catalyzed HMF oxidation, only NiFe/BiVO_4_ achieved complete conversion of HMF to FDCA. Adapted from ref 294. Copyright 2025 American Chemical Society.

Bhattacharjee and co-workers have developed a bias-free photodriven biohybrid system that facilitates the conversion of PET into glycolate and formic acid, Figure 34.^296^ The designed photobioelectrochemical cell facilitated the degradation of PET fibers under strongly basic conditions.

**Figure 34:**
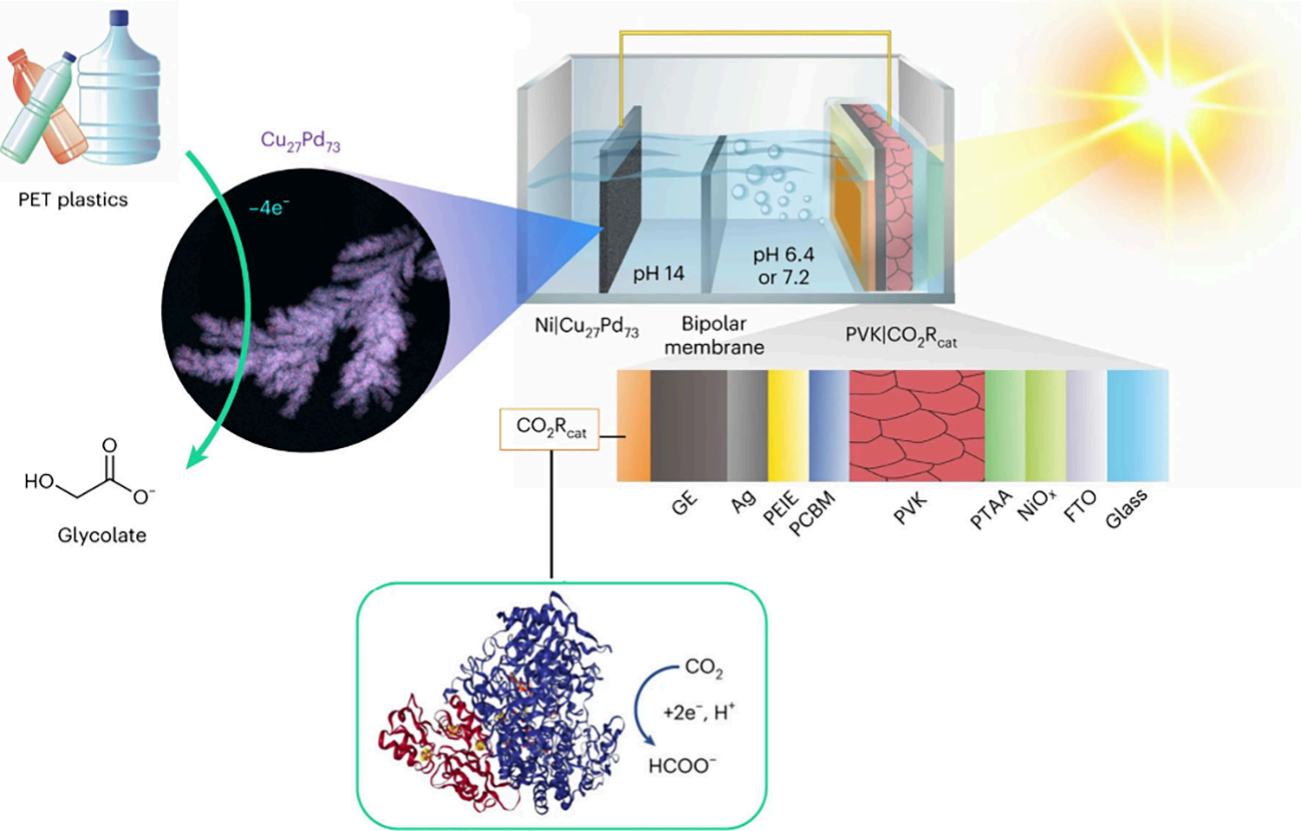
Schematic representation of the two-compartment Cu_27_Pd_73_||PVK|CO_2_R_cat_ PEC system with FDH. Adapted with permission from ref 296. Copyright 2023 SNCSC.

The anode comprising a Cu_25_Pd_73_ catalyst enables the reforming of the PET into glycolic acid. The photocathode developed the required potential to activate the oxidation of PET derivatives and the reduction of CO_2_ to formic acid using the FDH enzyme as the biocatalyst. The system was constructed with a bipolar membrane to enable the use of an appropriate pH value for the raw PET degradation and CO_2_ reduction by the enzymatic process. Furthermore, the designed configuration showed good stability for at least 10 h and continuous generation of formate and glycolate as the sole products with high faradaic efficiencies, Figure 34. This work presented for the first time an unbiased PEC biohybrid that enables the reforming of PET into fuel or value-added chemicals. While this process clearly points toward real-world application,^297^ it also holds some drawbacks: (i) it requires the utilization of strong pH values at the anode. (ii) It uses novel metal ions as catalysts. (iii) The photocathode fabrication is not straightforward.

In recent years, biotic PET degradation has been suggested. Since its initial discovery, intensive work has aimed to improve PETase enzyme activity to achieve reasonable rates suitable for industrial applications. Unlike chemical degradation, it can operate under ambient conditions and could be coupled with a PEC for further reforming. Recently, Murphy and co-workers introduced a batch-fed system that enables one-pot extrusion and enzymatic hydrolysis of PET with high efficiency and at a comparable cost to an industrial process.^298^ Such a system should be fully integrated with biotic-abiotic photoelectrochemical cells to enable light-induced reactions toward fuel or fine chemicals without any applied bias. A great demonstration toward this goal was recently presented by Kim and co-workers, Figure 35.^299^ In this research work, a Fe_2_O_3_-based photoanode was used to enable the photoconversion of degraded PET polymers into small hydrocarbons such as formate and acetate. The photoanode hole enabled the oxidation of the hydrolyzed PET under alkaline conditions, while the photogenerated electrons were further used for bioconversion reactions. As presented, the carbon-based cathode was coupled with 3 different routes that can enable biocatalysis. H_2_O_2_ or NADH was generated electrochemically using anthraquinone-2-carboxylic acid or [Cp*Rh­(bpy)­H_2_O]^2+^ electrocatalyst, respectively. The authors demonstrated key processes such as oxyfunctionalization, reductive animation, and asymmetric hydrogenation. While the developed photobioelectrochemical cell requires applied bias and alkaline conditions for its operation, it clearly puts the foundation toward efficient light-induced waste conversion into beneficial commodities such as formic and acetic acids and further enables fine chemical production using biocatalysis, where the cofactors are recycled electrochemically.

**Figure 35:**
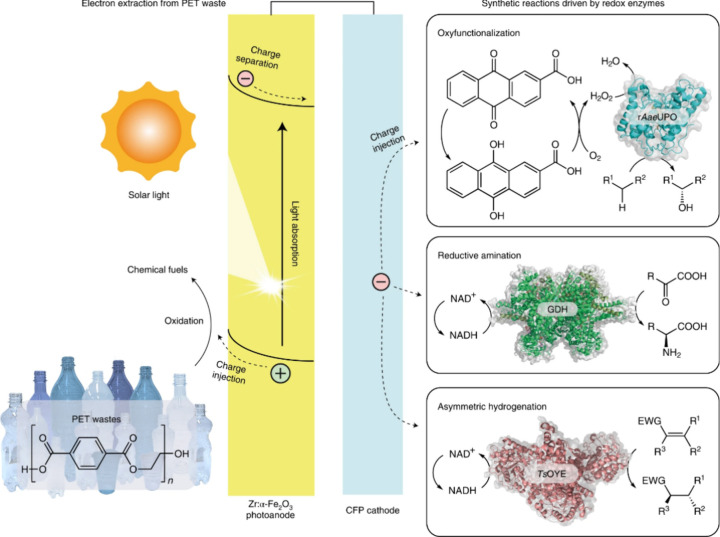
CFP-based cathodes reduce O_2_ to H_2_O_2_ for biocatalytic oxyfunctionalization and NAD^+^ to NADH for enzymatic amination and asymmetric hydrogenation. For the biocatalytic synthesis, a Zr:α-Fe_2_O_3_ photoanode extracts electrons from hydrolyzed PET solutions obtained from postconsumer PET waste. Adapted with permission from ref 299. Copyright 2022 Springer Nature.

### 7.2 Coupling PEC with Bacteria Cells toward Electrical Energy, Fuel, or Fine Chemical Production

Microbial fuel cells for the generation of electrical current were introduced over 100 years ago, and the power outputs and device constructions have since significantly evolved. Nevertheless, these cells require a feedstock, such as glucose, to generate currents and are mainly limited to electrical energy. Bacterial cells contain sophisticated machinery that can produce high-value chemicals or, alternatively, facilitate essential degradation processes.

As presented, photodriven CO_2_ reduction via enzymatic processes has great promise. Indeed, small biotic catalysts can assist in CO_2_ reduction, but they have a limited lifetime, high production costs, and cannot regenerate.^300^ Those limitations led researchers to utilize the living organisms directly as catalysts. Several families and genera of microorganisms have been known to convert gases such as carbon dioxide and nitrogen into valuable chemicals, e.g., ammonia.^301,302^ Interestingly, these micro-organisms’ activity can be enhanced by external stimuli such as electrical current applied via electrodes.^303,304^ Therefore, an external electron flow could drive microorganisms to enhance catalytic conversion toward fuel production.

In a recent study by Xiao *et al.*, a hybrid microbial photoelectrochemical system for CO_2_ reduction was presented.^305^ The system was composed of a TiO_2_/CdS photoanode and a carbon-cloth biocathode with adsorbed bacteria toward the conversion of CO_2_ into methane, a useful gas fuel for industrial processes, Figure 36a. The TiO_2_/CdS photoanode absorbed visible light and generated photocurrent. TiO_2_ is a wide-band gap semiconductor with limited absorbance in the visible range. Therefore, it was coupled with a CdS layer, which has improved visible-range absorption. Under light irradiation, an excited electron was transferred to the biocathode, while the sacrificial electron donor S^2–^ was used to close the holes formed and prevent self-oxidation of the CdS layer. The photocurrent was transferred to the bacteria via the chitosan-coated electrode. Chitosan was covalently bound to the cathode after acid treatment and NHC-EDS cross-linking. The electrostatic interaction between chitosan’s positive charge and the bacterial membrane’s negative charge enabled ET through the cathode. The photoanode and the biocathode operated at different pH levels. Therefore, a cation exchange membrane was required. To examine the biocathode’s CO_2_ reduction capabilities, cyclic voltammetry was used. bioelectrocatalytic currents could be observed at an onset of −0.45 V vs Ag/AgCl. Control experiments lacking the methanobacteria did not present any currents under the same conditions.

**Figure 36:**
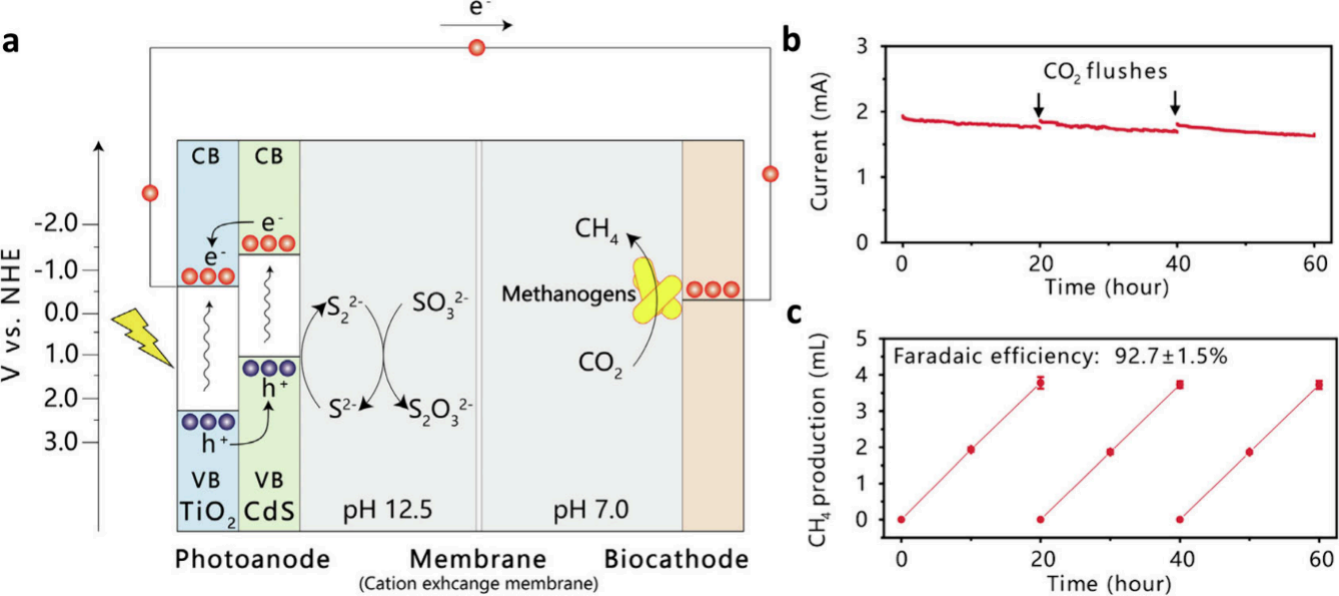
PBEC for the synthesis of FDCA, a precursor for biodegradable plastics. (a) Schematic representation of the system. The left cell contains the TiO_2_/CdS photoanode, where photocurrent is generated in response to visible light. Using sulfur as an electron source, the generated photocurrent flows toward the cathode. There, electrons are shuttled inside the methanogen bacteria to allow CO_2_ reduction. (b) PBEC photocurrent stability over time. The arrow represents an additional influx of high-purity CO_2_ gas. (c) Methane production over time in the presence of visible light. Adapted with permission from ref 305. Copyright 2020 Elsevier.

The onset potential was very close to the thermodynamic redox potential of CO_2_/CH_4_ (ca. −0.44 V vs Ag/AgCl, pH 7.0), with <50 mV overpotential for CO_2_ reduction. The authors suggest a direct electron-transfer mechanism, which is consistent with the observed low overpotential, Figure 36b. The cathodic current slightly decreased over time due to CO_2_ depletion, which was restored upon reintroducing higher CO_2_ levels. In addition, FE reached 92.7%, Figure 36c, indicating that most photocurrent was effectively channeled toward CO_2_ reduction. Overall, despite the system’s dependence on anaerobic conditions, electron donors, and CO_2_ influx, it displayed high selectivity and activity.

The system produced 15.0 L m^–2^ of methane gas per day, where the final product was a mixture of methane and CO_2_. Such outstanding selectivity minimizes downstream processing costs. Overall, the presented system provided a photocurrent to CO_2_-reducing bacteria. The bacteria were adsorbed to the cathode via electrostatic interactions with chitosan. The system’s impressive operational stability could be further enhanced by using other photoanodes. A different photoanode with a similar bandgap could eliminate the need for a cation exchange membrane. Furthermore, an improved understanding of the electron transfer process could provide essential knowledge toward the development of more efficient PECs.

Alternatively, a photocathode can be coupled to a microbial bioanode toward bias-free syngas generation. Lu and co-workers presented a biofuel cell coupled with a PEC. The developed bioanode uses wastewater streams for its activation.^306^ The mixed microbial community was integrated into anodes wired to two different circuits, Figure 37. The first bioanode was coupled to an oxygen-reducing cathode. The power generated was added to a second photoactivated PEC via a flyback circuit. The developed potential enabled the photoreduction of CO_2_ to CO at a silicon-based photocathode. The presented work provides an unbiased CO_2_ reduction system that operates without any external energy input other than light irradiation. The use of wastewater as an electron donor for the bioanode activation provides both a cheap carbon source and, in parallel, can be useful for water treatment. Unlike inorganic catalysts or enzyme-based fuel cells, bacterial cells can be regenerated, thereby providing extended lifespan operation.

**Figure 37:**
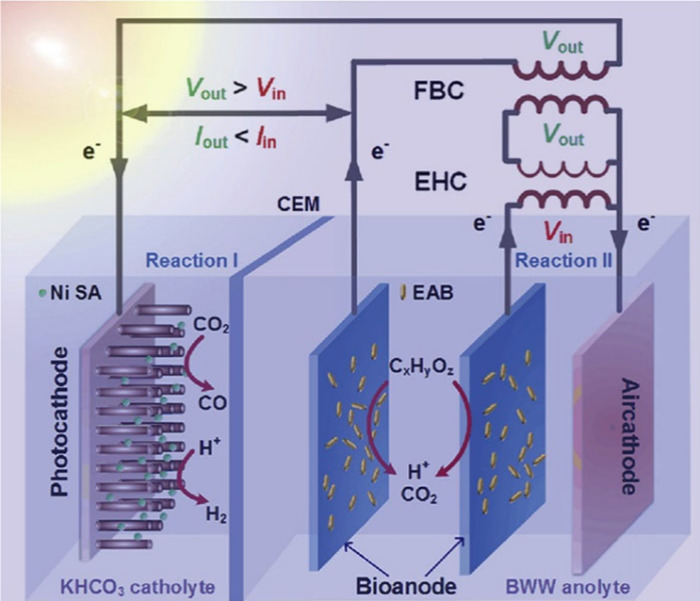
Proposed MPEC system for spontaneous solar-driven CO_2_RR. The voltage (V_in_) generated by coupling waste organics (C_X_H_Y_O_Z_) oxidation on one electroactive bioanode with oxygen reduction on an air cathode (Reaction II) was first boosted to a larger output voltage (V_out_) by an energy-harvesting circuit (EHC). The electrical energy produced was temporarily stored as electromagnetic energy. Further, a flyback circuit (FBC) was able to isolate V_out_ from Reaction II to power the solar-driven CO_2_RR on a photocathode, which is coupled with another electroactive bioanode (Reaction I). The FBC decouples Reactions I and II and combines them in one solution environment without inducing a chemical short-circuit. The waste organics (C_X_H_Y_O_Z_) in brewery wastewater (BWW) served as electron donors. Adapted with permission from ref 306. Copyright 2020 Elsevier.

A different approach integrated a CuO/ZnO/CuO-based photoanode with *S. oneidensis MR-1* bacteria to assemble a biotic-abiotic photobioelectrochemical cell for hydrogen generation, Figure 38.^307^ Under light irradiation, the photoanode generated a photocurrent that was utilized at the cathode for hydrogen generation. The holes accumulated at the photoanode were closed by the bacteria, which acted as electron donors and catalysts for lactate oxidation. Although the generated photocurrents were low, the cell showed impressive long-term stability, losing only 25% of its activity over 47 h of operation.

**Figure 38:**
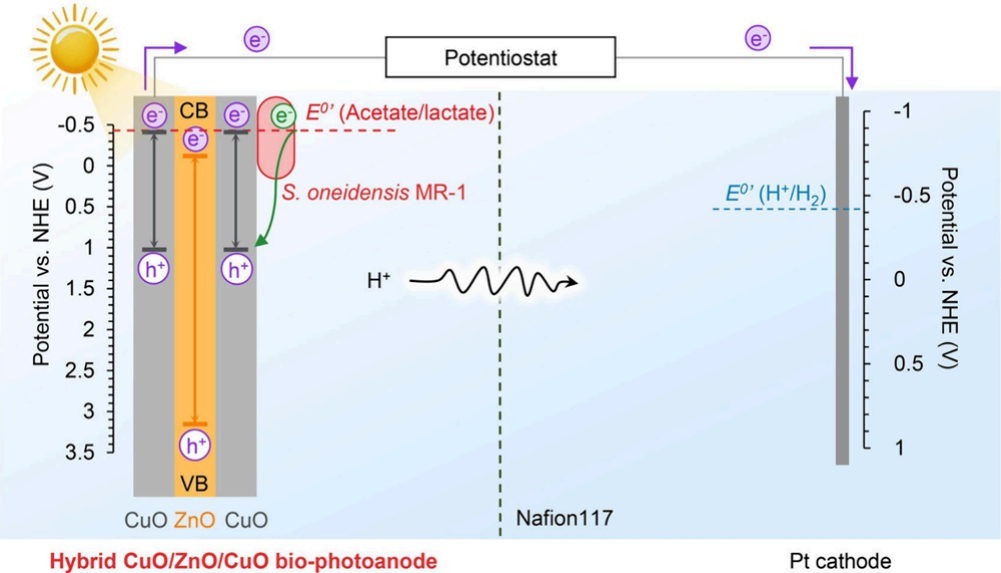
Schematic image of a proposed new M-PEC cell for H_2_ production with a biophotoanode consisting of CuO/ZnO/CuO and electroactive *S. oneidensis* MR-1. Adapted with permission from ref 307. Copyright 2024 Elsevier.

As presented, using waste as an electron source could be greatly beneficial in such biotic-abiotic cells. Furthermore, constructing unbiased systems could enable their use in real-world applications, even in areas not connected to the grid. Cellulose is the most abundant polymer on Earth. Therefore, utilizing raw waste materials comprising high cellulose content for energy generation could be very beneficial. Most known bacterial cells that facilitate catalytic cellulose degradation processes are susceptible to oxygen. Therefore, integrating biotic cellulose degradation with a water-oxidation process is hindered by the oxygen released. Moreover, water concentration in aqueous solutions reaches 55 M, which limits the oxidation of other small molecules. Another critical issue is solubility. Cellulose and other high-cellulose raw materials have low solubility. Therefore, direct photooxidation of these materials by photoanodes cannot be facilitated. Shemesh and co-workers presented a holistic approach to tackle those challenges.^308^ A photobioelectrochemical system was developed, in which *Clostridium thermocellum* was used as a biocatalyst for the degradation of raw waste materials. *Clostridium* bacteria are extremely oxygen-sensitive bacteria that secrete the cellulosome complex into the medium, Figure 39a.

**Figure 39:**
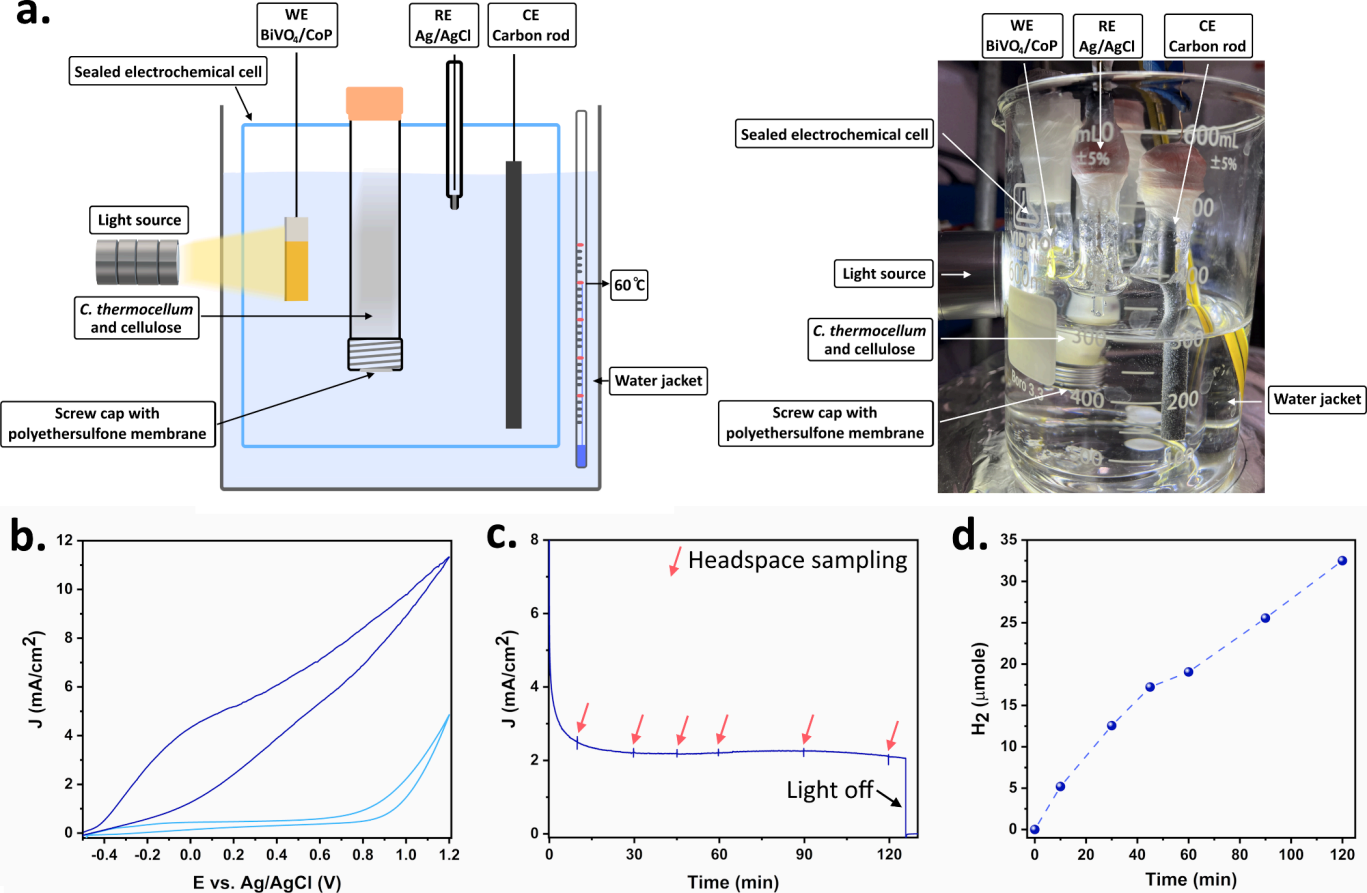
Hydrogen is generated in the MPEC comprising BiVO_4_/CoP photoanode, *Clostridium thermocellum*, and cellulose as a carbon source. (a) Schematic illustration of the constructed cell (left). An image of the constructed cell (right). For the cell construction, a specially designed sealed electrochemical cell was used. The cell consisted of BiVO_4_/CoP photoanode as the working electrode, Ag/AgCl as a reference electrode, and carbon as a counter. The outer cell solution was PB, pH 7.3, 0.2 M. The cellulose and bacteria were placed in a screw cap and were separated from the outer cell solution using a poly­(ether sulfone) membrane. A water jacket maintained the temperature in the cell solution. (b) CV measurements were performed before and after a 5 h preincubation step. A scan rate of 5 mV/s was used. (c) Chronoamperometry (CA) test performed while the photoanode was subjected to 0 V vs Ag/AgCl. Samples were taken from the headspace of the cell during the CA test. The sampling times are marked with arrows. (d) The H_2_ generated during the CA run was monitored using gas chromatography equipped with a thermal conductivity detector (GC-TCD). The cell solution was maintained at 60 °C under anaerobic conditions (O_2_ < 0.5 ppm). The photoanode was back-illuminated with white LED light during the CA measurement. Adapted from ref 308. Available under a CC BY-NC-ND license. Copyright 2025 The Author(s).

The complex consists of exo and endo β-1,4 glycosidic bond hydrolases. These enzymes enable the degradation of cellulose into cellobiose, a diglucose molecule. To utilize directly the energy stored in these sugar molecules, a photo bioanode was coupled to the bioreactor, Figure 39a. The designed BiVO_4_-based photoanode fully suppressed the water oxidation reaction while enabling the photooxidation of cellobiose, Figure 39b. As presented, photooxidation can generate high photocurrents with an onset potential of −0.45 V vs Ag/AgCl (pH 7.3). At this pH, the generated potential falls short by ∼ 200 mV to enable bias-free hydrogen generation. Therefore, a 0 V vs Ag/AgCl reference electrode was applied to drive the reaction toward hydrogen generation. As presented in Figure 39c, d, H_2_ fuel can be generated by light irradiation and an applied potential. It should be noted that the cathode in this work was not optimized to produce hydrogen, and therefore, the applied potential could be minimized by lowering the required overpotential. The designed photobioelectrochemical cell consisted of an internal bioreactor chamber that separates the insoluble raw material and the enzymes from the electrochemical cell. The poly­(ether sulfone) membrane allows degraded cellulose materials, mainly cellobiose, to diffuse to the photoanode, generating photocurrents. The electrochemical cell design, therefore, prevents fouling on the electrode surface and limits the adverse effects of shear forces on the electrode caused by the floating cellulosic material. Furthermore, an unseparated cell design presented a colored solution attributed to the oxidized quinone derivatives, which limit light penetration to the photoanode. Overall, the presented biotic-abiotic cell enabled the generation of electrical power or hydrogen (biased) while photo-oxidizing raw cellulose-containing materials such as leaves and paper sheets.

Photoinduced chemical reactions open new possibilities for novel chemistries or for eliminating the need for external energy for activation. The significant advances in biotechnology and synthetic biology provide us with state-of-the-art tools to redesign and reconfigure bacterial cells^309^ for the production of essential chemical products, sometimes even noncanonical ones.^310,311^ Furthermore, most chemical reactions facilitated by natural oxidoreductases are chiral, which in many cases is critical to activate specific processes in the cells, or could lead to adverse activity if the wrong enantiomer is used. The pharmaceutical industry aims to develop processes that lead to enantioselective manufacturing. Taking this approach, only the active molecules are produced, which, in turn, minimizes costs, increases the drug safety, and eliminates the need for expensive postmanufacturing processes for separation, such as HPLC. By coupling photoinduced reactions, for example, by combining PECs with enzymatic cascades or bioengineered bacteria, energy costs can be reduced and efficient precursor production improved. Bachar, Meirovich, and co-workers introduced a biotic-abiotic system that promoted the production of a chiral amine in a light-induced photobioelectrochemical cell.^312^ Imine reductase, IRED, is an essential NADPH-dependent enzyme that facilitates the production of chiral cyclic imines that are being used as precursors in the pharmaceutical industry. For its continuous operation, NADPH is required. As mentioned in the Introduction section, the photosynthesis process utilizes PSII and sequential charge separation to activate the FNR enzyme, which regenerates NADP and is further used in the dark cycle. Here, the photoanode oxidizes a sacrificial electron donor to generate a high reducing potential, which is then used to reduce methyl viologen, a redox-active mediator. The developed photobioelectrochemical cell can activate an enzymatic cascade to generate NADPH, which in turn is used for facilitating the (R)-2-methylpyrrolidine enantiomer, as shown in Figure 40a. The enzymatic cascade operated without any externally applied bias with the only input being visible-light irradiation. This concept was extended to whole-cell bacterial catalysis. *E. coli* was bioengineered to express both the FNR and IRED. NADPH is an essential cofactor that is naturally expressed in the cell. Therefore, IRED can be directly activated during a naturally occurring dark process in the cell. However, by coupling the PEC with the cathodic bioprocess, the NADPH levels in the cell were increased, thereby improving (R)-2-methylpyrrolidine enantiomer production by 5-fold, Figure 40b,c. The MV concentration was kept low at 0.3 mM to prevent toxicity to the bacterial cell; and enabled a continuous electron supply to FNR, thereby activating the enzymatic cascade. The developed *in vitro* system reached 99.5% substrate-to-product conversion, whereas the unbiased microbial one reached 69%. As recently suggested, the developed methodology could be easily adapted to any NADPH-dependent enzymatic cascade, both *in vivo* and *in vitro*.^313^

**Figure 40:**
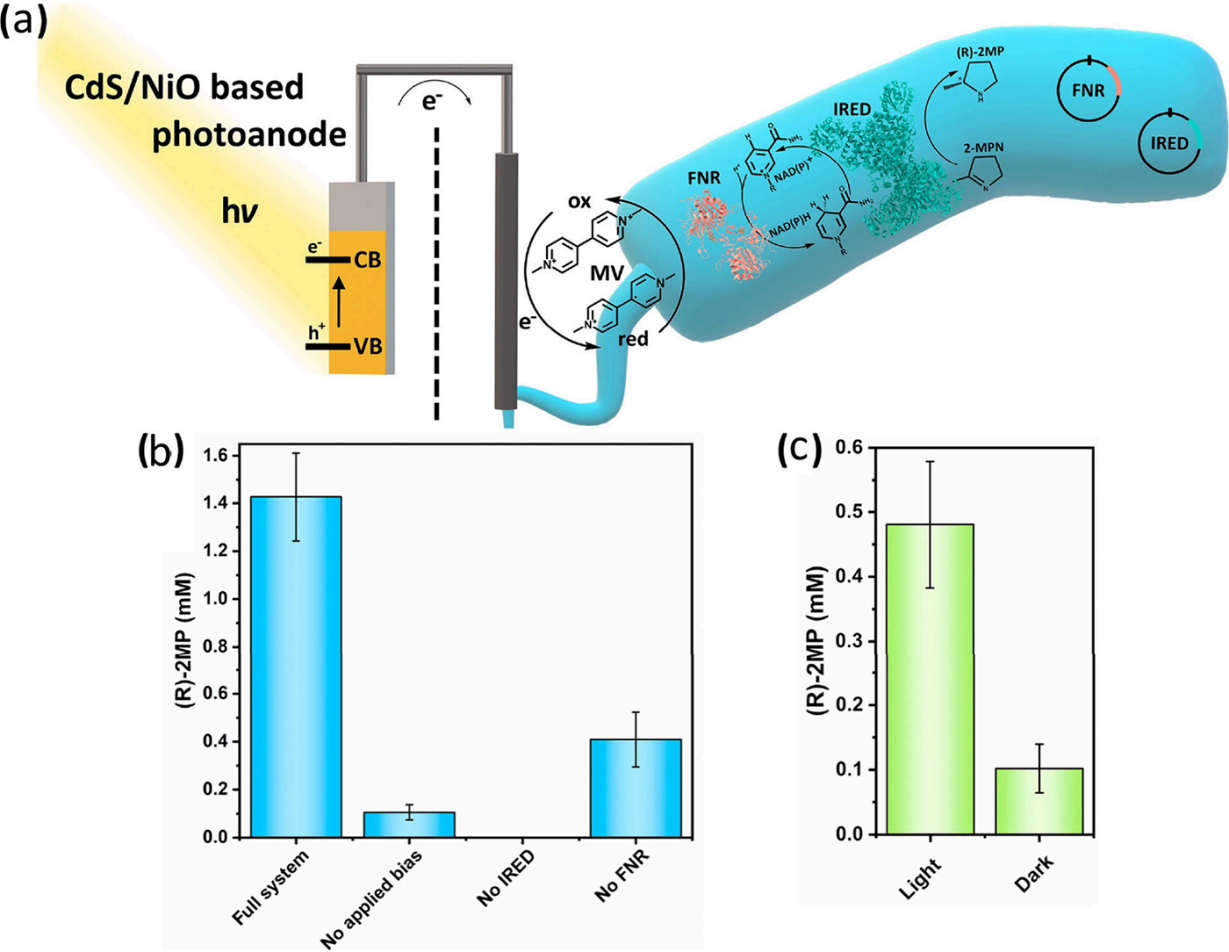
Bias-free PEC for microbial chiral amine production. (a) Schematic of the full microbial photoelectrochemical cell (MPEC) constructed by coupling a CdS/NiO photoanode to a biocathode comprising bacteria expressing the ferredoxin NADP^+^ reductase (FNR) and imine reductase (IRED) for light-induced enantioselective chiral amine generation. Protein structures were visualized by ChimeraX, Protein Data Bank (PDB) 2B5O, and 4D3S for FNR and IRED, respectively. (b) (R)-2-methylpyrrolidine [(R)-2MP] concentration in the cathodic cell chamber after 20 h of cell activation. All measurements were performed in PB 0.2 M pH 7.3 in an anaerobic environment (Ar-filled glovebox, O_2_ < 0.2 ppm), while methyl viologen (MV) (0.3 mM) and 2-MPN (10 mM) were dissolved in the cell solution (final OD_600 nm_: 0.7). Cell activation was performed in the presence of BL21 *Escherichia coli* expressing FNR and/or IRED. Carbon rods were used as working and counter electrodes, while Ag/AgCl served as a reference electrode. A bias potential of −0.75 V versus Ag/AgCl was applied during cell activation. Additional measurement without any external bias application was performed as a control. (c) (R)-2MP concentration quantification after 20 h of unbiased MPEC under white LED illumination (5 W, LEDSupply) compared with the same configuration under dark conditions. Here, 50 mM of ascorbic acid (AA) was dissolved in the anodic cell solution. The cathodic cell solution contained 0.3 mM MV, 10 mM 2-MPN, and BL21 *E. coli* expressing FNR and IRED in a final optical density (OD)_600 nm_ of 0.7. All measurements were performed in phosphate buffer (PB) 0.2 M, pH 7.3, in an anaerobic environment (Ar-filled glovebox, O_2_ < 0.2 ppm). Reproduced with permission from ref 312. Copyright 2025 Elsevier.

While the system offers many advantages and can be adapted to activate any NADPH- or NADH-dependent enzyme, it still requires an unnatural redox mediator for activation. Furthermore, although the system allows bias-free, light-induced activation, it still depends on a sacrificial electron donor. Future configurations should aim to use natural redox mediators and waste materials as sacrificial electron donors. Alternatively, Z-scheme configurations that could develop ∼ −0.7 V vs Ag/AgCl while oxidizing water could lead to outstanding performance.

## 8 Photodriven Bioconversion of Waste into Fuels or Value-Added Chemicals

### 8.1 Biomass Photobioconversion to Fuel and Value-Added Chemicals

As presented in previous sections, colloidal nanomaterials, such as quantum dots and nanorods, can be used for fuel generation. As presented, upon light irradiation, electrons gain the energy to overcome the bandgap, switching from the valence band to the conduction band. If the excited electrons’ energy level is high enough, it can thermodynamically allow H_2_ or CO_2_ reduction processes. The energy levels of nanomaterials can be tuned by controlling their dimensions. Therefore, it can be adapted to various chemical reactions. Furthermore, QDs exhibit extremely high excitation coefficients, which make them ideal for artificial photosynthesis systems.^314^ Indeed, tremendous efforts toward the utilization of NPs or nanorods for H_2_, N_2_, or CO_2_ reduction have been developed. CdS NPs have a band gap of 2.4–2.7 eV, which allows absorption in the visible spectrum, and their CB edges are positioned at ∼ −1 V vs Ag/AgCl. This strong reducing power can readily reduce protons to hydrogen (−0.62 V). However, kinetic barriers often limit production rates. The use of core–shell structures improved charge separation, extending the excited-electron lifetime and limiting recombination. Alivisatos’ lab^315^ and others^316,317^ introduced methodologies for synthesizing such nanoparticles or nanorods with an addon, a metal cluster or tip catalyst grown on top of the nanoelements for improved catalytic performance.^318^

Alternatively, the Dukovic group coupled biocatalysts to the semiconductor nanomaterials to enable efficient hydrogen generation.^319,320^ The hydrogenase enzyme was integrated into the CdS nanorods, which injected the excited electrons to facilitate the water reduction process. These biohybrids have great promise toward a more sustainable and efficient H_2_ generation platform. Nevertheless, those biohybrids require sacrificial electron donors for continuous activation. The excitation process leaves holes in the valence band, which have a strong oxidation potential that can be further utilized for oxidative photocatalytic processes. CdS NP, a common photocatalyst, develops a potential of ∼ −1.4 V vs Ag/AgCl when excited. This potential could, in principle, facilitate the water oxidation reaction (+0.81 V). However, these photogenerated holes promote rapid self-oxidation of sulfide ions, which causes a change in the lattice structure and subsequently leads to a loss of photocatalytic activity.^3,5^ Replacing the essential sacrificial electron donor with waste material could be greatly beneficial, as both waste degradation and fuel generation can be achieved in one photocatalytic process. Moreover, H_2_ generation from renewable sources is not economical. Valorization of waste by the photocatalytic process, while coupling it with H_2_ generation or CO_2_ reduction processes, can make it cost-effective for real-world applications.

Wakerley and co-workers have presented a waste-to-hydrogen photocatalytic process.^321^ CdS NPs were placed in a basic pH to form a cadmium oxide layer. By irradiation, wood or leaves were photooxidized to generate hydrogen fuel. Unlike many photodegradation configurations, which produced OH^–^ or O_2_^–^ radicals, here the use of a strong base partially degrades the cellulose, hemicellulose, or lignin, which in turn, photooxidizes to yield formic acid or is released as CO_2_, Figure 41. Nevertheless, the extreme basic condition used in this work prevents coupling it with the biodegradation process. Also, in terms of sustainability, ambient conditions should be sought in order to minimize the need for further waste treatment.

**Figure 41:**
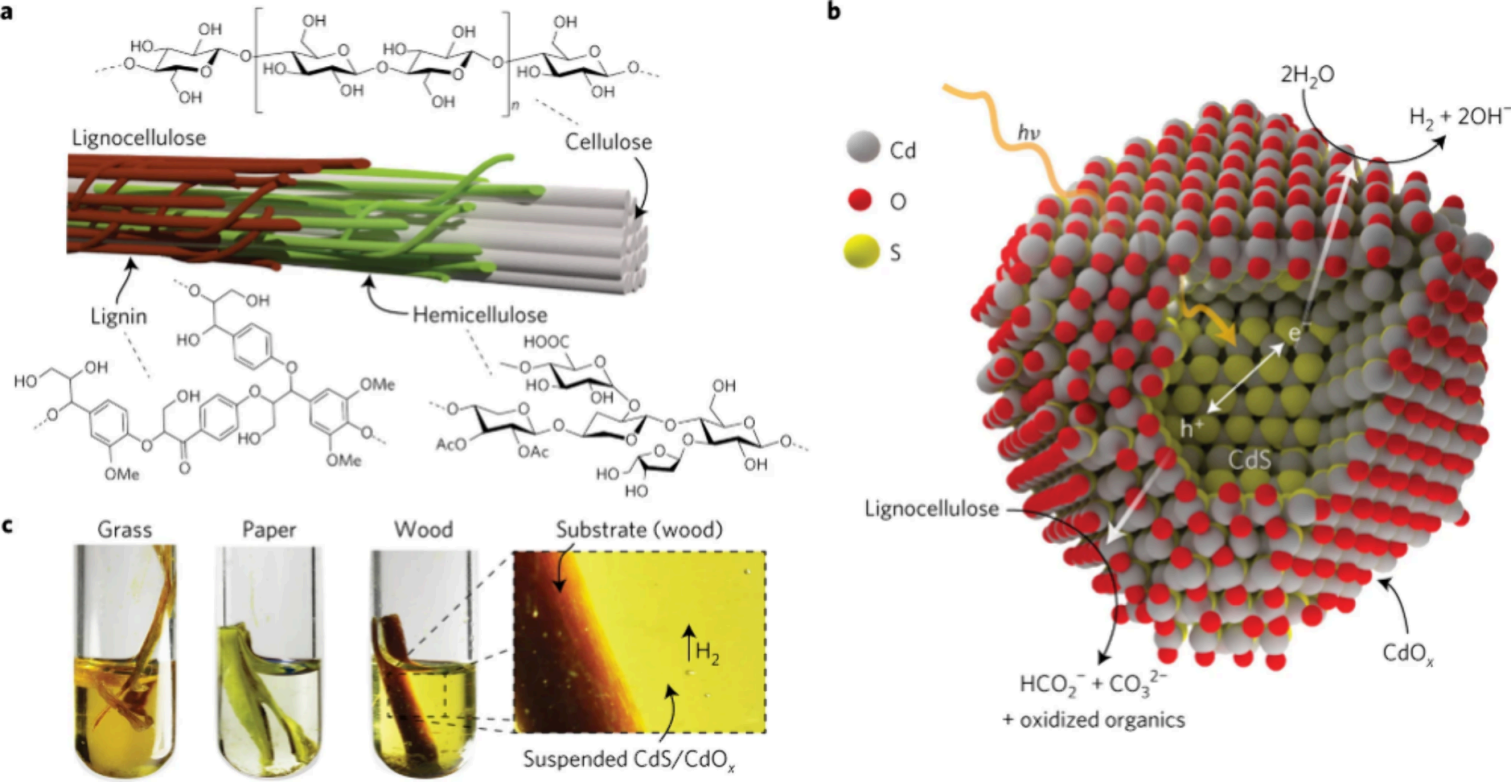
Lignocellulose exists as microfibrils in plant cell walls and is comprised of cellulose surrounded by the less crystalline polymers hemicellulose and lignin. These components can be photoreformed into H_2_ using semiconducting CdS coated with CdOx (the CdOx surface is believed to contain some −OH functionality, but H atoms have been removed in the illustration for clarity). Light absorption by CdS generates electrons and holes, which travel to the CdOx surface and undertake proton reduction and lignocellulose oxidation, respectively. This combination creates a highly robust photocatalyst that can generate H_2_ from crude sources of lignocellulose when suspended in alkaline solution and irradiated with sunlight. Adapted with permission from ref 321. Copyright Springer Nature 2017.

It has been shown that glucose can be used as a sacrificial electron donor.^322^ Ideally, hydrogen production by the photochemical process should be coupled to an enzymatic process that facilitates biomass or plastic waste degradation into small soluble molecules. The latter, in turn, may be further exploited as a sacrificial electron donor. Furthermore, an optimized system may produce value-added chemicals that can be further used in other industrial processes. As presented, raw biomass materials contain a high content of cellulose, which can be degraded by enzymatic reactions into sugars (glucose and cellobiose). Photooxidation of these materials into gluconic acid, a common food additive, or formic acid, which can be used as a fuel while producing H_2_, could provide a sustainable system with clear economic advantages compared to systems based on the commonly used ascorbic acid as a sacrificial electron donor.

Recently, Liu and co-workers presented a holistic configuration for the photochemical generation of H_2_.^323^ The designed configuration utilized a cellulose enzymatic complex to facilitate the degradation of wood chips that have a high cellulose content into glucose, Figure 42. The MoS_2_/ZnIn_2_S_4_ macrostructures facilitated the photogeneration of H_2_ fuel while photooxidizing the released glucose molecules. Rhamnose, glucuronic acid, glucose, xylose, and cellobiose were analyzed as products of the photocatalytic processes. These molecules may be further utilized as a side stream process. However, a single product is preferred to minimize postprocess separations. Similar approaches were recently reviewed^324–327^ or presented,^328^ focusing on the photocatalytic mechanism and substrate treatment.^329^

**Figure 42:**
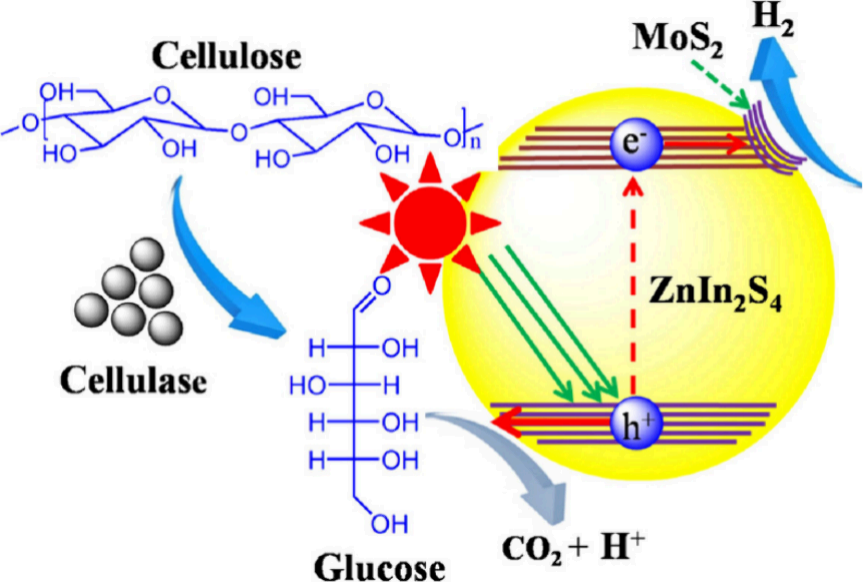
Schematic illustration for photocatalytic H_2_ production over the MoS_2_/ZnIn_2_S_4_ photocatalyst with the assistance of cellulose. Reproduced with permission from ref 323. Copyright 2022 Wiley-VCH GmbH.

While inorganic semiconductors are mainly used to promote photocatalytic reactions, Cd-based photocatalysts are toxic, and the use of cadmium-free materials is beneficial. Carbon nitride QDs are a great substitute. These materials are cheap to produce and absorb in the visible wavelength. Utilizing carbon nitride QDs as a photocatalyst for H_2_ fuel generation has high promise for future applications. Indeed, the use of carbon nitride for the photoreforming of lignocellulose has recently been demonstrated.^330^ For a broader scope, please refer to several key review articles recently published.^262,331–333^

### 8.2 Plastic Bioconversion

As mentioned, cellulose is the most abundant polymer on earth. Nevertheless, manufactured polymers are accumulating on earth and pose a threat to human health. While minimizing plastic use could reduce threats, reuse, recycling, or efficient degradation and valorization methodologies could significantly mitigate their adverse effects. Common polymers produced industrially on large scales include polyesters. Those include polyterephthalic acid, PET, and polylactic acid. The polymer chains can be degraded to their monomer form by heating under strong basic conditions. However, alternatives that use ambient conditions are more preferable. In the past decade, it has been shown that the PETase enzyme can degrade PET into bis-hydroxyethyl terephthalate (BHET) and monohydroxyethyl terephthalate (MHET), which can lead to full degradation to ethylene glycol and terephthalic acid. The enzyme degradation rate was very slow. Thus, for future applications, their rates and stability should be improved. Bioengineering techniques were used to enhance the catalytic activity of the enzyme (and its complementary enzyme, MHETase).^334–342^ In parallel, it has been found that proteinase K and other hydrolytic enzymes, such as cutinase, can facilitate the degradation of poly lactic acid into lactate.^343–346^

Coupling chemical or biological degradation processes with fuel production is highly desirable as both recycling and fuel production are gained in the process. Moreover, processes that yield value-added chemicals and do not require energy inputs or expensive separations may lead to real-world applications.^347,348^

Uekert and co-workers have presented a single-chamber reactor for the direct conversion of PET or PLA into H_2_ fuel and valuable organic chemicals.^349^ In their work, a cadmium-free photocatalyst, CN_*x*_|Ni_2_P, was utilized for H_2_ generation. The authors used pretreatment under basic conditions to allow polyester degradation. Subsequently, visible light was applied to the CN_*x*_|Ni_2_P photocatalyst to enable the generation of H_2_. As shown in Figure 43a,b, polyester microfiber and PET can be degraded and used as sacrificial electron sources for continuous hydrogen generation for at least 5 days. The authors also examined the activity of a scaled-up cell of 120 mL, as presented in Figure 43c. This work clearly presents the great potential of photocatalytic reforming as a tool for both hydrogen generation and parallel production of value-added chemicals. As mentioned above, coupling such processes may yield economically viable applications.

**Figure 43:**
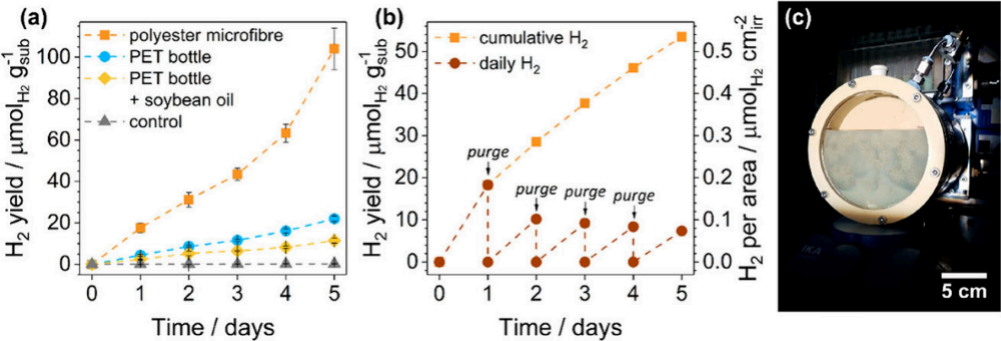
Photoreforming of nonrecyclable plastic waste. (a) Long-term photoreforming of polyester microfibers, a PET bottle, and an oil-coated PET bottle. (b) Upscaled photoreforming of polyester microfibers; the sample was purged every 24 h. Conditions: CN_*x*_|Ni_2_P (1.6 mg mL^–1^), 1 M KOH (2 mL for part a and 120 mL for part b), pretreated microfibers (5 mg mL^–1^) or PET bottle (25 mg mL^–1^) without or with soybean oil (5 mg mL^–1^), simulated solar light (AM 1.5G, 100 mW cm^–2^). (c) Photograph of the batch reactor in use. Reproduced from ref 349. Available under a CC BY 4.0 license. Copyright 2019 The Author(s). As presented, a high concentration of base was required for the degradation of the polyester. These conditions are less favorable, and alternative directions should include biotic-abiotic configurations mitigated by enzymatic or bacterial cell biodegradation. In this case, the degraded products can be used as electron donors to neutralize the holes generated by light irradiation.

Indeed, Bhattacharjee and co-workers coupled photocatalysis with enzymatic biodegradation.^350^ Two different PETase enzymes were used to facilitate PET or polycaprolactone, PCL, degradation. A Pt-loaded TiO_2_ (TiO_2_|Pt) or Ni_2_P-loaded carbon-nitride (CNx|Ni_2_P) photocatalyst with a cocatalyst was utilized for photoreforming and H_2_ generation, Figure 44. In the optimized process, AM 1.5G light irradiation and ambient temperature and pH conditions were used. It was found that TiO_2_-Pt presented much higher photoreforming performance. However, it has limited absorption in the visible wavelength range.

**Figure 44:**
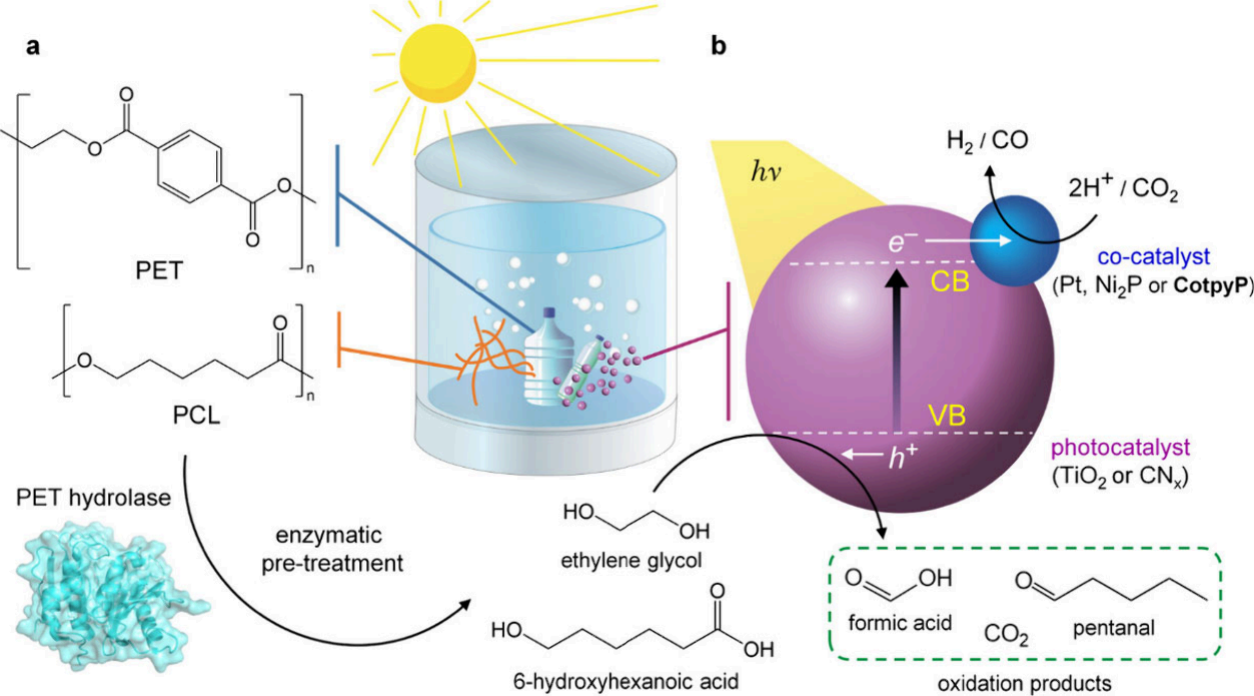
Schematic illustration of the photoreforming process with enzyme pretreatment. (a, b) Illustration of PET and PCL plastics undergoing (a) enzymatic pretreatment in solution followed by (b) photoreforming to yield valuable products. Adapted from ref 350. Available under a CC BY 4.0 license. Copyright 2023 The Author(s).

Recently, CdS@NiS nanocomposites were constructed and used for light-induced conversion of PLA into H_2_ fuel and pyruvate, a critical metabolite for the biotechnology fermentation industry, as shown in Figure 45a. Yelin and co-workers coupled the biodegradation process of PLA films facilitated by proteinase K with the photocatalytic process.^351^ Proteinase K has a wide range of hydrolytic capabilities. It can cleave amide bonds and degrade peptides or, alternatively, degrade polyesters such as PLA. The enzyme was used to degrade the PLA films into lactic acid monomers that were then used as sacrificial electron donors. The photocatalytic hydrogen generation process was enhanced by the deposition of NiS on the 9 nm CdS NPs. It was found that a very low concentration of NiS clusters on the NPs’ surface dramatically enhanced the hydrogen generation rates. Interestingly, the lactic acid photooxidation process has led to a single product, pyruvate, as shown in Figure 45b. The highly selective process offers significant advantages for future industrial applications as it minimizes the need for additional separation steps. The biotic-abiotic configuration used ambient conditions for its activation, which is critical for sustainability. Photoinduced H_2_ generation has been pursued for many years. However, the use of sacrificial electron donors and novel metals makes the process noneconomic. Here, no novel metals are used and waste plastic is used to produce sacrificial electron donors.

**Figure 45:**
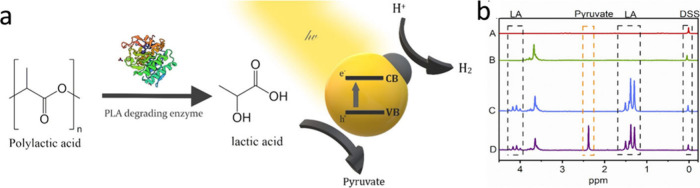
a. Schematic representation of the biotic-abiotic configuration. (i) biodegradation, (ii) photocatalytic reaction. Inset: High-resolution TEM image of the CdS@NiS nanoparticles. b. ^1^H NMR spectrum (A) only PB 0.2 M pH = 7.3 (B) illuminated NPs with no LA (C) NPs with LA not illuminated (D) illuminated NPs with LA. Adapted from ref 351. Available under a CC BY-NC 4.0 license. Copyright 2026 The Author(s).

Furthermore, the photooxidation process and the generation of pyruvate add to the economic gain. Ironically, the process has higher financial contribution potential than that achieved by the H_2_ fuel production. Thus, coupling these two processes is economically viable. Such production-coupling processes may incentivize the industry to adopt the biotic-abiotic approach. Just recently, an interesting bacterial consortium and an abiotic photocatalyst biohybrid were introduced. The microbial consortia were coupled with a TiO_2_/Cu_2_O photocatalyst to enable both the photodegradation of poly­(vinyl alcohol) and its further assimilation by the bacteria to produce enhanced cofactor regeneration and amino acid production.^352^

## 9 NAD(P)H Regeneration, Using a Photocatalytic Process

Biocatalytic systems enable the sustainable synthesis of complex molecules by minimizing the use of harsh reagents and reducing byproduct formation. However, many enzymatic processes depend on redox cofactors, which serve as essential electron or energy carriers. In photosynthetic organisms, NADPH drives carbon fixation through the Calvin–Benson–Bassham (CBB) cycle, converting CO_2_ into carbohydrates.^353^ Beyond photosynthesis, NADPH serves as a universal hydride donor for a broad spectrum of oxidoreductases, facilitating key redox transformations, such as fatty acid and nucleotide biosynthesis. Its regeneration and selective use are essential for enabling continuous biotransformations in both natural and engineered systems.^354^ Since the stoichiometric use of NADPH and other cofactors (e.g., ATP, FAD, and FMN) is often economically unfeasible at scale, efficient cofactor regeneration strategies are crucial for maintaining enzyme activity while minimizing costs.^355^

NAD­(P)H regeneration can be achieved through various approaches, including chemical reduction, photochemical methods, electrochemical methods, or coupling complementary enzymatic reactions.^356,357^ Among these, photochemical and (photo)­electrochemical methodologies have emerged as particularly attractive due to their ability to harness light energy for cofactor recycling. In the context of NAD­(P)H regeneration, the stereoselective reduction of NAD­(P)^+^ to its active form, 1,4-NAD­(P)­H, is a key consideration.^358^ The development of a homogeneous catalyst for electro-regeneration of NAD or NADP to the 1,4 active form opened new possibilities to interface electrodes with enzymatic activation.^359–362^ The CpRh­(bpy)­H_2_O catalyst was also conjugated to a photoinduced reaction to enable the generation of NAD­(P)­H. The conjugated process ties light-induced reactions with NAD­(P) regeneration and acts as an alternative to the photosynthesis process.^363–365^ It is important to note that direct photochemical reduction of NAD­(P) could be achieved using the NP or the photosensitizer as a catalyst; however, this leads to lower stereoselectivity. Limited NADPH regeneration selectivity forms dimers or the 1,2 or 1,6 isoforms, resulting in a nonviable process. In these configurations, sacrificial electron donors are required for continuous NADPH photorecycling. Alternatively, the photocatalytic reaction for NADPH generation can be coupled with a water oxidation reaction and a tailored charge separation and electron transfer chain to achieve an artificial Z-scheme configuration.^366^

In nature, only the 1,4-isomer is utilized by NADPH-dependent oxidoreductases. At the same time, nonselective photochemical reduction often leads to enzymatically inactive byproducts such as 1,2- and 1,6-NADPH isomers or dimers, which can inhibit enzyme activity.^367^ To address this, nature employs ferredoxin-NADP^+^ reductase (FNR), an enzyme that catalyzes the stereoselective formation of 1,4-NADPH.^368^ By coupling inorganic photocatalysts (including semiconductors, metal complexes, or carbon-based materials) with FNR, highly selective and efficient NADPH regeneration can be achieved under mild, light-driven conditions. Once a robust NADPH regeneration platform is established, it can be linked to downstream enzymatic reactions that require this cofactor. In such integrated systems, only catalytic quantities of NADP^+^ are needed as continuous regeneration sustains the biocatalytic cycle.

Solar-driven NADPH regeneration broadly divides into two main categories: photochemical and (photo)­electrochemical. Photochemical schemes use dissolved photosensitizers to harvest light and deliver reducing equivalents in homogeneous solutions, whereas (photo)­electrochemical strategies employ illuminated or biased electrodes as the driving force for selective cofactor recycling.

### 9.1 Photochemical NAD­(P)H Regeneration

The native solar-driven regeneration of NADPH in the photosynthetic electron transport chain has been demonstrated *in vitro* using purified PSI as the photosensitizer and FNR as the acceptor.^369^ The activation of various NADPH-dependent enzymes demonstrated the versatility of the system. However, it requires the labor-intensive isolation of PSI from cyanobacteria as well as the addition of DCPIP and ferredoxin as electron mediators. King and co-workers developed biohybrid systems comprising CdSe QDs coupled with FNR for the light-driven regeneration of NADPH.^370^ This regenerated NADPH was then used by alcohol dehydrogenase to reduce aldehydes to alcohols, simultaneously recycling NADP^+^ to close the catalytic cycle. This concept has been further developed by Bachar and co-workers in a study that demonstrated protein-mediated synthesis of photocatalytically active CdS QDs (Figure 46a).^371^ These biosynthesized QDs facilitated solar-driven NADPH regeneration that, in turn, was used for the activation of the imine reductase (IRED) enzyme. The latter catalyzes the asymmetric reduction of cyclic imines to yield chiral amines, which are highly valuable in pharmaceutical and agrochemical syntheses. For 1,4-NADH regeneration, the diaphorase enzyme has been employed for the direct reduction of NAD^+^ into enzymatically active NADH without oxidizing any other product. Yuan and co-workers reported high 1,4-NADH selectivity using immobilized diaphorase in a redox polymer modified with a cobaltocene mediator.^372^ Another interesting study has tested the incorporation of CdTe QDs with thylakoid extracts in artificial photosynthetic cells. The authors used inorganic CdTe QDs to enhance the flux of photogenerated electrons for the regeneration of various cofactors, including NADH and ATP. The developed platform demonstrated its promise in activating various cofactor-consuming oxidoreductases, including formate dehydrogenase and nitrogenase.^373^

**Figure 46:**
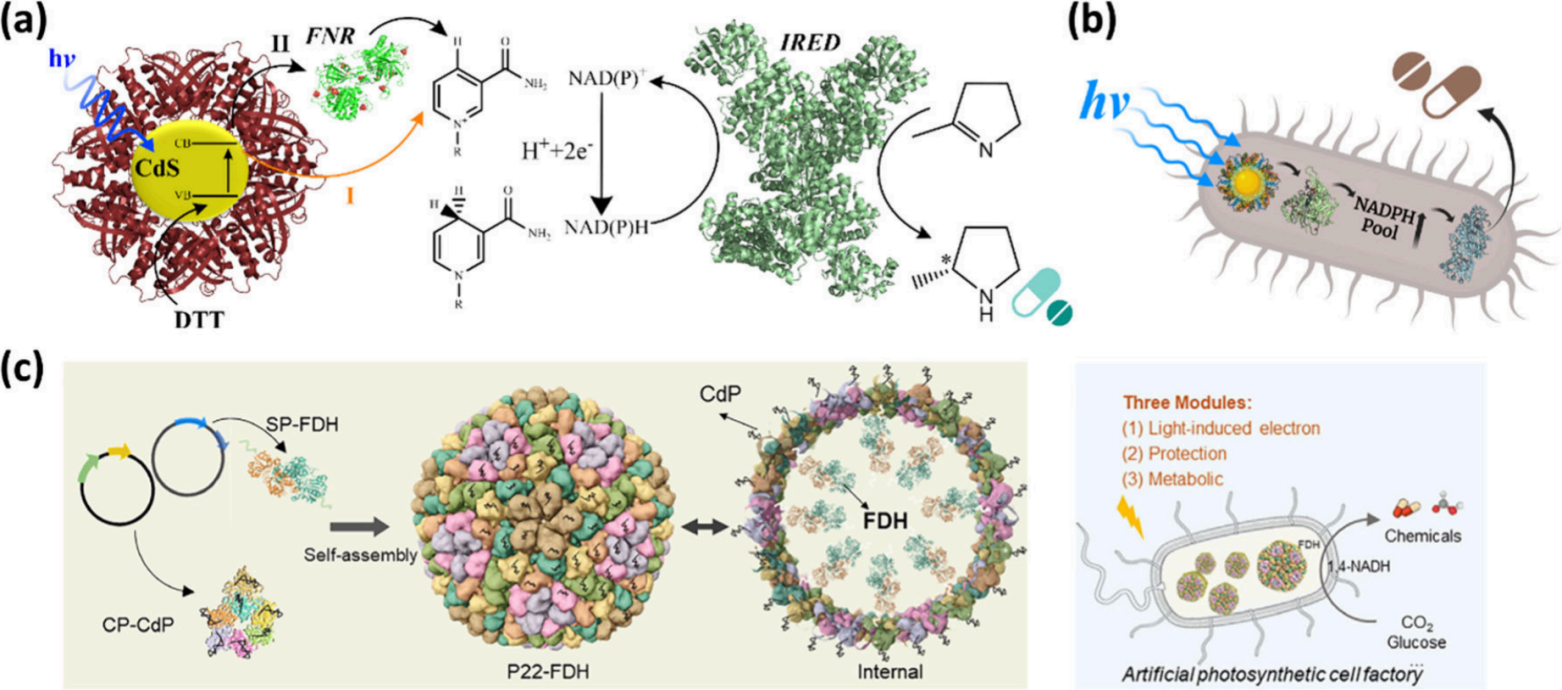
Using engineered protein cages in photosynthetic whole-cell biohybrids for directed bottom-up biosynthesis of inorganic nanomaterials to drive intracellular NADPH regeneration. (a) A demonstration of an *in vitro* cascade for CdS QDs biosynthesis and their use in FNR-facilitated NADPH regeneration. In turn, the reduced cofactor activates NADPH-dependent enzymatic activity for chiral product generation. Adapted with permission from ref 371. Copyright 2022 Wiley-VCH GmbH. (b) the *in vivo* implementation of the system presented in (a) inside a living *E. coli* for whole-cell biomanufacturing. Adapted from ref 209. Available under a CC BY-NC 4.0 license. Copyright 2023 The Author(s). (c) Designing protein cages for directed inorganic nanomaterials synthesis and colocalization of NADPH-consuming enzyme. This methodology is incorporated inside a living host for enhanced ET between the generated nanoparticles and the target reductive cascade. Adapted with permission from ref 377. Copyright 2025 Wiley-VCH GmbH.

Alternatively to FNR and diaphorase, selective 1,4-NADPH regeneration is commonly achieved using Rh-based electron mediators such as [Cp*Rh­(bpy)­(H_2_O)].^372^ Since first introduced as a selective catalyst for NAD­(P) regeneration, its use was widely extended to be activated electrochemically, photoelectrochemically,^299^ and photochemically. In a recent work, the latter was incorporated with InP QDs to enable solar-driven NAD­(P)­H regeneration for the activation of alcohol dehydrogenase.^374^ Additionally, Macchioni and co-workers have shown the incorporation of iridium-based complexes, enabling highly selective NAD^+^ reduction.^358^ Xing and co-workers incorporated a photocatalytically active ZnIn_2_S_4_ semiconductor with a Rh-based catalyst to enable selective NADH regeneration.^375^ Interestingly, in parallel with NAD^+^ reduction, the polarized semiconductor enables selective alcohol oxidation, which is utilized as a sustainable sacrificial electron donor platform in the system. However, these chemical catalysts are often required in relatively high concentrations, which might be incompatible with biological systems.^376^ Moreover, the use of enzymes to enable 1,4-NAD­(P)H selectivity is preferable in whole-cell biohybrid systems since it could be intrinsically expressed by the host bacterium, minimizing the external addition of chemicals to the system.

### 9.2 (Photo)­electrochemical NAD­(P)H Regeneration

Although colloidal inorganic nanomaterials are attractive light harvesters, practical deployment is hampered by reliance on toxic heavy-metal precursors and colloidal instability, which together undermine biocompatibility and scalability.^356^ Alternatively, NADPH regeneration can be embedded within a photoelectrochemical architecture using photoelectrodes to deliver electrons to a biocathode. This approach decouples the light absorber from the biological entity, offering a more controllable, scalable route to real-world implementation. Hambourger and co-workers have presented a dye-sensitized photobioelectrochemical cell which enables the oxidation of NAD­(P)­H, and the generation of hydrogen fuel.^378^ The designed system comprises the hydrogenase enzyme as a catalyst for hydrogen generation at the cathode, as shown in Figure 47. By excitation, high-energy electrons are shuttled from the anode to an external circuit. In parallel, the strong oxidative potential developed at the photoanode surface enables the oxidation of NADH to NAD^+^. While in most cases the NAD­(P)^+^ reduction process is preferred, as will be presented next, in some processes, regeneration of oxidation states of oxyreductases, such as alcohols to aldehydes, can be beneficial.

**Figure 47:**
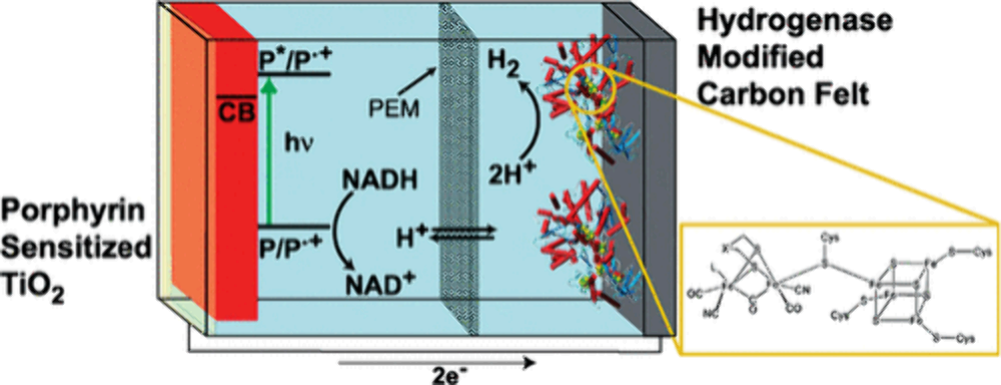
Schematic drawing of the dye-sensitized photobioelectrochemical cell comprising a NADH oxidation process by the photoanode and H_2_ generation by the hydrogenase enzyme at the cathode. Adapted from ref 378. Copyright 2008 American Chemical Society.

Minteer and co-workers reported a bioelectrocatalytic method for regenerating NADH via both enzymatic and electrocatalytic routes.^175^ In this work, an electrode has been modified to facilitate NADH regeneration and the reduction of acetoacetyl-CoA, which, in turn, was applied to the synthesis of polyhydroxybutyrate.^175^ Plumeré and colleagues demonstrated FNR-mediated solar-driven NADPH regeneration using a redox-active hydrogel to activate enzymatic reductive carboxylation.^379^ Our recent work has integrated an NADPH-regeneration module into the cathodic compartment of a PEC to achieve continuous, light-driven synthesis of chiral amines.^312^ In a representative setup, an illuminated CdS photoanode delivers electrons to a biocathode that regenerates NADPH to power an imine-reductase cascade; the device operates bias-free as a stand-alone system. The concept has been demonstrated both *in vitro* (with FNR and IRED) and *in vivo* (with whole-cell catalysts expressing the same enzymatic pathway).

### 9.3 Employing NAD­(P)H Regeneration Systems in Whole-Cell Biohybrids

In whole-cell photocatalytic biohybrids, light-driven NADPH regeneration is leveraged not merely to feed a single enzyme but to affect the cell’s intracellular redox state toward target reductive pathways. As previously mentioned, this methodology could be practically implemented after establishing an interface between an external light harvester and a living organism. Guo and co-workers engineered yeast–InP nanoparticle biohybrids in which light-excited, cadmium-free nanoparticles inject electrons into the cytosol, raising the free NADPH/NADP^+^ ratio and thereby driving shikimic-acid production.^380^ This study established a viable whole-cell route to *in vivo*, light-powered NADPH regeneration for fine-chemical synthesis, with maintained cell viability and a modular interface that is broadly adaptable to other NADPH-dependent pathways. Yuan and co-workers coated Ni-doped semiconductor ZnGa_2_O_4_ with polydopamine, claiming improved electron transfer between the inorganic photocatalyst and intracellular components, including NADPH.^381^ Moreover, the heterologous expression of rhodopsin allowed for solar-driven ATP regeneration, thereby boosting CO_2_ fixation and lycopene biosynthesis. In a recent article, a similar approach for dual intracellular NAD­(P)H and ATP enhancement was enabled by eosin Y and rhodopsin, respectively, in Gram-negative *Cupriavidus necator*.^382^ These energy-rich cofactors were used to enhance CO_2_ fixation to acetoin. Liu and colleagues investigated photocatalytic material-microbial interfaces using proteomics and metabolomics and reported that illuminated biohybrids maintain redox and energy homeostasis comparable to hydrogen-fed controls.^383^ This work supports the assumption that a steady photoelectron supply can sustain intracellular reducing power (including NADPH) in whole-cell systems without disrupting cell metabolism. Recently, photosynthetic microbes were combined with inorganic photoabsorbers, allowing the organisms to store chemical energy that can be released in the dark to enable “all-weather” biomanufacturing.^384^ It has been shown that steady external electron transfer provided by the inorganic light-harvesters allows for enhancing intracellular NADPH pools that, in turn, expand the operating window for light-driven reductive biocatalysis. Spatial organization further improves efficacy. Engineered condensates or protein cages can colocalize photosensitizers with NADPH-consuming enzymes, shorten electron-transfer distances, and insulate productive chemistry from competing sinks. Liu *et al.* designed a modular self-assembled protein-cage strategy in living bacteria that spatially colocalizes photoactive components with redox enzymes to achieve selective intracellular NADH regeneration, which in turn boosts CO_2_-fixation activity (Figure 45c).^377^ The desired effect is ultimately measured by sustained elevation of the NADPH/NADP^+^ ratio, representing the enhancement of reducing power to drive desired reductive biotransformations. It is noteworthy to mention that this NADPH/NADP^+^ ratio is commonly measured using commercial colorimetric kits based on simple redox processes. While these methods are valid for ordinary *in vivo* redox balance measurements, one should take into consideration the effect of the artificial inorganic redox moieties on the assay readout. Therefore, we believe that validating such data with other methodologies (e.g., LC-MS) is highly important. Remaining challenges include mediator biocompatibility, competition with native metabolism, matching NADPH supply with ATP demand, and adoption of heavy-metal-free light harvesters for scalable operation. Overall, the integration of inorganic photocatalysis and whole-cell biocatalysis offers a powerful and sustainable route for cofactor recycling and light-driven enzymatic synthesis, with significant implications for green chemistry and synthetic biology.

The biotic-abiotic configurations presented in this work use redox proteins, enzymes, or bacterial cells, in conjunction with redox polymers and abiotic elements. Each of these elements has a specific redox potential for activation. In order to design new artificial photosynthesis and biotic-abiotic configurations, one should take into consideration the energy levels and the redox potential of each component. While most of the redox proteins have a clear redox potential, in some, like the iron-protein, Fe-P of the nitrogenase, their potentials are shifted as a result of ATP binding. Also, both biotic and abiotic elements can undergo photoexcitation and have excited electrons with higher energy levels. The band gap and energy levels of semiconductor materials at the nanoscale can be tuned by controlling their dimensions, a phenomenon known as the quantum size effect. Toward new configuration design, we summarized the majority of the elements used in the review in comprehensive energy diagrams, as shown in Figure 48. For convenience, we have separated the energy levels and redox potentials into three panels: (i) band gap and energy levels of semiconductors. (ii) Redox mediators and photosensitizers. (iii) Photosynthetic proteins, redox proteins, and enzymes.

**Figure 48:**
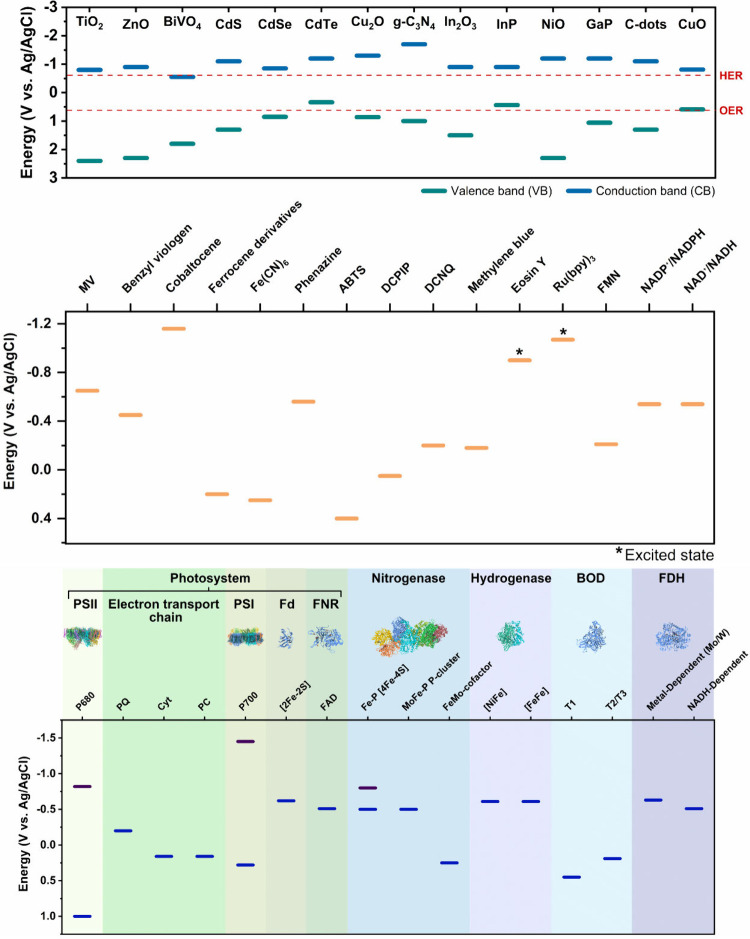
Energy diagrams of commonly used semiconductors, redox mediators and biotic elements.

## 10 Qualitative and Quantitative Methodologies to Follow Waste Conversion Using Biohybrid Configurations

The photosynthesis process converts light energy into chemical energy through a series of light- and dark-reaction steps. Over the last couple of decades, various configurations have been developed to enable the capture and conversion of CO_2_ into fuels. These methodologies comprise a chemical catalyst, a biocatalyst, or a photocatalyst. Most designed systems do not include both light-induced water oxidation and CO_2_ assimilation apparatuses. The 2e^–^ and 2H^+^ reduction processes could generate formic acid, which can be used directly as a fuel or as a hydrogen carrier. Many photo­(bio)­catalytic and photo­(bio)­electrocatalytic configurations have been developed to facilitate CO_2_ reduction processes to formic acid. These configurations omit the water oxidation process for electron generation and instead use sacrificial electron donors. One such experiment should consider several issues:

Most of the experiments were performed in aqueous solutions. Therefore, the dominant form is that of formate. In its charged form, it cannot be measured accurately by a gas chromatograph.

Formic acid can be extracted after acidification and distillation and separation. However, one should be cautious as such a process can yield false-positive results. Photoinduced reactions oxidize sacrificial electron donors, such as ascorbic acid, TEOA, DTT, and EDTA. These molecules undergo oxidation and further degradation. The process may lead to the degradation of the oxidized sacrificial electron donor backbone, yielding formic acid, which is mistakenly thought to originate from the CO_2_ reduction process. To minimize false-positive results, ^13^CO_2_ should be used as the CO_2_ precursor. H NMR measurements of ^13^C formic acid show a distinguishable signal,^385^ which confirms that the formate origin is the marked CO_2_. Furthermore, an internal standard, e.g., DSS, should be added.

While ammonia production is not directly connected to photosynthesis, it is of great importance to many industrial processes and is therefore indirectly related. Ammonia is commonly detected by fluorescence or absorption probes. The most frequently used probe is phthalaldehyde, which reacts with ammonia in the presence of thiols to exhibit a strong fluorescence at 475 nm.^386^ One should take into account that the obtained fluorescence is greatly affected by the pH, the presence of proteins, and other amines. Therefore, to limit false positives, H NMR measurements should also be included. Ammonium ions give a distinguishable triplet split at 6.42, 7.07, and 7.72 ppm, with a spacing between the triplet peaks of 52 Hz. The origin of the ammonium can be further verified using 15N-labeled substrate material. The ammonium exhibits doublet peaks at 6.57 and 7.48, separated by 72.8 Hz.^387^ additional spiking tests with ammonia should be performed to confirm that the results align with the desired products.

While GC can easily detect methane, H_2_, and small MW alcohols, equipped with TCD and FID detectors and a molecular sieve column, fine chemicals may require several steps of derivatization and separation. Moreover, fine chemicals are often chiral and should therefore be detected and identified using a chiral column. In many cases, a derivatization process is required for improved separation into the organic phase.^388^

The efficiency of waste conversion using biohybrid configurations can be assessed using various parameters that reflect the utilization of light energy, charge, and chemical energy.

To enable an accurate assessment and valid comparison between the different system configurations, standardized experimental protocols should be established. Several parameters have been defined to characterize various system configurations including electrochemical, photoelectrochemical, and photochemical configurations.

The efficiency of electrochemical reactions is commonly evaluated using Faradaic efficiency. **Faradaic efficiency** (FE) reflects the selectivity of an electrochemical reaction toward the generation of a particular product.^389,390^ It is defined as the ratio between the charge utilized for the generation of the specific product to the total charge passed through the electrochemical system, according to eq 1:

1 $$FE\left(\%\right)=\frac{Q_{\mathrm{product}}}{Q_{\mathrm{total}}}\times 100$$

For products present in the liquid phase, the FE is calculated using eq 2:^389^

2 $$FE_{\mathrm{liquid}}\left(\%\right)=\frac{N\times n\times F}{Q_{\mathrm{total}}}\times 100$$

Where N is the number of moles of the generated product, n is the number of electron moles required for the generation of 1 mol of product, and F is the Faraday constant (96485 C·mol^–1^).

Several parameters have been defined to characterize the efficiency of photoelectrochemical systems. **Incident photon to current efficiency (IPCE)** is a widely used measure for photoactive devices that describes how photon-induced electron–hole pairs are converted into useful electrical energy. IPCE is also referred to as the **External Quantum Efficiency (EQE)**.^391^ The IPCE is a function of photon wavelength (λ), as it reflects the photocurrent response of the system under illumination at each specific wavelength point.^391^ The IPEC measure is described according to the eq 3:

3 $$IPCE_{\left(\lambda \right)}\%=\frac{I_{\mathrm{el}}}{I_{\mathrm{ph}}}\times 100$$

Where *I*_el_ represents the flux of electrons flowing through the external circuit (mol·m^–2^·sec^–1^) and *I*_ph_ denotes the incident photon flux (mol·m^–2^·sec^–1^). The electron flux is derived from the ratio of the photocurrent density (J, μA·cm^–2^) to the Faraday constant (96485 C·mol^–1^). The photon flux is calculated from the power density of the incident light (W, W·m^–2^) and the wavelength (λ, nm) divided by the Avogadro number (*N*_A_ = 6.022 × 10^23^ mol^–1^), Planck’s constant (*h* = 6.626 × 10^–34^ J·sec) and the speed of light (c = 2.998 × 10^8^ m·sec^–1^), according to eq 4:^392,393^

4 $$IPCE_{\left(\lambda \right)}\%=\frac{\frac{J\times 10^{-2}}{F}}{\frac{W\times \lambda \times 10^{-9}}{N_{A}\times h\times c}}\times 100≅\frac{1240\times J}{\lambda \times W}$$

**Applied bias Photon-to-Current Efficiency (ABPE)** is an additional performance measure that describes the efficiency of a photoelectrochemical system to convert incident photons into electrical current under an externally applied bias. It is calculated according to eq 5:^394^

5 $$ABPE\%=\frac{J\times \left(1.23-V\right)}{P}\times 100$$

where *J* is the photocurrent density generated under an applied voltage of V vs RHE. *P* stands for the light intensity.

**Quantum yield (QY)** or **quantum efficiency** is commonly used to evaluate the activity of photocatalysts. The quantum yield of a photochemical reaction is defined as the ratio between the number of reacted electrons and the number of photons, of a specific wavelength, absorbed by the photocatalyst, according to eq 6:^395,396^

6 $$QY\left(\%\right)=\frac{number\;of\;reacted\;electrons}{number\;of\;absorbed\;photons}\times 100\%$$

The absorbed photon flux is affected by factors such as light intensity, the optical properties of the photocatalysts, and the substrate.^396,397^ Measuring the absorbed photon flux is straightforward in homogeneous systems but challenging for heterogeneous reactions. In these systems, light scattering and reflection reduce the fraction of incident light that is absorbed. The losses can amount to 13–76% of the total incident photon flux.^396,397^ The **apparent quantum yield (AQY)** is widely used to evaluate the photocatalytic performance, as it considers the total number of incident photons rather than only those absorbed by the photocatalyst. By replacing the number of absorbed photons with the number of incident photons in the quantum yield calculation, the resulting value corresponds to the AQY.^395^

The number of incident photons can be determined according to the ratio between the total energy of the monochromatic light and the energy of one photon, according to eq 7:

7 $$Number\;of\;incident\;photons=\frac{E_{total}}{E_{photon}}=\frac{P\times S\times t_{irr}\times \lambda }{h\times c}$$

Where P is the incident monochromatic light power density (W·m^–2^), S is the irradiated area (m^2^), *t*_irr_ is irradiation time (sec), λ is the wavelength (m), *h* is Planck’s constant (6.626 × 10^–34^ J·sec), and c is the speed of light (2.998 × 10^8^ m·sec^–1^).

The number of reacted electrons can be evaluated according to the number of target product molecules generated and the number of electrons required for their generation. The AQY is therefore calculated according to eq 8:

8 $$AQY\left(\%\right)=\frac{number\;\mathrm{of}\;\mathrm{reacted}\;\mathrm{electrons}}{\mathrm{number}\;\mathrm{of}\;\mathrm{incident}\;\mathrm{photons}}=\frac{n\times N_{A}\times N}{\frac{P\times S\times t_{\mathrm{irr}}\times \lambda }{h\times c}}=\frac{N\times N_{A}\times n_{\mathrm{product}}\times h\times c}{P\times S\times t_{\mathrm{irr}}\times \lambda }$$

Where *N* is the number of electrons required for the generation of one molecule of the target product, *NA* is the Avogadro number (6.022 × 10^23^ mol^–1^), and *n*_product_ is the amount of the target product generated (mol).

### 10.1 Electron Transfer Process in Biotic-Abiotic Systems

As presented, the different biotic-abiotic artificial photosynthesis configurations require efficient electron transfer processes. These processes include electrical communication between enzymes and electrodes, enzymes and nanomaterials, the biotic internal electron transfer process to enzymes’ active sites, and more. As can be learned from the photosynthesis process, efficient electron transfer processes can be achieved by compatible redox centers with appropriate redox potentials and tailored electron transfer distances between those centers. When these parameters are optimized, efficient electron transfer rates can be gained. Furthermore, it is critical to design configurations that minimize back reactions, which hinder efficient electron transfer. These principles are essential in any electron transfer process, as proved in the Marcus theory. eq 9 presents the Marcus equation, which correlates the electron transfer rate with the distance between the electron donor and the electron acceptor.^398^ In many of the designed configurations presented, enzymes or photosynthetic proteins are electronically coupled with electrodes or nanomaterials. In most cases, protein scaffolds act as insulators that prevent and direct electron transfer processes as a result of short distances between the proteins’ active sites and the electrode. Exceptionals can be found in the case of hydrogenases, laccase, bilirubin oxidase, and FNRs. In order to overcome those limitations, MET processes can be used where redox mediators or redox polymers can act as relays to enable the electrical wiring of biotic elements.^399^ Advances in bioengineering, computational structure prediction, and the use of noncanonical amino acids open new ways to conjugate and orient proteins’ active sites or internal redox mediators with surfaces.^52,400–406^ Dictating the conjugation position prevents stochastic orientation, which can ultimately lead to short and efficient electron transfer processes.

9 $$k_{et}\propto e^{\left[-\beta \left(d-d_{0}\right)\right]}\cdot e^{\left[\frac{-{\left(\Delta G+\lambda \right)}^{2}}{4RT\lambda }\right]}$$

β = electron - coupling constant
Δ*G* = free energy change
λ = reorganization energy
*d* = distance separating the electron and donor.
*d*_0_ = van der Waals distance.

eq 9. The electron transfer Marcus theory equation. Where d is the distance between the electron donor and the electron acceptor, d_0_ is the van der Waals distance. Δ*G* is the free energy change, and λ is the reorganization energy associated with the electron transfer process.

While most of the configurations presented in this work are facilitated by electron transfer processes to isolated proteins, in some biohybrid systems, for example, in living cells or thylakoid membranes, different approaches are required. Membranes are insulators with a thickness above 2 nm that prevent any direct electron transfer process through hopping or tunneling mechanisms.^407^ Gram-negative bacteria often have a membrane reaching 4–7 nm; therefore, the DET process should not occur. To overcome these issues, redox relay structures and proteins have evolved to enable electrical communication of bacterial cells with their surroundings.^408,409^ Multiheme proteins such as MtrA and MtrC^205^ enable short-distance electron transfer processes on a microsecond time scale.^408,410^ This can be achieved by a hopping mechanism occurring between neighboring hemes with distances shorter than 2 nm. Using these internal electron transfer processes, a long-chain electron transfer process can be achieved.^411^ The heme orientation plays a critical role in the electron transfer process. More details of this fascinating electron transfer process through membranes can be found in recent reviews and research articles.^412–414^

## 11 Conclusion and Outlook

### 11.1 The Boundaries between the Biotic and the Abiotic Have Faded

Over the last few decades, blocks from different sets have been integrated to overcome the known limitations of materials science, biotechnology, (photo)­electrochemistry, and more. Once the methodologies for connecting the different blocks and characterizing them were developed and implemented, new possibilities emerged for developing applications using both the new and old blocks. The quest for renewable energy and artificial photosynthesis processes has been reignited in the past decade. Coupling light absorbers with extinction coefficients of a magnitude higher than those of the natural apparatus with living cells can improve the natural photoinduced reactions. A well-designed whole-cell interfaced configuration could potentially morph nonphotosynthetic bacteria into photosynthetic ones.

Clarivate citation report of the terms “biohybrid” or “biotic abiotic” exhibits exponential growth both in published papers and citations. An “old-school” organic chemist or biotechnology engineer can leverage their strong skill sets and knowledge to achieve further goals. Why limit the cofactor set to NAD­(P)­H? One can now design analogs^415–418^ that can shuttle through organic solvents^419,420^ or activate noncanonical enzymes.^421,422^ With advances in synthetic biology, these analogs can be activated *in vivo* to drive a specific enzymatic cascade without interfering with natural biochemical pathways. Proteins can be used as a “metal organic framework-like” system,^423^ or DNA can be used as a scaffold for photosensitizers,^424^ charge separation,^425,426^ or as an electronic chip.^132^

As presented, the development of biotic-abiotic configurations opens many new directions and will soon be available for real-world industrial applications. Critical development focuses on the photosynthetic apparatus mimicry, and indeed, significant advances have been made. Toward the full realization of biotic-abiotic configurations, several points should be addressed:

### 11.2 Sacrificial Electron Donor

While many creative and novel biohybrid configurations have been presented in the previous sections, these systems require sacrificial electron donors, such as TEOA, DTT, or ascorbic acid, for continuous activation. Derived from sustainability and economic reasons, these molecules cannot be used in practical applications. As shown, the generation of formic acid or H_2_ by biocatalytic, electrocatalytic, or photocatalytic reactions cannot be justified while consuming equal or greater chemical energy or incurring higher production costs. Another drawback is the release of CO_2_. To solve these issues, two approaches may be used:

- Incorporate water oxidation (bio)­catalysts.
- Utilized waste has a sacrificial electron donor.

On the one hand, following nature by incorporating a water-oxidation catalyst offers significant advantages; on the other hand, many (bio)­catalysts for H_2_ generation or CO_2_ reduction are adversely affected in the presence of oxygen. This issue can be addressed by smart design that limits oxygen diffusion to the photoreduction process, or by biotechnological engineering to develop a (bio)­catalyst selective for the desired process. The incorporation of incorporating oxygen-protecting layers could be a valid solution.^239,240^ Alternatively, exploiting waste material as a sacrificial electron donor offers significant advantages, as it does not lead to evolved oxygen (if water-oxidation competing reaction is suppressed),^287,308^ and it degrades undesired materials that could have value for recycling or valorization. Such processes can be coupled with oxygen-sensitive catalysts that facilitate the reduction of CO_2_ or H_2_O. Another critical aspect is technoeconomic validity. Green hydrogen is currently too expensive to compete with conventional hydrogen production methodologies. Its price ranges between 3 and 8$ per kg, while the aim is to reach 1$. By coupling a hydrogen generation system with the valorization of waste into fine chemicals or commodities, e.g., vanillin or pyruvate, the overall gain makes it economical. Pyruvate is currently in high demand for fermentation processes as the cultured meat industry grows, and its price range per kg is 1–11$.

Hence, producing pyruvate from PLA waste while generating H_2_ fuel could make it economical! Those directions should be the primary focus of researchers advancing this field. With that in mind, current maturation can be found mainly in the production of commodities. For example, New Iridium (Kemvera), offers light-activated organic photocatalysts for commodity production. The company offers commodities like acetic acid and ethyl acetate using biobased and CO_2_ feedstocks.^427^ Fronting fossil fuels commodities production is a challenging task due to the low price of the produced materials. On the other hand, producing pharmaceuticals may lead better cost-effectiveness margin due to the high price of such materials. A critical challenge toward that realization is device engineering. How can light-induced reactions fit industrial applications? Should it use LED light or sun irradiation? Can we combine both to limit dark cycles? Can we scale up current photoelectrochemical and photochemical configurations into industrial size reactors? This gap is not yet filled.

Another interesting direction to pursue is the activation of whole-cell-based biohybrids that can operate without the addition of an external electron donor. This can be achieved by coupling the photochemical reaction to internally produced donors. These could be generated by knockouts^428^ or added enzymatic processes.^429^ For example, by the addition of the thiosulfate reductase gene,^430^ sulfides can be accumulated internally and further used as an electron donor or for NP formation. In a different path, lactate production or H_2_O_2_ production by oxidases may lead to an appropriate electron donor. These directions should be further developed and coupled with whole-cell biohybrid systems.

### 11.3 Stability

A significant obstacle in catalysis is stability. Unlike what is commonly thought, this obstacle is shared across the different disciplines, including both biological and inorganic origin catalysts. In many cases, transient photocurrents or short-term fuel production are reported in leading journals, suggesting a bright future for renewable energy and artificial photosynthesis. Fundamental research is critical to generating new knowledge. Nonetheless, in H_2_ production or CO_2_ reduction, the research has matured to the point that it is mandatory to demonstrate stability for long-term activation,^238^ ranging from a few hours to days or more. If photoelectrochemical or electrochemical cells are utilized, one should clarify the activation barriers in terms of applied bias or the gained photocurrent in a bias-free configuration. In many cases, the applied potential is so high that electrical currents can be facilitated by the electrode surface toward oxidizing (or reducing) electron donor/acceptor molecules, which makes the light irradiation redundant!

Biohybrid configurations’ stability is limited both by the biological components and entities, and the abiotic elements. Often, the biohybrid photosensitizer, its cocatalyst, or the conjugated electrode is constructed using metal ions such as Cd, Cu, Ni, Co, Bi, Mo, Ti, Bi, and more. While some of these metal ions are available in living systems, their concentrations are extremely low, which limits toxicity. However, at higher concentrations required for internal or external NPs growth, or in conjugation with photo or electroactive electrodes, these ions can leach and act as enzyme inhibitors. Eventually, these ions can accumulate and fully inhibit the biological cascade in artificial configurations or bacteria growth and proper functioning. To limit their adverse effect, one should aim for alternating their oxidation state or accumulating in an insensitive compartment. These methodologies also occur naturally in bacterial species;^431–433^ however, they can be enhanced and improved by bioengineering and synthetic biology.^434,435^ In some cases, Metallothioneins (MTs), protein-rich in strong intercalation moieties such as cysteines, are used to remove heavy metals from the intercellular fluids and limit their negative effect.^436^ As presented, these challenges were mainly addressed by maintaining the environment with relatively low concentrations of toxic metal ions (>0.3 mM). For enhanced tolerance against those toxic elements, activated elements that can specifically react with the ions by reduction or complexation should be added or internally grown. Such elements (proteins, peptides, or externally added metal–organic complexes) that mimic the natural processes at metal ion toxic-tolerant bacteria should improve above the state of the art.^437–439^ As mentioned, the abiotic elements also suffer from instability and degradation in conjugation to biological components, aqueous solutions, natural and non-natural reducing agents, and photoirradiation. The addition of protecting layers, such as ligands (NPs) or thin films (electrodes), could greatly improve stability. The addition of sacrificial electron donors and limiting photoirradiation flux is crucial to limit back reactions, self-oxidation, and degradation of photosensitizers. Designing configurations that can internally generate electron donors could greatly improve any nanobio hybrid design. In general, such systems have great promise; however, maturation is required for real-world applications that may enable mass production of commodities and fuels.

With all achieved advances, without worldwide national policies supporting the shift to renewable energy, and specifically to artificial photosynthesis systems, most of these technologies will not advance beyond the pilot stage.^440^ The dependence on fossil fuels was challenged recently during the shortage of gas supply from Russia to Germany. This induced the use of alternative methods, including connecting the renewable energy resources with the smart grid.^441,442^ Currently, 23% of Germany’s energy can be attributed to renewable resources, although artificial photosynthesis technologies are not yet adapted. Governments and policymakers should not be led by energy crises or political hurdles to push toward renewable energy resources. Alternatively, we should continuously seek to reduce the dependency on fossil fuels, bearing in mind that the sun’s irradiation can support all the required energy on earth, as has been demonstrated for billions of years of photosynthesis. Utilizing artificial photosynthesis processes may add the missing block to enable sustainability while meeting the needs of modern life.^443,444^ An interesting demonstration of bench-to-application technology is H2Pro, a recently established startup company utilizing electrochemical cells to generate green H_2_ fuel. The company uses a technology developed at the Technion to decouple the generation of H_2_ and O_2_ in the electrolysis process.^21^ This important feature allows the direct generation and collection of green H_2_ without the use of an expensive membrane or separation techniques, which simplifies the required engineering of large-scale production and reduces costs. Coupling the technology directly to PV enables the generation of a photoinduced H_2_ fuel. Another interesting direction uses core–shell QDs with a cocatalyst for the generation of H_2_. Here, the technology developed at QD-SOL uses a sacrificial electron donor and light irradiation to produce fuel with a high efficiency. The maturation of the interfaced biotic-abiotic configuration is still hindered, and these processes are currently ambitious in terms of industry. Adapting the conditions that should fit the biological element, the abiotic element, and the industrial limitations and constraints is challenging. Efficient collection of the sun’s irradiation is another great challenge. Nevertheless, some scaled-up PEC devices have already been introduced.^445^ With the continuous development in the field, it is reasonable to suggest that higher TRLs should be reached in the near future.

In summary, the review summarizes the development of biotic-abiotic systems, the different approaches, and critical obstacles that need to be addressed in order to realize artificial photosynthesis, photoreforming, and light-induced energy generation systems.

## Abbreviations

ABPE: applied bias photon to current efficiency
ABTS: 2,2′ azino bis­(3 ethylbenzothiazoline 6 sulfonic acid)
ALD: atomic layer deposition
AQY: apparent quantum yield
ATP: adenosine triphosphate
BFC: biofuel cell
BOD: bilirubin oxidase
BHET: bis­(2 hydroxyethyl) terephthalate
C_*x*_Ny: carbon nitride
COF: covalent organic framework
DCPIP: 2,6 dichlorophenolindophenol
DET: direct electron transfer
DTT: dithiothreitol
EET: extracellular electron transfer
EQE: external quantum efficiency
FDCA: 2,5 furandicarboxylic acid
FDH: formate dehydrogenase
FAD: flavin adenine dinucleotide
FMN: flavin mononucleotide
FNR: ferredoxin–NADP^+^ reductase
FTO: fluorine doped tin oxide
HER: hydrogen evolution reaction
HMF: Five hydroxymethylfurfural
HRP: horseradish peroxidase
IPCE: incident photon to current efficiency
IQE: internal quantum efficiency
ITO: indium tin oxide
kET: electron transfer rate constant
MET: mediated electron transfer
MWCNT: multi walled carbon nanotube
MV: methyl viologen
N.A.: not available
N.Q.: not quantified
NAD^+^: nicotinamide adenine dinucleotide
NADH: reduced nicotinamide adenine dinucleotide
NADP^+^: nicotinamide adenine dinucleotide phosphate
NADPH: reduced nicotinamide adenine dinucleotide phosphate
NAD­(P)­H: reduced nicotinamide adenine dinucleotide (phosphate)
NHE: normal hydrogen electrode
NP(s): nanoparticle(s)
NR(s): nanorod(s)
OCV: open circuit voltage
OER: oxygen evolution reaction
ORR: oxygen reduction reaction
PBEC: photo bioelectrochemical cell
PBV: polybenzyl viologen
PEC: photoelectrochemical cell
PET: polyethylene terephthalate
PCE: power conversion efficiency
PLA: polylactic acid
PS: photosensitizer
PSI: photosystem I
PSII: photosystem II
QD(s): quantum dot(s)
QE: quantum efficiency
QY: quantum yield
RHE: reversible hydrogen electrode
SAM: self-assembled monolayer
SED: sacrificial electron donor
SHE: standard hydrogen electrode
SP1: stable protein 1
STH: solar to hydrogen efficiency
TEOA: triethanolamine
TEMPO: 2,2,6,6 tetramethylpiperidine 1 oxyl
TOF: turnover frequency
TRL: technology readiness level

## References

[1] Nelson N., Ben-Shem A. (2004). The Complex Architecture of Oxygenic Photosynthesis. Nat. Rev. Mol. Cell Biol..

[2] Blankenship R. E., Tiede D. M., Barber J., Brudvig G. W., Fleming G., Ghirardi M., Gunner M. R., Junge W., Kramer D. M., Melis A., Moore T. A., Moser C. C., Nocera D. G., Nozik A. J., Ort D. R., Parson W. W., Prince R. C., Sayre R. T. (2011). Comparing Photosynthetic
and Photovoltaic Efficiencies
and Recognizing the Potential for Improvement. Science.

[3] Walter M. G., Warren E. L., McKone J. R., Boettcher S. W., Mi Q., Santori E. A., Lewis N. S. (2010). Solar Water Splitting Cells. Chem. Rev..

[4] Grätzel M. (2001). Photoelectrochemical
Cells. Nature.

[5] Bard A. J. (1979). Photoelectrochemistry
and Heterogeneous Photo-Catalysis at Semiconductors. J. Photochem..

[6] Feher G., Allen J. P., Okamura M. Y., Rees D. C. (1989). Structure and Function
of Bacterial Photosynthetic Reaction Centres. Nature.

[7] Wang H., Li J., Feng Y., He D., Fan X., Wang B., Cai Z., Zeng C., Xiao K. (2026). Cross-Scale Design of Abiotic-Biotic
Interfaces for Semi-Artificial Photosynthesis. Chem. Sci..

[8] Umena Y., Kawakami K., Shen J.-R., Kamiya N. (2011). Crystal Structure of
Oxygen-Evolving Photosystem II at a Resolution of 1.9 Å. Nature.

[9] Zhang C., Chen C., Dong H., Shen J.-R., Dau H., Zhao J. (2015). A Synthetic Mn4Ca-Cluster
Mimicking the Oxygen-Evolving Center of
Photosynthesis. Science.

[10] Maayan G., Gluz N., Christou G. (2018). A Bioinspired
Soluble Manganese Cluster
as a Water Oxidation Electrocatalyst with Low Overpotential. Nat. Catal.

[11] Cohen Y., Gluz N., Bamany S., Maayan G., Yehezkeli O. (2020). Layer by Layer
Assembly of a Bio-Inspired Manganese Cluster for Electrocatalytic
Water Oxidation. J. Catal..

[12] Blakemore J. D., Crabtree R. H., Brudvig G. W. (2015). Molecular
Catalysts for Water Oxidation. Chem. Rev..

[13] Concepcion J. J., Jurss J. W., Brennaman M. K., Hoertz P. G., Patrocinio A. O. T., Murakami Iha N. Y., Templeton J. L., Meyer T. J. (2009). Making Oxygen with Ruthenium Complexes. Acc. Chem. Res..

[14] Duan L., Bozoglian F., Mandal S., Stewart B., Privalov T., Llobet A., Sun L. (2012). A Molecular Ruthenium Catalyst with
Water-Oxidation Activity Comparable to That of Photosystem II. Nature Chem..

[15] Noll N., Krause A.-M., Beuerle F., Würthner F. (2022). Enzyme-like
Water Preorganization in a Synthetic Molecular Cleft for Homogeneous
Water Oxidation Catalysis. Nat. Catal.

[16] Gorlin Y., Jaramillo T. F. (2010). A Bifunctional Nonprecious Metal Catalyst for Oxygen
Reduction and Water Oxidation. J. Am. Chem.
Soc..

[17] Youngblood W. J., Lee S.-H. A., Kobayashi Y., Hernandez-Pagan E. A., Hoertz P. G., Moore T. A., Moore A. L., Gust D., Mallouk T. E. (2009). Photoassisted Overall Water Splitting in a Visible
Light-Absorbing Dye-Sensitized Photoelectrochemical Cell. J. Am. Chem. Soc..

[18] Kanan M. W., Yano J., Surendranath Y., Dincǎ M., Yachandra V. K., Nocera D. G. (2010). Structure and Valency
of a Cobalt-Phosphate
Water Oxidation Catalyst Determined by in Situ X-Ray Spectroscopy. J. Am. Chem. Soc..

[19] Kanan M. W., Nocera D. G. (2008). In Situ Formation
of an Oxygen-Evolving Catalyst in
Neutral Water Containing Phosphate and Co2+. Science.

[20] Pijpers J. J. H., Winkler M. T., Surendranath Y., Buonassisi T., Nocera D. G. (2011). Light-Induced Water Oxidation at Silicon Electrodes
Functionalized with a Cobalt Oxygen-Evolving Catalyst. Proc. Natl. Acad. Sci. U. S. A..

[21] Dotan H., Landman A., Sheehan S. W., Malviya K. D., Shter G. E., Grave D. A., Arzi Z., Yehudai N., Halabi M., Gal N., Hadari N., Cohen C., Rothschild A., Grader G. S. (2019). Decoupled Hydrogen
and Oxygen Evolution by a Two-Step
Electrochemical-Chemical Cycle for Efficient Overall Water Splitting. Nat. Energy.

[22] Nishiyama H., Yamada T., Nakabayashi M., Maehara Y., Yamaguchi M., Kuromiya Y., Nagatsuma Y., Tokudome H., Akiyama S., Watanabe T., Narushima R., Okunaka S., Shibata N., Takata T., Hisatomi T., Domen K. (2021). Photocatalytic Solar
Hydrogen Production from Water on a 100-M2 Scale. Nature.

[23] Fromme P., Jordan P., Krauß N. (2001). Structure
of Photosystem I. Biochimica et Biophysica Acta
(BBA) - Bioenergetics.

[24] Jordan P., Fromme P., Witt H. T., Klukas O., Saenger W., Krauß N. (2001). Three-Dimensional
Structure of Cyanobacterial Photosystem
I at 2.5 Å Resolution. Nature.

[25] Millsaps J.
F., Bruce B. D., Lee J. W., Greenbaum E. (2001). Nanoscale
Photosynthesis: Photocatalytic Production of Hydrogen by Platinized
Photosystem I Reaction Centers. Photochem. Photobiol..

[26] Grimme R. A., Lubner C. E., Bryant D. A., Golbeck J. H. (2008). Photosystem I/Molecular
Wire/Metal Nanoparticle Bioconjugates for the Photocatalytic Production
of H2. J. Am. Chem. Soc..

[27] Gorka M., Schartner J., van der Est A., Rögner M., Golbeck J. H. (2014). Light-Mediated Hydrogen
Generation in Photosystem I:
Attachment of a Naphthoquinone-Molecular Wire-Pt Nanoparticle to the
A1A and A1B Sites. Biochemistry.

[28] Utschig L.
M., Dimitrijevic N. M., Poluektov O. G., Chemerisov S. D., Mulfort K. L., Tiede D. M. (2011). Photocatalytic
Hydrogen Production
from Noncovalent Biohybrid Photosystem I/Pt Nanoparticle Complexes. J. Phys. Chem. Lett..

[29] Utschig L. M., Soltau S. R., Tiede D. M. (2015). Light-Driven
Hydrogen Production
from Photosystem I-Catalyst Hybrids. Curr. Opin.
Chem. Biol..

[30] Iwuchukwu I. J., Vaughn M., Myers N., O’Neill H., Frymier P., Bruce B. D. (2010). Self-Organized Photosynthetic Nanoparticle
for Cell-Free Hydrogen Production. Nat. Nanotechnol..

[31] Gisriel C. J., Malavath T., Qiu T., Menzel J. P., Batista V. S., Brudvig G. W., Utschig L. M. (2024). Structure of a Biohybrid Photosystem
I-Platinum Nanoparticle Solar Fuel Catalyst. Nat. Commun..

[32] Nagakawa H., Takeuchi A., Takekuma Y., Noji T., Kawakami K., Kamiya N., Nango M., Furukawa R., Nagata M. (2019). Efficient
Hydrogen Production Using Photosystem I Enhanced by Artificial Light
Harvesting Dye. Photochem. Photobiol. Sci..

[33] Govorov A.
O., Carmeli I. (2007). Hybrid Structures
Composed of Photosynthetic System
and Metal Nanoparticles: Plasmon Enhancement Effect. Nano Lett..

[34] Frolov L., Wilner O., Carmeli C., Carmeli I. (2008). Fabrication of Oriented
Multilayers of Photosystem I Proteins on Solid Surfaces by Auto-Metallization. Adv. Mater..

[35] Kim Y., Shin D., Chang W. J., Jang H. L., Lee C. W., Lee H.-E., Nam K. T. (2015). Hybrid Z-Scheme Using Photosystem
I and BiVO4 for Hydrogen Production. Adv. Funct.
Mater..

[36] Lima-Melo, Y. ; Kılıç, M. ; Aro, E.-M. ; Gollan, P. J. Photosystem I Inhibition, Protection and Signalling: Knowns and Unknowns. Front. Plant Sci. 2021, 12. 10.3389/fpls.2021.791124. PMC8671627 34925429

[37] Teodor A. H., Bruce B. D. (2020). Putting Photosystem I to Work: Truly
Green Energy. Trends Biotechnol..

[38] Yehezkeli O., Tel-Vered R., Michaeli D., Willner I., Nechushtai R. (2014). Photosynthetic
Reaction Center-Functionalized Electrodes for Photo-Bioelectrochemical
Cells. Photosynth Res..

[39] Badura A., Kothe T., Schuhmann W., Rögner M. (2011). Wiring Photosynthetic
Enzymes to Electrodes. Energy Environ. Sci..

[40] Ham M.-H., Choi J. H., Boghossian A. A., Jeng E. S., Graff R. A., Heller D. A., Chang A. C., Mattis A., Bayburt T. H., Grinkova Y. V., Zeiger A. S., Van Vliet K. J., Hobbie E. K., Sligar S. G., Wraight C. A., Strano M. S. (2010). Photoelectrochemical
Complexes for Solar Energy Conversion That Chemically and Autonomously
Regenerate. Nat. Chem..

[41] Lebedev N., Trammell S. A., Spano A., Lukashev E., Griva I., Schnur J. (2006). Conductive Wiring of
Immobilized Photosynthetic Reaction
Center to Electrode by Cytochrome c. J. Am.
Chem. Soc..

[42] Trammell S. A., Wang L., Zullo J. M., Shashidhar R., Lebedev N. (2004). Orientated Binding of Photosynthetic Reaction Centers
on Gold Using Ni-NTA Self-Assembled Monolayers. Biosens. Bioelectron..

[43] Kondo M., Nakamura Y., Fujii K., Nagata M., Suemori Y., Dewa T., Iida K., Gardiner A. T., Cogdell R. J., Nango M. (2007). Self-Assembled Monolayer
of Light-Harvesting Core Complexes from
Photosynthetic Bacteria on a Gold Electrode Modified with Alkanethiols. Biomacromolecules.

[44] Yehezkeli O., Tel-Vered R., Michaeli D., Nechushtai R., Willner I. (2013). Photosystem I (PSI)/Photosystem
II (PSII)-Based Photo-Bioelectrochemical
Cells Revealing Directional Generation of Photocurrents. Small.

[45] Carmeli I., Frolov L., Carmeli C., Richter S. (2007). Photovoltaic Activity
of Photosystem I-Based Self-Assembled Monolayer. J. Am. Chem. Soc..

[46] Faulkner C. J., Lees S., Ciesielski P. N., Cliffel D. E., Jennings G. K. (2008). Rapid Assembly
of Photosystem I Monolayers on Gold Electrodes. Langmuir.

[47] Ciesielski P. N., Scott A. M., Faulkner C. J., Berron B. J., Cliffel D. E., Jennings G. K. (2008). Functionalized Nanoporous Gold Leaf Electrode Films
for the Immobilization of Photosystem I. ACS
Nano.

[48] Ciesielski P. N., Faulkner C. J., Irwin M. T., Gregory J. M., Tolk N. H., Cliffel D. E., Jennings G. K. (2010). Enhanced Photocurrent Production
by Photosystem I Multilayer Assemblies. Adv.
Funct. Mater..

[49] Yehezkeli O., Wilner O. I., Tel-Vered R., Roizman-Sade D., Nechushtai R., Willner I. (2010). Generation of Photocurrents by Bis-Aniline-Cross-Linked
Pt Nanoparticle/Photosystem I Composites on Electrodes. J. Phys. Chem. B.

[50] Badura A., Guschin D., Kothe T., Kopczak M. J., Schuhmann W., Rögner M. (2011). Photocurrent
Generation by Photosystem 1 Integrated
in Crosslinked Redox Hydrogels. Energy Environ.
Sci..

[51] Terasaki N., Yamamoto N., Hiraga T., Yamanoi Y., Yonezawa T., Nishihara H., Ohmori T., Sakai M., Fujii M., Tohri A., Iwai M., Inoue Y., Yoneyama S., Minakata M., Enami I. (2009). Plugging a Molecular Wire into Photosystem
I: Reconstitution of the Photoelectric Conversion System on a Gold
Electrode. Angew. Chem., Int. Ed..

[52] Mershin, A. ; Matsumoto, K. ; Kaiser, L. ; Yu, D. ; Vaughn, M. ; Nazeeruddin, Md. K. ; Bruce, B. D. ; Graetzel, M. ; Zhang, S. Self-Assembled Photosystem-I Biophotovoltaics on Nanostructured TiO2 and ZnO. Sci. Rep. 2012, 2. 10.1038/srep00234. PMC3270499 22355747

[53] Manocchi A.
K., Baker D. R., Pendley S. S., Nguyen K., Hurley M. M., Bruce B. D., Sumner J. J., Lundgren C. A. (2013). Photocurrent Generation
from Surface Assembled Photosystem I on Alkanethiol Modified Electrodes. Langmuir.

[54] Kothe T., Pöller S., Zhao F., Fortgang P., Rögner M., Schuhmann W., Plumeré N. (2014). Engineered Electron-Transfer Chain
in Photosystem 1 Based Photocathodes Outperforms Electron-Transfer
Rates in Natural Photosynthesis. Chem. Eur.
J..

[55] Herzallh N. S., Cohen Y., Mukha D., Neumann E., Michaeli D., Nechushtai R., Yehezkeli O. (2020). Photosynthesis Z-Scheme Biomimicry:
Photosystem I/BiVO4 Photo-Bioelectrochemical Cell for Donor-Free Bias-Free
Electrical Power Generation. Biosens. Bioelectron..

[56] Kothe T., Plumeré N., Badura A., Nowaczyk M. M., Guschin D. A., Rögner M., Schuhmann W. (2013). Combination of A Photosystem 1-Based
Photocathode and a Photosystem 2-Based Photoanode to a Z-Scheme Mimic
for Biophotovoltaic Applications. Angew. Chem.,
Int. Ed..

[57] Buesen D., Hoefer T., Zhang H., Plumeré N. (2019). A Kinetic
Model for Redox-Active Film Based Biophotoelectrodes. Faraday Discuss..

[58] Wang P., Frank A., Zhao F., Nowaczyk M. M., Conzuelo F., Schuhmann W. (2023). A Biomimetic
Assembly of Folded Photosystem I Monolayers
for an Improved Light Utilization in Biophotovoltaic Devices. Bioelectrochemistry.

[59] Zhao F., Wang P., Ruff A., Hartmann V., Zacarias S., Pereira I. A. C., Nowaczyk M., Rögner M., Conzuelo F., Schuhmann W. (2019). A Photosystem I Monolayer with Anisotropic
Electron Flow Enables Z-Scheme like Photosynthetic Water Splitting. Energy Environ. Sci..

[60] Wang P., Zhao F., Frank A., Zerria S., Lielpetere A., Ruff A., Nowaczyk M. M., Schuhmann W., Conzuelo F. (2021). Rational Design of a Photosystem
I Photoanode for the
Fabrication of Biophotovoltaic Devices. Adv.
Energy Mater..

[61] Wang P., Frank A., Zhao F., Szczesny J., Junqueira J. R. C., Zacarias S., Ruff A., Nowaczyk M. M., Pereira I. A. C., Rögner M., Conzuelo F., Schuhmann W. (2021). Closing the
Gap for Electronic Short-Circuiting: Photosystem I Mixed Monolayers
Enable Improved Anisotropic Electron Flow in Biophotovoltaic Devices. Angew. Chem., Int. Ed..

[62] Zhao F., Conzuelo F., Hartmann V., Li H., Nowaczyk M. M., Plumeré N., Rögner M., Schuhmann W. (2015). Light Induced
H2 Evolution from a Biophotocathode Based on Photosystem 1 - Pt Nanoparticles
Complexes Integrated in Solvated Redox Polymers Films. J. Phys. Chem. B.

[63] Wang P., Frank A., Appel J., Boehm M., Strabel N., Nowaczyk M. M., Schuhmann W., Conzuelo F., Gutekunst K. (2023). In Vivo Assembly
of Photosystem I-Hydrogenase Chimera for In Vitro PhotoH2 Production. Adv. Energy Mater..

[64] Zhao F., Hardt S., Hartmann V., Zhang H., Nowaczyk M. M., Rögner M., Plumeré N., Schuhmann W., Conzuelo F. (2018). Light-Induced Formation of Partially Reduced Oxygen
Species Limits the Lifetime of Photosystem 1-Based Biocathodes. Nat. Commun..

[65] Zhao F., Ruff A., Rögner M., Schuhmann W., Conzuelo F. (2019). Extended Operational Lifetime of
a Photosystem-Based
Bioelectrode. J. Am. Chem. Soc..

[66] Renger G. (2012). Mechanism
of Light Induced Water Splitting in Photosystem II of Oxygen Evolving
Photosynthetic Organisms. Biochimica et Biophysica
Acta (BBA) - Bioenergetics.

[67] Vass I., Styring S., Hundal T., Koivuniemi A., Aro E., Andersson B. (1992). Reversible
and Irreversible Intermediates during Photoinhibition
of Photosystem II: Stable Reduced QA Species Promote Chlorophyll Triplet
Formation. Proc. Natl. Acad. Sci. U. S. A..

[68] Hartmann V., Harris D., Bobrowski T., Ruff A., Frank A., Pomorski T. G., Rögner M., Schuhmann W., Adir N., Nowaczyk M. M. (2020). Improved Quantum Efficiency in an
Engineered Light Harvesting/Photosystem II Super-Complex for High
Current Density Biophotoanodes. J. Mater. Chem.
A.

[69] Noji T., Suzuki H., Gotoh T., Iwai M., Ikeuchi M., Tomo T., Noguchi T. (2011). Photosystem II-Gold Nanoparticle
Conjugate as a Nanodevice for the Development of Artificial Light-Driven
Water-Splitting Systems. J. Phys. Chem. Lett..

[70] Feng X., Jia Y., Cai P., Fei J., Li J. (2016). Coassembly of Photosystem
II and ATPase as Artificial Chloroplast for Light-Driven ATP Synthesis. ACS Nano.

[71] Lee K. Y., Park S.-J., Lee K. A., Kim S.-H., Kim H., Meroz Y., Mahadevan L., Jung K.-H., Ahn T. K., Parker K. K., Shin K. (2018). Photosynthetic Artificial Organelles
Sustain and Control ATP-Dependent Reactions in a Protocellular System. Nat. Biotechnol..

[72] Miller T. E., Beneyton T., Schwander T., Diehl C., Girault M., McLean R., Chotel T., Claus P., Cortina N. S., Baret J.-C., Erb T. J. (2020). Light-Powered
CO2 Fixation in a Chloroplast
Mimic with Natural and Synthetic Parts. Science.

[73] Gaut N. J., Adamala K. P. (2020). Toward Artificial Photosynthesis. Science.

[74] Voloshin R. A., Shumilova S. M., Zadneprovskaya E. V., Zharmukhamedov S. K., Alwasel S., Hou H. J. M., Allakhverdiev S. I. (2022). Photosystem
II in Bio-Photovoltaic Devices. Photosynthetica.

[75] McEvoy J. P., Brudvig G. W. (2006). Water-Splitting Chemistry of Photosystem II. Chem. Rev..

[76] Miyachi M., Ikehira S., Nishiori D., Yamanoi Y., Yamada M., Iwai M., Tomo T., Allakhverdiev S. I., Nishihara H. (2017). Photocurrent Generation of Reconstituted
Photosystem
II on a Self-Assembled Gold Film. Langmuir.

[77] Terasaki N., Iwai M., Yamamoto N., Hiraga T., Yamada S., Inoue Y. (2008). Photocurrent Generation Properties of Histag-Photosystem II Immobilized
on Nanostructured Gold Electrode. Thin Solid
Films.

[78] Cai P., Feng X., Fei J., Li G., Li J., Huang J., Li J. (2015). Co-Assembly of Photosystem II/Reduced
Graphene Oxide Multilayered Biohybrid Films for Enhanced Photocurrent. Nanoscale.

[79] Kato M., Cardona T., Rutherford A. W., Reisner E. (2013). Covalent Immobilization
of Oriented Photosystem II on a Nanostructured Electrode for Solar
Water Oxidation. J. Am. Chem. Soc..

[80] Kato M., Cardona T., Rutherford A. W., Reisner E. (2012). Photoelectrochemical
Water Oxidation with Photosystem II Integrated in a Mesoporous Indium-Tin
Oxide Electrode. J. Am. Chem. Soc..

[81] Sokol K. P., Mersch D., Hartmann V., Zhang J. Z., Nowaczyk M. M., Rögner M., Ruff A., Schuhmann W., Plumeré N., Reisner E. (2016). Rational Wiring of Photosystem II
to Hierarchical Indium Tin Oxide Electrodes Using Redox Polymers. Energy Environ. Sci..

[82] Maly J., Masojidek J., Masci A., Ilie M., Cianci E., Foglietti V., Vastarella W., Pilloton R. (2005). Direct Mediatorless
Electron Transport between the Monolayer of Photosystem II and Poly­(Mercapto-*p*-Benzoquinone) Modified Gold Electrode—New Design
of Biosensor for Herbicide Detection. Biosens.
Bioelectron..

[83] Yehezkeli O., Tel-Vered R., Wasserman J., Trifonov A., Michaeli D., Nechushtai R., Willner I. (2012). Integrated Photosystem II-Based Photo-Bioelectrochemical
Cells. Nat. Commun..

[84] Mersch D., Lee C.-Y., Zhang J. Z., Brinkert K., Fontecilla-Camps J. C., Rutherford A. W., Reisner E. (2015). Wiring of Photosystem II to Hydrogenase
for Photoelectrochemical Water Splitting. J.
Am. Chem. Soc..

[85] Sokol K. P., Robinson W. E., Oliveira A. R., Warnan J., Nowaczyk M. M., Ruff A., Pereira I. A. C., Reisner E. (2018). Photoreduction of CO2
with a Formate Dehydrogenase Driven by Photosystem II Using a Semi-Artificial
Z-Scheme Architecture. J. Am. Chem. Soc..

[86] Zhang J. Z., Reisner E. (2020). Advancing Photosystem II Photoelectrochemistry for
Semi-Artificial Photosynthesis. Nat. Rev. Chem..

[87] Fang X., Sokol K. P., Heidary N., Kandiel T. A., Zhang J. Z., Reisner E. (2019). Structure-Activity Relationships of Hierarchical Three-Dimensional
Electrodes with Photosystem II for Semiartificial Photosynthesis. Nano Lett..

[88] Xuan M., Li J. (2021). Photosystem II-Based
Biomimetic Assembly for Enhanced Photosynthesis. Natl. Sci. Rev..

[89] Zhang J. Z., Sokol K. P., Paul N., Romero E., van Grondelle R., Reisner E. (2016). Competing Charge Transfer Pathways at the Photosystem
II-Electrode Interface. Nat. Chem. Biol..

[90] Nioradze N., Ciornii D., Kölsch A., Göbel G., Khoshtariya D. E., Zouni A., Lisdat F. (2021). Electrospinning for
Building 3D Structured Photoactive Biohybrid Electrodes. Bioelectrochemistry.

[91] Ciornii D., Kölsch A., Zouni A., Lisdat F. (2019). A Precursor-Approach
in Constructing 3D ITO Electrodes for the Improved Performance of
Photosystem I-Cyt c Photobioelectrodes. Nanoscale.

[92] Morlock S., Subramanian S. K., Zouni A., Lisdat F. (2022). Bio-Inorganic Hybrid
Structures for Direct Electron Transfer to Photosystem I in Photobioelectrodes. Biosens. Bioelectron..

[93] Chen X., Lawrence J. M., Wey L. T., Schertel L., Jing Q., Vignolini S., Howe C. J., Kar-Narayan S., Zhang J. Z. (2022). 3D-Printed Hierarchical Pillar Array Electrodes for
High-Performance Semi-Artificial Photosynthesis. Nat. Mater..

[94] Riedel M., Wersig J., Ruff A., Schuhmann W., Zouni A., Lisdat F. (2019). A Z-Scheme-Inspired Photobioelectrochemical
H2O/O2 Cell with a 1 V Open-Circuit Voltage Combining Photosystem
II and PbS Quantum Dots. Angew. Chem., Int.
Ed..

[95] van
Mieghem F., Brettel K., Hillman B., Kamlowski A., Rutherford A. W., Schlodder E. (1995). Charge Recombination Reactions in
Photosystem II. 1. Yields, Recombination Pathways, and Kinetics of
the Primary Pair. Biochemistry.

[96] Jiang J., Spies J. A., Swierk J. R., Matula A. J., Regan K. P., Romano N., Brennan B. J., Crabtree R. H., Batista V. S., Schmuttenmaer C. A., Brudvig G. W. (2018). Direct Interfacial Electron Transfer
from High-Potential Porphyrins into Semiconductor Surfaces: A Comparison
of Linkers and Anchoring Groups. J. Phys. Chem.
C.

[97] Jaramillo A., Satta A., Pinto F., Faraloni C., Zittelli G. C., Silva Benavides A. M., Torzillo G., Schumann C., Méndez J. F., Berggren G., Lindblad P., Parente M., Esposito S., Diano M. (2025). Outlook on Synthetic Biology-Driven
Hydrogen Production: Lessons
from Algal Photosynthesis Applied to Cyanobacteria. Energy Fuels.

[98] Elman T., Ho T. T. H., Milrad Y., Hippler M., Yacoby I. (2022). Enhanced Chloroplast-Mitochondria
Crosstalk Promotes Ambient Algal-H2 Production. CR-PHYS-SC.

[99] Kanygin A., Milrad Y., Thummala C., Reifschneider K., Baker P., Marco P., Yacoby I., Redding K. E. (2020). Rewiring
Photosynthesis: A Photosystem I-Hydrogenase Chimera That Makes H2in
Vivo. Energy Environ. Sci..

[100] Materna K. L., Jiang J., Regan K. P., Schmuttenmaer C. A., Crabtree R. H., Brudvig G. W. (2017). Optimization of
Photoanodes for Photocatalytic
Water Oxidation by Combining a Heterogenized Iridium Water-Oxidation
Catalyst with a High-Potential Porphyrin Photosensitizer. ChemSusChem.

[101] Chen S., Qi Y., Li C., Domen K., Zhang F. (2018). Surface Strategies for Particulate
Photocatalysts toward Artificial
Photosynthesis. Joule.

[102] Yoshino S., Takayama T., Yamaguchi Y., Iwase A., Kudo A. (2022). CO2 Reduction Using Water as an Electron
Donor over Heterogeneous Photocatalysts Aiming at Artificial Photosynthesis. Acc. Chem. Res..

[103] Xiao M., Pelicano C. M., Antonietti M. (2025). Promoted Photosynthesis
in Seawater by Carbon Nitride and Related Systems. ACS Catal..

[104] Yehezkeli O., Bedford N. M., Park E., Ma K., Cha J. N. (2016). Semiconductor-Based, Solar-Driven Photochemical Cells
for Fuel Generation from Carbon Dioxide in Aqueous Solutions. ChemSusChem.

[105] Gao F., Liu G., Chen A., Hu Y., Wang H., Pan J., Feng J., Zhang H., Wang Y., Min Y., Gao C., Xiong Y. (2023). Artificial
Photosynthetic Cells with Biotic-Abiotic
Hybrid Energy Modules for Customized CO2 Conversion. Nat. Commun..

[106] Zhi T., Fu T., Zhan H., Zhou R., Yang M., Gao C., Wang P., Zhan S., Zhou Q. (2025). Photosynthetic Biohybrid
Systems: A Promising Approach for Energy and Environmental Applications. Environ. Sci. Technol..

[107] Song W., Zhang X., Li W., Li B., Liu B. (2025). Engineering Biotic-Abiotic Hybrid Systems for Solar-to-Chemical
Conversion. Chem..

[108] Robertson I. L. B., Zhang H., Reisner E., Butt J. N., Jeuken L. J. C. (2024). Engineering of Bespoke Photosensitiser-Microbe
Interfaces
for Enhanced Semi-Artificial Photosynthesis. Chem. Sci..

[109] Zhang W., Xiong C., Chen P., Fu B., Mao X. (2025). Elucidating
Energy Conversion Pathways at Biotic/Abiotic Interfaces
in Microbe-Semiconductor Hybrids. J. Am. Chem.
Soc..

[110] Liu G., Gao F., Gao C., Xiong Y. (2021). Bioinspiration toward
Efficient Photosynthetic Systems: From Biohybrids to Biomimetics. Chem. Catalysis.

[111] Fang X., Kalathil S., Reisner E. (2020). Semi-Biological
Approaches
to Solar-to-Chemical Conversion. Chem. Soc.
Rev..

[112] Kornienko N., Zhang J. Z., Sakimoto K. K., Yang P., Reisner E. (2018). Interfacing
Nature’s Catalytic Machinery with
Synthetic Materials for Semi-Artificial Photosynthesis. Nat. Nanotechnol..

[113] Wang Q., Domen K. (2020). Particulate Photocatalysts
for Light-Driven
Water Splitting: Mechanisms, Challenges, and Design Strategies. Chem. Rev..

[114] Chen, Y. ; Xu, B. ; Yao, R. ; Chen, C. ; Zhang, C. Mimicking the Oxygen-Evolving Center in Photosynthesis. Front. Plant Sci. 2022, 13. 10.3389/fpls.2022.929532. PMC9302449 35874004

[115] Chen Y., Su Y., Han J., Chen C., Fan H., Zhang C. (2024). Synthetic Mn3Ce2O5-Cluster Mimicking the Oxygen-Evolving
Center in Photosynthesis. ChemSusChem.

[116] Yao R., Li Y., Chen Y., Xu B., Chen C., Zhang C. (2021). Rare-Earth Elements Can Structurally and Energetically Replace the
Calcium in a Synthetic Mn4CaO4-Cluster Mimicking the Oxygen-Evolving
Center in Photosynthesis. J. Am. Chem. Soc..

[117] Zhang C., Chen C., Dong H., Shen J.-R., Dau H., Zhao J. (2015). A Synthetic Mn4Ca-Cluster Mimicking the Oxygen-Evolving
Center of Photosynthesis. Science.

[118] Kanady J. S., Lin P.-H., Carsch K. M., Nielsen R. J., Takase M. K., Goddard W. A. I., Agapie T. (2014). Toward Models for the
Full Oxygen-Evolving Complex of Photosystem II by Ligand Coordination
To Lower the Symmetry of the Mn3CaO4 Cubane: Demonstration That Electronic
Effects Facilitate Binding of a Fifth Metal. J. Am. Chem. Soc..

[119] Zhai Q., Xie S., Fan W., Zhang Q., Wang Y., Deng W., Wang Y. (2013). Photocatalytic
Conversion
of Carbon Dioxide with Water into Methane: Platinum and Copper­(I)
Oxide Co-Catalysts with a Core-Shell Structure. Angew. Chem., Int. Ed..

[120] Liao G., Gong Y., Zhang L., Gao H., Yang G.-J., Fang B. (2019). Semiconductor Polymeric Graphitic
Carbon Nitride Photocatalysts: The “Holy Grail” for
the Photocatalytic Hydrogen Evolution Reaction under Visible Light. Energy Environ. Sci..

[121] Wang X., Maeda K., Thomas A., Takanabe K., Xin G., Carlsson J. M., Domen K., Antonietti M. (2009). A Metal-Free
Polymeric Photocatalyst for Hydrogen Production from Water under Visible
Light. Nat. Mater..

[122] Takata T., Jiang J., Sakata Y., Nakabayashi M., Shibata N., Nandal V., Seki K., Hisatomi T., Domen K. (2020). Photocatalytic Water Splitting with
a Quantum Efficiency of Almost
Unity. Nature.

[123] Nishiyama H., Yamada T., Nakabayashi M., Maehara Y., Yamaguchi M., Kuromiya Y., Nagatsuma Y., Tokudome H., Akiyama S., Watanabe T., Narushima R., Okunaka S., Shibata N., Takata T., Hisatomi T., Domen K. (2021). Photocatalytic Solar Hydrogen Production from Water on a 100-M2 Scale. Nature.

[124] Tan Y.-X., Zhang X., Wang Y., Yao J. (2024). Molecular
Assembly of Functional Motifs for Artificial Photosynthesis. Acc. Mater. Res..

[125] Kärkäs M. D., Verho O., Johnston E. V., Åkermark B. (2014). Artificial Photosynthesis: Molecular
Systems for Catalytic
Water Oxidation. Chem. Rev..

[126] Gust D., Moore T. A. (1989). Mimicking Photosynthesis. Science.

[127] Zhang B., Sun L. (2019). Artificial Photosynthesis:
Opportunities
and Challenges of Molecular Catalysts. Chem.
Soc. Rev..

[128] Seeman N. C., Sleiman H. F. (2018). DNA Nanotechnology. Nat. Rev.
Mater..

[129] Seeman N. C. (1982). Nucleic
Acid Junctions and Lattices. J. Theor. Biol..

[130] Rothemund P. W. K. (2006). Folding
DNA to Create Nanoscale Shapes and Patterns. Nature.

[131] Tel-Vered R., Yehezkeli O., Yildiz H. B., Wilner O. I., Willner I. (2008). Photoelectrochemistry with Ordered CdS Nanoparticle/Relay
or Photosensitizer/Relay Dyads on DNA Scaffolds. Angew. Chem., Int. Ed..

[132] Hung A. M., Micheel C. M., Bozano L. D., Osterbur L. W., Wallraff G. M., Cha J. N. (2010). Large-Area Spatially
Ordered Arrays
of Gold Nanoparticles Directed by Lithographically Confined DNA Origami. Nat. Nanotechnol..

[133] Wilner O. I., Orbach R., Henning A., Teller C., Yehezkeli O., Mertig M., Harries D., Willner I. (2011). Self-Assembly
of DNA Nanotubes with Controllable Diameters. Nat. Commun..

[134] Zhou X., Mandal S., Jiang S., Lin S., Yang J., Liu Y., Whitten D. G., Woodbury N. W., Yan H. (2019). Efficient Long-Range,
Directional Energy Transfer through DNA-Templated
Dye Aggregates. J. Am. Chem. Soc..

[135] Grossi G., Dalgaard Ebbesen Jepsen M., Kjems J., Andersen E. S. (2017). Control
of Enzyme Reactions by a Reconfigurable DNA
Nanovault. Nat. Commun..

[136] Piperberg G., Wilner O. I., Yehezkeli O., Tel-Vered R., Willner I. (2009). Control of Bioelectrocatalytic Transformations
on DNA Scaffolds. J. Am. Chem. Soc..

[137] Wilner O.
I., Weizmann Y., Gill R., Lioubashevski O., Freeman R., Willner I. (2009). Enzyme Cascades
Activated on Topologically
Programmed DNA Scaffolds. Nat. Nanotechnol..

[138] Tao K., Xue B., Han S., Aizen R., Shimon L. J. W., Xu Z., Cao Y., Mei D., Wang W., Gazit E. (2020). Bioinspired Suprahelical
Frameworks as Scaffolds for Artificial Photosynthesis. ACS Appl. Mater. Interfaces.

[139] Xue B., Li Y., Yang F., Zhang C., Qin M., Cao Y., Wang W. (2014). An Integrated
Artificial Photosynthesis System Based
on Peptide Nanotubes. Nanoscale.

[140] Sun Z., Diebolder C. A., Renault L., de Groot H. (2019). A Semisynthetic Peptide-Metalloporphyrin
Responsive Matrix for Artificial Photosynthesis. ChemPhotoChem..

[141] Wang C., O’Hagan M. P., Willner B., Willner I. (2022). Bioinspired
Artificial Photosynthetic Systems. Chem. Eur.
J..

[142] Luo G.-F., Biniuri Y., Chen W.-H., Wang J., Neumann E., Marjault H.-B., Nechushtai R., Winkler M., Happe T., Willner I. (2020). Modelling Photosynthesis
with ZnII-Protoporphyrin All-DNA G-Quadruplex/Aptamer Scaffolds. Angew. Chem., Int. Ed..

[143] Hemmig E. A., Creatore C., Wünsch B., Hecker L., Mair P., Parker M. A., Emmott S., Tinnefeld P., Keyser U. F., Chin A. W. (2016). Programming Light-Harvesting
Efficiency Using DNA Origami. Nano Lett..

[144] Hart S. M., Gorman J., Bathe M., Schlau-Cohen G. S. (2023). Engineering
Exciton Dynamics with Synthetic DNA Scaffolds. Acc. Chem. Res..

[145] Bui H., Díaz S. A., Fontana J., Chiriboga M., Veneziano R., Medintz I. L. (2019). Utilizing the Organizational Power
of DNA Scaffolds for New Nanophotonic Applications. Advanced Optical Materials.

[146] Aldaye F. A., Palmer A. L., Sleiman H. F. (2008). Assembling
Materials
with DNA as the Guide. Science.

[147] Kim J. H., Lee M., Lee J. S., Park C. B. (2012). Self-Assembled
Light-Harvesting Peptide Nanotubes for Mimicking Natural Photosynthesis. Angew. Chem., Int. Ed..

[148] Ma K., Yehezkeli O., Domaille D. W., Funke H. H., Cha J. N. (2015). Enhanced
Hydrogen Production from DNA-Assembled Z-Scheme TiO2-CdS Photocatalyst
Systems. Angew. Chem., Int. Ed..

[149] Gust D., Moore T. A., Moore A. L. (2012). Realizing Artificial
Photosynthesis. Faraday Discuss..

[150] Keijer T., Bouwens T., Hessels J., Reek J. N. H. (2021). Supramolecular
Strategies in Artificial Photosynthesis. Chem.
Sci..

[151] Li H., Li F., Zhang B., Zhou X., Yu F., Sun L. (2015). Visible Light-Driven
Water Oxidation Promoted by Host-Guest Interaction
between Photosensitizer and Catalyst with A High Quantum Efficiency. J. Am. Chem. Soc..

[152] Zhang H., Weiss I., Rudra I., Jo W. J., Kellner S., Katsoukis G., Galoppini E., Frei H. (2021). Controlling and Optimizing Photoinduced
Charge Transfer across Ultrathin
Silica Separation Membrane with Embedded Molecular Wires for Artificial
Photosynthesis. ACS Appl. Mater. Interfaces.

[153] Katsoukis G., Frei H. (2018). Heterobinuclear Light Absorber Coupled
to Molecular Wire for Charge Transport across Ultrathin Silica Membrane
for Artificial Photosynthesis. ACS Appl. Mater.
Interfaces.

[154] Cornejo J. A., Sheng H., Edri E., Ajo-Franklin C. M., Frei H. (2018). Nanoscale Membranes That Chemically Isolate and Electronically Wire
up the Abiotic/Biotic Interface. Nat. Commun..

[155] Zhang L., Liu J., Lan Y.-Q. (2024). Hetero-Motif
Molecular
Junction Photocatalysts: A New Frontier in Artificial Photosynthesis. Acc. Chem. Res..

[156] Dong L.-Z., Zhang L., Liu J., Huang Q., Lu M., Ji W.-X., Lan Y.-Q. (2020). Stable
Heterometallic Cluster-Based
Organic Framework Catalysts for Artificial Photosynthesis. Angew. Chem., Int. Ed..

[157] Lu M., Liu J., Li Q., Zhang M., Liu M., Wang J.-L., Yuan D.-Q., Lan Y.-Q. (2019). Rational Design
of Crystalline Covalent Organic Frameworks for Efficient CO2 Photoreduction
with H2O. Angew. Chem., Int. Ed..

[158] Hong Y. H., Nilajakar M., Lee Y.-M., Nam W., Fukuzumi S. (2024). Artificial Photosynthesis
for Regioselective Reduction
of NAD­(P)+ to NAD­(P)H Using Water as an Electron and Proton Source. J. Am. Chem. Soc..

[159] Hong Y. H., Lee Y.-M., Nam W., Fukuzumi S. (2022). Molecular
Photocatalytic Water Splitting by Mimicking Photosystems I and II. J. Am. Chem. Soc..

[160] Andrei V., Chiang Y.-H., Rahaman M., Anaya M., Kang T., Ruggeri E., Stranks S. D., Reisner E. (2025). Modular Perovskite-BiVO4
Artificial Leaves towards Syngas Synthesis on a M2 Scale. Energy Environ. Sci..

[161] O’Regan B., Grätzel M. (1991). A Low-Cost,
High-Efficiency Solar
Cell Based on Dye-Sensitized Colloidal TiO2 Films. Nature.

[162] Yella A., Lee H.-W., Tsao H. N., Yi C., Chandiran A. K., Nazeeruddin Md. K., Diau E. W.-G., Yeh C.-Y., Zakeeruddin S. M., Grätzel M. (2011). Porphyrin-Sensitized Solar Cells
with Cobalt (II/III)-Based Redox Electrolyte Exceed 12% Efficiency. Science.

[163] Luo J., Im J.-H., Mayer M. T., Schreier M., Nazeeruddin M. K., Park N.-G., Tilley S. D., Fan H. J., Grätzel M. (2014). Water Photolysis
at 12.3% Efficiency via Perovskite Photovoltaics and Earth-Abundant
Catalysts. Science.

[164] Andrei V., Roh I., Lin J.-A., Lee J., Shan Y., Lin C.-K., Shelton S., Reisner E., Yang P. (2025). Perovskite-Driven Solar C2 Hydrocarbon Synthesis from CO2. Nat. Catal.

[165] Young J. L., Steiner M. A., Döscher H., France R. M., Turner J. A., Deutsch T. G. (2017). Direct Solar-to-Hydrogen
Conversion via Inverted Metamorphic Multi-Junction Semiconductor Architectures. Nat. Energy.

[166] Gu J., Yan Y., Young J. L., Steirer K. X., Neale N. R., Turner J. A. (2016). Water Reduction by a P-GaInP2 Photoelectrode Stabilized
by an Amorphous TiO2 Coating and a Molecular Cobalt Catalyst. Nat. Mater..

[167] Khaselev O., Turner J. A. (1998). A Monolithic Photovoltaic-Photoelectrochemical
Device for Hydrogen Production via Water Splitting. Science.

[168] Yeung, C. W. S. ; Andrei, V. ; Lee, T. H. ; Durrant, J. R. ; Reisner, E. Organic Semiconductor-BiVO4 Tandem Devices for Solar-Driven H2O and CO2 Splitting. Adv. Mater. 2024, 10.1002/adma.202404110. 38943473

[169] Brown K. A., King P. W. (2020). Coupling Biology
to Synthetic Nanomaterials
for Semi-Artificial Photosynthesis. Photosynth
Res..

[170] Cestellos-Blanco S., Zhang H., Kim J. M., Shen Y., Yang P. (2020). Photosynthetic Semiconductor Biohybrids
for Solar-Driven Biocatalysis. Nat. Catal.

[171] Bachar O., Cohen R., Meirovich M. M., Cohen Y., Yehezkeli O. (2023). Biotic-Abiotic
Hybrids for Bioanalytics
and Biocatalysis. Curr. Opin. Biotechnol..

[172] Gamache M. T., Kurth L., Filmon D. T., Plumeré N., Berggren G. (2023). *E. Coli* -Based Semi-Artificial
Photosynthesis:
Biocompatibility of Redox Mediators and Electron Donors in [FeFe]
Hydrogenase Driven Hydrogen Evolution. Energy
Adv..

[173] Chen S., Takata T., Domen K. (2017). Particulate Photocatalysts
for Overall Water Splitting. Nat. Rev. Mater..

[174] Adam D., Bosche L., Castaneda-Losada L., Winkler M., Apfel U.-P., Happe T. (2017). Sunlight-Dependent
Hydrogen Production by Photosensitizer/Hydrogenase Systems. ChemSusChem.

[175] Alkotaini B., Abdellaoui S., Hasan K., Grattieri M., Quah T., Cai R., Yuan M., Minteer S. D. (2018). Sustainable
Bioelectrosynthesis of the Bioplastic Polyhydroxybutyrate: Overcoming
Substrate Requirement for NADH Regeneration. ACS Sustainable Chem. Eng..

[176] Kim J. Y. H., Jo B. H., Cha H. J. (2011). Production
of Biohydrogen
by Heterologous Expression of Oxygen-Tolerant Hydrogenovibrio Marinus
[NiFe]-Hydrogenase in Escherichia Coli. J. Biotechnol..

[177] Reisner E., Powell D. J., Cavazza C., Fontecilla-Camps J. C., Armstrong F. A. (2009). Visible Light-Driven H _2_ Production by Hydrogenases
Attached to Dye-Sensitized TiO _2_ Nanoparticles. J. Am. Chem. Soc..

[178] Lorenzi M., Gamache M. T., Redman H. J., Land H., Senger M., Berggren G. (2022). Light-Driven [FeFe]
Hydrogenase Based
H _2_ Production in *E. Coli*: A Model Reaction
for Exploring *E. Coli* Based Semiartificial Photosynthetic
Systems. ACS Sustainable Chem. Eng..

[179] Achilleos D. S., Kasap H., Reisner E. (2020). Photocatalytic Hydrogen
Generation Coupled to Pollutant Utilisation Using Carbon Dots Produced
from Biomass. Green Chem..

[180] Jia J., Seitz L. C., Benck J. D., Huo Y., Chen Y., Ng J. W. D., Bilir T., Harris J. S., Jaramillo T. F. (2016). Solar Water
Splitting by Photovoltaic-Electrolysis with a Solar-to-Hydrogen Efficiency
over 30%. Nat. Commun..

[181] Choudhury S., Baeg J.-O., Park N.-J., Yadav R. K. (2014). A Solar
Light-Driven, Eco-Friendly Protocol for Highly Enantioselective Synthesis
of Chiral Alcohols via Photocatalytic/Biocatalytic Cascades. Green Chem..

[182] Kim G.-M., Choi Y., Choi K. R., Lee I., Kim J., Lee B., Lee S. Y., Lee D. C. (2025). *In Vivo* Synthesis
of Semiconductor Nanoparticles in *Azotobacter
Vinelandii* for Light-Driven Ammonia Production. Nanoscale.

[183] Inoue T., Fujishima A., Konishi S., Honda K. (1979). Photoelectrocatalytic
Reduction of Carbon Dioxide in Aqueous Suspensions of Semiconductor
Powders. Nature.

[184] Hutton G. A. M., Reuillard B., Martindale B. C. M., Caputo C. A., Lockwood C. W. J., Butt J. N., Reisner E. (2016). Carbon Dots
as Versatile Photosensitizers for Solar-Driven Catalysis with Redox
Enzymes. J. Am. Chem. Soc..

[185] Holá K., Pavliuk M. V., Németh B., Huang P., Zdražil L., Land H., Berggren G., Tian H. (2020). Carbon Dots and [FeFe] Hydrogenase Biohybrid Assemblies for Efficient
Light-Driven Hydrogen Evolution. ACS Catal..

[186] Utschig L. M., Brahmachari U., Mulfort K. L., Niklas J., Poluektov O. G. (2022). Biohybrid
Photosynthetic Charge Accumulation Detected
by Flavin Semiquinone Formation in Ferredoxin-NADP^+^ Reductase. Chem. Sci..

[187] Utschig L. M., Silver S. C., Mulfort K. L., Tiede D. M. (2011). Nature-Driven
Photochemistry for Catalytic Solar Hydrogen Production: A Photosystem
I-Transition Metal Catalyst Hybrid. J. Am. Chem.
Soc..

[188] Miller M., Robinson W. E., Oliveira A. R., Heidary N., Kornienko N., Warnan J., Pereira I. A. C., Reisner E. (2019). Interfacing
Formate Dehydrogenase with Metal Oxides for the Reversible Electrocatalysis
and Solar-Driven Reduction of Carbon Dioxide. Angew. Chem., Int. Ed..

[189] Liu Y., Rodríguez-Jiménez S., Song H., Pannwitz A., Kim D., Coito A. M., Manuel R. R., Webb S., Su L., Bonke S. A., Milton R. D., Pereira I. A. C., Bonnet S., Hammarström L., Reisner E. (2025). Bio-Inspired Self-Assembly of Enzyme-Micelle
Systems for Semi-Artificial Photosynthesis. Angew. Chem. Int. Ed.

[190] Zhang H., Jaenecke J., Bishara-Robertson I. L., Casadevall C., Redman H. J., Winkler M., Berggren G., Plumeré N., Butt J. N., Reisner E., Jeuken L. J. C. (2024). Semiartificial
Photosynthetic Nanoreactors for H2 Generation. J. Am. Chem. Soc..

[191] Brown K. A., Harris D. F., Wilker M. B., Rasmussen A., Khadka N., Hamby H., Keable S., Dukovic G., Peters J. W., Seefeldt L. C., King P. W. (2016). Light-Driven
Dinitrogen
Reduction Catalyzed by a CdS:Nitrogenase MoFe Protein Biohybrid. Science.

[192] Meirovich M. M., Bachar O., Shemesh M., Cohen Y., Popik A., Yehezkeli O. (2024). Light-Driven,
Bias-Free Nitrogenase-Based
Bioelectrochemical Cell for Ammonia Generation. Biosens. Bioelectron..

[193] Ding Y., Lee C. C., Hu Y., Ribbe M. M., Nagpal P., Chatterjee A. (2023). Light-Driven Transformation of Carbon
Monoxide into Hydrocarbons Using CdS@ZnS:VFe Protein Biohybrids. ChemSusChem.

[194] Meirovich M. M., Bachar O., Yehezkeli O. (2020). Artificial,
Photoinduced Activation of Nitrogenase Using Directed and Mediated
Electron Transfer Processes. Catalysts.

[195] Meirovich M. M., Bachar O., Nandi R., Amdursky N., Yehezkeli O. (2021). Tailoring Quantum Dot Sizes for Optimal
Photoinduced
Catalytic Activation of Nitrogenase. ChemSusChem.

[196] Liu Y., Bin Mohamad Annuar A., Rodríguez-Jiménez S., Yeung C. W. S., Wang Q., Coito A. M., Manuel R. R., Pereira I. A. C., Reisner E. (2024). Solar Fuel
Synthesis Using a Semiartificial
Colloidal Z-Scheme. J. Am. Chem. Soc..

[197] Sasaki Y., Kato H., Kudo A. (2013). [Co­(Bpy)­3]­3+/2+ and
[Co­(Phen)­3]­3+/2+ Electron Mediators for Overall Water Splitting under
Sunlight Irradiation Using Z-Scheme Photocatalyst System. J. Am. Chem. Soc..

[198] Li L., Xu Z., Huang X. (2021). Whole-Cell-Based
Photosynthetic Biohybrid
Systems for Energy and Environmental Applications. ChemPlusChem..

[199] Sakimoto K. K., Kornienko N., Yang P. (2017). Cyborgian Material
Design for Solar Fuel Production: The Emerging Photosynthetic Biohybrid
Systems. Acc. Chem. Res..

[200] Sétif P. (2015). Electron-Transfer Kinetics in Cyanobacterial
Cells:
Methyl Viologen Is a Poor Inhibitor of Linear Electron Flow. Biochimica et Biophysica Acta (BBA) - Bioenergetics.

[201] Liang J., Chen Z., Yin P., Hu H., Cheng W., Shang J., Yang Y., Yuan Z., Pan J., Yin Y., Li W., Chen X., Gao X., Qiu B., Wang B. (2023). Efficient Semi-Artificial Photosynthesis of Ethylene
by a Self-Assembled InP-Cyanobacterial Biohybrid System. ChemSusChem.

[202] Ye J., Gu W., Hu J., Chen L., Yang C., Gao J., Zhou S. (2025). Toward Next-Generation
Semiartificial Photosynthesis:
Multidisciplinary Engineering of Biohybrid Systems. Chem. Rev..

[203] Jiang X., van Wonderen J. H., Butt J. N., Edwards M. J., Clarke T. A., Blumberger J. (2020). Which Multi-Heme
Protein Complex
Transfers Electrons More Efficiently? Comparing MtrCAB from Shewanella
with OmcS from Geobacter. J. Phys. Chem. Lett..

[204] Firer-Sherwood M., Pulcu G. S., Elliott S. J. (2008). Electrochemical
Interrogations of the Mtr Cytochromes from Shewanella: Opening a Potential
Window. J. Biol. Inorg. Chem..

[205] Han H.-X., Tian L.-J., Liu D.-F., Yu H.-Q., Sheng G.-P., Xiong Y. (2022). Reversing Electron
Transfer Chain
for Light-Driven Hydrogen Production in Biotic-Abiotic Hybrid Systems. J. Am. Chem. Soc..

[206] Sabella S., Carney R. P., Brunetti V., Malvindi M. A., Al-Juffali N., Vecchio G., Janes S. M., Bakr O. M., Cingolani R., Stellacci F., Pompa P. P. (2014). A General Mechanism
for Intracellular Toxicity of Metal-Containing Nanoparticles. Nanoscale.

[207] Mahana A., Guliy O. I., Mehta S. K. (2021). Accumulation
and
Cellular Toxicity of Engineered Metallic Nanoparticle in Freshwater
Microalgae: Current Status and Future Challenges. Ecotoxicology and Environmental Safety.

[208] Wang Y., Liu Y., Bai L., Wang J., Zhao N., Cui D., Zhao M. (2024). Low-Toxicity Self-Photosensitized
Biohybrid Systems for Enhanced Light-Driven H2 Production. International Journal of Molecular Sciences.

[209] Bachar O., Meirovich M. M., Yehezkeli O. (2024). Integrated
Biotic-Abiotic Solar Driven NADPH Regeneration Platform in *Escherichia Coli* for Chemical Biomanufacturing Applications. Adv. Funct Materials.

[210] DeJong E. S., Chang C., Gilson M. K., Marino J. P. (2003). Proflavine
Acts as a Rev Inhibitor by Targeting the High-Affinity Rev Binding
Site of the Rev Responsive Element of HIV-1. Biochemistry.

[211] Sakimoto K. K., Wong A. B., Yang P. (2016). Self-Photosensitization
of Nonphotosynthetic Bacteria for Solar-to-Chemical Production. Science.

[212] Peidong Yang H. Z. (2018). Bacteria Photosensitized by Intracellular
Gold Nanoclusters
for Solar Fuel Production. Nat. Nanotechnol..

[213] Honda Y., Hagiwara H., Ida S., Ishihara T. (2016). Application
to Photocatalytic H _2_ Production of a Whole-Cell Reaction
by Recombinant *Escherichia Coli* Cells Expressing
[FeFe]-Hydrogenase and Maturases Genes. Angew.
Chem..

[214] Wang B., Zeng C., Chu K. H., Wu D., Yip H. Y., Ye L., Wong P. K. (2017). Enhanced Biological
Hydrogen Production from *Escherichia Coli* with Surface
Precipitated Cadmium Sulfide Nanoparticles. Adv. Energy Mater..

[215] Zhang H., Casadevall C., Van Wonderen J. H., Su L., Butt J. N., Reisner E., Jeuken L. J. C. (2023). Rational Design
of Covalent Multiheme Cytochrome-Carbon Dot Biohybrids for Photoinduced
Electron Transfer. Adv. Funct Materials.

[216] Song S., Ivanov T., Doan-Nguyen T. P., da Silva L. C., Xie J., Landfester K., Cao S. (2025). Synthetic Biomolecular Condensates: Phase-Separation Control, Cytomimetic
Modelling and Emerging Biomedical Potential. Angew. Chem., Int. Ed..

[217] Mouhib M., Reggente M., Li L., Schuergers N., Boghossian A. A. (2023). Extracellular Electron Transfer Pathways to Enhance
the Electroactivity of Modified Escherichia Coli. Joule.

[218] Rowe S. F., Le Gall G., Ainsworth E. V., Davies J. A., Lockwood C. W. J., Shi L., Elliston A., Roberts I. N., Waldron K. W., Richardson D. J., Clarke T. A., Jeuken L. J. C., Reisner E., Butt J. N. (2017). Light-Driven
H _2_ Evolution and C=C or C=O Bond Hydrogenation by *Shewanella Oneidensis*: A Versatile Strategy for Photocatalysis
by Nonphotosynthetic Microorganisms. ACS Catal..

[219] Li H., Yu X., Qin Y., Jiang T., Wang J., Cai Z., Xu J., Ge Y., Sun H., Qi Z., Liu J. (2024). Synergistic Approaches
for Enhanced Light-Driven Hydrogen Production:
A Membrane-Anchoring Protein-Engineered Biohybrid System with Dual
Photosensitizers Strategy. ACS Materials Lett..

[220] Kalathil S., Rahaman M., Lam E., Augustin T. L., Greer H. F., Reisner E. (2024). Solar-Driven Methanogenesis through
Microbial Ecosystem Engineering on Carbon Nitride. Angew. Chem. Int. Ed.

[221] Dinis-Oliveira R. J., Duarte J. A., Sánchez-Navarro A., Remião F., Bastos M. L., Carvalho F. (2008). Paraquat Poisonings:
Mechanisms of Lung Toxicity, Clinical Features, and Treatment. Critical Reviews in Toxicology.

[222] Michaelis L., Hill E. S. (1933). The Viologen Indicators. J. Gen. Physiol..

[223] Clifford E. R., Bradley R. W., Wey L. T., Lawrence J. M., Chen X., Howe C. J., Zhang J. Z. (2021). Phenazines
as Model
Low-Midpoint Potential Electron Shuttles for Photosynthetic Bioelectrochemical
Systems. Chem. Sci..

[224] Li H., Yu X., Wu Y., Li C., Xu Z., Liu W., Chen S., Sun H., Ge Y., Qi Z., Liu J. (2024). Membraneless Organelles Assembled
by AuNPs-Enzyme Integration in
Non-Photosynthetic Bacteria: Achieving High Specificity and Selectivity
for Solar Hydrogen Production. Chemical Engineering
Journal.

[225] Yu X., Li H., Xu C., Xu Z., Chen S., Liu W., Zhang T., Sun H., Ge Y., Qi Z., Liu J. (2024). Liquid-Liquid Phase Separation-Mediated
Photocatalytic Subcellular
Hybrid System for Highly Efficient Hydrogen Production. Advanced Science.

[226] Luo B., Wang Y., Li D., Shen H., Xu L., Fang Z., Xia Z., Ren J., Shi W., Yong Y. (2021). A Periplasmic Photosensitized Biohybrid System for Solar Hydrogen
Production. Adv. Energy Mater..

[227] Heyman A., Levy I., Altman A., Shoseyov O. (2007). SP1 as a Novel
Scaffold Building Block for Self-Assembly Nanofabrication of Submicron
Enzymatic Structures. Nano Lett..

[228] Wei W., Sun P., Li Z., Song K., Su W., Wang B., Liu Y., Zhao J. (2018). A Surface-Display Biohybrid
Approach to Light-Driven Hydrogen Production in Air. Sci. Adv..

[229] Wang Q., Kalathil S., Pornrungroj C., Sahm C. D., Reisner E. (2022). Bacteria-Photocatalyst Sheet for
Sustainable Carbon Dioxide Utilization. Nature
Catalysis.

[230] Kim T. W., Choi K.-S. (2014). Nanoporous BiVO4 Photoanodes with
Dual-Layer Oxygen Evolution Catalysts for Solar Water Splitting. Science.

[231] Lee D. K., Choi K.-S. (2018). Enhancing Long-Term
Photostability
of BiVO 4 Photoanodes for Solar Water Splitting by Tuning Electrolyte
Composition. Nature Energy.

[232] Li B., Qin Q., Jian C., Cai Q., Liu W. (2021). Boosting the
Quantum Efficiency of the BiVO4 Photoanode by Increasing the Oxygen
Vacancies for Highly-Efficient Solar Water Oxidation. Dalton Trans..

[233] Qayum A., Guo M., Wei J., Dong S., Jiao X., Chen D., Wang T. (2020). An in Situ
Combustion
Method for Scale-up Fabrication of BiVO4 Photoanodes with Enhanced
Long-Term Photostability for Unassisted Solar Water Splitting. J. Mater. Chem. A.

[234] Patil Kunturu P., Lavorenti M., Bera S., Johnson H., Kinge S., van de Sanden M. C. M., Tsampas M. N. (2024). Scaling up BiVO4
Photoanodes on Porous Ti Transport Layers for Solar Hydrogen Production. ChemSusChem.

[235] Mukha D., Cohen Y., Yehezkeli O. (2020). BiVO4/Bilirubin
Oxidase Photo­(Bio)­Electrochemical Cells for Unbiased Light-Triggered
Electrical Power Generation. ChemSusChem.

[236] Cohen R., Cohen Y., Mukha D., Yehezkeli O. (2021). Oxygen Insensitive
Amperometric Glucose Biosensor Based on FAD Dependent Glucose Dehydrogenase
Co-Entrapped with DCPIP or DCNQ in a Polydopamine Layer. Electrochim. Acta.

[237] Herzallh N. S., Cohen Y., Cohen R., Chmelnik O., Shoham Y., Yehezkeli O. (2021). Cellulose to Electricity Conversion
by an Enzymatic Biofuel Cell. Sustainable Energy
Fuels.

[238] Hardt S., Stapf S., Filmon D. T., Birrell J. A., Rüdiger O., Fourmond V., Léger C., Plumeré N. (2021). Reversible
H2 Oxidation and Evolution by Hydrogenase
Embedded in a Redox Polymer Film. Nat. Catal.

[239] Cheng F., Pavliuk O., Hardt S., Hunt L. A., Cai B., Kubart T., Hammarström L., Plumeré N., Berggren G., Tian H. (2024). Embedding Biocatalysts in a Redox
Polymer Enhances the Performance of Dye-Sensitized Photocathodes in
Bias-Free Photoelectrochemical Water Splitting. Nat. Commun..

[240] Plumeré N., Rüdiger O., Oughli A. A., Williams R., Vivekananthan J., Pöller S., Schuhmann W., Lubitz W. (2014). A Redox Hydrogel Protects
Hydrogenase from High-Potential
Deactivation and Oxygen Damage. Nature Chem..

[241] Edwardes Moore E., Andrei V., Zacarias S., Pereira I. A. C., Reisner E. (2020). Integration
of a Hydrogenase in a Lead Halide Perovskite
Photoelectrode for Tandem Solar Water Splitting. ACS Energy Lett..

[242] Zheng T., Zhang M., Wu L., Guo S., Liu X., Zhao J., Xue W., Li J., Liu C., Li X., Jiang Q., Bao J., Zeng J., Yu T., Xia C. (2022). Upcycling CO2 into Energy-Rich Long-Chain Compounds via Electrochemical
and Metabolic Engineering. Nat. Catal.

[243] Kuk S. K., Singh R. K., Nam D. H., Singh R., Lee J.-K., Park C. B. (2017). Photoelectrochemical Reduction of
Carbon Dioxide to Methanol through a Highly Efficient Enzyme Cascade. Angew. Chem., Int. Ed..

[244] Lee Y. W., Boonmongkolras P., Son E. J., Kim J., Lee S. H., Kuk S. K., Ko J. W., Shin B., Park C. B. (2018). Unbiased
Biocatalytic Solar-to-Chemical Conversion
by FeOOH/BiVO4/Perovskite Tandem Structure. Nat. Commun..

[245] Yeung C. W. S., Liu Y., Vahey D. M., Cobb S. J., Andrei V., Coito A. M., Manuel R. R., Pereira I. A. C., Reisner E. (2025). Semi-Artificial
Leaf Interfacing Organic Semiconductors
and Enzymes for Solar Chemical Synthesis. Joule.

[246] Choi D. S., Lee H., Tieves F., Lee Y. W., Son E. J., Zhang W., Shin B., Hollmann F., Park C. B. (2019). Bias-Free In Situ H2O2 Generation
in a Photovoltaic-Photoelectrochemical
Tandem Cell for Biocatalytic Oxyfunctionalization. ACS Catal..

[247] Zhou L., Tan Y., Wang J., Xu W., Yuan Y., Cai W., Zhu S., Zhu J. (2016). 3D Self-Assembly
of Aluminium Nanoparticles for Plasmon-Enhanced Solar Desalination. Nature Photon.

[248] Fan P., Wu H., Zhong M., Zhang H., Bai B., Jin G. (2016). Large-Scale Cauliflower-Shaped
Hierarchical Copper Nanostructures
for Efficient Photothermal Conversion. Nanoscale.

[249] Burek B. O., de Boer S. R., Tieves F., Zhang W., van Schie M., Bormann S., Alcalde M., Holtmann D., Hollmann F., Bahnemann D. W., Bloh J. Z. (2019). Photoenzymatic Hydroxylation
of Ethylbenzene Catalyzed by Unspecific Peroxygenase: Origin of Enzyme
Inactivation and the Impact of Light Intensity and Temperature. ChemCatChem..

[250] Rapson T. D., Gregg C. M., Allen R. S., Ju H., Doherty C. M., Mulet X., Giddey S., Wood C. C. (2020). Insights
into Nitrogenase Bioelectrocatalysis for Green Ammonia Production. ChemSusChem.

[251] Liu C., Sakimoto K. K., Colón B. C., Silver P. A., Nocera D. G. (2017). Ambient
Nitrogen Reduction Cycle Using a Hybrid Inorganic-Biological System. Proc. Natl. Acad. Sci. U. S. A..

[252] Badalyan A., Yang Z.-Y., Seefeldt L. C. (2024). A Voltammetric
Study
of Nitrogenase MoFe-Protein Using Low-Potential Electron Transfer
Mediators. Bioelectrochemistry.

[253] Seefeldt L. C., Peters J. W., Beratan D. N., Bothner B., Minteer S. D., Raugei S., Hoffman B. M. (2018). Control
of Electron
Transfer in Nitrogenase. Curr. Opin Chem. Biol..

[254] Clinger A., Yang Z.-Y., Pellows L. M., King P., Mus F., Peters J. W., Dukovic G., Seefeldt L. C. (2024). Hole-Scavenging
in Photo-Driven N2 Reduction Catalyzed by a CdS-Nitrogenase MoFe Protein
Biohybrid System. Journal of Inorganic Biochemistry.

[255] Vansuch G. E., Mulder D. W., Chica B., Ruzicka J. L., Yang Z.-Y., Pellows L. M., Willis M. A., Brown K. A., Seefeldt L. C., Peters J. W., Dukovic G., King P. W. (2023). Cryo-Annealing
of Photoreduced CdS Quantum Dot-Nitrogenase MoFe Protein Complexes
Reveals the Kinetic Stability of the E4­(2N2H) Intermediate. J. Am. Chem. Soc..

[256] Lee Y. S., Yuan M., Cai R., Lim K., Minteer S. D. (2020). Nitrogenase Bioelectrocatalysis: ATP-Independent Ammonia
Production Using a Redox Polymer/MoFe Protein System. ACS Catal..

[257] Milton R. D., Cai R., Abdellaoui S., Leech D., De Lacey A. L., Pita M., Minteer S. D. (2017). Bioelectrochemical
Haber-Bosch Process: An Ammonia-Producing H2/N2 Fuel Cell. Angew. Chem., Int. Ed..

[258] Ding J., Zhao J., Zhang H., Dong S. (2024). Bias-Free
Glucose/O2 Bio-Photoelectrochemical System for Multi-Energy Conversion
and Phenolic Pollutant Degradation. Biosens.
Bioelectron..

[259] Liu C.-G., Xiao Y., Xia X.-X., Zhao X.-Q., Peng L., Srinophakun P., Bai F.-W. (2019). Cellulosic Ethanol
Production: Progress, Challenges and Strategies for Solutions. Biotechnology Advances.

[260] Harris A. W., Yehezkeli O., Hafenstine G. R., Goodwin A. P., Cha J. N. (2017). Light-Driven Catalytic
Upgrading
of Butanol in a Biohybrid Photoelectrochemical System. ACS Sustainable Chem. Eng..

[261] Hang T., Wu L., Liu W., Yang L., Zhang T. (2024). Research Progress of Bifunctional
Photocatalysts for Biomass Conversion
and Fuel Production. Advanced Energy and Sustainability
Research.

[262] Granone L. I., Sieland F., Zheng N., Dillert R., Bahnemann D. W. (2018). Photocatalytic
Conversion of Biomass into Valuable
Products: A Meaningful Approach?. Green Chem..

[263] Ibrahim N., Kamarudin S. K., Minggu L. J. (2014). Biofuel from Biomass
via Photo-Electrochemical Reactions: An Overview. J. Power Sources.

[264] Sun Y., Miao J., Fan X., Zhang K., Zhang T. (2024). Recent Progress
in Electrochemical Conversion from Biomass Derivatives into High-Value-Added
Chemicals. Small Structures.

[265] Troiano D. T., Studer M. H.-P. (2025). Microbial Consortia
for the Conversion
of Biomass into Fuels and Chemicals. Nat. Commun..

[266] Osman A. I., Mehta N., Elgarahy A. M., Al-Hinai A., Al-Muhtaseb A. H., Rooney D. W. (2021). Conversion of Biomass
to Biofuels
and Life Cycle Assessment: A Review. Environ.
Chem. Lett..

[267] Begum Y. A., Kumari S., Jain S. K., Garg M. C. (2024). A Review
on Waste Biomass-to-Energy: Integrated Thermochemical and Biochemical
Conversion for Resource Recovery. Environ. Sci.:
Adv..

[268] Yehezkeli O., Tel-Vered R., Michaeli D., Willner I., Nechushtai R. (2014). Photosynthetic
Reaction Center-Functionalized Electrodes
for Photo-Bioelectrochemical Cells. Photosynth
Res..

[269] Tel-Vered R., Willner I. (2014). Photo-Bioelectrochemical Cells for
Energy Conversion, Sensing, and Optoelectronic Applications. ChemElectroChem..

[270] Wang F., Liu X., Willner I. (2013). Integration
of Photoswitchable
Proteins, Photosynthetic Reaction Centers and Semiconductor/Biomolecule
Hybrids with Electrode Supports for Optobioelectronic Applications. Adv. Mater..

[271] Herrero-Medina Z., Wang P., Lielpetere A., Bashammakh A. S., Alyoubi A. O., Katakis I., Conzuelo F., Schuhmann W. (2022). A Biophotoelectrode Based on Boronic Acid-Modified *Chlorella Vulgaris* Cells Integrated within a Redox Polymer. Bioelectrochemistry.

[272] Xing T., Lv Y., Wu G., Zhang Z., Zhang W., Wang X., Chen Z., Zhao W., Conzuelo F., Zhao F. (2025). A Novel Biofuel
Cell Based on Galactose
Oxidase and Bilirubin Oxidase for Efficient Glycerol Conversion and
Electricity Generation. Chemical Engineering
Journal.

[273] Silva C. C. G., Martins G., Luís A., Rojas-Mantilla H. D., Rovisco A., Martins R., Fortunato E., Pereira I. A. C., Zanoni M. V. B., Garrido S. S., Conzuelo F. (2025). Microalgae-Based
Hybrid Biophotoelectrode for Efficient Light Energy Conversion. ACS Electrochem..

[274] Franco J. H., Bonaldo J. V., Minteer S. D., De Andrade A. R. (2025). Enhanced
Biofuel Cells Based on a Hybrid Enzymatic/Bimetallic Composite for
Complete Lactate Catalytic Electrooxidation. ACS Mater. Au.

[275] Simoska O., Cummings D. A., Gaffney E. M., Langue C., Primo T. G., Weber C. J., Witt C. E., Minteer S. D. (2023). Enhancing the Performance of Microbial
Fuel Cells via
Metabolic Engineering of Escherichia Coli for Phenazine Production. ACS Sustainable Chem. Eng..

[276] Weliwatte N. S., Grattieri M., Minteer S. D. (2021). Rational Design
of Artificial Redox-Mediating Systems toward Upgrading Photobioelectrocatalysis. Photochem. Photobiol. Sci..

[277] Sokol K. P., Robinson W. E., Warnan J., Kornienko N., Nowaczyk M. M., Ruff A., Zhang J. Z., Reisner E. (2018). Bias-Free
Photoelectrochemical Water Splitting with Photosystem II on a Dye-Sensitized
Photoanode Wired to Hydrogenase. Nat. Energy.

[278] Shi Q., Duan H. (2022). Recent Progress in Photoelectrocatalysis beyond Water
Oxidation. Chem. Catalysis.

[279] Khan H., Bera S., Jung M.-J., Kwon S.-H. (2024). Rational
Design of Photoanodes to Produce Value-Added Chemicals Coupled with
Hydrogen. ChemElectroChem..

[280] Ouyang D., Wang F., Gao D., Han W., Hu X., Qiao D., Zhao X. (2022). Light-Driven Lignocellulosic
Biomass
Conversion for Production of Energy and Chemicals. iScience.

[281] Uekert T., Pichler C. M., Schubert T., Reisner E. (2021). Solar-Driven
Reforming of Solid Waste for a Sustainable Future. Nat. Sustain.

[282] Kim J., Um Y., Han S., Hilberath T., Kim Y. H., Hollmann F., Park C. B. (2022). Unbiased Photoelectrode
Interfaces for Solar Coupling of Lignin Oxidation with Biocatalytic
C=C Bond Hydrogenation. ACS Appl. Mater. Interfaces.

[283] Choi Y., Mehrotra R., Lee S.-H., Nguyen T. V. T., Lee I., Kim J., Yang H.-Y., Oh H., Kim H., Lee J.-W., Kim Y. H., Jang S.-Y., Jang J.-W., Ryu J. (2022). Bias-Free
Solar Hydrogen Production at 19.8 mA Cm-2 Using Perovskite
Photocathode and Lignocellulosic Biomass. Nat.
Commun..

[284] Ko M., Pham L. T. M., Sa Y. J., Woo J., Nguyen T. V. T., Kim J. H., Oh D., Sharma P., Ryu J., Shin T. J., Joo S. H., Kim Y. H., Jang J.-W. (2019). Unassisted
Solar Lignin Valorisation Using a Compartmented Photo-Electro-Biochemical
Cell. Nat. Commun..

[285] Kageshima Y., Yoshimura T., Koh S., Mizuno M., Teshima K., Nishikiori H. (2021). Photoelectrochemical
Complete Decomposition
of Cellulose for Electric Power Generation. ChemCatChem..

[286] Le Duy N., Chuang P.-C., Lin C.-Y., Lai Y.-H. (2025). Photoelectrochemical
Valorization of Cellulose over Bismuth-Based Oxide Modified Titanium
Dioxide Photoanodes. J. Photochem. Photobiol.,
A.

[287] Shemesh M., Cohen Y., Cohen R., Meirovich M. M., Herzallh N. S., Chmelnik O., Shoham Y., Yehezkeli O. (2023). Light-Driven
and Bias-Free Direct Conversion of Cellulose to Electrical Power. Cell Reports Physical Science.

[288] dos Santos L., Climent V., Blanford C. F., Armstrong F. A. (2010). Mechanistic
Studies of the ‘Blue’ Cu Enzyme, Bilirubin Oxidase,
as a Highly Efficient Electrocatalyst for the Oxygen Reduction Reaction. Phys. Chem. Chem. Phys..

[289] Jiang D., Zhang L., Yue Q., Wang T., Huang Q., Du P. (2021). Efficient Suppression
of Surface
Charge Recombination by CoP-Modified Nanoporous BiVO4 for Photoelectrochemical
Water Splitting. Int. J. Hydrogen Energy.

[290] Shen X., Zhao L., Fan W., Ren J., Wang Q., Wang A., Shang D., Zhu W. (2021). Efficient
Photoelectrochemical Water Oxidation of Cobalt Phthalocyanine Decorated
BiVO4 Photoanode by Improving Kinetics. Appl.
Surf. Sci..

[291] Tong H., Jiang Y., Zhang Q., Jiang W., Wang K., Luo X., Lin Z., Xia L. (2019). Boosting Photoelectrochemical
Water Oxidation with Cobalt Phosphide Nanosheets on Porous BiVO4. ACS Sustainable Chem. Eng..

[292] Das P., Tiwari P. (2019). Thermal Degradation
Study of Waste Polyethylene Terephthalate
(PET) under Inert and Oxidative Environments. Thermochim. Acta.

[293] Ugduler S., Van Geem K. M., Denolf R., Roosen M., Mys N., Ragaert K., De Meester S. (2020). Towards Closed-Loop Recycling of
Multilayer and Coloured PET Plastic Waste by Alkaline Hydrolysis. Green Chem..

[294] Zhang Y., Zhao P., Yu Z., Zhao J., Zhai J., Dong S. (2025). NiFe-LDH/BiVO4-BOD
Photocatalytic
Fuel Cell for Bias-Free and Selective 5-Hydroxymethylfurfural Oxidation
to 2,5-Furandicarboxylic Acid. ACS Appl. Mater.
Interfaces.

[295] Gao R.-T., Wang L. (2020). Stable Cocatalyst-Free BiVO4 Photoanodes
with Passivated Surface States for Photocorrosion Inhibition. Angew. Chem., Int. Ed..

[296] Bhattacharjee S., Rahaman M., Andrei V., Miller M., Rodríguez-Jiménez S., Lam E., Pornrungroj C., Reisner E. (2023). Photoelectrochemical CO2-to-Fuel
Conversion with Simultaneous
Plastic Reforming. Nature Synthesis.

[297] Li J., Ma H.-P., Zhao G., Huang G., Sun W., Peng C. (2024). Plastic Waste Conversion
by Leveraging Renewable Photo/Electro-Catalytic
Technologies. ChemSusChem.

[298] Murphy N. P., Dempsey S. H., DesVeaux J. S., Uekert T., Chang A. C., Mailaram S., Alherech M., Alt H. M., Ramirez K. J., Norton-Baker B., Bell E. L., Singer C. A., Pickford A. R., McGeehan J. E., Sobkowicz M. J., Beckham G. T. (2025). Process Innovations to Enable Viable
Enzymatic Poly­(Ethylene
Terephthalate) Recycling. Nat. Chem. Eng..

[299] Kim J., Jang J., Hilberath T., Hollmann F., Park C. B. (2022). Photoelectrocatalytic
Biosynthesis Fuelled by Microplastics. Nat.
Synth.

[300] Hanson A. D., McCarty D. R., Henry C. S., Xian X., Joshi J., Patterson J. A., García-García J. D., Fleischmann S. D., Tivendale N. D., Millar A. H. (2021). The Number of Catalytic
Cycles in an Enzyme’s Lifetime and Why It Matters to Metabolic
Engineering. Proc. Natl. Acad. Sci. U. S. A..

[301] Diekert G. (1990). CO2 Reduction to Acetate in Anaerobic Bacteria. FEMS Microbiology Reviews.

[302] Seefeldt L. C., Yang Z.-Y., Lukoyanov D. A., Harris D. F., Dean D. R., Raugei S., Hoffman B. M. (2020). Reduction
of Substrates by Nitrogenases. Chem. Rev..

[303] Catania C., Karbelkar A. A., Furst A. L. (2021). Engineering the
Interface between Electroactive Bacteria and Electrodes. Joule.

[304] Bird L. J., Kundu B. B., Tschirhart T., Corts A. D., Su L., Gralnick J. A., Ajo-Franklin C. M., Glaven S. M. (2021). Engineering Wired Life: Synthetic Biology for Electroactive
Bacteria. ACS Synth. Biol..

[305] Xiao S., Li Z., Fu Q., Li Y., Li J., Zhang L., Liao Q., Zhu X. (2020). Hybrid Microbial
Photoelectrochemical
System Reduces CO2 to CH4 with 1.28% Solar Energy Conversion Efficiency. Chemical Engineering Journal.

[306] Lu L., Li Z., Chen X., Wang H., Dai S., Pan X., Ren Z. J., Gu J. (2020). Spontaneous Solar Syngas
Production
from CO2 Driven by Energetically Favorable Wastewater Microbial Anodes. Joule.

[307] Matsuo R., Watanabe S., Okabe S. (2025). Microbial Photoelectrochemical
Cell Using Hybrid CuO/ZnO/CuO and Shewanella Oneidensis MR-1 Anode
for Hydrogen Production. Chemical Engineering
Journal.

[308] Shemesh M., Cohen Y., Meirovich M. M., Shulami S., Fishman A., Yehezkeli O. (2025). Microbial-Photoelectrochemical
Cell for the Conversion of Raw Cellulose Materials into Electrical
Power and Chemicals. Biosens. Bioelectron..

[309] Gleizer S., Ben-Nissan R., Bar-On Y. M., Antonovsky N., Noor E., Zohar Y., Jona G., Krieger E., Shamshoum M., Bar-Even A., Milo R. (2019). Conversion of Escherichia
Coli to Generate All Biomass Carbon from CO2. Cell.

[310] Kan S. B. J., Lewis R. D., Chen K., Arnold F. H. (2016). Directed
Evolution of Cytochrome c for Carbon-Silicon Bond Formation: Bringing
Silicon to Life. Science.

[311] Romero P. A., Arnold F. H. (2009). Exploring Protein
Fitness Landscapes
by Directed Evolution. Nat. Rev. Mol. Cell Biol..

[312] Bachar O., Meirovich M. M., Leshinsky N., Yehezkeli O. (2025). Microbial/Enzymatic Light-Induced
NADPH Regeneration
Electrochemical Cells for Continuous Electrosynthesis of Enantioselective
Products. Trends Biotechnol..

[313] Liu G., Jiang Y. (2025). Towards Biocatalytic Conversion Driven
by (Photo)­Electrocatalytic
Cofactor Regeneration. Trends Biotechnol..

[314] Ben-Shahar Y., Scotognella F., Kriegel I., Moretti L., Cerullo G., Rabani E., Banin U. (2016). Optimal Metal Domain
Size for Photocatalysis with Hybrid Semiconductor-Metal Nanorods. Nat. Commun..

[315] Dukovic G., Merkle M. G., Nelson J. H., Hughes S. M., Alivisatos A. P. (2008). Photodeposition of Pt on Colloidal CdS and CdSe/CdS
Semiconductor Nanostructures. Adv. Mater..

[316] Mokari T., Rothenberg E., Popov I., Costi R., Banin U. (2004). Selective
Growth of Metal Tips onto Semiconductor Quantum Rods and
Tetrapods. Science.

[317] Habas S. E., Yang P., Mokari T. (2008). Selective
Growth of
Metal and Binary Metal Tips on CdS Nanorods. J. Am. Chem. Soc..

[318] Amirav L., Alivisatos A. P. (2010). Photocatalytic
Hydrogen Production
with Tunable Nanorod Heterostructures. J. Phys.
Chem. Lett..

[319] Wilker M. B., Shinopoulos K. E., Brown K. A., Mulder D. W., King P. W., Dukovic G. (2014). Electron Transfer Kinetics in CdS
Nanorod-[FeFe]-Hydrogenase Complexes and Implications for Photochemical
H2 Generation. J. Am. Chem. Soc..

[320] Brown K.
A., Wilker M. B., Boehm M., Dukovic G., King P. W. (2012). Characterization
of Photochemical Processes for H2
Production by CdS Nanorod-[FeFe] Hydrogenase Complexes. J. Am. Chem. Soc..

[321] Wakerley D. W., Kuehnel M. F., Orchard K. L., Ly K. H., Rosser T. E., Reisner E. (2017). Solar-Driven Reforming
of Lignocellulose
to H2 with a CdS/CdOx Photocatalyst. Nat. Energy.

[322] Li C., Wang H., Ming J., Liu M., Fang P. (2017). Hydrogen Generation
by Photocatalytic Reforming of Glucose with Heterostructured CdS/MoS2
Composites under Visible Light Irradiation. Int. J. Hydrogen Energy.

[323] Liu Q.-Y., Wang P., Zhang F.-G., Yuan Y.-J. (2022). Visible-Light-Driven
Photocatalytic Cellulose-to-H2 Conversion by MoS2/ZnIn2S4 Photocatalyst
with Cellulase Assistance. ChemPhysChem.

[324] Hang T., Wu L., Liu W., Yang L., Zhang T. (2024). Research Progress of Bifunctional Photocatalysts for Biomass Conversion
and Fuel Production. Advanced Energy and Sustainability
Research.

[325] Ban L., Zhang Y., Sun D., Zhou Y., Li Y., Xu C., Yang S., Zhang H. (2025). Photocatalytic Hydrogen Evolution
Driven by Advanced Metal Sulfides from Sustainable Multilevel Biomass
and Waste Plastics. Adv. Funct. Mater..

[326] Qi M.-Y., Conte M., Anpo M., Tang Z.-R., Xu Y.-J. (2021). Cooperative Coupling of Oxidative
Organic Synthesis and Hydrogen
Production over Semiconductor-Based Photocatalysts. Chem. Rev..

[327] Ashraf M., Ullah N., Khan I., Tremel W., Ahmad S., Tahir M. N. (2023). Photoreforming of
Waste Polymers
for Sustainable Hydrogen Fuel and Chemicals Feedstock: Waste to Energy. Chem. Rev..

[328] Tang J.-P., Chen Y., Wang Z.-Y., Hu Y.-H., Wang J.-H., Bao L., Zhao Z.-Y., Yuan Y.-J. (2025). Sustainable
H2 Production from Lignocellulosic Biomass over MoS2Modified Sulfur
Vacancy Enriched ZnIn2S4 Photocatalyst. ACS
Catal..

[329] Luu X.-C., Phan Thi L.-A., Raizada P., Singh P., Nguyen L. H., Ghotekar S., Nguyen V.-H. (2024). Solar-Driven Reforming
of Lignocellulosic Biomass to Renewable Biohydrogen: A Review. ChemCatChem..

[330] Kasap H., Achilleos D. S., Huang A., Reisner E. (2018). Photoreforming
of Lignocellulose into H2 Using Nanoengineered Carbon Nitride under
Benign Conditions. J. Am. Chem. Soc..

[331] Ismael M., Shang Q., Yue J., Wark M. (2024). Photooxidation
of Biomass for Sustainable Chemicals and Hydrogen Production on Graphitic
Carbon Nitride-Based Materials: A Comprehensive Review. Materials Today Sustainability.

[332] Akhundi A., Badiei A., Ziarani G. M., Habibi-Yangjeh A., Muñoz-Batista M. J., Luque R. (2020). Graphitic Carbon Nitride-Based
Photocatalysts: Toward Efficient Organic Transformation for Value-Added
Chemicals Production. Molecular Catalysis.

[333] Wang J., Kumar P., Zhao H., Golam Kibria M., Hu J. (2021). Polymeric Carbon Nitride-Based Photocatalysts
for Photoreforming
of Biomass Derivatives. Green Chem..

[334] Chen Z., Duan R., Xiao Y., Wei Y., Zhang H., Sun X., Wang S., Cheng Y., Wang X., Tong S., Yao Y., Zhu C., Yang H., Wang Y., Wang Z. (2022). Biodegradation
of Highly
Crystallized Poly­(Ethylene Terephthalate) through Cell Surface Codisplay
of Bacterial PETase and Hydrophobin. Nat. Commun..

[335] Sevilla M. E., Garcia M. D., Perez-Castillo Y., Armijos-Jaramillo V., Casado S., Vizuete K., Debut A., Cerda-Mejía L. (2023). Degradation of PET Bottles by an Engineered Ideonella
Sakaiensis PETase. Polymers (Basel).

[336] Qiu J., Chen Y., Zhang L., Wu J., Zeng X., Shi X., Liu L., Chen J. (2024). A Comprehensive
Review on Enzymatic
Biodegradation of Polyethylene Terephthalate. Environmental Research.

[337] Groseclose T. M., Nguyen H. B. (2025). Recent Advances in Enzyme Engineering
for Improved Deconstruction of Poly­(Ethylene Terephthalate) (PET)
Plastics. Commun. Mater..

[338] Ellis L. D., Rorrer N. A., Sullivan K. P., Otto M., McGeehan J. E., Román-Leshkov Y., Wierckx N., Beckham G. T. (2021). Chemical and Biological Catalysis
for Plastics Recycling
and Upcycling. Nat. Catal.

[339] Knott B. C., Erickson E., Allen M. D., Gado J. E., Graham R., Kearns F. L., Pardo I., Topuzlu E., Anderson J. J., Austin H. P., Dominick G., Johnson C. W., Rorrer N. A., Szostkiewicz C. J., Copié V., Payne C. M., Woodcock H. L., Donohoe B. S., Beckham G. T., McGeehan J. E. (2020). Characterization and Engineering
of a Two-Enzyme System
for Plastics Depolymerization. Proc. Natl. Acad.
Sci. U. S. A..

[340] Erickson E., Gado J. E., Avilán L., Bratti F., Brizendine R. K., Cox P. A., Gill R., Graham R., Kim D.-J., König G., Michener W. E., Poudel S., Ramirez K. J., Shakespeare T. J., Zahn M., Boyd E. S., Payne C. M., DuBois J. L., Pickford A. R., Beckham G. T., McGeehan J. E. (2022). Sourcing Thermotolerant
Poly­(Ethylene Terephthalate) Hydrolase Scaffolds from Natural Diversity. Nat. Commun..

[341] Yoshida S., Hiraga K., Takehana T., Taniguchi I., Yamaji H., Maeda Y., Toyohara K., Miyamoto K., Kimura Y., Oda K. (2016). A Bacterium That Degrades and Assimilates
Poly­(Ethylene Terephthalate). Science.

[342] Müller R.-J., Schrader H., Profe J., Dresler K., Deckwer W.-D. (2005). Enzymatic
Degradation of Poly­(Ethylene Terephthalate):
Rapid Hydrolyse Using a Hydrolase from T. Fusca. Macromol. Rapid Commun..

[343] Hajighasemi M., Nocek B. P., Tchigvintsev A., Brown G., Flick R., Xu X., Cui H., Hai T., Joachimiak A., Golyshin P. N., Savchenko A., Edwards E. A., Yakunin A. F. (2016). Biochemical and Structural Insights
into Enzymatic Depolymerization of Polylactic Acid and Other Polyesters
by Microbial Carboxylesterases. Biomacromolecules.

[344] Shalem A., Yehezkeli O., Fishman A. (2024). Enzymatic Degradation
of Polylactic Acid (PLA). Appl. Microbiol. Biotechnol..

[345] Masaki K., Kamini N. R., Ikeda H., Iefuji H. (2005). Cutinase-Like
Enzyme from the Yeast Cryptococcus Sp. Strain S-2 Hydrolyzes Polylactic
Acid and Other Biodegradable Plastics. Appl.
Environ. Microbiol..

[346] de Oliveira M. V. D., Calandrini G., da Costa C. H. S., da
Silva de Souza C. G., Alves C. N., Silva J. R. A., Lima A. H., Lameira J. (2025). Evaluating Cutinase from Fusarium Oxysporum as a Biocatalyst
for the Degradation of Nine Synthetic Polymer. Sci. Rep.

[347] Sullivan K. P., Werner A. Z., Ramirez K. J., Ellis L. D., Bussard J. R., Black B. A., Brandner D. G., Bratti F., Buss B. L., Dong X., Haugen S. J., Ingraham M. A., Konev M. O., Michener W. E., Miscall J., Pardo I., Woodworth S. P., Guss A. M., Román-Leshkov Y., Stahl S. S., Beckham G. T. (2022). Mixed Plastics Waste Valorization
through Tandem Chemical Oxidation and Biological Funneling. Science.

[348] Yan N. (2022). Recycling Plastic Using a Hybrid
Process. Science.

[349] Uekert T., Kasap H., Reisner E. (2019). Photoreforming
of Nonrecyclable
Plastic Waste over a Carbon Nitride/Nickel Phosphide Catalyst. J. Am. Chem. Soc..

[350] Bhattacharjee S., Guo C., Lam E., Holstein J. M., Rangel Pereira M., Pichler C. M., Pornrungroj C., Rahaman M., Uekert T., Hollfelder F., Reisner E. (2023). Chemoenzymatic Photoreforming: A Sustainable Approach
for Solar Fuel Generation from Plastic Feedstocks. J. Am. Chem. Soc..

[351] Yelin K., Shalem A., Meirovich M. M., Cohen A., Kornblum L., Cohen Y., Fishman A., Yehezkeli O. (2026). Photo-Driven Valorization of Polylactic Acid into Hydrogen
Fuel and Pyruvate Using a Biotic-Abiotic Configuration. Int. J. Hydrogen Energy.

[352] Zhang J., Sun Z., Chen Z., Tan X., Xiao X., Liang Y., Xiong J., Ni B.-J. (2026). Biotic-Abiotic
Hybrid System for Efficient Soluble Plastic Degradation. Nano Lett..

[353] Nelson N., Yocum C. F. (2006). Structure and Function
of Photosystems
I and II. Annu. Rev. Plant Biol..

[354] Song W., Zhang X., Li W., Li B., Liu B. (2025). Engineering Biotic-Abiotic Hybrid Systems for Solar-to-Chemical
Conversion. Chem..

[355] Jones W., Burnett J. W. H., Shi J., Howe R. F., Wang X. (2020). Improving Photocatalytic Energy Conversion
via NAD­(P)­H. Joule.

[356] Jain V., Pillai P. P. (2025). A Path to Perpetual
Chemical Synthesis *via* Photocatalytic Cofactor Regeneration. Chem. Sci..

[357] Browne L. B. F., Sudmeier T., Landis M. A., Allen C. S., Vincent K. A. (2024). Controlled
Biocatalytic Synthesis of a Metal Nanoparticle-Enzyme
Hybrid: Demonstration for Catalytic H2-Driven NADH Recycling. Angew. Chem., Int. Ed..

[358] Trotta C., Menendez Rodriguez G., Zuccaccia C., Macchioni A. (2024). Electrochemical NADH Regeneration
Mediated by Pyridine
Amidate Iridium Complexes Interconverting 1,4- and 1,6-NADH. ACS Catal..

[359] Steckhan E. (1986). Indirect Electroorganic Syntheses—A
Modern Chapter
of Organic Electrochemistry [New Synthetic Methods. Angew. Chem. Int. Ed. Engl..

[360] Ruppert R., Herrmann S., Steckhan E. (1987). Efficient Indirect
Electrochemical In-Situ Regeneration of Nadh:Electrochemically Driven
Enzymatic Reduction of Pyruvate Catalyzed by d-Ldh. Tetrahedron Lett..

[361] Hollmann F., Witholt B., Schmid A. (2002). [Cp*Rh­(Bpy)­(H2O)]­2+:
A Versatile Tool for Efficient and Non-Enzymatic Regeneration of Nicotinamide
and Flavin Coenzymes. Journal of Molecular Catalysis
B: Enzymatic.

[362] Hollmann F., Schmid A., Steckhan E. (2001). The First Synthetic
Application of a Monooxygenase Employing Indirect Electrochemical
NADH Regeneration. Angew. Chem., Int. Ed..

[363] Nam D. H., Lee S. H., Park C. B. (2010). CdTe, CdSe,
and
CdS Nanocrystals for Highly Efficient Regeneration of Nicotinamide
Cofactor Under Visible Light. Small.

[364] Lee S. H., Nam D. H., Kim J. H., Baeg J.-O., Park C. B. (2009). Eosin Y-Sensitized Artificial Photosynthesis
by Highly
Efficient Visible-Light-Driven Regeneration of Nicotinamide Cofactor. ChemBioChem..

[365] Liu J., Antonietti M. (2013). Bio-Inspired
NADH Regeneration by Carbon Nitride Photocatalysis
Using Diatom Templates. Energy Environ. Sci..

[366] Hong Y. H., Nilajakar M., Lee Y.-M., Nam W., Fukuzumi S. (2024). Artificial
Photosynthesis for Regioselective Reduction
of NAD­(P)+ to NAD­(P)H Using Water as an Electron and Proton Source. J. Am. Chem. Soc..

[367] Yun C.-H., Kim J., Hollmann F., Park C. B. (2022). Light-Driven
Biocatalytic Oxidation. Chem. Sci..

[368] Pueyo J. J., Gómez-Moreno C. (1992). Photochemical Regeneration of NADPH
Using the Enzyme Ferredoxin-NADP+ Reductase. Enzyme Microb. Technol..

[369] Medipally H., Guarneri A., Pospisil L., Franssen M. C. R., van Berkel W. J. H., Paul C. E., Nowaczyk M. M. (2023). Light-Driven
NADPH Cofactor Recycling by Photosystem I for Biocatalytic Reactions. ChemCatChem..

[370] Brown K. A., Wilker M. B., Boehm M., Hamby H., Dukovic G., King P. W. (2016). Photocatalytic Regeneration
of Nicotinamide
Cofactors by Quantum Dot-Enzyme Biohybrid Complexes. ACS Catal..

[371] Bachar O., Meirovich M. M., Zeibaq Y., Yehezkeli O. (2022). Protein-Mediated
Biosynthesis of Semiconductor Nanocrystals for Photocatalytic NAD­(P)­H
Regeneration and Chiral Amine Production. Angew.
Chem., Int. Ed..

[372] Yuan M., Kummer M. J., Milton R. D., Quah T., Minteer S. D. (2019). Efficient NADH Regeneration by a Redox Polymer-Immobilized
Enzymatic System. ACS Catal..

[373] Gao F., Liu G., Chen A., Hu Y., Wang H., Pan J., Feng J., Zhang H., Wang Y., Min Y., Gao C., Xiong Y. (2023). Artificial
Photosynthetic Cells with Biotic-Abiotic
Hybrid Energy Modules for Customized CO2 Conversion. Nat. Commun..

[374] Chakraborty I. N., Jain V., Roy P., Kumar P., Vinod C. P., Pillai P. P. (2024). Photocatalytic Regeneration
of Reactive
Cofactors with InP Quantum Dots for the Continuous Chemical Synthesis. ACS Catal..

[375] Xing F., Xue X., Li J., Liu J., Wang W., Dong W., Yuan H., Liu J. (2024). Sustainable
Photocatalytic Biological Cofactor Regeneration Fueled by Selective
Alcohol Oxidation over Polarized ZnIn_2_ S_4_. ACS Catal..

[376] Wang W., Liu J. (2023). Hydride Transfer for
NADH Regeneration:
From Nature, Beyond Nature. Adv. Energy and
Sustain Res..

[377] Yu X., Li H., Bao S., Wu Y., Li C., Xu Z., Xu J., Wang T., Liu J. (2026). Self-Assembled Protein
Cages in Living Bacterial Photocatalysis: Modular Design Achieves
Selective Regeneration of NADH and Efficient CO2 Fixation. Adv. Funct. Mater..

[378] Hambourger M., Gervaldo M., Svedruzic D., King P. W., Gust D., Ghirardi M., Moore A. L., Moore T. A. (2008). [FeFe]-Hydrogenase-Catalyzed H2 Production in a Photoelectrochemical
Biofuel Cell. J. Am. Chem. Soc..

[379] Castañeda-Losada L., Adam D., Paczia N., Buesen D., Steffler F., Sieber V., Erb T. J., Richter M., Plumeré N. (2021). Bioelectrocatalytic
Cofactor Regeneration
Coupled to CO _2_ Fixation in a Redox-Active Hydrogel for
Stereoselective C-C Bond Formation. Angew. Chem.,
Int. Ed..

[380] Guo J., Suástegui M., Sakimoto K. K., Moody V. M., Xiao G., Nocera D. G., Joshi N. S. (2018). Light-Driven Fine
Chemical Production in Yeast Biohybrids. Science.

[381] Chen N., Shen R., He T., Xi J., Zhao R., Du N., Yang Y., Yu L., Yuan Q. (2025). A Photosynthesis-derived
Bionic System for Sustainable Biosynthesis. Angew. Chem..

[382] Tian Y., Guo Z., He J., Xu D., Li W.-W., Cheng S., Song H. (2025). Light-Driven Eosin
Y-Ralstonia Eutropha Biohybrid for CO2 Conversion to Acetoin via Specific
Photo-Induced Electron Transfer and Metabolic Engineering. Journal of CO2 Utilization.

[383] Guan X., Erşan S., Xie Y., Park J., Liu C. (2024). Redox and Energy Homeostasis Enabled by Photocatalytic Material-Microbial
Interfaces. ACS Nano.

[384] Chen N., Xi J., He T., Shen R., Zhao R., Chi H., Yao J., Du N., Yu L., Zhang Y., Peng T., Liu T., Yuan Q. (2025). Beyond Natural
Synthesis via Solar-Decoupled Biohybrid Photosynthetic System. Chem..

[385] Lim C.-H., Ilic S., Alherz A., Worrell B. T., Bacon S. S., Hynes J. T., Glusac K. D., Musgrave C. B. (2019). Benzimidazoles
as Metal-Free and Recyclable Hydrides for CO2 Reduction to Formate. J. Am. Chem. Soc..

[386] Taylor S., Ninjoor V., Dowd D. M., Tappel A. L. (1974). Cathepsin
B2Measurement by Sensitive Fluorometric Ammonia Analysis. Anal. Biochem..

[387] Cohen Y., Shemesh M., Meirovich M. M., Zeibaq Y., Yehezkeli O. (2025). Light-Driven Bias-Free Photoelectrochemical
Cells for Hemin-Catalyzed Ammonia Generation. ACS Appl. Energy Mater..

[388] Büchsenschütz H. C., Vidimce-Risteski V., Eggbauer B., Schmidt S., Winkler C. K., Schrittwieser J. H., Kroutil W., Kourist R. (2020). Stereoselective Biotransformations
of Cyclic Imines in Recombinant Cells of Synechocystis Sp. PCC 6803. ChemCatChem..

[389] Dutta N., Bagchi D., Chawla G., Peter S. C. (2024). A Guideline
to Determine Faradaic Efficiency in Electrochemical CO2 Reduction. ACS Energy Lett..

[390] Kempler P. A., Nielander A. C. (2023). Reliable
Reporting of Faradaic Efficiencies
for Electrocatalysis Research. Nat. Commun..

[391] Ellis, D. S. ; Piekner, Y. ; Grave, D. A. ; Schnell, P. ; Rothschild, A. Considerations for the Accurate Measurement of Incident Photon to Current Efficiency in Photoelectrochemical Cells. Frontiers in Energy Research 2022, 9. 10.3389/fenrg.2021.726069.

[392] Lai Y.-H., Kato M., Mersch D., Reisner E. (2014). Comparison
of Photoelectrochemical Water Oxidation Activity of a Synthetic Photocatalyst
System with Photosystem II. Faraday Discuss..

[393] Kato M., Zhang J. Z., Paul N., Reisner E. (2014). Protein Film
Photoelectrochemistry of the Water Oxidation Enzyme Photosystem II. Chem. Soc. Rev..

[394] Yao Y., Wu Z., Zhao Z., Sun Z., Li T., Li Z., Lu X., Chen Z. (2025). Architecting
a Bias-Free Photoelectrochemical
CO2 Reduction System for Sustainable Formic Acid. Advanced Science.

[395] Kisch H., Bahnemann D. (2015). Best Practice in Photocatalysis:
Comparing Rates or Apparent Quantum Yields?. J. Phys. Chem. Lett..

[396] Anh Nguyen T. K., Trán-Phú T., Daiyan R., Minh Chau Ta X., Amal R., Tricoli A. (2024). From Plastic
Waste
to Green Hydrogen and Valuable Chemicals Using Sunlight and Water. Angew. Chem., Int. Ed..

[397] Kisch H. (2010). On the Problem of Comparing Rates
or Apparent Quantum Yields in Heterogeneous
Photocatalysis. Angew. Chem..

[398] Marcus R. A., Sutin N. (1985). Electron Transfers
in Chemistry and
Biology. Biochimica et Biophysica Acta (BBA)
- Reviews on Bioenergetics.

[399] Willner, I. ; Katz, E. Bioelectronics - An Introduction. In Bioelectronics; John Wiley & Sons, Ltd, 2005; pp 1–13. 10.1002/352760376X.ch1.

[400] Al-Lolage F. A., Bartlett P. N., Gounel S., Staigre P., Mano N. (2019). Site-Directed Immobilization of Bilirubin Oxidase for Electrocatalytic
Oxygen Reduction. ACS Catal..

[401] Algov I., Grushka J., Zarivach R., Alfonta L. (2017). Highly Efficient
Flavin-Adenine Dinucleotide Glucose Dehydrogenase Fused to a Minimal
Cytochrome C Domain. J. Am. Chem. Soc..

[402] Sharma A., Alfonta L. (2025). Engineering Strategies in Bio-Photoelectrochemical
Cells for Sustainable Energy and Environmental Applications. Chem. Commun..

[403] Gihaz S., Herzallh N. S., Cohen Y., Bachar O., Fishman A., Yehezkeli O. (2022). The Structure
of Bilirubin Oxidase
from Bacillus Pumilus Reveals a Unique Disulfide Bond for Site-Specific
Direct Electron Transfer. Biosensors.

[404] Kobayashi A., Taketa M., Sowa K., Kano K., Higuchi Y., Ogata H. (2023). Structure and Function
Relationship
of Formate De–hydrogenases: An Overview of Recent Progress. IUCrJ..

[405] Ichikawa K., Adachi T., Sowa K. (2025). Structural Bioelectrochemistry
of Direct Electron Transfer-Type Multimeric Dehydrogenases: Basic
Principle and Rational Strategies. Bioelectrochemistry.

[406] Chemla Y., Kaufman F., Amiram M., Alfonta L. (2024). Expanding
the Genetic Code of Bioelectrocatalysis and Biomaterials. Chem. Rev..

[407] Mostajabi Sarhangi S., Matyushov D. V. (2023). Electron
Tunneling in Biology: When
Does It Matter?. ACS Omega.

[408] Beratan D. N. (2021). Multiple Hops Move Electrons from
Bacteria to Rocks. Proc. Natl. Acad. Sci. U.
S. A..

[409] Huang K. C., Mukhopadhyay R., Wen B., Gitai Z., Wingreen N. S. (2008). Cell Shape and Cell-Wall Organization in Gram-Negative
Bacteria. Proc. Natl. Acad. Sci. U. S. A..

[410] Page C. C., Moser C. C., Chen X., Dutton P. L. (1999). Natural
Engineering Principles of Electron Tunnelling in Biological Oxidation-Reduction. Nature.

[411] Wang F., Gu Y., O’Brien J. P., Yi S. M., Yalcin S. E., Srikanth V., Shen C., Vu D., Ing N. L., Hochbaum A. I., Egelman E. H., Malvankar N. S. (2019). Structure
of Microbial Nanowires Reveals Stacked Hemes That Transport Electrons
over Micrometers. Cell.

[412] Moe A., Di Trani J., Rubinstein J. L., Brzezinski P. (2021). Cryo-EM Structure
and Kinetics Reveal Electron Transfer by 2D Diffusion of Cytochrome
c in the Yeast III-IV Respiratory Supercomplex. Proc. Natl. Acad. Sci. U. S. A..

[413] Wang F., Mustafa K., Suciu V., Joshi K., Chan C. H., Choi S., Su Z., Si D., Hochbaum A. I., Egelman E. H., Bond D. R. (2022). Cryo-EM Structure
of an Extracellular Geobacter OmcE Cytochrome Filament Reveals Tetrahaem
Packing. Nat. Microbiol.

[414] Zakizadeh Tabari, M. ; Hochbaum, A. I. Electron Transport across the Cell Envelope via Multiheme C-Type Cytochromes in Geobacter Sulfurreducens. Front. Chem. 2025, 13. 10.3389/fchem.2025.1621274. PMC12307469 40740311

[415] Black W. B., Perea S., Li H. (2023). Design, Construction,
and Application of Noncanonical Redox Cofactor Infrastructures. Curr. Opin. Biotechnol..

[416] Kenney K. C., LaFortune T. P., Weiss G. A. (2025). Nicotinamide Cofactor
Biomimetics: Design and Structure Activity Relationships. ACS Catal..

[417] Paul C. E., Arends I. W. C. E., Hollmann F. (2014). Is Simpler Better?
Synthetic Nicotinamide Cofactor Analogues for Redox Chemistry. ACS Catal..

[418] Orsi E., Hernández-Sancho J. M., Remeijer M. S., Kruis A. J., Volke D. C., Claassens N. J., Paul C. E., Bruggeman F. J., Weusthuis R. A., Nikel P. I. (2024). Harnessing Noncanonical Redox Cofactors
to Advance
Synthetic Assimilation of One-Carbon Feedstocks. Curr. Opin. Biotechnol..

[419] Holtmann D., Hollmann F. (2022). Is Water the Best Solvent for Biocatalysis?. Molecular Catalysis.

[420] Hollmann F., Arends I. W. C. E., Buehler K., Schallmey A., Bühler B. (2011). Enzyme-Mediated Oxidations for the Chemist. Green Chem..

[421] Wang X., Feng Y., Guo X., Wang Q., Ning S., Li Q., Wang J., Wang L., Zhao Z. K. (2021). Creating Enzymes
and Self-Sufficient Cells for Biosynthesis
of the Non-Natural Cofactor Nicotinamide Cytosine Dinucleotide. Nat. Commun..

[422] Köhler V., Wilson Y. M., Dürrenberger M., Ghislieri D., Churakova E., Quinto T., Knörr L., Häussinger D., Hollmann F., Turner N. J., Ward T. R. (2013). Synthetic
Cascades Are Enabled by Combining Biocatalysts with Artificial Metalloenzymes. Nature Chem..

[423] Zhang Z., Chiang H. T., Xia Y., Avakyan N., Sonani R. R., Wang F., Egelman E. H., De Yoreo J. J., Pozzo L. D., Tezcan F. A. (2025). Design of Light- and Chemically Responsive
Protein Assemblies through Host-Guest Interactions. Chem.

[424] Tel-Vered R., Yehezkeli O., Yildiz H. B., Wilner O. I., Willner I. (2008). Photoelectrochemistry
with Ordered CdS Nanoparticle/Relay
or Photosensitizer/Relay Dyads on DNA Scaffolds. Angew. Chem., Int. Ed..

[425] Ma K., Harris A. W., Cha J. N. (2018). DNA Assembled
Photoactive Systems. Curr. Opin. Colloid Interface
Sci..

[426] Lu N., Pei H., Ge Z., Simmons C. R., Yan H., Fan C. (2012). Charge Transport within
a Three-Dimensional DNA Nanostructure Framework. J. Am. Chem. Soc..

[427] Savage N. (2023). How to Lower Carbon Levels Using
Light. Nature.

[428] Edwards H., Yang Z., Xu P. (2020). Characterization of
Met25 as a Color Associated Genetic Marker in *Yarrowia Lipolytica*. Metabolic Engineering Communications.

[429] Bang S.-W., Clark D. S., Keasling J. D. (2000). Engineering
Hydrogen
Sulfide Production and Cadmium Removal by Expression of the Thiosulfate
Reductase Gene (phsABC) from Salmonella Enterica Serovar Typhimurium
in Escherichia Coli. Appl. Environ. Microbiol..

[430] Bang S.-W., Clark D. S., Keasling J. D. (2000). Engineering Hydrogen
Sulfide Production and Cadmium Removal by Expression of the Thiosulfate
Reductase Gene (phsABC) from Salmonella Enterica Serovar Typhimurium
in Escherichia Coli. Appl. Environ. Microbiol..

[431] Pande, V. ; Pandey, S. C. ; Sati, D. ; Bhatt, P. ; Samant, M. Microbial Interventions in Bioremediation of Heavy Metal Contaminants in Agroecosystem. Front. Microbiol. 2022, 13. 10.3389/fmicb.2022.824084. PMC9120775 35602036

[432] Ayub A., Wani A. K., Malik S. M., Ayub M., Chopra C., Singh R., Malik T. (2025). Harnessing Microbes
and Plants for Bioremediation of Heavy Metal Contaminants: Current
Paradigms and Future Perspectives. Environmental
Challenges.

[433] Umrania V. V. (2006). Bioremediation of Toxic Heavy Metals
Using Acidothermophilic
Autotrophes. Bioresour. Technol..

[434] Jaiswal, S. ; Shukla, P. Alternative Strategies for Microbial Remediation of Pollutants via Synthetic Biology. Front. Microbiol. 2020, 11. 10.3389/fmicb.2020.00808. PMC7249858 32508759

[435] Ruan Z., Chen K., Cao W., Meng L., Yang B., Xu M., Xing Y., Li P., Freilich S., Chen C., Gao Y., Jiang J., Xu X. (2024). Engineering Natural Microbiomes toward Enhanced Bioremediation by
Microbiome Modeling. Nat. Commun..

[436] Ding C., Ding Z., Liu Q., Liu W., Chai L. (2024). Advances in Mechanism for the Microbial Transformation
of Heavy Metals:
Implications for Bioremediation Strategies. Chem. Commun..

[437] Ren X., Zhao L., Shen J., Zhou P., Zhao K., Yuan C., Xing R., Yan X. (2025). Engineered Microbial
Platform Confers Resistance against Heavy Metals via Phosphomelanin
Biosynthesis. Nat. Commun..

[438] Diep, P. ; Mahadevan, R. ; Yakunin, A. F. Heavy Metal Removal by Bioaccumulation Using Genetically Engineered Microorganisms. Front. Bioeng. Biotechnol. 2018, 6. 10.3389/fbioe.2018.00157. PMC6215804 30420950

[439] Nnaji N. D., Anyanwu C. U., Miri T., Onyeaka H. (2024). Mechanisms
of Heavy Metal Tolerance in Bacteria: A Review. Sustainability.

[440] Faunce T., Styring S., Wasielewski M. R., Brudvig G. W., Rutherford A. W., Messinger J., Lee A. F., Hill C. L., deGroot H., Fontecave M., MacFarlane D. R., Hankamer B., Nocera D. G., Tiede D. M., Dau H., Hillier W., Wang L., Amal R. (2013). Artificial Photosynthesis
as a Frontier Technology for Energy Sustainability. Energy Environ. Sci..

[441] Loch-Temzelides, T. So Much for German Efficiency: A Warning for Green Policy Aspirations? Rice University’s Baker Institute for Public Policy, 2024. https://www.bakerinstitute.org/research/so-much-german-efficiency-warning-green-policy-aspirations.

[442] Schill W.-P., Guéret A., Roth A., Schmidt F. (2025). Germany Should
Accelerate Its Renewable Energy Transition. Commun. Earth Environ.

[443] Wei H., Liu B., Zhu T., Liu Y., Zhang L., Chen N., Li W. (2026). Advances in Green Technologies
for
Biocatalytic Synthesis of Chiral Compounds: From Enzymatic Catalysis
to Multidisciplinary Collaborative Innovation. Bioorganic Chemistry.

[444] Li Y., Wu S., Huangfu K., Huang H., Yu M., Qiu Y., Liu S. (2026). Light-Driven Artificial Photosynthesis: Integrating
Inorganic Photosensitizers with Biological Systems for Sustainable
Biosynthesis. J. Mater. Chem. A.

[445] Nishiyama H., Yamada T., Nakabayashi M., Maehara Y., Yamaguchi M., Kuromiya Y., Nagatsuma Y., Tokudome H., Akiyama S., Watanabe T., Narushima R., Okunaka S., Shibata N., Takata T., Hisatomi T., Domen K. (2021). Photocatalytic Solar
Hydrogen Production from Water on a 100-M2 Scale. Nature.

